# Good practices with injectables: digital technology for nursing education to control infections

**DOI:** 10.1590/0034-7167-2021-0716

**Published:** 2022-09-09

**Authors:** Camila Eugenia Roseira, Thais Roberto Magalhães Fittipaldi, Lívia Cristina Scalon da Costa, Darlyani Mariano da Silva, Ana Angélica Lima Dias, Rosely Moralez de Figueiredo

**Affiliations:** I Centro Paula Souza. São Carlos, São Paulo, Brazil; II Irmandade da Santa Casa de Misericórdia de Rio Claro. Rio Claro, São Paulo, Brazil; III Universidade Federal de São Carlos. São Carlos, São Paulo, Brazil; IV Universidade Federal de São João del-Rei. Divinópolis, Minas Gerais, Brazil

**Keywords:** Education, Nursing, Nursing Care, Infection Control, Educational Technology, Injections, Intravenous, Educación en Enfermería, Seguridad del Paciente, Control de Infecciones, Tecnología Educacional, Inyecciones Intravenosas, Educação em Enfermagem, Cuidados de Enfermagem, Controle de Infecções, Tecnologia Educacional, Injeções Intravenosas

## Abstract

**Objectives:**

to build, validate, implement, and evaluate an educational strategy for nursing professionals and students aiming at good practices in administrating injectable medications.

**Methods:**

methodological study for the development of an open course, without tutoring, in a virtual learning environment about good practices with injectable medications.

**Results:**

ten evaluators validated the educational material that supported the course “Good Practices with Injectables: actions for infection control” regarding objectives, structure, and relevance for the e-book and podcast. The evaluation by the target population (17 individuals) suggests that it is relevant and motivating. However, the forum may be the least attractive tool, and other studies should be conducted to identify its effectiveness as a tool for content retention in open courses.

**Conclusions:**

the course is open and has no mentoring for students and nursing professionals with validated educational material for this purpose may be used for nursing education in formal or informal settings.

## RESUMO

**Objetivos:**

construir, validar, implementar e avaliar uma estratégia educativa para profissionais e estudantes de Enfermagem visando às boas práticas na administração de medicações injetáveis.

**Métodos:**

estudo metodológico para o desenvolvimento de um curso aberto, sem tutoria, em ambiente virtual de aprendizagem sobre boas práticas com medicações injetáveis.

**Resultados:**

dez avaliadoras validaram o material educativo que embasou o curso “Boas Práticas com Injetáveis: ações para o controle de infecção” quanto a objetivos, estrutura e relevância para o e-book e podcast. A avaliação pela população-alvo (17 sujeitos) sugere que ele é relevante e motivador, contudo o fórum pode ser a ferramenta menos atrativa, devendo-se realizar outros estudos para identificar sua efetividade enquanto ferramenta para retenção de conteúdo em cursos abertos.

**Conclusões:**

o curso aberto e sem tutoria para estudantes e profissionais de enfermagem, com material educativo validado para esse fim, pode ser utilizado para educação em enfermagem, em ambientes formais ou informais.

## RESUMEN

**Objetivos:**

construir, validar, implementar y evaluar estrategia educativa para profesionales y estudiantes de Enfermería visando a buenas prácticas en la administración de medicamentos inyectables.

**Métodos:**

estudio metodológico para el desarrollo de curso abierto, sin tutoría, en ambiente virtual de aprendizaje sobre buenas prácticas con medicamentos inyectables.

**Resultados:**

diez evaluadoras validaron el material educativo que basó el curso “Buenas Prácticas con Inyectables: acciones para el control de infección” cuanto a objetivos, estructura y relevancia para el e-book y podcast. La evaluación por la población-objeto (17 sujetos) sugiere que él es relevante y motivador, pero el foro puede ser la herramienta menos atractiva, debiéndose realizar otros estudios para identificar su efectividad mientras herramienta para retención de contenido en cursos abiertos.

**Conclusiones:**

el curso abierto y sin tutoría para estudiantes y profesionales de enfermería, con material educativo validado para ese fin, puede ser utilizado para educación en enfermería, en ambientes formales o informales.

## INTRODUCTION

The administration of injectables is a frequent practice for nursing professionals, and, as a global phenomenon, the estimate is that millions of people need this care daily^(1)^. International Studies report an estimated rate per person of 1.1^(2)^ and 1.64^(3)^ injections per year.

The scenario regarding good practices with injectables is diverse. On the one hand, studies show that infection control measures such as hand hygiene (92.8%) and disinfection of vials and trays (91.4%) are referred by professionals who perform injectable medications by the subcutaneous route^(4)^; on the other hand, there are data evidencing the risk of transmission of pathogens via the bloodstream through inadequacies in practice with injectables. Examples are hepatitis C transmission among patients in a Taiwanese institution in 2017, with probable cause related to contamination of the environment and medications (the rate of injections was significantly higher for those who had positive serology - 4.4 injections/day)^(5)^ and the re-use of the same syringe to flushing in more than one patient per professional who did not know the risk of the practice^(6)^.

Although less frequently encountered when compared to central venous catheters^(7)^, peripheral catheter-related bloodstream infections have been reported in studies addressing bacteremia (of which 22.8% are related to peripheral catheters with a predominance of coagulase-negative staphylococci - 40.6%)^(8)^, infections caused by gram-positive (58%), gram-negative (35.8%) microorganisms, *Candida spp.* (6.2%), among others; and is related to risk of death when blood culture positive for *Staphylococcus aureus* ^(7)^.

Therefore, safe injection is one of the focuses of the prevention of infections^(9)^. However, the literature still points out inadequacies in the practice of this in health services^(10-11)^, such as low adherence to hand hygiene^(10-11)^ and the use of gloves for intravenous administration, absence of disinfection of vials and contamination of inputs during opening^(11)^. All this converges to the need for investment in educational strategies that address good practices with injectables to stimulate the insertion of a safe routine for both the patient and the professional and the environment.

Among these strategies, information, and communication technologies (ICT) are mentioned, which are efficient tools in the several topics of health education, such as interaction and critical thinking skills and collaboration of nursing students, promoting satisfaction and motivation for learning^(12-13)^.

Virtual learning environment (VLE) and the use of hypertexts are examples of ICTs applied to the context of practices with injectable medications^(14)^. Undergraduate nursing students are the target audience for courses focusing on these topics^(15-18)^, and they evaluate the educational strategies used as satisfactory.

The practices with injectable medications contemplate actions inserted both in the nursing professionals’ routine in work environments and the students’, and researchers point out gaps in the conduct of this practice, which indicates the need for improvement^(19)^.

The study^(19)^ demonstrates that it is necessary to present ideas or concepts to professionals related to the theme to bring new meanings to knowledge and practice, favoring biosafety and patient safety.

Among the theories that can support the presentation of educational material based on ICTs, is the Meaningful Learning Theory (MLT) developed by David Ausubel^(20)^. The MLT enables the re-elaboration of the individual’s prior knowledge based on the retention of what makes sense and is significant for the transformation of their practice, providing reflexive criticism about their conduct regarding good injectable practices.

Researchers did not find the elaboration of ICTs subsidized by the MLT for nursing professionals and students on practices for the prevention of infections associated with the preparation of injectables in health services in the literature.

## OBJECTIVES

To build, validate, implement, and evaluate an educational strategy for nursing professionals and students aiming at good practices in administrating injectable medications.

## METHODS

### Ethical aspects

The present work includes part of the thesis “Good Practices in injectable medications: a digital educational strategy for nursing professionals and students” approved by the Human Research Ethics Committee of the Federal University of São Carlos, according to resolution N^o^ 466/12 of the Ministry of health.

### Design, period, and place of study

This is a methodological study for elaboration, validation, implementation, and evaluation of an open course on good practices with injectables, between February 2019 and January 2021, which had its stage in an online format. The Portal of Open Courses of the Federal University of São Carlos distributed the course (Poca-UFSCar).

### Population or sample; inclusion and exclusion criteria

The stage related to contextual analysis had nursing professionals registered with the regional Nursing Council of the state of São Paulo and who developed/had developed activities related to nursing, linked to the Internet and Informatics (to complete and digitally submit the completed questionnaire), as this data was collected through an electronic questionnaire sent by that Council.

For the validation stage of the educational material, eighteen individuals were invited via email for convenience, selected according to the following criteria: have clinical experiences in infection control area related to health care or information and communication technology applied to nursing; or own publication/research on the topic; or have knowledge about the construction of questionnaires, or act professionally as a nurse.

Although there were no criteria for the enrollment of individuals in the course and its implementation on the platform, after the implementation of the course “Good Practices with Injectables: actions for infection control”, the professional or nursing student over 18 years old and who had fully completed it, were invited to evaluate it for convenience (which was verified through the data registered in Poca-UFSCar).

### Study protocol

The four stages of the study were based on guidelines for the construction of educational materials for new modalities of teaching^(21)^, especially distance education, to contemplate the proposed objectives.

In the first stage, “Contextual analysis,” the study identified the content to be emphasized in the educational material of the course based on the practice referred to about experiences with injectables of 1,295 nursing professionals (assistants, technicians, and nurses) from the state of São Paulo, Brazil (1,298 professionals contributed, but three were excluded because, according to answers in the questionnaire, they had not yet performed work activities in this area)^(19)^.

Data from 2021 indicate that the State has 657,428 registered professionals, according to data from the Federal Council of Nursing, and professionals with more than one registration can be double counted^(22)^.

Professionals filled out an online questionnaire with forty-five questions distributed among four domains (preparation of the environment, preparation of medications, drug administration, and care after drug administration), relying on a frequency gradation scale (always, often, sometimes, rarely, and never). This process occurred between September and December 2017 through an email sent by the Regional Nursing Council of the state of São Paulo to those registered^(19)^.

The choice of practices that would subsidize the educational material was due to the mentioned relevance of the action with injectables, that is, the severity of the breakdown of the aseptic barrier involved and the consequent risk of infection associated with this practice. For this purpose, the percentage of professionals who reported this practice was calculated according to frequency gradation.

For the second stage, “development of educational tools,” based on the findings of the previous stage, the study produced the main text to subsidize the content to be contemplated in the course on good practices with injectable medications, according to recommendations of the Centers for Disease Control and Prevention (CDC), World Health Organization (WHO), National Health Surveillance Agency (ANVISA), as well as articles published in the area.

The study created the e-book, podcast, five evaluative questions of the content for certificate issuance, and two open questions triggering discussion for the forum, according to models provided by Poca-UFSCar.

Poca-UFSCar is a free resource that hosts several areas of knowledge and whose contents can be accessed by anyone using any device. Self-registration is made after making a registration on the page of the portal and confirmation of the registered email, according to its availability.

The images in the e-book were either acquired from the iStock photo library or taken by the author himself, while the podcast music was acquired in the Film Music domain. Both the images and the music used were Royalty Free. The study obtained an open license, the Creative Commons, to contemplate the licenses acquired for images and music that were part of the educational material. Thus, the material becomes reproducible, allowing users to copy, translate or modify, but citing the source, without using it for commercial purposes.

After being elaborated, the educational material was forwarded for content validation. For this purpose, the study used the Health Education Content Validation Instrument (IVCES)^(23)^ for e-book and podcast validation; and the Content Validity Index (IVC)^(24)^ for content validation of questions.

The IVCES is a generalist instrument for the validation of educational content in health, composed of 18 items evaluating objectives, structure/presentation, and relevance, with three answer options (0 = disagree; 1 = partially agree, and 2 = totally agree) for each item^(23)^. The IVC was applied to verify clarity (writing enables understanding of the concept and is expressed coherently) and pertinence (questions reflect the concepts involved) using a Likert-type scale from 1 to 4 (1 = unclear/ not pertinent; 2 = item needs major review to be clear/pertinent; 3 = item needs minor review to be clear/pertinent; 4 = clear/pertinent item)^(24)^.

Therefore, the course was registered in the Dean’s Office of Extension Courses (Proc. n^o^ 23112.015764/2020 90), in August 2020, as an extension activity, in the modality of distance education; and its structure was according to Chart 1.

**Chart 1:** Structure of the course "Good Practices with Injectables: actions for infection control", São Carlos, São Paulo, Brazil, 2020

Tool	Objective	Content	Instrument used for validation
E-book “Good Practices with Injectables: actions for Infection Control”	To address infection control measures associated with the administration of injectables with a focus on patient safety.	Guarantee of a safe environment (surface preparation and tray for the safe preparation of injectables); Care for sterility of single-use inputs; Specifications for the use of multidose and single-dose vials; Moments of hand hygiene for practice with injectables (soap and water and 70% alcohol)	IVCES
Podcast “Good Practices with Injectable Medications: subsidies for self-care”	To address infection control measures associated with the administration of injectables with a focus on the safety of the professional.	Use of procedural gloves for intravenous injections and associated critical analysis of their use for intramuscular and subcutaneous administration of injectables; Adherence to the safety device attached to syringes and needles; No recapping of used needles; Care of the injury after an accident with piercing-cutting; Measures to prevent complications arising from a piercing-cutting accident for the professional.	IVCES
Questionnaire	Issue attendance certificate, when above 70% correct in five objective questions.	Hand hygiene; Presentation of diluent in ampoules for sole use; Critical analysis for glove use; *Flushing* for catheter maintenance and reduction of contamination in catheter lumen; Prohibition of reuse of disposable inputs; Prevention of accidents by piercing-cutting material; After-care following accidents by piercing-cutting material.	IVC
Forum	Stimulate interaction among course participants.	Sharing experiences on accidents by piercing-cutting material and training with syringes and needles with safety devices.	IVC

*IVCES - Health Education Content Validation Instrument; IVC - Content Validity Index.*

The evaluators received the educational material in its first version by email (e-book, podcast, and transcription), guidelines for completing the IVCES and IVC, and a Google Forms link for validation in September 2020. Among eighteen potential evaluators who were invited to participate in the validation of the educational material by electronic means, thirteen responded positively; and, at the deadline for return (21 days) of the completed form, ten returned it.

The third stage (“Implementation”) of the course called “Good Practices with Injectables: actions for infection control” took place in November 2020, at Poca-UFSCar, with the help of the specialized team; while its disclosure took place through social networks.

After the implementation of the course, it is indicated for evaluation. There are learning evaluation models that can be used to identify the effects of a course on a given population. Among these, Kirkpatrick’s^(25)^ stands out, proposing four levels of evaluation. They are: reaction (perception of the population about the course for learning), learning (it assesses knowledge acquisition through the course), behavior (it evaluates behavior change with the application of the course content), and results (it identifies the influence that the course taught has on the institution)^(25)^.

In the fourth stage, “Course evaluation” participants completed a structured questionnaire using a Google Forms link (November 2020 to January 2021). Researchers developed this instrument to identify the perception of the participants. It contained sociodemographic questions and twenty-five questions about the content, relevance, functionality, attractiveness of the educational tools, and motivation of the individual when taking the course; the answers were on a scale of agreement (from “totally agree” to “totally disagree”). There was also space for suggestions and observations.

The Google Forms link for access to the research and TCLE was disclosed using two paths: 1) along with the access link to the course through social networks, instant messaging applications, communication platforms; and 2) by message to those who have completed the course and viewed the e-book and podcast/transcription, to whom the invitation was inserted as “Communication” and in the “General Forum” field of the course page.

### Analysis of results and statistics

IVCES and IVC analyzed the content validation data. For the interpretation of IVCES, the study considered the sum of all domains (objectives ranging between 0 and 10; structure/presentation, between 0 and 20; and relevance, between 0 and 6): the higher the score (that is, the closer to 36), the more efficient the educational content^(23)^.

The CVI was calculated based on the sum of answers 3 or 4, divided by the sum of answers, and this value should be equal to or greater than 0.78 for the permanence of the item^(24)^.

After returning the instruments used for validation of the educational material and evaluation of the perception of the target population in the course “Good Practices with Injectables: actions for infection control,” the answers were coded, stored, and analyzed using descriptive statistics, relative and absolute frequency by Microsoft Excel.

## RESULTS

For contextual analysis, the study considered the following practices for the construction of educational tools: hand hygiene in the stages of preparation and administration of injectable medications (86.0%), hand hygiene with an alcohol solution before intravenous medication administration (66.0%), use of inputs with damaged packaging (7.6%), use of multidose vials for two or more patients (10.8%), use of gloves for the administration of intravenous, intramuscular and subcutaneous medications (80.5%, 65.4%, and 59.5%, respectively), reuse of occluders (8.3%) and storage of these in inappropriate places (2.3%), reuse of syringes for salinization of peripheral venous access in different patients (1.2%), recoating of needles after use (4.9%) and training for the use of needles with safety device (13.0%)^(19)^.

The first version of the e-book had ten pages; as for the podcast, four minutes, and 52 seconds. The questions built for certification of the course and those triggering discussion of the forum were forwarded to the evaluators in Word format, the Microsoft’s text editor.

Then, the educational material was sent for content validation.

The material was evaluated by ten women aged between 24 and 57 years old (36.1%±9.7), with 40% having a master’s degree, 20% having a doctorate, and 10% having a degree and conducting scientific initiation on the subject. Among the evaluators, 30% worked in health care services and 20% in the Hospital Infection Control Service (SCIH); and 30% were teachers (20%, in higher education; and 10%, technical course).

Table 1 shows the descriptive analysis of educational tools according to IVCES dimensions.

**Table 1:** Descriptive analysis of the scores of educational tools according to the health education content validation instrument, São Carlos, São Paulo, Brazil, 2020

Educational Tool	Domain	Maximum	Minimum	Average (DP)	Median	Mode
E-book “Good Practices with Injectables: actions for infection control”	Objective	10	8	9.7(±0.68)	10	10
	Structure/Presentation	20	18	19.6(±0.84)	20	20
	Relevance	6	4	5.8(±0.63)	6	6
	Total	36	30	35.1(±2.02)	36	36
Podcast “Good Practices with Injectable Medications: subsidies for self-care”	Objective	10	8	9.8(±0.63)	10	10
	Structure/Presentation	20	19	19.9(±0.32)	20	20
	Relevance	6	4	5.8(±0.63)	6	6
	Total	36	31	35.5(±1.6)	36	36

The main suggestions of the evaluators considered for the e-book and podcast referred to the inclusion of information and questions about good practices, in addition to the adequacy of the text for a provocative interactive language.

After evaluators’ suggestions, the last version of the e-book had twelve pages (including its transcription), and the podcast had five minutes.

Regarding the validation of the questionnaire for the evaluation of the course content, the evaluation identified CVI as equal to 1 for clarity and relevance both for the issues related to the issuance of the certificate and for those of the forum, that is, they were either clear and pertinent or needed a small textual review to achieve these qualities. The review suggestion was accepted although the questions already had CVI higher than 0.78.

The target population evaluated the course between November 20, 2020, and January 17, 2021, when the link to participate in the survey (via Google Forms) remained active, having received fifty-one contributions. By this time, 118 people had completed the questionnaire, of which eighty-five viewed the course material.

Twenty-five people in total did not take the course before answering the questionnaire, according to the activity completion report that monitors the activities of the enrolled students; four had not accessed the e-book or podcast/transcription and questionnaire for completion of the activity; four carried out the survey before taking the course, which was verified on the course page; and one did not accept that. After exclusion criteria, the study considered the perception of seventeen participants about the course.

Among the individuals who evaluated the course after implantation, 82.4% were female, aged between 18 and 57 years (30.7% 10.8); 94.1% reported coming from the State of Sao Paulo, and the others from Angola. The most used device for the course was the computer (70,6%). Among the participants, 58.8% said they were exclusively students, among whom 57.1% said they had a technical course; 28.6%, a graduate degree in Nursing; and 14.3%, a bachelor’s degree in Nursing. Among those who reported exclusively being professionals (29.4%), 80.0% reported being nurses and 20.0% nursing technicians. The participant could select more than one response option regarding their performance.

The perception of these individuals about the course Good Practices with Injectables” was summarized in Table 2.

**Table 2:** Perception of the target population about the course “Good Practices with Injectables”, São Carlos, São Paulo, Brazil, 2021

Item	I totally agree (%)	I partially agree (%)	I do not agree or disagree (%)	I disagree partially (%)	I totally disagree (%)
The content covered by the educational material is related to the objective of the course.	100.0	-	-	-	-
The content covered by the course is relevant to the practice with injectables in health services.	100.0	-	-	-	-
The content covered is helpful for course participants.	100.0	-	-	-	-
The content is exposed clearly and coherently.	100.0	-	-	-	-
The amount of suggested workload is sufficient to address the content.	82.4%	17.6%	-	-	-
The educational material has clear, objective, and dialogical language.	100.0	-	-	-	-
The course has a simple interface and is easy to manage.	94.1	5.9	-	-	-
The course presents itself attractively.	88.2	11.8	-	-	-
The sequence of the content is arranged logically.	94.1	5.9	-	-	-
The participant is encouraged to develop an active and independent role during the handling of the course material.	82.4	17.6	-	-	-
The participant is encouraged to reflect critically on the practice of injectables in health services.	100.0	-	-	-	-
The course instigates the theoretical deepening of the topic.	94.1	5.9	-	-	-
The educational material of the text “Good Practices with Injectables: actions for infection control” is distributed harmonically in the space limited to the page (text and images).	100.0	-	-	-	-
The educational material of the text “Good Practices with Injectables: actions for infection control” is distributed harmonically in the space limited to the page (text and images).	100.0	-	-	-	-
The educational material of the text “Good Practices with Injectables: actions for infection control” has an easy-to-read text.	100.0	-	-	-	-
The educational material of the text “Good Practices with Injectables: actions for infection control” conveys the message clearly and concisely.	100.0	-	-	-	-
The images of the educational material of the text “Good Practices with Injectables: actions for infection control” are significant for learning.	100.0	-	-	-	-
The links provided for access complement the course material.	100.0	-	-	-	-
The audio of the podcast “Good Practices with Injectable Medications: subsidies for self-care” presents sound fluidity and good diction.	94.1	5.9	-	-	-
The forum allows interaction between the participants of the course.	64.7	11.8	23.5	-	-
The forum favors learning and knowledge sharing among participants.	70.6	11.8	17.6	-	-
The activity proposed for the issuance of the certificate is pertinent to the contents worked in the material on Good Practices with Injectables.	100.0	-	-	-	-
The activity proposed for the issuance of the certificate provides the relationship between theory and practice with injectables.	94.1	5.9	-	-	-
The activity proposed for the issuance of the certificate is motivating and stimulates the resolution with enthusiasm.	82.3	11.8	5.9	-	-
Tools such as audio, links, and image loading feature good functionality.	88.2	-	5.9	5.9	-

*O “hífen” (-) representa dado numérico igual a 0.*

The course can be accessed through the link https://cursos.poca.ufscar.br/course/view.php?id=79.

## DISCUSSION

ICTs are strategies enhancers of learning in the development of VLE^(26)^ and have been used in nursing education^(27)^, whether in academic settings^(28-29)^, supporting partial-presential disciplines, whether in continuing education^(30)^. The relevance of VLE is in the autonomy of the individual, in the dialogical relationship, and in the possibility of deepening themes^(30)^.

To that end, the contextual analysis of the scenario, corresponding to the first stage of the present work, was of paramount importance, because, according to the MLT, it is necessary to identify the individual’s prior knowledge so that the content is significant, and can be added, transformed, or modified the contents that are already in their cognitive structure^(31)^.

Still on the MLT, when inserting the course in an open platform and validating the content of the material on good practices with injectables, the study considered two other conditions necessary for meaningful learning: the student’s predisposition to learn and a material with significant potential, respectively^(31)^.

In general, the perception of the course “Good Practices with Injectables: actions for infection control” was satisfactory. However, the tools for interaction between individuals, implemented through the forum, may have been flagged as the least attractive.

Because it is a course without mentoring, this finding is like that found in a study^(28)^ that addresses the low interactivity among nursing students participating in a VLE on Diabetes Mellitus, while differing from the attractiveness of this tool in courses with tutorials focused on continuing education with nursing staff^(30)^. Therefore, this point may demonstrate how challenging open courses without mentoring still are, as those who rely on it can be a preference on the part of students^(32)^, and the use of forums tends to be better accepted in courses with tutoring.

The interaction among participants is related to the better effectiveness of open courses, as well as the use of more than one tool addressing the content^(33)^, which was used in this course. Thus, with the offer of the possibility of monitoring the media through the e book and audio transcription, it was possible for this to be printed for storage and later use for use in non-computer environments^(21)^.

Regarding the use of podcasts as an educational tool, there is still low exploitation of this resource within formal education in Brazil^(34)^, while its effectiveness as a tool for meaningful learning depends on its application context^(35)^. As suggested in the literature^(36)^, the use of different educational resources in the context of online courses, when planned and produced in a standardized way, can provide better performances by the individuals.

Considering the questions about the motivation regarding the use of VLE, the study sought to identify how satisfied the participant was with the course, which, according to MLT, is something that influences learning and stimulates the continuity of the learning process^(37)^.

A study aimed to identify factors related to the motivation of undergraduate students of health courses from a private institution in the state of São Paulo to attend online disciplines in the Moodle environment, which had tools like the present course (text material in PDF, examinations based on multiple-choice questions with five alternatives, and interaction forum). As a result, the study identified that the predisposition to take a distance course, a suitable place for studies, and conciliation of studies, and use of other sites were factors that positively influenced the motivation of students^(32)^.

The course “Good Practices with Injectables: actions for infection control” used the following resources to achieve motivation: use of family language; the relationship between content and elements with potential for belonging to the cognitive structure of the participants; evaluation containing situations equivalent to those exemplified in the material (but not the same, as suggested by MLT); offer of a certificate; implementation of the course on an open platform, in which the student is responsible for time management and, consequently, recognizes his commitment to completion, that is, there is a will for achievement.

These findings corroborate the potential of VLE to generate participants’ autonomy, motivation for learning, and professional training^(38)^. With this, it is possible to use the course in its entirety or the material contemplating the content to complement the other methodologies of formal or informal education to subsidize the qualification of the workforce^(32)^.

### Study limitations

The course “Good Practices with Injectables: actions for infection control” was evaluated according to the perception of the target population, that is, at the first level of the Kirkpatrick model^(25)^. Given the importance and impact on subsequent levels, there is a need for its evaluation in the other classes to identify its effectiveness for content retention.

### Contributions to the field of Nursing

The course “Good Practices with Injectables: actions for infection control” consolidated at the end of this study can complement nursing education and professional update, both in a formal and informal environment, free of charge.

In addition, the materials contemplating the course can be downloaded and stored for later viewing or even be printed for use in environments without a computer.

## CONCLUSIONS

The course “Good Practices with Injectables: actions for infection control” was built based on validated educational material, distributed on a portal of free open courses without tutoring for students and nursing professionals.

Data from the perception of course participants point to the potential of this material in nursing education. The course was considered attractive and consistent with the practice conducted in health services. However, the forum seems to have had little use since it was not associated with the interaction tool by all participants.

This course can contribute to the development of new tools to improve nursing practices, from graduation to professional training, in addition to raising awareness of critical reflection on actions with injectable medications.

## SUPPLEMENTARY MATERIAL

ROSEIRA, Camila Eugenia. Good practices in injectable medications: a digital educational strategy for nursing professionals and students. Thesis (doctorate in Health Sciences) - Graduate Program in Nursing, Federal University of São Carlos. São Carlos, São Paulo. 2020. Available on: https://repositorio.ufscar.br/handle/ufscar/14189

0034-7167-reben-75-06-e20210716-sup01
