# Supplementary material for: Good practices with injectables: digital technology for nursing education to control infections
Source: Rev Bras Enferm. 2022 Sep 9;75(6):e20210716. doi: 10.1590/0034-7167-2021-0716 (PMC9749769; doi:10.1590/0034-7167-2021-0716)
Supplement: 0034-7167-reben-75-06-e20210716-sup01 [file 0034-7167-reben-75-06-e20210716-sup01.pdf]

UNIVERSIDADE FEDERAL DE SÃO CARLOS
CENTRO DE CIÊNCIAS BIOLÓGICAS E DA SAÚDE
PROGRAMA DE PÓS-GRADUAÇÃO EM ENFERMAGEM

CAMILA EUGENIA ROSEIRA

BOAS PRÁTICAS EM MEDICAÇÕES INJETÁVEIS: uma estratégia educacional digital para profissionais e estudantes de enfermagem

SÃO CARLOS -SP
2021

CAMILA EUGENIA ROSEIRA

BOAS PRÁTICAS EM MEDICAÇÕES INJETÁVEIS: uma estratégia educacional digital para profissionais e estudantes de enfermagem

Tese apresentada ao Programa de Pós-Graduação em Enfermagem da Universidade Federal de São Carlos, para obtenção do título de doutora em Ciências da Saúde.

Orientadora: Dra. Rosely Moralez de Figueiredo

São Carlos-SP
2021

UNIVERSIDADE FEDERAL DE SÃO CARLOS

Centro de Ciências Biológicas e da Saúde
Programa de Pós-Graduação em Enfermagem

Folha de Aprovação

Defesa de Tese de Doutorado da candidata Camila Eugenia Roseira, realizada em 26/02/2021.

Comissão Julgadora:

Profa. Dra. Rosely Moralez de Figueiredo (UFSCar)

Profa. Dra. Adriana Maria da Silva Felix (FCMSC-SP)

Profa. Dra. Ana Angélica Lima Dias (FUNREI)

Profa. Dra. Aline Helena Appoloni Eduardo (UFSCar)

Profa. Dra. Silvia Helena Zem Mascarenhas (UFSCar)

O presente trabalho foi realizado com apoio da Coordenação de Aperfeiçoamento de Pessoal de Nível Superior - Brasil (CAPES) - Código de Financiamento 001.
O Relatório de Defesa assinado pelos membros da Comissão Julgadora encontra-se arquivado junto ao Programa de Pós-Graduação em Enfermagem.

Dedico este trabalho a todos os profissionais e estudantes de enfermagem, em especial, aos alunos do curso técnico em enfermagem da Etec Paulino Botelho

AGRADECIMENTO

À Deus, pela proteção, força e oportunidade de aprendizado, ao longo deste caminho.

Aos meus pais, José e Helena, por todo apoio, cuidado e companheirismo durante toda minha trajetória acadêmica.

À minha orientadora, Profa. Dra. Rosely, pela confiança, paciência e acolhimento às minhas dúvidas e colocações com carinho, atenção e conhecimento.

Às amigas da pós-graduação, com quem compartilhei momentos de muito aprendizado, e com quem pude contar durante o desenvolvimento deste trabalho.

Às amigas Thais Magalhães, Darlyani Mariano e Livia Scalon, em especial, pela parceria, solicitude, prontidão, presença e incentivo.

Às amigas e companheiras de trabalho da Etec Paulino Botelho, pela parceira, compreensão e por ajudarem na divulgação do curso fruto deste trabalho, em especial Ana Paula Martinelli, Cristiane Leite, Dilceli Valério, Dirlei Martins e Glaucia Negrè.

À professora dra Fabiana Orlandi, pela disponibilidade em auxiliar durante o desenvolvimento deste trabalho.

Aos profissionais do Programa de Pós-Graduação em Enfermagem, por toda disponibilidade e paciência ao longo deste processo.

Aos juízes e avaliadoras que aceitaram participar deste trabalho, pela disponibilidade e por compartilharem seu conhecimento e atenção.

Aos participantes da pesquisa, pela disponibilidade e atenção, possibilitando a coleta de dados.

A toda a equipe do Portal de Cursos Abertos da Universidade Federal de São Carlos, em especial Cleide e Priscila, com quem pude contar durante todo o desenvolvimento do material educativo.

Às Profas. Dras. Adriana, Ana Angélica, Aline e Silvia, que compuseram a banca de defesa, pela disponibilidade, atenção e contribuições para com este trabalho.

Ao Prof. Dr. Mauro Antônio Pires Dias da Silva pelo apoio à pesquisa durante a gestão do Conselho Regional de Enfermagem do estado de São Paulo (2017) pela divulgação do questionário para coleta de dados.

À Coordenação de Aperfeiçoamento de Pessoal de Nível Superior pelo financiamento do primeiro artigo publicado a partir dos dados deste trabalho.

RESUMO

As Boas Práticas com Medicações Injetáveis envolvem medidas que previnem danos à saúde do paciente e do profissional, em particular infecções. Para estimular as práticas seguras incentiva-se a disponibilização de materiais educativos abordando este tema, suportada pela Tecnologia de Informática e Comunicação (TIC). Dessa forma, objetivou-se construir e validar material educativo para profissionais e estudantes de enfermagem visando às boas práticas na administração de medicações injetáveis. Trata-se de estudo com delineamento metodológico múltiplo, o qual consistiu na construção e validação do Questionário sobre Boas Práticas com Medicações Injetáveis (QBMI), estruturado em quatro domínios para a contextualização sobre este cenário. O QBMI foi distribuído eletronicamente aos profissionais inscritos no Conselho Regional de Enfermagem de São Paulo. No total, 1925 profissionais participaram desta etapa. A maioria dos respondentes (41,62%) pertencia ao nível médio e atuante em serviço hospitalar (57,45%). De forma geral, as respostas configuraram-se dentro das práticas seguras com injetáveis, contudo, algumas ações referidas apresentaram risco de infecção, uma vez que a frequência "sempre" foi assinalada por: 1,24% para a reutilização da mesma seringa para salinização de acessos venosos de pacientes diferentes; 8,26% para reuso de oclusores de dupla-via e 2,32% para armazenamento inapropriado destes; 80,46% para uso de luvas para administração de medicações endovenosas; 12,97% para participação em treinamentos para manuseio de agulhas e cateteres com dispositivos de segurança e 20,08% para o transporte de seringas e agulhas após utilização, desprotegidas. Estes dados subsidiaram a construção e validação do curso gratuito, aberto e sem tutoria "Boas Práticas com Injetáveis: ações para o controle de infecção", composto por ebook (sobre higienização das mãos e ações para o controle de infecção, como a proibição de reutilização de insumos), podcast contendo ações para o autocuidado do profissional (uso de luvas, participação em treinamentos e prevenção de acidentes com perfurocortantes), fórum para estimular a interação entre os participantes e um questionário de avaliação para emissão de certificado. O material do curso foi validado por conteúdo, sendo considerado adequado por todas as avaliadoras, enquanto as questões foram consideradas claras e pertinentes. Para avaliar a reação ao curso, após disponibilização deste no Portal de Cursos Abertos (PoCA), 17 participantes deixaram sua percepção sobre ele, apontando para a relevância temática e aceitabilidade das ferramentas (sendo o fórum, possivelmente, a ferramenta menos atrativa). Com isso, identificou-se que a maioria dos profissionais de enfermagem referem práticas seguras com medicações injetáveis, contudo, há ações que devem ser consideradas a fim de subsidiar a prática segura. Para tanto, o curso construído, validado e disponibilizado de forma gratuita e aberta pode ser utilizado enquanto ferramenta de apoio por todos os envolvidos na área.

Palavras-chave: Estudos de Validação. Cuidados de Enfermagem. Injeções Intramusculares. Injeções Intravenosas. Educação a Distância.

ABSTRACT

Safe Injection Practices involve measures that prevent damage to the health of the patient and the professional, such as infections. To stimulate safe practices, the availability of educational materials addressing this theme, supported by Information and Communication Technology (ICT). The objective was to build and validate educational material for nursing professionals and students aiming at good practices in the administration of injectable medications. This study consisted of the construction and validation of the Safe Injection Practices Questionnaire (SIPQ), structured in four domains for contextualization of this scenario. The SIPQ was distributed electronically to professionals enrolled in the Regional Nursing Council of Sao Paulo. In total, 1925 professionals participated in this stage. Most professionals (41.62%) it belonged to the middle level and was active in hospital service (57.45%). In general, the answers were configured within safe practices with injectables, however, some actions mentioned presented risk to infection, since the frequency "always" was indicated by: 1.24% for the reuse of the same syringe for salinization of venous accesses of different patients; 8.26% for reuse of intravenous catheter extension plugs and 2.32% for inappropriate storage of these; 80.46% for the use of gloves for the administration of intravenous injections; 12.97% for participation in training for the handling of needles and catheters with safety devices and 20.08% for the transport of syringes and needles unprotected after use. These data supported the construction and validation of the open on-line course and without tutoring " Safe Injection Practices: actions for infection control", composed of ebook (hand hygiene and actions for infection control, such as the prohibition of reuse of inputs), podcast containing actions for the professional healthcare (use of gloves, participation in training and prevention of accidents with sharps) , forum to stimulate interaction between participants and an evaluation questionnaire for certificate issuance. The course material was validated by content, being considered adequate by all evaluators, while the questions were considered clear and pertinent. To evaluate the reaction to the course, after making this available in the Portal de Cursos Abertos (PoCA), 17 participants left their perception about it, pointing to the thematic relevance and acceptability of the tools (the forum being possibly the least attractive tool). Therefore, it was noted that most nursing professionals report Safe Injection Practices, however, there are actions that should be considered in order to subsidize safe practice. To this do so, the course built, validated and made available free of charge and openly can be used as a support tool by all those involved in the area.

Keyword: Nursing Care. Injections, Intramuscular. Injections, Intravenous. Education, Distance.

LISTA DE FIGURAS

Figura 1 - Etapas da elaboração do material educativo sobre Boas Práticas em Medicações Injetáveis conforme sugerido por Filatro e Cairo (2015). 30
Figura 2 – Esquema sobre os componentes das Boas Práticas com Injetáveis a serem abordados no material educativo proposto. São Carlos, SP, 2020. 102
Figura 3 - Capa e primeira página do ebook “Boas Práticas com Injetáveis: ações para o controle de infecção: elaboração e validação por conteúdo”. São Carlos, SP, 2020. 104
Figura 4 - Modelo estrutural das questões obrigatórias para conclusão do curso "Boas Práticas com Injetáveis: ações para o controle de infecção". São Carlos, SP, 2020. 106
Figura 5 - Adequação de figura após validação de conteúdo do ebook “Boas Práticas com Injetáveis: ações para o controle de infecção” a relevância, segundo o Instrumento de Validação de Conteúdo Educativo em Saúde. São Carlos, SP, 2020..........115
Figura 6 - Página inicial do PoCA, acessada via navegador Google Chrome. São Carlos, SP, 2021. 126
Figura 7 - Página inicial do curso "Boas Práticas com Injetáveis: ações para o controle de infecção". São Carlos, SP, 2021. 127
Figura 8 - Interface da apresentação do curso "Boas Práticas com Injetáveis: ações para o controle de infecção"...128
Figura 9 - Página do curso "Boas Práticas com Injetáveis: ações para o controle de infecção". 128
Figura 10 - Página do curso "Boas Práticas com Injetáveis: ações para o controle de infecção". 129
Figura 11 - Página do curso "Boas Práticas com Injetáveis: ações para o controle de infecção". 129
Figura 12 - Página do curso "Boas Práticas com Injetáveis: ações para o controle de infecção". 130
Figura 13 - Página do curso "Boas Práticas com Injetáveis: ações para o controle de infecção". 130
Figura 14 - Página do curso "Boas Práticas com Injetáveis: ações para o controle de infecção". 131
Figura 15 - Página do curso "Boas Práticas com Injetáveis: ações para o controle de infecção". 131
Figura 16 - Página do curso "Boas Práticas com Injetáveis: ações para o controle de infecção". 132
Figura 17 - Página do curso "Boas Práticas com Injetáveis: ações para o controle de infecção". 132
Figura 18 - Página do curso "Boas Práticas com Injetáveis: ações para o controle de infecção". 133
Figura 19 - Página do curso "Boas Práticas com Injetáveis: ações para o controle de infecção". 133
Figura 20 - Página do curso "Boas Práticas com Injetáveis: ações para o controle de infecção". 134
Figura 21 - Página do curso "Boas Práticas com Injetáveis: ações para o controle de infecção". 134

Figura 22 - Página do curso "Boas Práticas com Injetáveis: ações para o controle de infecção". 135
Figura 23 - Página do curso "Boas Práticas com Injetáveis: ações para o controle de infecção". 135
Figura 24 - Página do curso "Boas Práticas com Injetáveis: ações para o controle de infecção". 136
Figura 25 - Página do curso "Boas Práticas com Injetáveis: ações para o controle de infecção". 136
Figura 26 - Licença do material educativo do curso "Boas Práticas com Injetáveis". 137
Figura 27 - Convite deixado na plataforma para participar da pesquisa sobre a percepção do curso "Boas Práticas com Injetáveis"..138

LISTA DE QUADROS

Quadro 1 - Legenda de valores para cálculo do Índice de Validade de Conteúdo do instrumento para coleta de dados. São Carlos, SP, Brasil, 2017..33
Quadro 2 - Modificações realizadas no Questionário para Identificação das Boas Práticas com Medicações Injetáveis..52
Quadro 2 - Modificações realizadas no Questionário para Identificação das Boas Práticas com Medicações Injetáveis..53
Quadro 2 - Modificações realizadas no Questionário para Identificação das Boas Práticas com Medicações Injetáveis..54
Quadro 2 - Modificações realizadas no Questionário para Identificação das Boas Práticas com Medicações Injetáveis..55
Quadro 2 - Modificações realizadas no Questionário para Identificação das Boas Práticas com Medicações Injetáveis..56
Quadro 2 - Modificações realizadas no Questionário para Identificação das Boas Práticas com Medicações Injetáveis..57
Quadro 1 - Adequações realizadas no *ebook* “Boas Práticas com Injetáveis: ações para o controle de infecção” para atender às sugestões das avaliadoras sobre a adequação aos objetivos da ferramenta educativa. São Carlos, SP, 2020………………………..111
Quadro 4 - Adequações realizadas no ebook “Boas Práticas com Injetáveis: ações para o controle de infecção: elaboração e validação por conteúdo” para atender às sugestões das avaliadoras sobre a adequação a estrutura da ferramenta educativa. São Carlos, SP, 2020........................113
Quadro 4 - Adequações realizadas no ebook “Boas Práticas com Injetáveis: ações para o controle de infecção: elaboração e validação por conteúdo” para atender às sugestões das avaliadoras sobre a adequação a estrutura da ferramenta educativa. São Carlos, SP, 2020........................114
Quadro 5 - Adequações realizadas no podcast “Boas Práticas com Medicações Injetáveis: subsídios para o autocuidado” para atender às sugestões das avaliadoras sobre a adequação a estrutura da ferramenta educativa. São Carlos, SP, 2020..118
Quadro 6 - Escaleta do podcast “Boas Práticas com Medicações Injetáveis: subsídios para o autocuidado” pós validação do conteúdo pelas avaliadoras. São Carlos, SP, 2020...................120
Quadro 7 - Adequação da redação do feedback da primeira questão avaliativa do curso "Boas Práticas com Injetáveis", São Carlos, SP, 2020...121
Quadro 8 - Adequação da redação da segunda questão avaliativa do curso "Boas Práticas com Injetáveis", São Carlos, SP, 2020...122
Quadro 9 - Adequação da redação da terceira questão avaliativa do curso "Boas Práticas com Injetáveis", São Carlos, SP, 2020...123
Quadro 10 - Adequação da redação do feedback da terceira questão avaliativa do curso "Boas Práticas com Injetáveis", São Carlos, SP, 2020...123
Quadro 11 - Adequação da redação da quarta questão avaliativa do curso "Boas Práticas com Injetáveis", São Carlos, SP, 2020...124
Quadro 12 - Adequação da redação da alternativa ‘c’ da quinta questão avaliativa do curso "Boas Práticas com Injetáveis", São Carlos, SP, 2020...125

LISTA DE TABELAS

Tabela 1 - Avaliação da concordância da adequação dos itens do domínio "preparo do ambiente” do Questionário para Identificação das Boas Práticas com Medicações Injetáveis. São Carlos, SP, Brasil, 2017..46
Tabela 2 - Avaliação da concordância da adequação dos itens do domínio "preparo das medicações” do Questionário para Identificação das Boas Práticas com Medicações Injetáveis. São Carlos, SP, Brasil, 2017..47
Tabela 3 - Avaliação da concordância da adequação dos itens do domínio "administração medicamentosa” do Questionário para Identificação das Boas Práticas com Medicações Injetáveis. São Carlos, SP, Brasil, 2017..48
Tabela 3 - Avaliação da concordância da adequação dos itens do domínio "administração medicamentosa” do Questionário para Identificação das Boas Práticas com Medicações Injetáveis. São Carlos, SP, Brasil, 2017..49
Tabela 4 - Avaliação da concordância da adequação dos itens do domínio "cuidados após administração medicamentosa” do Questionário para Identificação das Boas Práticas com Medicações Injetáveis. São Carlos, SP, Brasil, 2017...50
Tabela 5 - Pontuação do instrumento QBMI utilizado para coleta de dados referente às práticas de medicações injetáveis segundo número de itens. São Carlos-SP, Brasil. 2018...58
Tabela 6 - Frequência referida de atividades relacionadas ao preparo do ambiente pelos profissionais do estado de São Paulo. São Carlos, SP, 2018..64
Tabela 6 - Frequência referida de atividades relacionadas ao preparo do ambiente pelos profissionais do estado de São Paulo. São Carlos, SP, 2018..65
Tabela 8 - Frequência referida de atividades relacionadas à administração das medicações injetáveis pelos profissionais do estado de São Paulo. São Carlos, SP, 2018...............66
Tabela 8 - Frequência referida de atividades relacionadas à administração das medicações injetáveis pelos profissionais do estado de São Paulo. São Carlos, SP, 2018...............67
Tabela 8 - Frequência referida de atividades relacionadas à administração das medicações injetáveis pelos profissionais do estado de São Paulo. São Carlos, SP, 2018...............68
Tabela 9 - Frequência referida de atividades relacionadas aos cuidados após a administração medicamentosa pelos profissionais do estado de São Paulo. São Carlos, SP, 2018....69
Tabela 10 - Análise descritiva de escore de domínios e do instrumento segundo graus de instrução profissionais. São Carlos, SP, 2018...71
Tabela 11 - Diferença significativa entre os graus se instrução de acordo com componentes do instrumento de coleta de dados sobre preparo do ambiente para administração de medicações injetáveis segundo frequência referida. São Carlos, SP, 2018...................73
Tabela 12 - Diferença significativa entre os graus se instrução de acordo com componentes do instrumento de coleta de dados sobre preparo das medicações para administração de medicações injetáveis segundo frequência referida. São Carlos, SP, 2018...................75
Tabela 13 - Diferença significativa entre os graus se instrução de acordo com componentes do instrumento de coleta de dados sobre administração medicamentosa segundo frequência referida. São Carlos, SP, 2018..77
Tabela 13 - Diferença significativa entre os graus se instrução de acordo com componentes do instrumento de coleta de dados sobre administração medicamentosa segundo frequência referida. São Carlos, SP, 2018..78
Tabela 13 - Diferença significativa entre os graus se instrução de acordo com componentes do instrumento de coleta de dados sobre administração medicamentosa segundo frequência referida. São Carlos, SP, 2018..79
Tabela 14 - Diferença significativa entre os graus se instrução de acordo com componentes do instrumento de coleta de dados sobre cuidados após administração medicamentosa segundo frequência referida. São Carlos, SP, 2018..81

Tabela 15 - Análise descritiva de escore de domínios e do instrumento segundo tempo de atuação profissional. São Carlos, SP, 2018..83
Tabela 16 - Diferença significativa entre o tempo de atuação em anos na área com componentes do instrumento de coleta de dados sobre as etapas para a administração de medicações injetáveis segundo frequência referida. São Carlos, SP, 2018.................85
Tabela 16 - Diferença significativa entre o tempo de atuação em anos na área com componentes do instrumento de coleta de dados sobre as etapas para a administração de medicações injetáveis segundo frequência referida. São Carlos, SP, 2018.................86
Tabela 17 - Análise descritiva de escore de domínios e do instrumento entre serviços hospitalares e não hospitalares. São Carlos, SP, 2018..88
Tabela 18 - Diferença significativa entre instituições hospitalares e outros serviços de acordo com componentes do instrumento de coleta de dados sobre preparo do ambiente para administração de medicações injetáveis segundo frequência referida. São Carlos, SP, 2018...90
Tabela 19 - Diferença significativa entre instituições hospitalares e outros serviços de acordo com componentes do instrumento de coleta de dados sobre preparo de medicações para administração de injetáveis segundo frequência referida. São Carlos, SP, 2018...92
Tabela 20 - Diferença significativa entre instituições hospitalares e outros serviços de acordo com componentes do instrumento de coleta de dados sobre administração de injetáveis segundo frequência referida. São Carlos, SP, 2018..94
Tabela 20 - Diferença significativa entre instituições hospitalares e outros serviços de acordo com componentes do instrumento de coleta de dados sobre administração de injetáveis segundo frequência referida. São Carlos, SP, 2018..95
Tabela 20 - Diferença significativa entre instituições hospitalares e outros serviços de acordo com componentes do instrumento de coleta de dados sobre administração de injetáveis segundo frequência referida. São Carlos, SP, 2018..96
Tabela 20 - Diferença significativa entre instituições hospitalares e outros serviços de acordo com componentes do instrumento de coleta de dados sobre administração de injetáveis segundo frequência referida. São Carlos, SP, 2018...97
Tabela 20 - Diferença significativa entre instituições hospitalares e outros serviços de acordo com componentes do instrumento de coleta de dados sobre administração de injetáveis segundo frequência referida. São Carlos, SP, 2018...98
Tabela 21 - Diferença significativa entre instituições hospitalares e outros serviços de acordo com componentes do instrumento de coleta de dados sobre cuidados após administração medicamentosa segundo frequência referida. São Carlos, SP, 2018.........................100
Tabela 21 - Diferença significativa entre instituições hospitalares e outros serviços de acordo com componentes do instrumento de coleta de dados sobre cuidados após administração medicamentosa segundo frequência referida. São Carlos, SP, 2018.........................101
Tabela 22 - Análise descritiva dos escores do ebook “Boas Práticas com Injetáveis: ações para o controle de infecção” aos objetivos, segundo o Instrumento de Validação de Conteúdo Educativo em Saúde. São Carlos, SP, 2020...110
Tabela 23 - Frequência relativa sobre concordância das avaliadoras sobre a adequabilidade do ebook “Boas Práticas com Injetáveis: ações para o controle de infecção” aos objetivos, segundo o Instrumento de Validação de Conteúdo Educativo em Saúde. São Carlos, SP, 2020...110
Tabela 24 - Frequência relativa sobre concordância das avaliadoras sobre a adequabilidade do ebook “Boas Práticas com Injetáveis: ações para o controle de infecção” a estrutura, segundo o Instrumento de Validação de Conteúdo Educativo em Saúde. São Carlos, SP, 2020...111
Tabela 25 - Frequência relativa sobre concordância das avaliadoras sobre a adequabilidade do ebook “Boas Práticas com Injetáveis: ações para o controle de infecção” a relevância,

segundo o Instrumento de Validação de Conteúdo Educativo em Saúde. São Carlos, SP, 2020....115
Tabela 26 - Análise descritiva dos escores do podcast “Boas Práticas com Medicações Injetáveis: subsídios para o autocuidado” segundo o Instrumento de Validação de Conteúdo Educativo em Saúde. São Carlos, SP, 2020....116
Tabela 27 - Frequência relativa da concordância das avaliadoras sobre a adequabilidade do podcast “Boas Práticas com Medicações Injetáveis: subsídios para o autocuidado” aos objetivos, segundo o Instrumento de Validação de Conteúdo Educativo em Saúde. São Carlos, 2020....116
Tabela 28 - Frequência relativa sobre concordância das avaliadoras sobre a adequabilidade do podcast “Boas Práticas com Medicações Injetáveis: subsídios para o autocuidado” a estrutura, segundo o Instrumento de Validação de Conteúdo Educativo em Saúde. São Carlos, 2020....117
Tabela 29 - Frequência relativa da concordância das avaliadoras sobre a adequabilidade do podcast “Boas Práticas com Medicações Injetáveis: subsídios para o autocuidado” a relevância, segundo o Instrumento de Validação de Conteúdo Educativo em Saúde. São Carlos, 2020....119
Tabela 30 - Frequência relativa segundo gradação de concordância da percepção da população alvo sobre o curso "Boas Práticas com Injetáveis: ações para o controle de infecção". São Carlos, SP, 2021....140
Tabela 30 - Frequência relativa segundo gradação de concordância da percepção da população alvo sobre o curso "Boas Práticas com Injetáveis: ações para o controle de infecção". São Carlos, SP, 2021....141
Tabela 30 - Frequência relativa segundo gradação de concordância da percepção da população alvo sobre o curso "Boas Práticas com Injetáveis: ações para o controle de infecção". São Carlos, SP, 2021....14

LISTA DE SIGLAS

ANVISA – Agência Nacional de Vigilância Sanitária
AVA – Ambiente Virtual de Aprendizagem
CDC – Centers for Disease Control and Prevention
Coren-SP – Conselho Regional de Enfermagem do Estado de São Paulo
CVP – Cateter Venoso Periférico
EAD – Educação a Distância
EPI – Equipamento de Proteção Individual
EV – Endovenosa
HM – Higienização das Mãos
IAVP – Infecção Relacionada a Acesso Vascular Periférico
ICS – Infecção de Corrente Sanguínea
IRAS – Infecção Relacionada a Assistência à Saúde
IM – Intramuscular
IVC – Índice de Validade de Conteúdo
IVCES - Instrumento de Validação de Conteúdo Educativo em Saúde
MIT – Instituto de Tecnologia de Massachusetts
Moodle – Modular Object-Oriented Dynamic Learning Environment
PDF – Portable Document Format
PoCA – Portal de Cursos Abertos
SC – Subcutânea
SCIH – Serviço de Controle de Infecção Hospitalar
SeAD-UFSCar – Secretaria Geral de Educação a Distância da Universidade Federal de São Carlos
SP – São Paulo
TAS – Teoria da Aprendizagem Significativa
TCLE – Termo de Consentimento Livre e Esclarecido
TIC – Tecnologia da Informação e Comunicação
QBMI – Questionário para Identificação das Boas Práticas com Medicações Injetáveis
UFSCar – Universidade Federal de São Carlos
UTI – Unidade de Terapia Intensiva

SUMÁRIO

APRESENTAÇÃO 19
1 INTRODUÇÃO 20
2 FUNDAMENTAÇÃO TEÓRICA 20
2.1 BOAS PRÁTICAS COM MEDICAÇÕES INJETÁVEIS: CONTEXTUALIZAÇÃO TEÓRICO-PRÁTICA 20
2.2 O USO DE TECNOLOGIA DA INFORMAÇÃO PARA O ENSINO EM SAÚDE 24
2.3 A TEORIA DA APRENDIZAGEM SIGNIFICATIVA 27
3 OBJETIVOS 28
3.1 OBJETIVO GERAL 29
3.2 OBJETIVOS ESPECÍFICOS 29
4 MATERIAIS E MÉTODOS 29
4.1 ANÁLISE CONTEXTUAL 30
4.1.1 Prática com Medicações Injetáveis: Elaboração e Validação do Instrumento para Coleta de Dados 31
4.1.1.2 Construção do Questionário para Identificação das Boas Práticas com Medicações Injetáveis (QBMI) 31
4.1.1.3 Validação por Conteúdo do Questionário para Identificação das Boas Práticas com Medicações Injetáveis 31
4.1.1.3.1 Critérios para validação de conteúdo do Questionário para Identificação das Boas Práticas com Medicações Injetáveis 32
4.1.1.3.2 Seleção de Especialistas para Validação do Questionário para Identificação das Boas Práticas com Medicações Injetáveis 32
4.1.1.3.3 Análise dos dados 33
4.1.2 Prática Referida de Profissionais de Enfermagem sobre Medicações Injetáveis 33
4.1.2.1 Tipo de Estudo 34
4.1.2.2 Participantes da Pesquisa 34
4.1.2.2.1 Critérios de Seleção 34
4.1.2.3 Coleta de Dados 34
4.1.2.5 Análise dos Dados 35
4.2 DESENVOLVIMENTO 36
4.2.1 Curso "Boas Práticas com Injetáveis: Ações para o Controle de Infecção": Planejamento, Elaboração, Validação e Revisão das Ferramentas Educativas 37
4.2.1.1 Elaboração das Ferramentas Educativas 37
4.2.1.2 Validação por Conteúdo das Ferramentas Educativas 38
4.2.1.2.1 Critérios para Validação por Conteúdo 38
4.2.1.2.2 Seleção de Avaliadores para Validação por Conteúdo 39
4.2.1.2.3 Análise dos Dados 40
4.3 IMPLEMENTAÇÃO 40
4.4 AVALIAÇÃO 41
4.4.1 Construção do Questionário para Coleta de Dados 41
4.4.2 Participantes da Pesquisa 42
4.4.2.1 Critérios de Seleção 42
4.4.3 Análise dos Dados 42
4.5 ASPECTOS ÉTICOS DA PESQUISA 43

5 RESULTADOS ... 43
5.1 ANÁLISE CONTEXTUAL ... 43
5.1.1 Prática com Medicações Injetáveis: Elaboração e Validação do Instrumento para Coleta De Dados ... 43
5.1.1.1 Caracterização dos Juízes ... 44
5.1.1.1 Validação do Questionário de Boas Práticas de Medicações Injetáveis ... 44
5.1.2 Prática referida de profissionais de enfermagem sobre medicações injetáveis ... 60
5.1.2.1 Participantes da pesquisa ... 61
5.1.2.2 Prática referida com Medicações Injetáveis ... 63
5.2 DESENVOLVIMENTO ... 102
5.2.1 Curso "Boas Práticas com Injetáveis: ações para o controle de infecção": planejamento, elaboração, validação e revisão das ferramentas educativas ... 102
5.2.1.1 Elaboração das ferramentas educativas ... 102
5.2.1.1 A construção do ebook "Boas Práticas com Injetáveis: Ações para o Controle de Infecção" 104
5.2.1.2 A construção do podcast "Boas Práticas com Medicações Injetáveis: Subsídios para o Autocuidado" 105
5.2.1.3 Questões avaliativas do conteúdo do curso e questões para interação interparticipantes no fórum 106
5.2.1.2 Validação por conteúdo das ferramentas educativas ... 109
5.2.1.2.1 Caracterização das avaliadoras 109
5.2.1.2.2 Processo de validação do ebook "Boas Práticas com Injetáveis: Ações para o Controle de Infecção 109
5.2.1.2.3 Processo de validação do podcast "Boas Práticas com Medicações Injetáveis: subsídios para o autocuidado" 116
5.2.1.2.4 Processo de validação do questionário para certificação e questões do fórum do curso "Boas Práticas com Medicações Injetáveis: ações para o controle de infecção" 121
5.3 IMPLEMENTAÇÃO ... 126
5.4 AVALIAÇÃO ... 137
5.4.1 Construção do Instrumento de Coleta de Dados ... 138
5.4.2 Caracterização dos Participantes da Pesquisa ... 138
5.4.3 A percepção dos participantes sobre o curso "Boas práticas com Injetáveis: ações para o controle de infecção" ... 139
6 DISCUSSÃO DOS RESULTADOS ... 143
6.1 PRÁTICA COM INJETÁVEIS: UMA ANÁLISE CONTEXTUAL ... 143
6.1.1 Questionário para Identificação das Boas Práticas com Medicações Injetáveis: Sobre seu Processo de Construção e Validação ... 143
6.1.2 Prática Referida de Profissionais de Enfermagem com Medicações Injetáveis ... 144
6.1.2.1 A Higienização das Mãos Durante a Prática com Medicações Injetáveis ... 146
6.1.2.2 Preparo do Ambiente para Administração de Injetáveis ... 147
6.1.2.3 O Preparo das Medicações Injetáveis: uma Prática Asséptica ... 148
6.1.2.4 Administração de Injetáveis: os Riscos Presentes nos Detalhes ... 150
6.1.2.5 Profissional de Enfermagem e o Cuidado com sua Segurança ao Manipular Perfurocortantes ... 156
6.2 CURSO "BOAS PRÁTICAS COM INJETÁVEIS: AÇÕES PARA O CONTROLE DE INFECÇÃO": DO DESENVOLVIMENTO À IMPLEMENTAÇÃO ... 162
6.2.1 O Processo de Validação e Revisão do Material Educativo do Curso "Boas Práticas com Injetáveis: Ações para o Controle de Infecção" ... 164

6.3 AVALIAÇÃO DO CURSO “BOAS PRÁTICAS COM INJETÁVEIS: AÇÕES PARA O CONTROLE DE INFECÇÃO” CONFORME PERCEPÇÃO DA POPULAÇÃO-ALVO 166
6.4 LIMITAÇÕES DO ESTUDO 171
6.5 CONTRIBUIÇÕES PARA A ENFERMAGEM.. 171
7 CONSIDERAÇÕES FINAIS 172
8 CONCLUSÕES 173
REFERÊNCIAS 174
APÊNDICE A – TERMO DE CONSENTIMENTO LIVRE E ESCLARECIDO ENCAMINHADO AOS JUÍZES PARA VALIDAÇÃO DO QUESTIONÁRIO PARA IDENTIFICAÇÃO DAS BOAS PRÁTICAS COM MEDICAÇÕES INJETÁVEIS 184
APÊNDICE B – TERMO DE CONSENTIMENTO LIVRE E ESCLARECIDO ENCAMINHADO AOS PARTICIPANTES DA PESQUISA QUE RESPONDERAM AO QUESTIONÁRIO PARA IDENTIFICAÇÃO DAS BOAS PRÁTICAS COM MEDICAÇÕES INJETÁVEIS 186
APÊNDICE C - TERMO DE CONSENTIMENTO LIVRE E ESCLARECIDO ENCAMINHADO AOS AVALIADORES PARA VALIDAÇÃO DO MATERIAL EDUCATIVO DO CURSO “BOAS PRÁTICAS COM INJETÁVEIS: AÇÕES PARA O CONTROLE DE INFECÇÃO” 188
APÊNDICE D TERMO DE CONSENTIMENTO LIVRE E ESCLARECIDO ENCAMINHADO A POPULAÇÃO-ALVO PARA AVALIAR A PERCEPÇÃO DO CURSO “BOAS PRÁTICAS COM INJETÁVEIS: AÇÕES PARA O CONTROLE DE INFECÇÃO” 189
APÊNDICE E – VERSÃO DO QUESTIONÁRIO PARA IDENTIFICAÇÃO DAS BOAS PRÁTICAS COM MEDICAÇÕES INJETÁVEIS ENCAMINHADO AO COMITÊ DE JUÍZES PARA VALIDAÇÃO 191
APÊNDICE F – RECOMENDAÇÕES PRESENTES NA LITERATURA PARA AS BOAS PRÁTICAS COM MEDICAÇÕES INJETÁVEIS, CONFORME CONTEÚDO DOS ITENS DO QUESTIONÁRIO PARA IDENTIFICAÇÃO DAS BOAS PRÁTICAS COM MEDICAÇÕES INJETÁVEIS 199
APÊNDICE G – QUESTIONÁRIO PARA IDENTIFICAÇÃO DAS BOAS PRÁTICAS COM MEDICAÇÕES INJETÁVEIS: VERSÕES PARA APLICAÇÃO EM CONTEXTOS DE SAÚDE.. 207
APÊNDICE H – QUESTIONÁRIO PARA IDENTIFICAÇÃO DAS BOAS PRÁTICAS COM MEDICAÇÕES INJETÁVEIS DISPONIBILIZADO NA PLATAFORMA SURVEYMONKEY AOS PARTICIPANTES DA PESQUISA 219
APÊNDICE J – QUESTIONÁRIO PARA VALIDAÇÃO DO MATERIAL EDUCATIVO DO CURSO “BOAS PRÁTICAS COM INJETÁVEIS: AÇÕES PARA O CONTROLE DE INFECÇÃO” ENCAMINHADO ÀS JUÍZAS 238
APÊNDICE K – EBOOK “BOAS PRÁTICAS COM INJETÁVEIS: AÇÕES PARA O CONTROLE DE INFECÇÃO” VALIDADO 276
APÊNDICE L – TRANSCRIÇÃO DO ÁUDIO DO PODCAST “BOAS PRÁTICAS COM MEDICAÇÕES INJETÁVEIS: SUBSÍDIOS PARA O AUTOCUIDADO”...................................... 288
APÊNDICE M – QUESTÕES AVALIATIVAS DO CURSO “BOAS PRÁTICAS COM INJETÁVEIS: AÇÕES PARA O CONTROLE DE INFECÇÃO” 292
APÊNDICE N – QUESTIONÁRIO PARA IDENTIFICAÇÃO DA PERCEPÇÃO DA POPULAÇÃO-ALVO SOBRE O CURSO “BOAS PRÁTICAS COM INJETÁVEIS: AÇÕES PARA O CONTROLE DE INFECÇÃO” 297
ANEXO A – DECLARAÇÃO EMITIDA PELO CONSELHO REGIONAL DE ENFERMAGEM REFERENTE A AUTORIZAÇÃO PARA DIVULGAÇÃO DO QUESTIONÁRIO PARA IDENTIFICAÇÃO DAS BOAS PRÁTICAS COM MEDICAÇÕES INJETÁVEIS VIA ELETRÔNICA AOS INSCRITOS DE SUA JURISDIÇÃO 315
ANEXO B – APROVAÇÃO DO PROJETO PELO COMITÊ DE ÉTICA E PESQUISA COM SERES HUMANOS DA UNIVERSIDADE FEDERAL DE SÃO CARLOS 316

APRESENTAÇÃO

Iniciei a graduação em enfermagem na Universidade Federal de São Carlos em 2008, mas, foi em 2010 que iniciei meu primeiro projeto de pesquisa sob orientação da professora Rosely Moralez e em conjunto com minha amiga Juliana Martinez.

Cito este momento porque a experiências desta convivência, bem como o desenvolvimento deste projeto foram primordiais para que, ao finalizar a graduação, iniciasse o mestrado, em 2012, como bolsista pela Coordenação de Aperfeiçoamento de Pessoal de Nível Superior e sob orientação da professora Rosely.

Após o mestrado, participei de um programa de residência multiprofissional e desenvolvi atividades laborais em serviços hospitalares, mas ainda tinha o desejo de retornar à pós-graduação, para realizar o doutorado.

O que me fez querer continuar desenvolvendo projetos de pesquisa foi observar o quanto isto possibilita o crescimento pessoal, também, e não apenas o olhar mais crítico para cada ação que realizamos ao longo de nossas atividades laborais.

Enquanto atuava como enfermeira, em 2016, tive a oportunidade de prestar concurso para professora no Centro Paula Souza, onde atuo até hoje.

Foi quando conversei novamente com a professora Rosely, passei pelo processo seletivo e demos andamento a este projeto sobre a construção de um material educativo sobre Boas Práticas com Medicações Injetáveis.

Alerta ao tema, toda prática que experienciava em serviços de saúde junto aos alunos do curso técnico em enfermagem ganhava novos significados, ouvir relatos de outros profissionais, ressignificar minhas próprias vivências com acidentes ocupacionais e buscar identificar o que poderia apresentar um desafio para as boas práticas me estimulavam a buscar estratégias para a construção do material educativo que abordamos neste trabalho.

Ao longo do desenvolvimento deste projeto, tive um aprendizado imensurável, seja a partir da expertise das docentes, das discussões em grupo de pesquisa ou discutindo sobre o tema com colegas, profissionais e alunos, e me sinto grata por poder retribuir todas estas contribuições com este estudo.

1 INTRODUÇÃO

Dentre as medidas que ensejam esforços para a prevenção de Infecção Relacionada a Assistência à Saúde (IRAS), cita-se a injeção segura (STEINKULLER *et al.*, 2018).

As práticas seguras envolvendo injetáveis incluem medidas que otimizam a segurança do paciente e do profissional de saúde, prevenido a geração de resíduos perigosos para a comunidade, danos e a transmissão de doenças (WHO, 2011; PERZ *et al., 201*0).

Contudo, a literatura aponta para os desafios encontrados na implantação destas medidas em serviços de saúde para a injeção segura (KOSSOVER-SMITH *et al*., 2017; MAGEROTE *et al*., 2011; NEGELISKII; LAUTERT, 2017; RAMOS, 2012; SCHUTIJSER et al., 2018) bem como os potenciais desfechos negativos que podem advir destas, como a transmissão de hepatites B e C e HIV (ARNOLD *et al.,* 2017; BÜCHNER *et al*., 2015; VAN TUONG *et al*., 2017).

Neste sentido, a literatura tem apontado para a efetividade das estratégias educativas pautadas pela Tecnologia da Informação e Comunicação (TIC) a fim de aprimorar conteúdos pertinentes para a prática da enfermagem (MÄNNISTÖ *et al*., 2019).

Para tanto, o presente trabalho buscou identificar como profissionais de enfermagem referem a prática com injetáveis e, a partir disto, elaborar e validar material educativo sobre o tema, a fim de subsidiar a atuação destes em serviços de saúde.

2 FUNDAMENTAÇÃO TEÓRICA

2.1 BOAS PRÁTICAS COM MEDICAÇÕES INJETÁVEIS: CONTEXTUALIZAÇÃO TEÓRICO-PRÁTICA

Embora as boas práticas com injetáveis permeiem ações que são primordiais para a segurança do paciente e do profissional e que devem contemplar todas as injeções parenterais, a maioria dos achados na literatura referem-se às administrações de medicamentos via EV e a prevenção de transmissão de microrganismos por esta.

Estas ações, envolvem: implementação de programas de controle de infecção nos serviços de saúde, Higienização das Mãos (HM), preparo asséptico das medicações

injetáveis com uso único de seringas e agulhas, desinfecção de diafragma de frascos e ampolas, utilização individualizada de frascos multidoses sempre que possível, descarte de perfurocortantes em recipientes padronizados e manutenção de estratégias para prevenção de exposição aos patógenos pelos colaboradores, tanto para serviço intra quanto extra-hospitalar (STEINKULLER *et al.*, 2018).

Como danos advindos da inserção de cateteres contaminados, podem ser citados os eventos não infecciosos como abcessos e reações tóxicas (RIGOTTI, 2012) e a Infecção de Corrente Sanguínea (ICS) ainda que mais raramente associada ao Cateter Venoso Periférico (CVP) (SATO *et al.*, 2017).

A ICS pode ser desencadeada pela migração de microrganismos da pele no local de inserção do cateter, culminando em colonização da ponta do cateter e da superfície do mesmo, por contaminação direta do cateter ou seu *hub* através do contato com mãos, cobertura com curativo não estéril, fluidos ou dispositivos contaminados e falhas na antissepsia de pele (BRASIL, 2017; O'GRADY *et al.*, 2011) .

Ressalta-se que a cobertura do dispositivo deve permitir a inspeção visual do sítio de punção venosa quanto aos sinais flogísticos o que deve ocorrer com frequência adequada a criticidade do paciente, entre uma hora e uma vez ao dia (BRASIL, 2017).

Frente à dor e parestesia no sítio de punção, deve-se retirar o cateter, bem como quando este dispositivo não se fizer mais necessário, se instalado em emergência com comprometimento da técnica asséptica, se for inutilizado nas últimas 24 horas, ou em 96 horas após sua instalação, podendo-se estender este prazo após avaliação clínica (BRASIL, 2017).

A inobservância destas estratégias pode causar flebite. Ressalta-se que o surgimento de flebite é um indicador da qualidade da assistência de enfermagem prestada, no âmbito hospitalar (MAGEROTE *et al.*, 2011).

Embora eficazes para a prevenção de danos, a literatura, tanto nacional quanto internacionalmente, tem demonstrado lacunas em relação à implantação destas estratégias na rotina de trabalho dos profissionais de saúde que demonstram baixa adesão às mesmas, principalmente à HM (SCHUTIJSER *et al.*, 2018).

A HM é uma importante medida de controle de infecção, detentora de baixo custo e ampla difusão cientificamente comprovada (BELELA-ANACLETO; PETERLINI; PEDREIRA, 2017).

Dentre os estudos nacionais, um realizado em hospital de Porto Alegre (SP) encontrou que apenas 28,3% dos funcionários de enfermagem realizavam HM antes do preparo de medicações injetáveis e 49% antes de administrá-las (NEGELISKII, 2015).

Este mesmo estudo (NEGELISKII, 2015) também identificou que: 64,7% dos profissionais realizavam desinfecção de conectores de acesso endovenoso, 66,5% realizavam antissepsia da pele para administração de medicações subcutâneas (SC) ou intramusculares (IM), 68,9% realizavam desinfecção de ampola ou frasco-ampola e 26,9% contaminaram o material durante a abertura de seu invólucro no preparo ou reutilizaram material descartável.

O mesmo trabalho supracitado investigou o uso de luvas, em que 59,1% utilizaram este Equipamento de Proteção Individual (EPI) para administração de medicações por vias Endovenosa (EV), SC e IM (NEGELISKII, 2015).

Após estratégia educativa (grupo focal), fora observado no estudo de Negeliskii (2015) que, embora não tenha havido melhora estatisticamente significativa para o processo de preparo e administração de medicações injetáveis, houve aumento de 71,7% dos profissionais que utilizaram luvas para administração de medicações via EV, SC ou IM e, melhora na desinfecção de ampolas e frasco-ampolas, bem como limpeza de conectores, contudo, a adesão à HM foi diminuída.

A redução da adesão da HM seguida do aumento do número de adesão às luvas chama a atenção, uma vez que o uso deste EPI não substitui a HM (BELELA-ANACLETO; PETERLINI; PEDREIRA, 2017).

Corroborando com os achados dos estudos supracitados, estudo desenvolvido no Rio de Janeiro constatou que de 324 observações diretas, em 88,77% não houve troca de agulha entre o preparo e administração do medicamento, em 80,70% não houve desinfecção das ampolas e 77,26% das bancadas de preparo de medicações, demonstrando o grande potencial de contaminação microbiológica destas (CAMERINI; SILVA, 2011).

As superfícies em que as medicações injetáveis são preparadas, também, requerem atenção para a manutenção da esterilidade da solução a ser administrada.

Para tanto, o álcool a 70% para desinfecção de superfícies é considerado um eficiente desinfetante, pois, é eficaz mesmo em presença de matéria orgânica (GRAZIANO *et al.*, 2012).

A solução supracitada, também, apresentou sua eficiência para a desinfecção de ampolas de vidro, contudo, pode não ser suficiente para a remoção de microrganismos de ampolas de plástico, sendo esta falha associada a contaminação de seus conteúdos e requerendo novos estudos para verificação da eficácia desta prática frente a este material (RIGOTTI, 2012).

Internacionalmente, encontra-se um cenário semelhante, no tocante aos desafios para o controle de infecção associado às práticas com injetáveis.

Assim como o estudo de Negeliskii (2015), estudo português identificou baixa adesão de enfermeiros a HM antes do preparo de medicações EV (32,4%) e após a administração destas (64,9%), para a desinfecção de oclusores e frascos (variando de 2,8 a 19,8%) de manuseio asséptico de dânulas (2,5%) e ocorrência de compartilhamento de frascos para múltiplas diluições (25%) (RAMOS, 2012).

Estudo iraniano realizado com frascos multidoses em hospital de ensino identificou que de 637 frascos avaliados, 5,6% estavam contaminados com bactérias, das quais 88,9% eram gram-positivas, com maior frequência de *Staphylococcus epidermidis*, sendo mais elevada em enfermaria pediátrica e menos em Unidade de Terapia Intensiva (UTI) (MOTAMEDIFAR; ASKARIAN, 2009).

Investigação de surtos de hepatite ocorridos, mesmo em ambiente extra-hospitalar, nos Estados Unidos destacou a contaminação de medicações injetáveis, reutilização de seringas ou soluções salinizantes, independentemente da via de administração (EV, IM ou intradérmica) demonstrando o quanto se faz emergente identificar fatores que influenciem na adesão às boas práticas com injetáveis (CARLSON; PERL, 2010; PERZ *et al.*, 2013; RIGOTTI, 2012).

Estudo italiano multicêntrico realizado com enfermeiros para captar o conhecimento destes acerca da prevenção de Infecção Relacionada a Acesso Vascular Periférico (IAVP) identificou que a maioria dos participantes da pesquisa (55% com mais de 10 anos de experiência laboral) identificava as recomendações sobre troca rotineira do CVP (90%), manipulação asséptica de conexão e desconexão das linhas venosas (55,2%) (CICOLINI *et al*., 2014).

Partindo dos achados supracitados, Cicolini et al (2014) reconhecem sobre a necessidade de treinamentos sobre o tema, não apenas focados no conteúdo em si, mas

também da influência da adesão às boas práticas na segurança do paciente para prevenir IAVP.

Ao buscar cursos voltados para a prática com injetáveis, observa-se que o controle de infecção pode estar inserido dentro do conteúdo, porém, não de forma tão enfatizada.

Desta forma, tem-se uma demanda pela difusão de conhecimento sobre o tema, visando reduzir lacunas como as aqui relatadas, a fim de garantir a segurança do paciente e do profissional ou estudante durante procedimentos envolvendo medicações injetáveis.

2.2 O USO DE TECNOLOGIA DA INFORMAÇÃO PARA O ENSINO EM SAÚDE

A utilização da Tecnologia da Informação e Comunicação (TIC) na educação permanente tem sido associada ao protagonismo dos profissionais, bem como favorecimento do acesso aos cursos, democratizando o conhecimento e possibilitando a transformação da prática (FARIAS *et al.*, 2017).

A Educação a Distância (EAD), regida pelo Decreto nº 9.057/2017 deve ser compreendida para além da distância física, devendo-se considerar, também, a distância temporal envolvida, visto possibilidade de flexibilização de acesso ao conteúdo (o qual utiliza TIC), o que pode ser adaptado a rotina do participante/aluno de vários níveis de educação (FERNANDES; HENN; KIST, 2020).

Em nível brasileiro, Fernandes, Henn e Kist (2020) abordam sobre o início da EAD, datando do século XX, e a utilização de correspondências para este fim, passado pelo apoio de mídias como televisão e rádio, e atualmente contando com a *internet*, o que facilitou o acesso aos cursos, atenuando a distância entre alunos e professores, sendo a Universidade Aberta do Brasil, criada em 2006, um importante marco nacional.

A fim de atenuar os impactos causados pela distância física, cursos na modalidade EAD buscam oportunizar a interação entre sujeitos (FERNANDES; HENN; KIST, 2020).

O Ambiente Virtual de Aprendizagem (AVA), enquanto ferramenta da EAD, potencializa a organização do aluno para a construção do conhecimento, seja esta individual ou coletiva, possibilitando a interação entre os participantes, para troca de informações, dúvidas, dentre outros (FERNANDES; HENN; KIST, 2020).

A EAD deve estimular tanto a autonomia quanto a disciplina dos alunos, devendo a metodologia na qual o AVA é pautado estar em consonância com isto (FERNANDES; HENN; KIST, 2020).

Dentre materiais educativos passíveis de elaboração pautados pela TIC, podem ser citados vídeos (ABEDIN *et al*., 2015; COGO; PERRY; SANTOS, 2015; FROTA, NATASHA MARQUES *et al*., 2013; ITAKUSSU *et al*., 2014; LIMA *et al*., 2017; PINTO *et al*., 2018; RODRIGUES JUNIOR *et al*., 2017; ROSA, 2015; SILVA *et al*., 2017; SOUSA et al., 2015), infográficos, (CARVALHO; ARAGÃO, 2012; ESCOBAR, 2018), *podcasts* (ABATE, 2013; SWIGART; LIANG, 2016) e *ebook* (FILATRO; CAIRO, 2015).

No campo da educação em saúde, estas ferramentas têm-se disseminado amplamente, evidenciando a aprendizagem significativa a partir da flexibilização de acessos por parte do participante (THOMAS; FONTANA, 2020).

Segundo revisão integrativa, o treinamento de enfermeiros mediado por tecnologia educacional mostra-se ferramenta de relevância para o desenvolvimento de conhecimento, atitudes e habilidades, com predomínio de publicações estadunidense, e de ferramentas como simulações e vídeos (KOBAYASHI; ARAÚJO, 2019).

Um exemplo de AVA é o Portal de Cursos Abertos (PoCA) da Universidade Federal de São Carlos (UFSCar).

O PoCA (derivada de “póka+ar”, que em tupi significa “bater com força, estourar”) e do sabiá-poca (pássaro frequentemente presente na UFCar), é uma plataforma disponibilizada pelo AVA do Modular Object-Oriented Dynamic Learning Environment (Moodle) e que contempla cursos à distância, abertos e gratuitos, sendo administrado por equipe de pedagogia, tecnologia da informação, audiovisual e comissão de avaliação de cursos da Secretaria Geral de Educação a Distância da UFSCar (SeAD-UFSCar) (UNIVERSIDADE FEDERAL DE SÃO CARLOS, c2020)

A plataforma supracitada oferece à população geral a oportunidade tanto formativa quanto de atualização com possibilidade de certificação à conclusão dos cursos após cumprimento de requisitos especificados a cada curso (UNIVERSIDADE FEDERAL DE SÃO CARLOS, c2020).

Relato trazido por Swigart e Liang (2016) apresenta um pouco do aspecto histórico dos cursos abertos de abrangência massiva, com início em 2002 através do Instituto de Tecnologia de Massachusetts (MIT), e adesão a partir de 2006 em países como

Estados Unidos, Japão, China, França e Reino Unido, em cursos oferecidos por universidades, sem interesse financeiro.

Foi em 2008 que a organização sem fins lucrativos, Khan Academy, passou a oferecer material didático de apoio às aulas de enfermagem e medicina, disponibilizando em formato de vídeo e sessões práticas para aproveitamento por alunos ou docentes das áreas, contemplando temas como doenças infecciosas, anatomia, fisiologia e antibioticoterapia (SWIGART; LIANG, 2016).

Estruturalmente, estas plataformas oferecem descrição do curso, plano de estudo, linha do tempo e tarefas a serem realizadas, podendo durar entre quatro e 10 semanas seguindo uma flexibilidade de acesso por parte do aluno que pode acessar sozinho ou em lugar coletivo, com opção de tutoria para discussão de temas pertinentes ao curso bem como fórum de discussão(MÄNNISTÖ *et al.*, 2019).

Sobre a aplicação da TIC ao ensino de enfermagem, este tem sido considerado um método efetivo para o desenvolvimento do pensamento crítico e proatividade do aluno no seu processo de ensino (COSTA et al., 2021).

A capacitação profissional on-line sem tutoria utilizando AVA (Moodle) a partir dos princípios da Andragogia, voltada para profissionais de 27 UTIs neonatal e pediátrica é um exemplo da utilização desta ferramenta (BUSSOTTI *et al*., 2016).

No tocante ao preparo e administração de medicação, observa-se que esforços têm sido dispendidos a fim de apresentar material educativo digital com potencial de uso em ambientes de ensino, como a construção e validação do jogo virtual “Medsafe” para a promoção da segurança do paciente (FRUTUOSO *et al*., 2019).

Ainda sobre temas permeando as práticas com injetáveis, cita-se hipermídia sobre punção venosa periférica, a qual mostrou-se efetiva para o ensino na graduação, enquanto apoio a professores e estudantes (FROTA *et al*., 2015).

Revisão sistemática com ensaios clínicos randomizados entre 2003 e 2018, demonstrou que temas como injeção intramuscular e HM utilizam ferramentas digitais, facilitando a interação, resolução de problemas e construção do aprendizado de estudantes de enfermagem, considerando-se países como Inglaterra, Espanha, Tailândia, Canadá e Taiwan (MÄNNISTÖ *et al.*, 2019).

Na Colômbia, um curso autoinstrucional a partir de aplicativo para celular mostrou-se efetivo enquanto ferramenta de apoio para ensino sobre administração

medicamentosa e identificou o impacto positivo da ferramenta junto a estudantes de enfermagem, bem como promoveu a inclusão tecnológica atrelada ao processo de ensino e aprendizagem com fortalecimento da autonomia do futuro profissional (RINCÓN; RIVEROS, 2020).

Não foi identificado cursos abertos para utilização por profissionais e estudantes de enfermagem do Brasil que acolhessem ao tema sobre Boas Práticas com Medicações Injetáveis com ênfase no controle de IRAS, abordando aspectos como preparo e manipulação da injeção segura e cuidados à saúde do profissional/estudante que realiza esta prática.

2.3 A TEORIA DA APRENDIZAGEM SIGNIFICATIVA

A escolha da teoria para subsidiar o presente trabalho considerou o cognitivismo, em que o processo educativo se dá a partir da aquisição de novas informações, as quais o indivíduo relacionará àquelas já presentes em sua estrutura mental de representações para só então armazená-las na memória, recuperando-as mediante necessidade do contexto (MOREIRA; MASINI, 2001).

Dentre as teorias que contemplam este construto cognitivista, encontra-se a Teoria da Aprendizagem Significativa (TAS) proposta por David Ausubel, em 1963 (SOUSA *et al.*, 2015).

Sobre a TAS, tem-se que o conhecimento prévio é primordial para a aprendizagem significativa, a qual deve contar, também com a predisposição do aluno em aprender e a apresentação de material potencialmente significativo, ou seja, o conteúdo deve estar relacionado com o conhecimento prévio do sujeito (AGRA *et al*., 2019; SILVA, 2020).

Aquilo que o indivíduo já conhece/sabe sobre um determinado tema, é definido como conceito subsunçor (MOREIRA; MASINI, 2001).

Para Ausubel, a aprendizagem significativa irá ocorrer quando novas informações forem ancoradas nesses conceitos subsunçores, e é este o ponto diferencial à aprendizagem mecânica, pois, esta interação gera um *continuum* do processo de aprendizagem (MOREIRA; MASINI, 2001).

A ação do indivíduo decorrente de decisões impessoais o torna passivo, visto que o significado atribuído à mesma é um reflexo do significado de outrem, reduzindo, desta forma, sua responsabilidade e participação ativa, levando-o a uma ação mecanizada (MOREIRA; MASINI, 2001).

O que se deseja, então, é o papel ativo do indivíduo pelo aprendizado, ou seja, tem-se que é a partir da consciência que o sujeito tem de sua ação que ele passa a desenvolver sua compreensão da realidade de forma contextualizada, atribuindo significado a ela (SOUSA *et al*., 2015).

Revisão integrativa realizada em 2015 com o intuito de sintetizar a produção científica em enfermagem pautada na TAS (SOUSA *et al.*, 2015) apresentou cinco artigos, dos quais, quatro faziam alusão a teoria, quatro citaram Ausubel e apenas dois seguiram completamente a estrutura para a aplicação desta teoria.

A TAS pode ser aplicada à enfermagem para a educação profissional, apresentando conceitos que possam reelaborar o conhecimento prévio do sujeito fazendo com que ele retenha aquilo que lhe faz sentido e seja significativo para a transformação da prática laboral a partir da problematização do processo de trabalho (STRUCHINER; GIANNELLA, 2006).

Aqui se enfatiza a importância desta teoria para o embasamento do material educativo a ser elaborado no presente trabalho: é preciso identificar o quanto o tema é familiar a população-alvo deste estudo, pois, é provável que estes já tenham um conhecimento prévio sobre o tema, sendo primordial a consideração deste cenário.

Para tanto, a fim de desenvolver material educativo sobre Boas Práticas com Medicações Injetáveis, visto necessidade de estratégias que aperfeiçoem a adesão à estas, almejou-se apresentar um diagnóstico situacional referente ao tema no estado de São Paulo (SP) e a partir deste construir uma ferramenta educativa pautada pela TAS e pela TIC, de modo a oferecer para os profissionais e estudantes da área da enfermagem, material atualizado e que auxilie na prevenção de transmissão de patógenos por meio das ações que permeiam esta prática.

3 OBJETIVOS

3.1 OBJETIVO GERAL

- Construir e validar material educativo sobre as boas práticas na administração de medicações injetáveis a partir da prática referida de profissionais de enfermagem.

3.2 OBJETIVOS ESPECÍFICOS

- Construir e validar um instrumento de mensuração de prática referida sobre práticas na administração de medicamentos injetáveis;
- Realizar um diagnóstico situacional sobre prática referida de administração de medicações injetáveis por profissionais de enfermagem do estado de São Paulo;
- Construir e validar, por conteúdo, material educativo sobre Boas Práticas em Medicações Injetáveis;
- Identificar a percepção de estudantes e profissionais de enfermagem sobre o material educativo proposto.

4 MATERIAIS E MÉTODOS

A fim de atender aos objetivos propostos, foram consideradas as seguintes etapas:

Figura 1 - Etapas da elaboração do material educativo sobre Boas Práticas em Medicações Injetáveis conforme sugerido por Filatro e Cairo (2015).

Análise contextual

- Objetivos educacionais
- Caracterização do público-alvo
- Levantamento de potencialidades e restrições

Desenvolvimento

- Planejamento das ferramentas para apresentação de conteúdo
- Elaboração de conteúdo inédito e propriedade intelectual
- Composição das ilustrações
- Gravação de locuções
- Elaboração e validação da primeira versão do conteúdo educativo
- Revisão do material educativo

Implementação

- Distribuição virtual

Avaliação

- Avaliação pela população-alvo

Fonte: Elaborado pela autora

4.1 ANÁLISE CONTEXTUAL

Para a análise contextual foi construído um questionário para coleta de dados sobre as práticas com medicações injetáveis e, a partir das respostas, identificada a prática referida de profissionais de enfermagem.

Desta forma, a partir da prática referida, foi possível caracterizar a população-alvo deste estudo quanto a idade, tempo de trabalho, tipo de serviço de saúde e identificar o quanto a prática se aproxima do que é preconizado pela literatura científica.

Este dado, enquanto conhecimento prévio, foi primordial para o desenvolvimento das ferramentas educativas que contemplaram o curso “Boas Práticas com Injetáveis: ações para o controle de infecção”, desenvolvido neste projeto.

4.1.1 Prática com Medicações Injetáveis: Elaboração e Validação do Instrumento para Coleta de Dados

4.1.1.2 Construção do Questionário para Identificação das Boas Práticas com Medicações Injetáveis (QBMI)

Visando a otimização do tempo e para atingir simultaneamente vários participantes, obtendo-se respostas rápidas e precisas (MARCONI, MA; LAKATOS, 2010), optou-se pela elaboração e validação de um questionário para a coleta de dados.

Denominado Questionário para Identificação das Boas Práticas com Medicações Injetáveis (QBMI), procedeu-se com a pesquisa de desenvolvimento metodológico, a qual visa o desenvolvimento, avaliação e aperfeiçoamento de instrumentos e estratégias metodológicas (POLIT; BECK, 2011).

Para tanto, foram consideradas referencias na literatura abordando tanto diretrizes quanto trabalhos que discorressem sobre a prática com injetáveis, nacionalmente.

4.1.1.3 Validação por Conteúdo do Questionário para Identificação das Boas Práticas com Medicações Injetáveis

A validação de conteúdo por especialistas garante características, como fidedignidade, validade e operatividade (MARCONI; LAKATOS, 2010).

Para tanto, foram utilizados critérios sugeridos por Coluci, Alexandre e Milani (2015) para validação por conteúdo de instrumentos de medida: avaliação dos domínios quanto à abrangência, e dos itens quanto à clareza e pertinência.

Por definição, consideraram-se (COLUCI; ALEXANDRE; MILANI, 2015):

- Clareza: avaliar se os itens são redigidos de modo que o conceito esteja compreensível e se expresse adequadamente ao que se espera medir.

- Pertinência: avaliar se os itens refletem os conceitos envolvidos, se são relevantes e adequados para atingir objetivos almejados.

- Abrangência: avaliar se o domínio estava contemplado adequadamente pelo conjunto de itens, bem como se todas as dimensões estavam inclusas.

Quanto ao QBMI, avaliado por via eletrônica, este era subdividido da seguinte forma: “preparo do ambiente” (cinco itens), “preparo das medicações” (sete itens), “administração medicamentosa” (23 itens) e “cuidados após administração medicamentosa” (sete itens).

Para a apreciação do instrumento de coleta de dados, considerou-se o período entre o convite aos juízes e a devolução do questionário de julho de 2017 a agosto do mesmo ano.

4.1.1.3.1 Critérios para validação de conteúdo do Questionário para Identificação das Boas Práticas com Medicações Injetáveis

Para que cada item permanecesse, seria necessária uma taxa de concordância do comitê de juízes maior que 90% por domínio, e pelo Índice de Validade de Conteúdo (IVC) uma taxa de concordância superior a 0,78 para permanência dos itens, individualmente (COLUCI; ALEXANDRE; MILANI, 2015).

4.1.1.3.2 Seleção de Especialistas para Validação do Questionário para Identificação das Boas Práticas com Medicações Injetáveis

Foram convidados, por conveniência, via eletrônica, para validação do QBMI, participantes que poderiam contemplar o comitê de juízes para validação por conteúdo do QBMI.

Para tanto, foram critérios de inclusão: ter experiência clínica na área de controle de infecção relacionada à assistência à saúde ou TIC aplicada à enfermagem, possuir publicação ou pesquisar sobre o tema, ter conhecimento sobre a construção de questionários ou atuar profissionalmente como enfermeiro e atuar em outros estados, que não São Paulo, visto que a coleta de dados utilizando o QBMI ocorreria neste.

Todos os participantes assinaram o Termo de Consentimento Livre e Esclarecido (TCLE) presente no Apêndice A.

4.1.1.3.3 Análise dos dados

Os dados originados das respostas dos juízes foram tabulados, codificados, armazenados em planilhas do Microsoft Excel e foram calculados a taxa de concordância quanto a abrangência e o IVC conforme as fórmulas a seguir (COLUCI; ALEXANDRE; MILANI, 2015):

$$\text{Taxa de concordância} = \frac{\text{número de participantes que concordaram}}{\text{número total de participantes}} \times 100$$

$$\text{IVC} = \frac{\text{número de respostas "3" ou "4"}}{\text{número total de respostas}}$$

Ressalta-se que os juízes avaliaram cada item do questionário em escala tipo *likert* de quatro pontos ordinais (conforme quadro 1), e a partir disto, foram calculadas as fórmulas acima.

Quadro 2 - Legenda de valores para cálculo do Índice de Validade de Conteúdo do instrumento para coleta de dados. São Carlos, SP, Brasil, 2017.

Valor	Definição
1	Não claro/ não pertinente
2	Item necessita de grande revisão para ser claro/pertinente
3	Item necessita de pequena revisão para ser claro/pertinente
4	Item claro/ pertinente

Fonte: COLUCI; ALEXANDRE; MILANI, 2015

As sugestões apresentadas pelos juízes foram discutidas pelos membros do grupo de pesquisa “Boas práticas para o controle da transmissão de patógenos relacionada à assistência à saúde" da UFSCar, consolidando o QBMI, na versão utilizada para coleta de dados desta pesquisa.

4.1.2 Prática Referida de Profissionais de Enfermagem sobre Medicações Injetáveis

4.1.2.1 Tipo de Estudo

Para esta etapa, utilizou-se o estudo tipo *survey*, em que se identificou a frequência referida dos profissionais de enfermagem sobre as ações relacionadas às práticas de administração de medicações injetáveis através da plataforma de dados SurveyMonkey® (https://pt.surveymonkey.com/r/boas_praticas_med_inj).

4.1.2.2 Participantes da Pesquisa

Os profissionais de enfermagem do estado de São Paulo (Brasil) foram convidados a participar desta etapa do estudo por meio de correio eletrônico, o qual continha o tema, objetivos, aspectos éticos da pesquisa e endereço para acesso eletrônico ao questionário, bem como o TCLE (APÊNDICE B).

Em 2017, havia 484.375 profissionais, com última atualização em 31 de janeiro, lembrando-se que um profissional pode ter seu registro ativo em mais de uma categoria (enfermeiro, auxiliar/técnico de enfermagem, obstetriz e atendente), segundo Conselho Regional de Enfermagem – SP (Coren-SP) (2017).

4.1.2.2.1 Critérios de Seleção

Para esta etapa, os participantes deveriam atender aos critérios de inclusão: ter registro ativo no Conselho Regional do Estado de São Paulo, possuir correio eletrônico cadastrado no referido órgão, desenvolver/ter desenvolvido atividade laboral relacionada à enfermagem (identificado a partir da pergunta sobre o tempo de exercício profissional); ter afinidade com informática e *internet* (identificado através da devolutiva do questionário).

Foram critérios de exclusão: não ter atuado na área da enfermagem, não responder integralmente ao QBMI, não ter afinidade com informática e internet.

4.1.2.3 Coleta de Dados

Para coleta de dados foi utilizado o QBMI validado, em formato eletrônico. A fim de viabilizar que os profissionais do estado de São Paulo respondessem a ele, contou-se

com o apoio do Coren-SP, conforme declaração em Anexo A, em dois momentos, sendo o primeiro em setembro de 2017 e o segundo em novembro do mesmo ano.

Para tanto, o QBMI deveria ser respondido de acordo com a frequência com que o participante considerasse atender às oportunidades em que a atividade ocorria em sua atividade laboral:

- Sempre: quando o sujeito realizasse a prática em 100% das oportunidades;
- Quase sempre: quando o sujeito realizasse a prática em 75% das oportunidades;
- Às vezes: quando o sujeito realizasse a prática em 50% das oportunidades;
- Quase nunca: quando o sujeito realizasse a prática em 25% das oportunidades
- Nunca: se esta atividade não ocorresse em nenhuma oportunidade que o sujeito tivesse.

Tem-se por oportunidade a ocorrência de determinada atividade durante sua prática, por exemplo, supervisores e docentes devem considerar a frequência com que realiza esta atividade quando esta ocorre, mesmo que esta não faça parte de suas atribuições diárias.

Ressalta-se que o QBMI em formato digital na plataforma SurveyMonkey® permaneceu ativo apenas para coleta de dados desta pesquisa (entre agosto de 2017 e março de 2018).

4.1.2.5 Análise dos Dados

Foram analisados apenas questionários preenchidos na íntegra, uma vez que cada item do QBMI requeria, obrigatoriamente, uma resposta para que fosse possível prosseguir: questionários que não contemplassem esse requisito foram desconsiderados no processo de análise (entre outubro/2018 e fevereiro de 2019).

Para descrever o perfil da amostra foram construídas tabelas de frequência das variáveis categóricas, com valores de frequência absoluta (n), frequência relativa (%), e estatística descritiva das variáveis numéricas, com valores de média, desvio-padrão, mínimo, máximo, mediana e quartis.

Para comparar os itens da escala entre as variáveis categóricas foram utilizados os testes qui-quadrado ou exato de Fisher. Para comparação dos itens entre as variáveis

numéricas foram usados os testes de Mann-Whitney (para duas categorias) e de Kruskal-Wallis (para três ou mais categorias), devido à ausência de distribuição normal dos escores. Para analisar a relação entre as variáveis numéricas foi utilizado o coeficiente de correlação de Spearman.

A fim de identificar o conhecimento sobre o assunto, a partir das respostas consolidadas no QBMI, foram somadas as pontuações dos sujeitos de acordo com cada variável, em cada domínio e no total de domínios (ou, todo o questionário) de acordo com a frequência referida (sempre=1, quase sempre=2, às vezes=3, quase nunca=4 e nunca=5).

Para efetuar os cálculos supracitados, consideraram-se os componentes já invertidos (conforme adequação das recomendações vigentes ao item questionado) e o QBMI interpretado no sentido de que quanto maior a pontuação, pior a prática referida, ou seja, o ideal é que haja pontuações baixas para estar em conformidade com as Boas Práticas com Medicações Injetáveis.

A análise estatística supracitada fora realizada por profissional da área, sob referências teóricas pertinentes (CONOVER, 1999; FLEISS; LEVIN; PAIK, 2003; HATCHER, 1994; NUNNALLY, 1978; PEREIRA, 1999; SIEGEL; CASTELLAN JÚNIOR, 2006; STREINER; NORMAN, 1995; TABACHNICK; FIDELL, 2007), com o programa computacional “The SAS System for Windows”[1].

Além disto, foi calculada a consistência interna do QBMI a partir do alfa de Cronbach, sendo considerada valor acima de 0,70 satisfatória (STREINER, 2003).

4.2 DESENVOLVIMENTO

Em consonância com Sousa *et al*. (2015) a fim de atingir os conhecimentos prévios dos sujeitos sobre boas práticas com medicações injetáveis, oportunizando a aprendizagem significativa, foi considerada a prática referida encontrada na devolutiva do QBMI pelos profissionais de enfermagem, conforme a *survey* da análise contextual.

Desta forma, o material foi elaborado a partir de elementos já conhecidos pela população-alvo deste estudo, contudo, enfatizando-se o que poderia ser aprimorado a fim

[1] Statistical Analysis System, versão 9.2. SAS Institute Inc, 2002-2008, Cary, NC, USA

de viabilizar práticas com medicações injetáveis mais seguras para todos os envolvidos, do ponto de vista do controle de infecção.

4.2.1 Curso "Boas Práticas com Injetáveis: Ações para o Controle de Infecção": Planejamento, Elaboração, Validação e Revisão das Ferramentas Educativas

4.2.1.1 Elaboração das Ferramentas Educativas

Após reunião remota em 10 de maio de 2020 com membros da equipe pedagógica da SeAD-UFSCar responsáveis pela administração do PoCA, foram enviadas diretrizes para a pesquisadora a fim de viabilizar a produção do material educativo.

Estas diretrizes contemplavam: encaminhar informações curriculares do proponente do curso, informações do curso (nome, descrição, palavras-chave, justificativa, carga horária, objetivos contendo o que seria esperado do aluno ao término do curso, unidade ou unidades de estudo, bibliografia básica do curso, público, imagem de bancos gratuitos de imagem para construção do *thumbnail* do curso pela equipe do PoCA).

A fim de abordar os temas pertinentes ao curso, elaborou-se um esquema hierárquico (partindo do tema mais geral para o mais específico), para subsidiar o desenvolvimento das ferramentas educativas, conforme sugere a TAS (MOREIRA, 2011a), de acordo com os achados na análise contextual.

Posteriormente, procedeu-se a criação de um texto-base que englobou todo o conteúdo a ser trabalhado na unidade de estudo prevista no planejamento educacional, o qual embasou a produção das ferramentas educativas.

Além disto, a fim de priorizar a interação entre os participantes, foram elaboradas duas questões disparadoras de discussão para o fórum, conforme sugerido pela plataforma que possibilitou a distribuição do curso.

A fim de viabilizar o certificado referente a conclusão do curso, cinco questões objetivas foram construídas, contemplando o mesmo referencial teórico utilizado para a construção das ferramentas educativas, contudo, não os mesmos exemplos abordados nas mídias digitais, conforme sugerido na TAS (MOREIRA, 2011a).

Foram seguidas sugestões de Filatro e Cairo (2015) e dos modelos e diretrizes do PoCA para a elaboração das mídias digitais ebook (texto digital) e podcast (áudio).

Para o ebook, foi disponibilizada a máscara e orientações sobre quantidade de páginas e linguagem a ser utilizada, bem como cores padronizadas, o qual deveria estar em Portable Document Format (PDF) e cujas imagens deveriam ser originadas de licença Royalty-Free (UNVERSIDADE FEDERAL DE SÃO CARLOS, c2020).

Sobre o podcast (quando a duração do áudio estivesse entre três e cinco minutos), deveria ser gravado em MP3 e com no máximo cinco minutos de duração (músicas, também, deveriam ser *Royalty Free*), deveria ainda ser realizada a transcrição do material, garantindo maior acessibilidade (UNVERSIDADE FEDERAL DE SÃO CARLOS, c2020).

Foi, também, disponibilizando tutoriais com dicas elaborados pelo SeAD-UFSCar (https://www.youtube.com/playlist?list=PLS08dhp2EdAbyJpJlHFtZzfLZj3p4cLRF) e INOVAEH (https://inovaeh.SeAD.ufscar.br/material-de-apoio/tutoriais/ferramentas-para-web-2-0/).

Para melhor aproximação com as licenças dos materiais, foi sugerido pela equipe pedagógica, e atendido pela pesquisadora, a inscrição em um curso na mesma plataforma que abordava este tema, antes da escolha do tipo de licença para o material do curso, de forma que este pudesse ser explorado de forma legal, posteriormente à sua disponibilização.

4.2.1.2 Validação por Conteúdo das Ferramentas Educativas

A validação do material educativo do curso “Boas Práticas com Injetáveis: ações para o controle de infecção” ocorreu em setembro de 2020.

4.2.1.2.1 Critérios para Validação por Conteúdo

Para a validação por conteúdo do *ebook* e do *podcast* foi utilizado o Instrumento de Validação de Conteúdo Educativo em Saúde (IVCES), um instrumento generalista que se destina à validação de conteúdo educativo em saúde (LEITE *et al.*, 2018).

São avaliados, a partir do IVCES, os objetivos, a estrutura/apresentação e a relevância do material educativo (LEITE *et al.*, 2018).

Enquanto objetivos, o IVCES avalia se o conteúdo da ferramenta educativa contempla o tema proposto, se adequa-se ao processo de ensino-aprendizagem, esclarece dúvidas, proporciona reflexão e incentiva mudança de comportamento (LEITE *et al.*, 2018).

Por estrutura/apresentação, o IVCES valida o conteúdo referente a linguagem (se apropriada ao público-alvo ao qual o material educativo se destina, se interativa), a informação (se correta, objetiva, esclarecedora e necessária), se a sequência de ideias é lógica, o tema atual e o tamanho do texto adequado (LEITE *et al.*, 2018).

Já a relevância, valida o conteúdo do material enquanto estímulo ao aprendizado, contribuição para o conhecimento e se desperta o interesse pelo tema (LEITE *et al.*, 2018).

O IVCES possui três opções de respostas (0,1 e 2) para avaliação em 18 itens. Sua leitura deve considerar a somatória de todos os domínios, quanto maior o escore, mais eficiente será o conteúdo educativo (LEITE *et al.*, 2018).

Já para a validação das questões avaliativas do conteúdo do curso (para emissão de certificado) e das duas questões do fórum (disparadoras de discussão, sem valor avaliativo), utilizou-se o IVC, conforme proposto por Coluci, Alexandre e Milani (2015).

Para tal, cada questão foi avaliada quanto à clareza (se a redação ocorreu de modo que o conceito estivesse compreensível e se expressava adequadamente ao que se esperava medir) e pertinência (se os itens refletiam os conceitos envolvidos), a partir de uma escala tipo *likert* de 1 a 4 (conforme quadro 2).

4.2.1.2.2 Seleção de Avaliadores para Validação por Conteúdo

Foi emitido convite via e-mail aos 18 possíveis avaliadores, selecionados por conveniência, para validação do material educativo, os quais obedeceram aos seguintes critérios: ser profissional da saúde atuante em serviços de atenção à saúde que realizassem práticas com materiais injetáveis ou em Comissão de Controle de Infecções Relacionadas à Assistência à Saúde, ser profissional ou realizar pesquisas na área de tecnologia da educação, segurança do paciente, risco biológico ou segurança ocupacional.

Mediante aceite, foi enviado novo e-mail, contendo:

- *Ebook* “Boas Práticas com Injetáveis: ações para o controle de infecção”;

- *Podcast* e transcrição “Boas Práticas com Medicações Injetáveis: subsídios para o autocuidado”;

- Cinco questões do tipo múltipla-escolha, avaliativas do conteúdo para emissão do certificado;

- Duas questões abertas disparadoras de discussão para o fórum;

- Link de acesso ao instrumento para validação (https://forms.gle/R1i955YXyt3jzbQJ8) via aplicativo de gerenciamento de pesquisas Google Forms, que continha, também, orientações de preenchimento e o TCLE (APÊNDICE C) para aceite eletrônico.

O material educativo, nesta etapa, já contemplava especificações disponibilizadas pelo PoCA.

4.2.1.2.3 Análise dos Dados

Os dados originados pelas devolutivas das avaliadoras foram armazenados, codificados e analisados em planilhas do Microsoft Excel.

Os dados do IVCES foram avaliados a partir da análise descritiva dos escores (média, mediana, desvio-padrão, mínimo e máximo), enquanto cada item foi avaliado individualmente, a partir das frequências absoluta e relativa a fim de identificar as possíveis melhorias a serem realizadas no *ebook* e no *podcast*.

Já o IVC foi calculado conforme a fórmula a seguir, considerando-se em conformidade quanto igual ou superior a 0,78 (COLUCI; ALEXANDRE; MILANI, 2015):

$$\text{IVC} = \frac{\text{número de respostas "3" ou "4"}}{\text{número total de respostas}}$$

A fim de descrever as sugestões e observações pelas avaliadoras, nomeou-se cada participante desta etapa com a letra “E” seguida por um número, o qual corresponde a ordem de retorno do instrumento devolvido por elas.

4.3 IMPLEMENTAÇÃO

A fim de apresentar o material educativo para estudantes e profissionais de enfermagem (para a implementação do material educativo), identificou-se no PoCA a possibilidade de acolher ao objetivo de atingir a esta população de forma massiva, a partir de um recurso gratuito e que apoia diversas áreas de conhecimento cujos conteúdos podem ser acessados por qualquer pessoa e a partir de qualquer dispositivo (UNVERSIDADE FEDERAL DE SÃO CARLOS, c2020).

Salienta-se que, embora qualquer pessoa possa acessar ao conteúdo da plataforma, de qualquer lugar do mundo, a apresentação da proposta de cursos é exclusiva da comunidade UFSCar ou outras Instituições de Ensino Superior, como docente ou técnico-administrativo (UNVERSIDADE FEDERAL DE SÃO CARLOS, c2020).

A proposta para divulgação e distribuição do material educativo foi apresentada via formulário eletrônico ao PoCA em 21 de março de 2020. O qual continha informações de contato e vínculos com a universidade da doutoranda e sua orientadora e sobre proposta do referido curso.

O material do curso foi inserido na plataforma a partir do apoio da equipe do PoCA, em novembro de 2020.

4.4 AVALIAÇÃO

A fim de identificar a percepção do curso “Boas Práticas Injetáveis: ações para o controle de infecção”, foi construído um questionário disponibilizado eletronicamente no Google Forms, o qual ficou disponível entre novembro de 2020 e janeiro de 2021.

O questionário deveria ser respondido em escala de concordância variando entre concordo totalmente e discordo totalmente.

Ressalta-se que ao acessar o *link* para o questionário, havia a coleta do endereço de e-mail, sendo necessário realizar *login* limitando, assim, uma resposta por pessoa, cuja resposta não poderia ser editada após o envio.

4.4.1 Construção do Questionário para Coleta de Dados

Tal instrumento dividia-se em duas partes: uma contendo questionário sociodemográfico e a outra para a avaliação do curso a partir da gradação de concordância

ao item, de acordo com a percepção do participante, a fim de identificar a aceitabilidade deste enquanto ferramenta educativa.

Foi disponibilizando um campo para que o participante pudesse escrever alguma sugestão ou observação conforme julgasse pertinente.

Os itens avaliados pela população-alvo questionavam tanto sobre a relevância do conteúdo abordado pelo curso quanto sobre a funcionalidade e atratividade das ferramentas escolhidas para a apresentação deste, bem como sobre se o participante se sentia envolvido e estimulado a se aprofundar no tema proposto a partir do seu manuseio.

O questionário construído para esta etapa contemplava o convite para avaliar o curso conforme a percepção e o TCLE (APÊNDICE D) para validar a participação na pesquisa.

4.4.2 Participantes da Pesquisa

4.4.2.1 Critérios de Seleção

Para identificar a percepção do curso “Boas práticas com injetáveis: ações para o controle de infecção” após validação e implementação no PoCA, foram critérios de inclusão: ter mais de 18 anos, ser profissional ou estudante de enfermagem, ter realizado o curso integralmente.

Já critério de exclusão: não ter realizado o curso antes de responder o questionário, o que foi identificado a partir do acesso ao relatório de conclusão das atividades que monitora as atividades dos alunos inscritos (acesso ao *ebook*, ao *podcast* ou transcrição e questionário para conclusão da atividade).

4.4.3 Análise dos Dados

Para identificar a percepção dos participantes da pesquisa, as respostas foram armazenadas, codificadas e analisadas em planilhas do Microsoft Excel a partir da frequência absoluta e relativa de cada item do questionário.

4.5 ASPECTOS ÉTICOS DA PESQUISA

O projeto foi aprovado pelo Comitê de Ética e Pesquisa com Seres Humanos da UFSCar, Parecer Nº 2.139.384 (ANEXO B) e a resolução 466/2012 do Conselho Nacional de Saúde respeitada em sua totalidade.

5 RESULTADOS

5.1 ANÁLISE CONTEXTUAL

5.1.1 Prática com Medicações Injetáveis: Elaboração e Validação do Instrumento para Coleta De Dados

Para a elaboração do QBMI foram utilizados diretrizes e orientações sobre o tema (AGÊNCIA NACIONAL DE VIGILÂNCIA SANITÁRIA; BRASIL, 2017; BRASIL, 2016; ORGANIZAÇÃO MUNDIAL DA SAÚDE, 2010, 2011) e artigos que discorriam sobre como profissionais de enfermagem manipulavam tais insumos (MONCAIO; FIGUEIREDO, 2009; NEGELISKII, 2015; PUGLIESE *et al.*, 2010).

Para tanto, o questionário supracitado subdividia-se em quatro domínios.

O primeiro domínio (preparo do ambiente) indagava sobre a desinfecção de balcões e limpeza de bandeja para o preparo de medicações injetáveis, verificação de esterilidade de insumos utilizados e HM.

O segundo domínio (preparo de medicações) contemplava HM, compartilhamento de frascos de grandes volumes de soluções para reconstituição de doses medicamentosas, compartilhamento de frascos multidoses para dois pacientes ou mais, assepsia de frascos antes da diluição e reuso de insumos descartáveis para o preparo de medicamentos.

O terceiro domínio (administração medicamentosa) abordava a salinização de CVP, reuso de insumos descartáveis para o mesmo paciente e pacientes diferentes, reuso de oclusores e armazenamento destes, HM, desobstrução forçada de CVP, antissepsia para administração de injeções EV, IM e SC, desinfecção de conectores antes da

administração medicamentosa, realização de treinamento para manipulação de agulhas com dispositivos de segurança e uso de luvas para administração de medicação injetável.

No quarto domínio (cuidados após administração medicamentosa) buscou-se conhecer a prática referente ao reencape de agulhas após sua utilização, HM, salinização de CVP, transporte de agulhas desprotegidas após sua utilização, separação entre corpo e agulha da seringa para o descarte, vacinação do profissional contra doenças infecciosas, fluxo de atendimento do profissional após exposição percutânea com material perfurocortante e cuidados a esta lesão.

O QBMI, após construção, ficou estruturado da seguinte forma:

- Preparo do ambiente: cinco itens;
- Preparo das medicações: sete itens;
- Administração medicamentosa: 23 itens;
- Cuidados após administração medicamentosa: sete itens.

Para a validação do QBMI construído pelas autoras e utilizado para coleta de dados (APÊNDICE E), foram convidados 14 especialistas, por e-mail, dos quais seis aceitaram participar desta etapa do estudo, porém apenas cinco responderam integralmente o questionário.

5.1.1.1 Caracterização dos Juízes

Dentre os cinco juízes que participaram da validação do QBMI, 80% (4/5) eram doutores, enquanto 20% (1/5) possuía mestrado como maior grau de instrução; 100% (5/5) atuante na área por 11 a 20 anos, sendo a atividade em ensino a principal, com 100% (5/5).

Ressalta-se que 80% (4/5) era proveniente do estado de Minas Gerais, enquanto 20% (1/5) do Rio de Janeiro.

Com relação ao tipo de serviço em que eles tinham vínculo, 40% (2/5) referiu serviço hospitalar, 80% (4/5) faculdades ou universidades, 20% (1/5) ambulatório ou centros de especialidades e a mesma porcentagem para outros (instituto federal). Considerou-se que o juiz poderia ter mais de um vínculo.

5.1.1.1 Validação do Questionário de Boas Práticas de Medicações Injetáveis

A concordância entre juízes segundo IVC para clareza e pertinência variou entre 0,80 e 1, e pode ser verificada nas tabelas de 1 a 4, conforme domínio de referência do QBMI.

Tabela 1 - Avaliação da concordância da adequação dos itens do domínio "preparo do ambiente" do Questionário para Identificação das Boas Práticas com Medicações Injetáveis. São Carlos, SP, Brasil, 2017.

**Item**	**IVC (Clareza)**	**IVC (Pertinência)**
Com que frequência você desinfeta balcão para preparo das medicações antes do início de suas atividades com álcool a 70% ou outras substâncias padronizadas em seu serviço?	0,80	1
Com que frequência você higieniza as bandejas de preparo e administração de medicações injetáveis a cada preparo destas?	1	1
Com que frequência você verifica a data de validade de materiais utilizados para administração medicamentosa antes de sua utilização?	1*	1*
Com que frequência você utiliza materiais para administração medicamentosa cuja embalagem esteja aberta?	1	1
Com que frequência você higieniza as mãos antes e após o preparo de medicações injetáveis?	1	1

Fonte: Dados da Pesquisa
Nota: *N=4

Tabela 2 - Avaliação da concordância da adequação dos itens do domínio "preparo das medicações" do Questionário para Identificação das Boas Práticas com Medicações Injetáveis. São Carlos, SP, Brasil, 2017.

Item	IVC (Clareza)	IVC (Pertinência)
Com que frequência você higieniza as mãos, com água e sabonete ou solução alcóolica, antes da administração de medicação endovenosas?	1*	1*
Com que frequência você compartilha frascos de soros (de 100 a 1000ml) no preparo de medicações no posto de enfermagem para reconstituição de doses medicamentosas?	0,80	0,80
Com que frequência você compartilha o mesmo frasco com multidoses para dois ou mais pacientes?	0,80	0,80
Com que frequência você utiliza a mesma agulha para aspirar medicações de frascos multidoses?	1	1
Com que frequência você realiza desinfecção com álcool a 70% das tampas de frascos multidoses antes da aspiração do medicamento a ser administrado?	1	1*
Com que frequência você utiliza medicações de frascos que constem data de abertura, volume de diluente (se aplicável), nome do profissional que realizou a abertura e reconstituiu a medicação?	0,80	0,80
Com que frequência você realiza limpeza de frascos e ampolas de medicação antes de aspirá-la?	1	1

Fonte: Dados da Pesquisa
Nota: *N=4

Tabela 3 - Avaliação da concordância da adequação dos itens do domínio "administração medicamentosa" do Questionário para Identificação das Boas Práticas com Medicações Injetáveis. São Carlos, SP, Brasil, 2017.

(continua)

Item	IVC (Clareza)	IVC (Pertinência)
Com que frequência você saliniza cateteres venosos periféricos intermitentes com solução fisiológica ou heparinizada?	0,80	1
Com que frequência você utiliza a mesma seringa para sorolização ou heparinização de cateteres venosos periféricos em diferentes pacientes?	1	1
Com que frequência você reutiliza a mesma tampinha, de fechamento de dupla via, em cateteres venosos periféricos após administração medicamentosa por esta via?	1	1
Com que frequência você armazena tampinha de cateter venoso periférico em mesas de cabeceira, bolsos de jaleco/avental ou outros locais para posterior reutilização desta?	1	1
Com que frequência você realiza a desobstrução de cateteres venosos periféricos com a infusão forçada de soro fisiológico?	1	1
Com que frequência realiza desinfecção de conectores com álcool a 70% antes da punção venosa periférica?	1	1
Com que frequência você reutiliza agulhas ou seringas para preparo de medicações do mesmo paciente?	0,80	0,80
Com que frequência você reutiliza agulhas ou seringas para preparo de medicações de pacientes diferentes?	1	1
Com que frequência você reutiliza seringas para salinização de pacientes diferentes?	0,80	0,80
Com que frequência você realiza antissepsia da pele do paciente com álcool a 70% para administrar medicações subcutâneas?	1	1
Com que frequência você realiza antissepsia da pele do paciente para realizar medicações, que não vacinas, pela via intramuscular?	1	1

Tabela 3 - Avaliação da concordância da adequação dos itens do domínio "administração medicamentosa” do Questionário para Identificação das Boas Práticas com Medicações Injetáveis. São Carlos, SP, Brasil, 2017.

(continuação)

Item	IVC (Clareza)	IVC (Pertinência)
Com que frequência você toca a área da pele do paciente em que já realizou antissepsia antes da administração do medicamento injetável?	1	1
Com que frequência você reutiliza seringas modificando a agulha desta para administração medicamentosa injetável no mesmo paciente ou em pacientes diferentes?	0,80	0,80
Com que frequência você realiza lavagem do acesso venoso após administração de medicamentos com SF0,9%?	1	1
Com que frequência você reutiliza materiais descartáveis como tampinhas de equipos ou polifix?	1	1
Com que frequência você recebe treinamento para manipulação de agulhas e cateteres com dispositivos de segurança?	1	1
Com que frequência você realiza ações de enfermagem com a integridade de suas mãos prejudicadas?	1*	1*
Com que frequência você utiliza luvas para administrar medicações intramusculares?	1	1
Com que frequência você utiliza luvas para administrar medicações subcutâneas?	1	1
Com que frequência você utiliza luvas para administrar medicamentos por via endovenosa?	1	1
Com que frequência você utiliza luvas para administrar medicamentos via endovenosa apenas para paciente sabidamente acometidos com vírus como HIV, hepatites B ou C?	1	1
Com que frequência realiza curativo estéril para manutenção dos cateteres de infusão medicamentosa?	1	1
Com que frequência realiza troca de acesso venoso periférico programada (entre 72 e 96 horas) antes dos sinais de flebite local?	1	1

Fonte: Dados da pesquisa
Nota: *N=4

Tabela 4 - Avaliação da concordância da adequação dos itens do domínio "cuidados após administração medicamentosa" do Questionário para Identificação das Boas Práticas com Medicações Injetáveis. São Carlos, SP, Brasil, 2017.

(conclusão)

**Item**	**IVC (Clareza)**	**IVC (Pertinência)**
Com que frequência você reencapa agulhas após realizar medicações no paciente antes de descartar os resíduos?	1	1
Com que frequência você higieniza as mãos antes e após a colocação de luvas para administração medicamentosa?	1	1
Com que frequência você transporta material como seringas e agulhas utilizadas desprotegidas até local adequado de descarte?	1	1
Com que frequência você separa a agulha do corpo da seringa após medicar pacientes para o descarte dos resíduos?	1	1
Com que frequência você se preocupa em vacinar-se contra doenças potencialmente passíveis de transmissão ocupacional, como a hepatite B?	1	1
Com que frequência você procura serviço de saúde ocupacional após exposição percutânea com material perfurocortante em até 72 horas após o acidente?	1	0,80
Com que frequência, após sofrer acidente com perfurocortante você julga adequado espremer a área afetada e colocar álcool a 70% para desinfetar a região para diminuir a probabilidade de infecção de patógenos transmitidos por via sanguínea?	1	1

Fonte: Dados da pesquisa

Como sugestões para melhoria dos itens, emergiram: modificação da redação de frases, inserção do termo salinização ou sorolização de forma padronizada, exemplificação de tipos de conectores, inserção de itens em outro domínio (referente ao uso de mesma seringa para preparo de medicações), manutenção da impessoalidade dos questionamentos e divisão de um item em dois quando este abordava duas práticas).

Houve também, a fim de viabilizar a análise estatística, a codificação dos itens e domínios, conforme sugerido por juiz.

As modificações realizadas e codificação dos itens do QBMI após validação podem ser verificadas no quadro 2.

Quadro 3 - Modificações realizadas no Questionário para Identificação das Boas Práticas com Medicações Injetáveis.

(continua)

Redação Inicial	Redação final	Código do item	Observação
Com que frequência você desinfeta balcão para preparo das medicações antes do início de suas atividades com álcool a 70% ou outras substâncias padronizadas em seu serviço?	Com que frequência higieniza o balcão para preparo das medicações, antes do início de suas atividades, com álcool a 70% ou outras soluções padronizadas em seu serviço?	DA1	-
Com que frequência você higieniza as bandejas de preparo e administração de medicações injetáveis a cada preparo destas?	Com que frequência higieniza as bandejas usadas no preparo e administração de medicações injetáveis antes de cada uso?	DA2	-
Com que frequência você verifica a data de validade de materiais utilizados para administração medicamentosa antes de sua utilização?	Com que frequência verifica a data de validade de materiais utilizados para administração de medicamentos antes de seu uso (por exemplo, seringas, agulhas)?	DA3	-
Com que frequência você utiliza materiais para administração medicamentosa cuja embalagem esteja aberta?	Com que frequência utiliza materiais para administração de medicamentos cujas embalagens estejam danificadas (por exemplo, tampinhas de equipo, cateteres e agulhas)?	DA4	-
Com que frequência você higieniza as mãos antes e após o preparo de medicações injetáveis?	Com que frequência higieniza as mãos antes e após o preparo de medicações injetáveis?	DA5	-
Com que frequência você compartilha frascos de soros (de 100 a 1000ml) no preparo de medicações no posto de enfermagem para reconstituição de doses medicamentosas?	Com que frequência compartilha frascos de soros (de 100 a 1000 mL) para reconstituição de doses de medicamentos?	DB1	-
Com que frequência você compartilha o mesmo frasco com multidoses para dois ou mais pacientes?	Com que frequência utiliza frascos multidoses para dois ou mais pacientes?	DB2	-
Com que frequência você realiza desinfecção com álcool a 70% das tampas de frascos multidoses antes da aspiração do medicamento a ser administrado?	Com que frequência realiza desinfecção com álcool a 70% das tampas de frascos multidoses antes da aspiração do medicamento a ser administrado?	DB3	-
Com que frequência você utiliza a mesma agulha para aspirar medicações de frascos multidoses?	Com que frequência utiliza a mesma agulha para aspirar medicações de frascos multidoses?	DB4	-

Quadro 2 - Modificações realizadas no Questionário para Identificação das Boas Práticas com Medicações Injetáveis.

(continuação)

Redação Inicial	Redação final	Código do item	Observação
Com que frequência você utiliza medicações de frascos que constem data de abertura do mesmo, volume de diluente (se aplicável), nome do profissional que realizou a abertura e reconstituiu a medicação?	Com que frequência identifica os frascos multidoses disponíveis na sua unidade com data de abertura, volume de diluente (se aplicável) e nome do profissional que reconstituiu a medicação?	DB4	-
Com que frequência você realiza assepsia de frascos e ampolas de medicação antes de aspirá-la?	Com que frequência realiza limpeza de frascos e ampolas de medicação com álcool a 70% antes de aspirá-la?	DB6	-
Com que frequência você reutiliza agulhas ou seringas para preparo de medicações do mesmo paciente?	Com que frequência reutiliza agulhas ou seringas para preparo de medicações do mesmo paciente?	DB7	Item removido do domínio “administração medicamentosa” para o domínio “preparo das medicações”
Com que frequência você reutiliza agulhas ou seringas para preparo de medicações de pacientes diferentes?	Com que frequência reutiliza agulhas ou seringas para preparo de medicações de pacientes diferentes?	DB8	Item removido do domínio “administração medicamentosa” para o domínio “preparo das medicações”
Com que frequência você saliniza cateteres venosos periféricos intermitentes com solução fisiológica ou heparinizada?	Com que frequência realiza a salinização de cateteres venosos periféricos intermitentes?	DB9	Item considerado no domínio “preparo das medicações” na análise de dados da coleta deste trabalho, contudo sugere-se manutenção deste item no domínio sobre administração medicamentosa para aplicações futuras do QBMI
Com que frequência você higieniza as mãos, com água e sabonete ou solução alcóolica, antes da administração de medicação endovenosas?	Com que frequência higieniza as mãos, com água e sabonete antes da administração de medicação endovenosa?	DC1	Item removido do domínio “preparo das medicações” para o domínio “administração medicamentosa”
Com que frequência você higieniza as mãos, com água e sabonete ou solução alcóolica, antes da administração de medicação endovenosas?	Com que frequência higieniza as mãos, com solução alcoólica ou soluções padronizadas, antes da administração de medicação endovenosa?	DC2	Item removido do domínio “preparo das medicações” para o domínio “administração medicamentosa”

Quadro 2 - Modificações realizadas no Questionário para Identificação das Boas Práticas com Medicações Injetáveis conforme sugestões dos juízes.

(continuação)

**Redação Inicial**	**Redação final**	**Código do item**	**Observação**
Com que frequência você utiliza a mesma seringa para sorolização ou heparinização de cateteres venosos periféricos em diferentes pacientes?	Com que frequência utiliza a mesma seringa para salinização de cateteres venosos periféricos em diferentes pacientes?	DC3	-
Com que frequência você reutiliza a mesma tampinha, de fechamento de dupla via, em cateteres venosos periféricos após administração medicamentosa por esta via?	Com que frequência reutiliza a mesma tampinha, de fechamento de dupla via, em cateteres venosos periféricos?	DC4	-
Com que frequência você armazena tampinha de cateter venoso periférico em mesas de cabeceira, bolsos de jaleco/avental ou outros locais para posterior reutilização desta?	Com que frequência armazena tampinha de cateter venoso periférico em mesas de cabeceira, bolsos de jaleco/avental ou outros locais para posterior reutilização desta?	DC5	-
Com que frequência você realiza a desobstrução de cateteres venosos periféricos com a infusão forçada de soro fisiológico?	Com que frequência realiza a desobstrução de cateteres venosos periféricos com a infusão forçada de soro fisiológico?	DC6	Incluído no domínio “administração medicamentosa”
Com que frequência você realiza a desobstrução de cateteres venosos periféricos com a infusão forçada de soro fisiológico?	Com que frequência realiza a desobstrução de cateteres venosos periféricos com a infusão forçada de solução de heparina?	DC7	Incluído no domínio “administração medicamentosa” acrescentado sobre a prática com heparina
Com que frequência realiza desinfecção de conectores com álcool a 70% antes da punção venosa periférica?	Com que frequência realiza desinfecção de conectores (dupla via, torneirinha ou injetor lateral de equipo) com álcool a 70% antes de administrar os medicamentos?	DC8	-
Com que frequência você reutiliza seringas para salinização de pacientes diferentes?	Com que frequência reutiliza a mesma seringa para salinização de acesso venoso periférico de pacientes diferentes?	DC9	Duplicado equivocadamente (DC3)

Quadro 2 - Modificações realizadas no Questionário para Identificação das Boas Práticas com Medicações Injetáveis conforme sugestões dos juízes.

(continuação)

Redação Inicial	Redação final	Código do item	Observação
Com que frequência você realiza assepsia da pele do paciente com álcool a 70% para administrar medicações subcutâneas?	Com que frequência realiza a antissepsia da pele do paciente com álcool a 70% para administração de medicações subcutâneas (exceto vacinas)?	DC10	-
Com que frequência você realiza assepsia da pele do paciente para realizar medicações, que não vacinas, pela via intramuscular?	Com que frequência realiza antissepsia da pele do paciente com álcool a 70% para administração de medicações intramusculares (exceto vacinas)?	DC11	-
Com que frequência você toca a área da pele do paciente em que já realizou assepsia antes da administração do medicamento injetável?	Com que frequência toca a área da pele do paciente, onde já realizou antissepsia, antes da administração do medicamento injetável?	DC12	-
Com que frequência você reutiliza seringas modificando a agulha desta para administração medicamentosa injetável no mesmo paciente ou em pacientes diferentes?	Com que frequência utiliza a mesma seringa, trocando apenas a agulha, para administração de medicamentos no mesmo paciente?	DC13	-
Com que frequência você reutiliza seringas modificando a agulha desta para administração medicamentosa injetável no mesmo paciente ou em pacientes diferentes?	Com que frequência utiliza a mesma seringa, trocando apenas a agulha, para administração de medicamentos em pacientes diferentes?	DC14	-
Com que frequência você realiza lavagem do acesso venoso após administração de medicamentos com SF0,9%?	Com que frequência realiza lavagem do acesso venoso periférico intermitente com SF 0,9% após administração de medicamentos?	DC15	-
Com que frequência você reutiliza materiais descartáveis como tampinhas de equipos ou polifix?	Com que frequência reutiliza materiais descartáveis como tampinhas de equipos ou dupla via?	DC16	-
Com que frequência você recebe treinamento para manipulação de agulhas e cateteres com dispositivos de segurança?	Com que frequência recebe treinamento para manipulação de agulhas e cateteres com dispositivos de segurança?	DC17	-

Quadro 2 - Modificações realizadas no Questionário para Identificação das Boas Práticas com Medicações Injetáveis.

(continuação)

Redação Inicial	Redação final	Código do item	Observação
Com que frequência você realiza ações de enfermagem com a integridade de suas mãos prejudicadas?	Com que frequência realiza administração de medicações com lesões de pele nas mãos?	DC18	-
Com que frequência você utiliza luvas para administrar medicações intramusculares?	Com que frequência utiliza luvas para administrar medicações intramusculares?	DC19	-
Com que frequência você utiliza luvas para administrar medicações subcutâneas?	Com que frequência utiliza luvas para administrar medicações subcutâneas?	DC20	-
Com que frequência você utiliza luvas para administrar medicamentos por via endovenosa?	Com que frequência utiliza luvas para administrar medicamentos por via endovenosa?	DC21	-
Com que frequência você utiliza luvas para administrar medicamentos via endovenosa apenas para paciente sabidamente acometidos com vírus como HIV, hepatites B ou C?	Com que frequência utiliza luvas para administrar medicamentos por via endovenosa apenas para paciente sabidamente acometidos com vírus como HIV, hepatites B ou C?	DC22	-
Com que frequência realiza curativo estéril para manutenção dos cateteres de infusão medicamentosa?	Com que frequência realiza curativo estéril para fixação/manutenção dos cateteres venosos periféricos?	DC23	-
Com que frequência realiza troca de acesso venoso periférico programada (entre 72 e 96 horas) antes dos sinais de flebite local?	Com que frequência realiza troca de acesso venoso periférico, conforme padronização do serviço, antes do aparecimento de sinais de flebite local?	DC24	-
Com que frequência você reencapa agulhas após realizar medicações no paciente antes de descartar os resíduos?	Com que frequência reencapa agulhas após realizar medicações?	DD1	-
Com que frequência você higieniza as mãos antes e após a colocação de luvas para administração medicamentosa?	Com que frequência higieniza as mãos antes e após o uso de luvas para administração de medicamentos?	DD2	-

Quadro 2 - Modificações realizadas no Questionário para Identificação das Boas Práticas com Medicações Injetáveis.

(conclusão)

Redação Inicial	Redação final	Código do item	Observação
Com que frequência você transporta material como seringas e agulhas utilizadas desprotegidas até local adequado de descarte?	Com que frequência transporta materiais usados, como seringas e agulhas, desprotegidos até o local de descarte?	DD3	-
Com que frequência você separa a agulha do corpo da seringa após medicar pacientes para o descarte dos resíduos?	Com que frequência separa a agulha do corpo da seringa para o descarte após medicar pacientes?	DD4	-
Com que frequência você se preocupa em vacinar-se contra doenças potencialmente passíveis de transmissão ocupacional, como a hepatite B?	Com que frequência procura informar-se sobre sua situação vacinal contra doenças potencialmente passíveis de transmissão ocupacional, como a hepatite B?	DD5	-
Com que frequência você procura serviço de saúde ocupacional após exposição percutânea com material perfurocortante em até 72 horas após o acidente?	Com que frequência costuma informar-se sobre o fluxo de atendimento pós exposição percutânea com material perfurocortante no seu local de trabalho?	DD6	-
Com que frequência, após sofrer acidente com perfurocortante você julga adequado espremer a área afetada e colocar álcool a 70% para desinfetar a região para diminuir a probabilidade de infecção de patógenos transmitidos por via sanguínea?	Com que frequência observa e/ou toma conhecimento de que pessoas da equipe de enfermagem após sofrer acidente com perfurocortante espremem a área afetada e colocam álcool a 70% para desinfetar a região?	DD7	-

Fonte: Dados da pesquisa

Desta forma, apesar de todos os itens atingirem IVC igual ou superior a 0,78 (COLUCI; ALEXANDRE; MILANI, 2015), as sugestões foram discutidas e analisadas em grupo de pesquisa, fazendo-se a adaptação necessária para melhorar a compreensão do instrumento pela população-alvo.

Houve, também, sugestão de inserção sobre eventos distratores ocorridos no ambiente por estar relacionado à segurança do paciente, descarte de algodão com sangue, inserção do item sobre vacinação do profissional no domínio “preparo do ambiente” ou “preparo da medicação” medicamentos especiais e luvas após procedimentos e elaboração de um domínio específico para acidentes ocupacionais, contudo, estas questões foram desconsideradas, no momento de discussão intragrupo de pesquisa.

A fim de melhor avaliar o instrumento, após encaminhá-lo para profissional estatístico, e para realização da análise dos dados da survey (vide seção 5.1.2) foi consolidado um escore para interpretação da prática referida dos profissionais, em que, quanto menor a pontuação mais adequada às boas práticas com injetáveis o respondente se encontra (tabela 5).

Tabela 5 - Pontuação do instrumento QBMI utilizado para coleta de dados referente às práticas de medicações injetáveis segundo número de itens. São Carlos-SP, Brasil. 2018.

Código do domínio	Domínio	Nº de Itens	Pontuação Máxima	Pontuação Mínima
DA	Preparo do ambiente	5	25	5
DB	Preparo das medicações	9	45	9
DC	Administração medicamentosa	24	120	24
DD	Cuidados após administração	7	35	7
TOTAL	-	45	225	45

Fonte: Dados da pesquisa

Ressalta-se que, a adequação da frequência desejável (variando entre “sempre” e “nunca”) a partir de cada prática questionada, norteou-se pelos achados na literatura, conforme abordado no “Apêndice F”.

Para tanto, foram consideradas referências como recomendações da OMS, da Agência Nacional de Vigilância Sanitária (ANVISA), do Centers for Disease Control and Prevention (CDC) (O’GRADY *et al.*, 2011), diretrizes (CONSELHO REGIONAL DE

ENFERMAGEM DE SÃO PAULO, 2010; SÃO PAULO, 2014) e pesquisas relevantes abordando antissepsia de pele (BINSWANGER *et al.*, 2000; D'AQUINO; SOUZA, 2012; HUTIN *et al.*, 2003; OLIVEIRA *et al.*, 2019; SEXSON; LINDAUER; HARVATH, 2017) e uso de luvas (MONCAIO; FIGUEIREDO, 2009).

Ressalta-se que o parecer COREN-SP CAT nº 014 / 2010, o qual discorria sobre o uso de luvas de procedimento para a administração de medicamentos, utilizado nesse trabalho, foi posteriormente revogado, conforme verificado em abril de 2021 em: https://portal.coren-sp.gov.br/wp-content/uploads/2021/01/CT_Parecer_014-2010_RL.pdf.

Desta forma, foram itens cuja frequência desejável é considerada "nunca" ou "quase nunca": DA4, DB1, DB2, DB4, DB7, DB8, DC3, DC4, DC5, DC6, DC7, DC9, DC12, DC13, DC14, DC16, DC18, DC22, DD1, DD3, DD4 e DD7.

Estes itens contemplam a frequência de:

- Utilização de materiais cujas embalagens estivessem violadas;
- Utilização de frascos de grandes volumes de solução para reconstituição de doses;
- Utilização de frascos multidoses para dois pacientes ou mais;
- Utilização de mesma agulha para aspiração de doses em frascos multidoses;
- Reutilização de agulhas para preparo de medicações;
- Reutilização de oclusores e seringa;
- Desobstrução forçada de cateteres;
- Adesão à técnica *no touch* para administração de injetáveis;
- Administração de injetáveis com lesões nas mãos;
- Uso de luvas para administração de injetáveis apenas para pacientes sabidamente com doenças infecciosas;
- Reencape de agulhas após sua utilização;
- Transporte inseguro de perfurocortantes utilizados até o local de descarte;
- Separação entre agulha e seringa após utilização para descarte;
- Cuidado inadequado com lesões por perfurocortante.

Quanto aos componentes DC10, DC19, DC20, estes não foram invertidos quanto à desejabilidade da frequência, mantendo o padrão dos demais componentes, pois, não foi identificado consenso sobre as práticas (antissepsia de pele para administração de

injetável via SC, uso de luvas para injetáveis via IM e SC), segundo busca na literatura (APÊNDICE F).

Há, portanto, possibilidade de adequação dos itens DC10, DC19 e DC20, conforme instituição de atuação e contexto de aplicação futura do QBMI.

Uma vez que a maioria dos participantes deste estudo referiu a frequência como “sempre” e não se buscou conhecer o contexto em que estas ações ocorriam, não foi possível realizar uma ponderação melhor para eles.

Quanto aos itens DC3 e DC9, devido a duplicação sobre reutilização de seringa para salinização de acesso venoso periférico de pacientes procedeu-se com a análise da concordância, o que permitiu verificar que não houve diferença significativa de frequência de respostas entre os itens (p=0,170), com concordância moderada (K>0.40): teste de simetria de Bowker: S=14,07; GL=10; P=0,170; Kappa ponderado: 0,409; IC 95%: (0,258; 0,559).

Desta forma, como ambos foram mantidos para a realização da análise estatística deste estudo, ocasionou um total de 45 itens para análise dos dados do presente estudo.

Para que possa ser utilizado, futuramente, considerando-se o processo de validação e a exclusão de item duplicado, obteve-se um instrumento para coleta de práticas referidas com injetáveis, contemplando 44 práticas com injetáveis a serem respondidas a partir de cinco opções de respostas (“sempre, quase sempre, às vezes, quase nunca e nunca”).

Ou seja, quanto mais próximo de 44 pontos, melhor a prática do sujeito que o responder, enquanto escores próximos a pontuação máxima de 220 configuram maior distância às Boas Práticas.

O instrumento supracitado com 44 itens (QBMI) encontra-se em apêndice G, em duas versões, uma para o avaliador e outra para o profissional ou estudante, com instruções no próprio instrumento.

5.1.2 Prática referida de profissionais de enfermagem sobre medicações injetáveis

Descrevem-se aqui os achados quanto à caracterização demográfica dos participantes desta etapa, bem como as práticas referidas com injetáveis segundo QBMI (APÊNDICE H).

5.1.2.1 Participantes da pesquisa

Ao todo, o QBMI recebeu 2056 acessos através do *link* https://pt.surveymonkey.com/r/boas_praticas_med_inj, que permaneceu ativo apenas para a coleta das condutas referidas com injetáveis.

Destes, 1338 acessaram ao TCLE, sendo 40 excluídos por não aceitarem participar do estudo.

Com isso, 1298 respostas do questionário na íntegra foram analisadas, sendo excluídos, posteriormente a esta análise, três questionários cujos participantes afirmaram não terem exercido atividades laborais relacionadas à enfermagem.

Ao todo, 1295 respostas foram consideradas para a prática referida dos profissionais de enfermagem.

Com relação à categoria profissional, 47,03% (609/1295) referiram ser enfermeiros, 39,07% (506/1295) técnicos de enfermagem e 21,08% (273/1295) auxiliares de enfermagem, enquanto 1,16% (15/1295) afirmaram outras ocupações, também (fisioterapeuta, graduação de enfermagem em curso, auxiliar de coleta laboratorial, inspetor de guarda civil metropolitano, cuidador de idosos, instrumentador cirúrgico, dentre outros).

Com relação à idade, esta obteve uma média de 38,44 (anos), mediana de 38 (anos), com desvio padrão de ±9,44, idade mínima de 18 e máxima de 74 anos.

Uma vez que o profissional poderia pertencer a mais de uma categoria, fora questionado sobre o maior grau de instrução. O qual pode ser visualizado no gráfico 1.

Gráfico 1 - Percentual de profissionais segundo grau de instrução. São Carlos, SP, 2018.

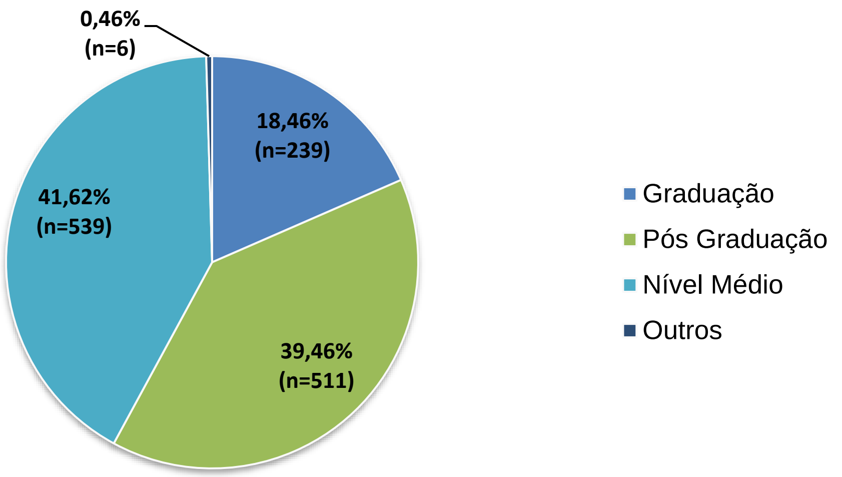

Fonte: Dados da pesquisa

Como pós-graduação foram referidos mestrado, especialização, doutorado, programa de residência, Mestre em Administração de Negócios (MBA) e auditoria em serviços de saúde. Já a categoria “outros” fora constituída por bacharel em direito, serviço social, pós-técnico, curso tecnólogo em gestão hospitalar e biomedicina.

Quanto ao tipo de serviço de exercício laboral, o hospitalar foi o predominantemente referido pelos participantes da pesquisa com 57,45% (744/1295), sendo seguido pela Atenção Básica com 16,14% (209/1295) e ambulatório referido por 12,74% (165/1295).

Apontamento para outros serviços (173/1295) foi constituído por locais como consultório de rua, Instituição de Longa Permanência (ILPI), Instituto de Pesquisa, cuidado domiciliar, Centro de Atenção Psicossocial (CAPS), Vigilância Sanitária, Centro de Reabilitação Psicossocial, indústria/empresa, Assistência Médica Ambulatorial (AMA), Serviço Especializado em Engenharia de Segurança e em Medicina do Trabalho (SESMT), penitenciária, escola e creche.

Em sua maioria, a atividade assistencial foi a mais referida atingindo 85,25% (1104/1295) das respostas, seguida por gerencial (227/1295) com 17,53% e de ensino referido por 11,27% (146/1295).

A partir do gráfico 2, abaixo, pode-se observar a quantidade de profissionais por tempo de trabalho (em anos) referente às atividades dentro da área da enfermagem.

Gráfico 2 - Percentual de profissionais segundo tempo de trabalho (em anos). São Carlos, SP, 2018.

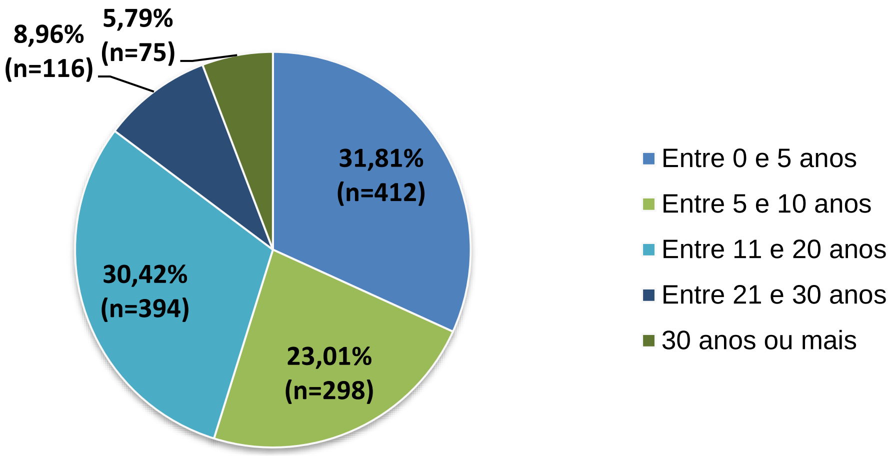

Fonte: Dados da pesquisa

5.1.2.2 Prática referida com Medicações Injetáveis

A frequência com que a população deste estudo referiu realizar as atividades pertinentes a cada domínio do QBMI pode ser visualizada nas tabelas a seguir:

Tabela 6 - Frequência referida de atividades relacionadas ao preparo do ambiente pelos profissionais do estado de São Paulo. São Carlos, SP, 2018.

**Item**	**Sempre**		**Quase sempre**		**Às vezes**		**Quase nunca**		**Nunca**	
	**N**	**%**	**N**	**%**	**N**	**%**	**N**	**%**	**N**	**%**
DA1- Com que frequência higieniza o balcão para preparo das medicações, antes do início de suas atividades, com álcool a 70% ou outras soluções padronizadas em seu serviço?	831	64,17	289	22,32	117	9,03	41	3,17	17	1,31
DA2- Com que frequência higieniza as bandejas usadas no preparo e administração de medicações injetáveis antes de cada uso?	966	74,59	231	17,84	61	4,71	20	1,54	17	1,31
DA3- Com que frequência verifica a data de validade de materiais utilizados para administração de medicamentos antes de seu uso (por exemplo, seringas, agulhas)?	747	57,68	255	19,69	136	10,50	101	7,80	56	4,32
DA4- Com que frequência utiliza materiais para administração de medicamentos cujas embalagens estejam danificadas (por exemplo, tampinhas de equipo, cateteres e agulhas)?	98	7,57	16	1,24	30	2,32	84	6,49	1067	82,39
DA5- Com que frequência higieniza as mãos antes e após o preparo de medicações injetáveis?	1113	85,95	144	11,12	25	1,93	9	0,69	4	0,31

Fonte: Dados da pesquisa

Tabela 7 - Frequência referida de atividades relacionadas ao preparo das medicações pelos profissionais do estado de São Paulo. São Carlos, SP, 2018.

	**Sempre**		**Quase sempre**		**Às vezes**		**Quase nunca**		**Nunca**	
	**N**	**%**	**N**	**%**	**N**	**%**	**N**	**%**	**N**	**%**
DB1- Com que frequência compartilha frascos de soros (de 100 a 1000 mL) para reconstituição de doses de medicamentos?	95	7,34	126	9,73	184	14,21	199	15,37	691	53,36
DB2- Com que frequência utiliza frascos multidoses para dois ou mais pacientes?	140	10,81	129	9,96	167	12,90	163	12,59	696	53,75
DB3- Com que frequência realiza desinfecção com álcool a 70% das tampas de frascos multidoses antes da aspiração do medicamento a ser administrado?	833	64,32	209	16,14	106	8,19	67	5,17	80	6,18
DB4- Com que frequência utiliza a mesma agulha para aspirar medicações de frascos multidoses?	52	4,02	79	6,10	102	7,88	117	9,03	945	72,97
DB5- Com que frequência identifica os frascos multidoses disponíveis na sua unidade com data de abertura, volume de diluente (se aplicável) e nome do profissional que reconstituiu a medicação?	969	74,83	117	9,03	57	4,40	38	2,93	114	8,80
DB6- Com que frequência realiza limpeza de frascos e ampolas de medicação com álcool a 70% antes de aspirá-la?	826	63,78	203	15,68	106	8,19	75	5,79	85	6,56
DB7- Com que frequência reutiliza agulhas ou seringas para preparo de medicações do mesmo paciente?	31	2,39	55	4,25	74	5,71	102	7,88	1033	79,77
DB8- Com que frequência reutiliza agulhas ou seringas para preparo de medicações de pacientes diferentes?	7	0,54	4	0,31	8	0,62	21	1,62	1255	96,91
DB9- Com que frequência realiza a salinização de cateteres venosos periféricos intermitentes?	893	68,96	170	13,13	99	7,64	44	3,40	89	6,87

Fonte: Dados da pesquisa

Tabela 8 - Frequência referida de atividades relacionadas à administração das medicações injetáveis pelos profissionais do estado de São Paulo. São Carlos, SP, 2018.

(continua)

Item	Sempre		Quase sempre		Às vezes		Quase nunca		Nunca	
	n	%	n	%	n	%	n	%	n	%
DC1- Com que frequência higieniza as mãos, com água e sabonete antes da administração de medicação endovenosa?	923	71,27	216	16,68	92	7,10	24	1,85	40	3,09
DC2- Com que frequência higieniza as mãos, com solução alcoólica ou soluções padronizadas, antes da administração de medicação endovenosa?	854	65,95	219	16,91	138	10,66	43	3,32	41	3,17
DC3- Com que frequência utiliza a mesma seringa para salinização de cateteres venosos periféricos em diferentes pacientes?	16	1,24	5	0,39	8	0,62	16	1,24	1250	96,53
DC4- Com que frequência reutiliza a mesma tampinha, de fechamento de dupla via, em cateteres venosos periféricos?	107	8,26	201	15,52	221	17,07	130	10,04	636	49,11
DC5- Com que frequência armazena tampinha de cateter venoso periférico em mesas de cabeceira, bolsos de jaleco/avental ou outros locais para posterior reutilização desta?	30	2,32	71	5,48	136	10,50	128	9,88	930	71,81
DC6- Com que frequência realiza a desobstrução de cateteres venosos periféricos com a infusão forçada de soro fisiológico?	117	9,03	145	11,20	332	25,64	229	17,68	472	36,45
DC7- Com que frequência realiza a desobstrução de cateteres venosos periféricos com a infusão forçada de solução de heparina?	13	1,00	15	1,16	51	3,94	108	8,34	1108	85,56

Tabela 8 - Frequência referida de atividades relacionadas à administração das medicações injetáveis pelos profissionais do estado de São Paulo. São Carlos, SP, 2018.

(continuação)

Item	Sempre		Quase sempre		Às vezes		Quase nunca		Nunca	
	n	%	n	%	n	%	n	%	n	%
DC8- Com que frequência realiza desinfecção de conectores (dupla via, torneirinha ou injetor lateral de equipo) com álcool a 70% antes de administrar os medicamentos?	748	57,76	179	13,82	119	9,19	74	5,71	175	13,51
DC9- Com que frequência reutiliza a mesma seringa para salinização de acesso venoso periférico de pacientes diferentes?	6	0,46	4	0,31	6	0,46	17	1,31	1262	97,45
DC10- Com que frequência realiza a antissepsia da pele do paciente com álcool a 70% para administração de medicações subcutâneas (exceto vacinas)?	1177	90,89	42	3,24	22	1,70	12	0,93	42	3,24
DC11- Com que frequência realiza antissepsia da pele do paciente com álcool a 70% para administração de medicações intramusculares (exceto vacinas)?	1245	96,14	19	1,47	10	0,77	6	0,46	15	1,16
DC12- Com que frequência toca a área da pele do paciente, onde já realizou antissepsia, antes da administração do medicamento injetável?	57	4,40	38	2,93	126	9,73	218	16,83	856	66,10
DC13- Com que frequência utiliza a mesma seringa, trocando apenas a agulha, para administração de medicamentos no mesmo paciente?	70	5,41	29	2,24	57	4,40	73	5,64	1066	82,32
DC14- Com que frequência utiliza a mesma seringa, trocando apenas a agulha, para administração de medicamentos em pacientes diferentes?	10	0,77	1	0,08	5	0,39	8	0,62	1271	98,15

Tabela 8 - Frequência referida de atividades relacionadas à administração das medicações injetáveis pelos profissionais do estado de São Paulo. São Carlos, SP, 2018.

(conclusão)

Item	Sempre		Quase sempre		Às vezes		Quase nunca		Nunca	
	**n**	**%**	**n**	**%**	**n**	**%**	**n**	**%**	**n**	**%**
DC15- Com que frequência realiza lavagem do acesso venoso periférico intermitente com SF 0,9% após administração de medicamentos?	865	66,80	165	12,74	118	9,11	39	3,01	108	8,34
DC16- Com que frequência reutiliza materiais descartáveis como tampinhas de equipos ou dupla via?	105	8,11	66	5,10	127	9,81	121	9,34	876	67,64
DC17- Com que frequência recebe treinamento para manipulação de agulhas e cateteres com dispositivos de segurança?	168	12,97	160	12,36	353	27,26	351	27,10	263	20,31
DC18- Com que frequência realiza administração de medicações com lesões de pele nas mãos?	40	3,09	43	3,32	178	13,75	274	21,16	760	58,69
DC19 - Com que frequência utiliza luvas para administrar medicações intramusculares?	847	65,4	97	7,49	121	9,34	116	8,96	114	8,80
DC20 - Com que frequência utiliza luvas para administrar medicações subcutâneas?	770	59,46	101	7,80	113	8,73	150	11,58	161	12,43
DC21- Com que frequência utiliza luvas para administrar medicamentos por via endovenosa?	1042	80,46	104	8,03	64	4,94	47	3,63	38	2,93
DC22- Com que frequência utiliza luvas para administrar medicamentos por via endovenosa apenas para paciente sabidamente acometidos com vírus como HIV, hepatites B ou C?	1090	84,17	20	1,54	14	1,08	15	1,16	156	12,05
DC23- Com que frequência realiza curativo estéril para fixação/manutenção dos cateteres venosos periféricos?	642	49,58	148	11,43	136	10,50	144	11,12	225	17,37
DC24- Com que frequência realiza troca de acesso venoso periférico, conforme padronização do serviço, antes do aparecimento de sinais de flebite local?	938	72,43	179	13,82	69	5,33	35	2,70	74	5,71

Fonte: Dados da pesquisa

Tabela 9 - Frequência referida de atividades relacionadas aos cuidados após a administração medicamentosa pelos profissionais do estado de São Paulo. São Carlos, SP, 2018.

Item	Sempre		Quase sempre		Às vezes		Quase nunca		Nunca	
	n	%	n	%	n	%	n	%	n	%
DD1- Com que frequência reencapa agulhas após realizar medicações?	63	4,86	59	4,56	117	9,03	133	10,27	923	71,27
DD2- Com que frequência higieniza as mãos antes e após o uso de luvas para administração de medicamentos?	1073	82,86	154	11,89	47	3,63	7	0,54	14	1,08
DD3- Com que frequência transporta materiais usados, como seringas e agulhas, desprotegidos até o local de descarte?	260	20,08	134	10,35	189	14,59	212	16,37	500	38,61
DD4- Com que frequência separa a agulha do corpo da seringa para o descarte após medicar pacientes?	203	15,68	88	6,80	123	9,50	125	9,65	756	58,38
DD5- Com que frequência procura informar-se sobre sua situação vacinal contra doenças potencialmente passíveis de transmissão ocupacional, como a hepatite B?	796	61,47	182	14,05	171	13,20	88	6,80	58	4,48
DD6- Com que frequência costuma informar-se sobre o fluxo de atendimento pós exposição percutânea com material perfurocortante no seu local de trabalho?	566	43,71	196	15,14	237	18,30	164	12,66	132	10,19
DD7- Com que frequência observa e/ou toma conhecimento de que pessoas da equipe de enfermagem após sofrer acidente com perfurocortante espremem a área afetada e colocam álcool a 70% para desinfetar a região?	194	14,98	166	12,82	200	15,44	212	16,37	523	40,39

Fonte: Dados da pesquisa

A fim de melhor configurar o cenário das práticas com injetáveis, buscou-se realizar a análise descritiva e testes estatísticos para identificar diferença significativa entre variáveis como categorias profissionais, tempo de trabalho (agrupado em ≤10 ou >10 anos para maior consistência nas análises comparativas) e tipos de serviços.

De forma geral, conforme tabela 10, observa-se que houve diferença estatística apenas no domínio “preparo do ambiente”, na comparação entre graus de instrução, em que participantes do nível médio obtiveram melhor prática referida.

Tabela 10 - Análise descritiva de escore de domínios e do instrumento segundo graus de instrução profissionais. São Carlos, SP, 2018.

Grau de Instrução	Domínio	N	Média	DP	Mínimo	Q1	Mediana	Q3	Máximo	Valor-p*
Nível Médio	Preparo do Ambiente	538	6,99	2,27	5,00	5,00	6,00	8,00	18,00	
	Preparo das Medicações	538	14,72	4,58	9,00	11,00	14,00	17,00	36,00	
	Administração Medicamentosa	538	43,04	8,79	24,00	37,00	42,50	48,00	72,00	
	Cuidados após administração medicamentosa	538	13,99	4,10	7,00	11,00	14,00	17,00	26,00	
	Escore total	538	78,74	15,09	49,00	68,00	77,50	87,00	139,00	
Graduação	Preparo do Ambiente	239	7,35	2,43	5,00	5,00	7,00	9,00	16,00	<0,01
	Preparo das Medicações	239	15,19	4,32	9,00	12,00	14,00	18,00	30,00	0,110
	Administração Medicamentosa	239	43,01	8,65	28,00	36,00	42,00	49,00	68,00	0,357
	Cuidados após administração medicamentosa	239	14,49	4,09	7,00	11,00	14,00	18,00	27,00	0,354
	Escore total	239	80,04	14,19	55,00	69,00	79,00	90,00	128,00	0,080
Pós-graduação	Preparo do Ambiente	511	7,81	2,83	5,00	6,00	7,00	9,00	21, 00	
	Preparo das Medicações	511	15,17	4,61	9,00	12,00	15,00	18,00	34,00	
	Administração Medicamentosa	511	43,85	9,36	25,00	37,00	43,00	50,00	77,00	
	Cuidados após administração medicamentosa	511	14,04	3,89	7,00	11,00	14,00	16,00	27,00	
	Escore total	511	80,87	15,98	49,00	69,00	80,00	91,00	134,00	

Fonte: Dados da pesquisa

Nota: DP: Desvio-Padrão; Q1: Primeiro Quartil; Q3: Terceiro Quartil, Valor-P referente ao teste de Kruskal-Wallis para comparação dos valores entre os 3 grupos.

Sobre o preparo do ambiente, foi observado diferença significativa entre os três níveis de graus de instrução para a limpeza de balcão para preparo das medicações, limpeza de bandejas e verificação da data de validade de insumos antes do uso, conforme destacado em negrito, na tabela 11.

Tabela 11 - Diferença significativa entre os graus se instrução de acordo com componentes do instrumento de coleta de dados sobre preparo do ambiente para administração de medicações injetáveis segundo frequência referida. São Carlos, SP, 2018.

**Código**	**Prática**	**Grau de Instrução**	**Sempre**		**Quase Sempre**		**Às Vezes**		**Quase Nunca**		**Nunca**	
			**n**	**%**	**n**	**%**	**n**	**%**	**n**	**%**	**n**	**%**
DA1	Verificação da data de validade de insumos antes do uso	NM (N=538)	396	**73,61**	95	17,66	37	6,88	5	0,93	5	0,93
		G (N=239)	149	62,34	55	**23,01**	22	**9,21**	8	**3,35**	5	2,09
		PG (N=511)	280	54,79	138	**27,01**	58	**11,35**	28	**5,48**	7	1,37
TESTE QUI-QUADRADO: X2=49,47; GL=8; P<0,001												
DA2	Limpeza de bandejas a cada preparo de medicações injetáveis	NM (N=538)	436	**81,04**	78	14,50	16	2,97	5	0,93	3	0,56
		G (N=239)	178	74,48	44	**18,41**	11	4,60	4	1,67	2	0,84
		PG (N=511)	346	67,71	108	**21,14**	34	6,65	11	2,15	12	2,35
TESTE QUI-QUADRADO: X2=29,71; GL=8; P<0,001												
DA3	Verificação da data de validade de insumos antes do uso	NM (N=538)	340	**63,20**	96	17,84	53	9,85	30	5,58	19	3,53
		G (N=239)	128	53,56	59	**24,69**	25	10,46	17	7,11	10	4,18
		PG (N=511)	272	53,23	100	19,57	58	11,35	54	**10,57**	27	5,28
TESTE QUI-QUADRADO: X2=20,20; GL=8; P=0,010												
DA5	Higienização das mãos antes e após o preparo de medicações injetáveis	NM (N=538)	474	**88,10**	56	10,41	6	1,12	2	0,37	0	0,00
		G (N=239)	214	**89,54**	21	8,79	4	1,67	0	0,00	0	0,00
		PG (N=511)	419	82,00	66	**12,92**	15	2,94	7	1,37	4	0,78
TESTE EXATO DE FISHER: P=0,010												

Fonte: Dados da pesquisa

Nota: NM: nível médio; G: Graduado; PG: pós-graduado; GL: Grau de liberdade; P: valor-p

Já no domínio preparo das medicações, foi observado diferença significativa entre os três níveis de graus de instrução para o compartilhamento de frascos de solução fisiológica para reconstituição de doses medicamentosas, utilização de frascos multidoses para dois ou mais pacientes e identificação de frascos multidoses, conforme destacado em negrito, na tabela 12.

Tabela 12 - Diferença significativa entre os graus se instrução de acordo com componentes do instrumento de coleta de dados sobre preparo das medicações para administração de medicações injetáveis segundo frequência referida. São Carlos, SP, 2018

Código	Prática	Grau de Instrução	Sempre		Quase Sempre		Às Vezes		Quase Nunca		Nunca	
			n	%	n	%	n	%	n	%	n	%
DB1	Compartilhamento de frascos de solução fisiológica para reconstituição de doses medicamentosas	NM (N=538)	48	**8,92**	56	10,41	67	12,45	70	13,01	297	**55,20**
		G (N=239)	22	**9,21**	25	10,46	30	12,55	33	13,81	129	53,97
		PG (N=511)	25	4,89	45	8,81	87	**17,03**	95	**18,59**	259	50,68
TESTE QUI-QUADRADO: X2=19,20; GL=8; P=0,014												
DB2	Utilização de frascos multidoses para dois ou mais pacientes	NM (N=538)	55	10,22	48	8,92	57	10,59	68	12,64	310	**57,62**
		G (N=239)	30	12,55	29	12,13	28	11,72	18	7,53	134	**56,07**
		PG (N=511)	55	10,76	52	10,18	81	**15,85**	76	**14,87**	247	48,34
TESTE QUI-QUADRADO: X2=20,00; GL=8; P=0,010												
DB5	Identificação de frascos multidoses (data, volume do diluente, nome do profissional)	NM (N=538)	422	**78,44**	37	6,88	21	3,90	12	2,23	46	8,55
		G (N=239)	176	73,64	33	**13,81**	4	1,67	8	3,35	18	7,53
		PG (N=511)	366	71,62	47	9,20	30	**5,87**	18	3,52	50	9,78
TESTE QUI-QUADRADO: X2=20,12; GL=8; P=0,010												

Fonte: (Dados de pesquisa. São Carlos, 2018)
Nota: NM: nível médio; G: Graduado; PG: pós-graduado; GL: Grau de liberdade; P: valor-p

Na tabela 13, em negrito, destacam-se diferenças significativas entre os três níveis de graus de instrução, referentes ao domínio administração medicamentosa, os quais contemplam a HM com água e sabão antes da administração medicamentosa, desobstrução forçada com solução fisiológica 0,9% de CVP, quebra da técnica *no touch* para administração de injetáveis, treinamento para manipulação de agulhas e cateteres com dispositivos de segurança, administração de medicações com lesões de pele nas mãos, e uso de luvas para administração de injetáveis em diferentes situações.

Tabela 13 - Diferença significativa entre os graus se instrução de acordo com componentes do instrumento de coleta de dados sobre administração medicamentosa segundo frequência referida. São Carlos, SP, 2018.

(continua)

**Código**	**Prática**	**Grau de Instrução**	**Sempre**		**Quase Sempre**		**Às Vezes**		**Quase Nunca**		**Nunca**	
			**n**	**%**	**n**	**%**	**n**	**%**	**n**	**%**	**n**	**%**
DC1	Higienização das mãos com água e sabão antes da administração de medicação endovenosa	NM (N=538)	409	**76,02**	75	13,94	31	5,76	6	1,12	17	3,16
		G (N=239)	171	**71,55**	41	17,15	17	7,11	4	1,67	6	2,51
		PG (N=511)	337	65,95	99	**19,37**	44	8,61	14	2,74	17	3,33
TESTE QUI-QUADRADO: X2=15,52; GL=8; P=0,049												
DC6	Desobstrução forçada com solução fisiológica 0,9% de cateteres venosos periféricos	NM (N=538)	67	**12,45**	60	11,15	135	25,09	85	15,80	191	35,50
		G (N=239)	17	7,11	25	10,46	63	26,36	36	15,06	98	**41,00**
		PG (N=511)	33	6,46	60	11,74	132	25,83	106	**20,74**	180	35,23
TESTE QUI-QUADRADO: X2=18,42; GL=8; P=0,018												
DC12	Quebra da técnica *no touch* para administração de injetáveis	NM (N=538)	27	5,02	12	2,23	38	7,06	82	15,24	379	**70,45**
		G (N=239)	8	3,35	6	2,51	35	**14,64**	33	13,81	157	65,69
		PG (N=511)	22	4,31	20	3,91	53	10,37	102	**19,96**	314	61,45
TESTE QUI-QUADRADO: X2=22,10; GL=8; P=0,005												

Tabela 13 - Diferença significativa entre os graus se instrução de acordo com componentes do instrumento de coleta de dados sobre administração medicamentosa segundo frequência referida. São Carlos, SP, 2018.

(continuação)

Código	Prática	Grau de Instrução	Sempre		Quase Sempre		Às Vezes		Quase Nunca		Nunca	
			n	%	n	%	n	%	n	%	n	%
DC17	Treinamento para manipulação de agulhas e cateteres com dispositivos de segurança	NM (N=538)	89	**16,54**	55	10,22	137	25,46	144	26,77	113	21,00
		G (N=239)	27	11,30	33	13,81	51	21,34	75	**31,38**	53	22,18
		PG (N=511)	52	10,18	70	13,70	163	**31,90**	130	25,44	96	18,79
TESTE QUI-QUADRADO: X2=23,00; GL=8; P=0,003												
DC18	Administração de medicações com lesões de pele nas mãos	NM (N=538)	16	2,97	19	3,53	63	11,71	89	16,54	351	**65,24**
		G (N=239)	6	2,51	8	3,35	33	13,81	58	**24,27**	134	56,07
		PG (N=511)	18	3,52	16	3,13	82	16,05	126	**24,66**	269	52,64
TESTE QUI-QUADRADO: X2=21,14; GL=8; P=0,007												
DC19	Uso de luvas para administração de medicações intramusculares	NM (N=538)	378	**70, 26**	35	6,51	42	7,81	40	7,43	43	7,99
		G (N=239)	159	**66,53**	22	9,21	27	11,30	20	8,37	11	4,60
		PG (N=511)	305	59,69	40	7,83	52	10,18	54	10,57	60	**11,74**
TESTE QUI-QUADRADO: X2=22,07; GL=8; P=0,005												
DC20	Uso de luvas para administração de medicações subcutâneas	NM (N=538)	356	**66,17**	26	4,83	48	8,92	48	8,92	60	11,15
		G (N=239)	143	**59,83**	23	9,62	26	10,88	28	11,72	19	7,95
		PG (N=511)	267	52,25	51	9,98	39	7,63	73	**14,29**	81	**15,85**
TESTE QUI-QUADRADO: X2=36,68; GL=8; P<0,001												

Tabela 13 - Diferença significativa entre os graus se instrução de acordo com componentes do instrumento de coleta de dados sobre administração medicamentosa segundo frequência referida. São Carlos, SP, 2018.

(conclusão)

Código	Prática	Grau de Instrução	Sempre		Quase Sempre		Às Vezes		Quase Nunca		Nunca	
			**n**	**%**	**n**	**%**	**n**	**%**	**n**	**%**	**n**	**%**
DC22	Uso de luvas para administração de medicações endovenosas apenas para paciente sabidamente acometidos com vírus como HIV, hepatites B ou C	NM (N=538)	479	**89,03**	8	1,49	2	0,37	4	0,74	45	8,36
DC22	Uso de luvas para administração de medicações endovenosas apenas para paciente sabidamente acometidos com vírus como HIV, hepatites B ou C	G (N=239)	194	81,17	3	1,26	6	2,51	2	0,84	34	**14,23**
		PG (N=511)	411	80,43	9	1,76	6	1,17	9	1,76	76	**14,87**
TESTE QUI-QUADRADO: X2=22,93; GL=8; P=0,004												

Fonte: Dados da pesquisa

Nota: NM: nível médio; G: Graduado; PG: pós-graduado; GL: Grau de liberdade; P: valor-p

Já na tabela 14, em negrito, destacam-se diferenças significativas entre os três níveis de graus de instrução, referentes ao domínio cuidados após administração medicamentosa, os quais contemplam o reencape de agulha após administração medicamentosa, a separação entre agulha e corpo da seringa para o descarte após medicar pacientes e ciência sobre fluxo de atendimento pós exposição percutânea no local de trabalho.

Tabela 14 - Diferença significativa entre os graus se instrução de acordo com componentes do instrumento de coleta de dados sobre cuidados após administração medicamentosa segundo frequência referida. São Carlos, SP, 2018.

Código	Prática	Grau de Instrução	Sempre		Quase Sempre		Às Vezes		Quase Nunca		Nunca	
			**n**	**%**	**n**	**%**	**n**	**%**	**n**	**%**	**n**	**%**
DD1	Reencape de agulha após administração medicamentosa	NM (N=538)	31	5,76	18	3,35	38	7,06	42	7,81	409	**76,02**
		G (N=239)	13	5,44	14	5,86	26	**10,88**	22	9,21	164	68,62
		PG (N=511)	19	3,72	27	5,28	53	10,37	68	**13,31**	344	67,32
TESTE QUI-QUADRADO: X2=20,92; GL=8; P=0,007												
DD4	Separação entre agulha e corpo da seringa para o descarte após medicar pacientes	NM (N=538)	96	**17,84**	33	6,13	50	9,29	41	7,62	318	59,11
		G (N=239)	52	**21,76**	12	5,02	23	9,62	15	6,28	137	57,32
		PG (N=511)	55	10,76	42	8,22	49	9,59	67	**13,11**	298	58,32
TESTE QUI-QUADRADO: X2=29,72; GL=8; P<0,001												
DD6	Ciência sobre fluxo de atendimento pós exposição percutânea com material perfurocortante no seu local de trabalho	NM (N=538)	212	39,41	81	15,06	107	19,89	71	13,20	67	**12,45**
		G (N=239)	102	42,68	33	13,81	36	15,06	32	13,39	36	**15,06**
		PG (N=511)	249	**48,73**	80	15,66	92	18,00	61	11,94	29	5,68
TESTE QUI-QUADRADO: X2=26,60; GL=8; P<0,001												

Fonte: Dados da pesquisa

Nota: NM: nível médio; G: Graduado; PG: pós-graduado; GL: Grau de liberdade; P: valor-p

Não foi encontrada diferença significativa entre o tempo de atuação profissional e melhores escores no QBM, visto tabela 15.

Tabela 15 - Análise descritiva de escore de domínios e do instrumento segundo tempo de atuação profissional. São Carlos, SP, 2018.

Tempo de atuação na enfermagem	Domínio	N	Média	D.P.	Mínimo	Q1	Mediana	Q3	Máximo	Valor-p*
0-10 anos	Preparo do Ambiente	710	7,44	2,58	5,00	5,00	7,00	9,00	21,00	0,218
	Preparo das Medicações	710	15,02	4,69	9,00	12,00	14,00	18,00	36,00	0,920
	Administração Medicamentosa	710	43,39	9,03	26,00	37,00	43,00	49,00	77,00	0,978
	Cuidados após administração medicamentosa	710	14,24	4,04	7,00	11,00	14,00	17,00	27,00	0,179
	Escore total	710	80,09	15,60	49,00	69,00	79,00	89,00	139,00	0,489
>10 anos	Preparo do Ambiente	585	7,29	2,53	5,00	5,00	6,00	9,00	19,00	
	Preparo das Medicações	585	14,91	4,38	9,00	12,00	14,00	18,00	32,00	
	Administração Medicamentosa	585	43,31	8,97	24,00	37,00	43,00	49,00	72,00	
	Cuidados após administração medicamentosa	585	13,93	3,97	7,00	11,00	14,00	16,00	27,00	
	Escore total	585	79,43	14,96	49,00	68,00	78,00	90,00	129,00	

Fonte: Dados da pesquisa

Nota: Valor-P referente ao teste de Mann-Whitney para comparação dos valores entre os 2 grupos

Na análise estatística entre aqueles com atuação profissional até dez anos e os com tempo superior a dez anos, foram encontradas diferenças significativas para salinização de CVP, HM com água e sabão antes da administração medicamentosa via EV, antissepsia da pele do paciente com álcool a 70% para administração de medicações via IM, uso de luvas em diferentes situações, ciência sobre fluxo de atendimento pós exposição percutânea no local de trabalho e ciência sobre cuidados inapropriados com lesão decorrente deste.

Tais dados são verificados na tabela 16.

Tabela 16 - Diferença significativa entre o tempo de atuação em anos na área com componentes do instrumento de coleta de dados sobre as etapas para a administração de medicações injetáveis segundo frequência referida. São Carlos, SP, 2018.

(continua)

**Código**	**Prática**	**Grau de Instrução**	**Sempre**		**Quase Sempre**		**Às Vezes**		**Quase Nunca**		**Nunca**	
			**n**	**%**	**n**	**%**	**n**	**n**	**%**	**n**	**%**	**n**
DB9	Salinização de cateteres venosos periféricos	0-10 anos (N=710)	499	**70,28**	106	**14,93**	48	6,76	15	2,11	42	5,92
		>10 anos (N=585)	394	67,35	64	10,94	51	8,72	29	**4,96**	47	**8,03**
TESTE QUI-QUADRADO: X2=15,63; GL=4; P=0,004												
DC1	Higienização das mãos com água e sabão antes da administração medicamentosa	0-10 anos (N=710)	491	69,15	118	16,62	56	**7,89**	19	**2,68**	26	**3,66**
		>10 anos (N=585)	432	**73,85**	98	16,75	36	6,15	5	0,85	14	2,39
TESTE QUI-QUADRADO: X2=9,76; GL=4; P=0,045												
DC11	Antissepsia da pele do paciente com álcool a 70% para administração de medicações intramusculares	0-10 anos (N=710)	681	95,92	13	**1,83**	3	0,42	1	0,14	12	1,69
		>10 anos (N=585)	564	96,41	6	1,03	7	**1,20**	5	**0,85**	3	0,51
TESTE EXATO DE FISHER: P=0,021												
DC19	Uso de luvas para administração de medicações intramusculares	0-10 anos (N=710)	519	**73,10**	50	7,04	50	7,04	43	6,06	48	6,76
		>10 anos (N=585)	328	56,07	47	8,03	71	**12,14**	73	**12,48**	66	**11,28**
TESTE QUI-QUADRADO: X2=45,77; GL=4; P<0,001												
DC20	Uso de luvas para administração de medicações subcutâneas	0-10 anos (N=710)	480	**67,61**	49	6,90	48	6,76	66	9,30	67	9,44
		>10 anos (N=585)	290	49,57	52	8,89	65	**11,11**	84	**14,36**	94	**16,07**
TESTE QUI-QUADRADO: X2=44,57; GL=4; P<0,001												

Tabela 16 - Diferença significativa entre o tempo de atuação em anos na área com componentes do instrumento de coleta de dados sobre as etapas para a administração de medicações injetáveis segundo frequência referida. São Carlos, SP, 2018.

(conclusão)

**Código**	**Prática**	**Sempre**	**Quase Sempre**		**Às Vezes**		**Quase Nunca**		**Nunca**		**Sempre**	
		**n**	**%**	**n**	**%**	**n**	**n**	**%**	**n**	**%**	**n**	**n**
DC21	Uso de luvas para administração de medicações endovenosas	0-10 anos (N=710)	586	**82,54**	56	7,89	22	3,10	23	3,24	23	3,24
		>10 anos (N=585)	456	77,95	48	8,21	42	**7,18**	24	4,10	15	2,56
TESTE QUI-QUADRADO: X2=12,84; GL=4; P=0,012												
DD6	Ciência sobre fluxo de atendimento pós exposição percutânea com material perfurocortante no seu local de trabalho	0-10 anos (N=710)	283	39,86	115	16,20	121	17,04	102	14,37	89	**12,54**
		>10 anos (N=585)	283	**48,38**	81	13,85	116	19,83	62	10,60	43	7,35
TESTE QUI-QUADRADO: X2=19,91; GL=4; P<0,001												
DD7	Cuidados inapropriados com lesão decorrente de acidente ocupacional com perfurocortante	0-10 anos (N=710)	92	12,96	71	10,00	101	14,23	100	14,08	346	**48,73**
		>10 anos (N=585)	102	**17,44**	95	**16,24**	99	16, 92	112	19,15	177	30,26
TESTE QUI-QUADRADO: X2=47,67; GL=4; P<0,001												

Fonte: Dados de pesquisa
Nota: GL: Grau de liberdade; P: valor-p

A comparação entre instituições hospitalares e não-hospitalares (outros serviços) sugeriu, que os serviços não-hospitalares apresentaram melhor prática referida para os domínios preparo do ambiente e cuidados após administração medicamentosa, enquanto os serviços hospitalares apresentaram melhor prática para o domínio administração medicamentosa e escore total do QBMI, conforme tabela 17.

Tabela 17 - Análise descritiva de escore de domínios e do instrumento entre serviços hospitalares e não hospitalares. São Carlos, SP, 2018.

Tipo de serviço	Domínio	N	Média	D.P.	Mínimo	Q1	Mediana	Q3	Máximo	Valor-p*
Instituição hospitalar	Preparo do Ambiente	744	7,54	±2,57	5,00	5,00	7,00	9,00	19,00	
	Preparo das Medicações	744	14,85	±4,55	9,00	12,00	14,00	18,00	34,00	
	Administração Medicamentosa	744	42,10	±8,72	24,00	36,00	41,00	47,00	77,00	
	Cuidados após administração medicamentosa	744	14,46	±4,06	7,00	11,00	14,50	17,00	27,00	
	Escore total	744	78,95	±15,38	49,00	68,00	77,00	88,00	139,00	
Outros Serviços	Preparo do Ambiente	551	7,14	±2,52	5,00	5,00	6,00	9,00	21,00	<0,001
	Preparo das Medicações	551	15,13	±4,55	9,00	12,00	15,00	18,00	36,00	0,195
	Administração Medicamentosa	551	45,04	±9,10	28,00	38,00	44,00	51,00	72,00	<0,001
	Cuidados após administração medicamentosa	551	13,62	±3,90	7,00	11,00	13,00	16,00	26,00	<0,001
	Escore total	551	80,93	±15,16	52,00	69,00	80,00	90,00	134,00	0,011

Fonte: Dados da pesquisa
Nota: DP: Desvio-Padrão; Q1: Primeiro Quartil; Q3: Terceiro Quartil; Valor-P referente ao teste de Mann-Whitney para comparação dos valores entre os 2 grupos

Sobre as diferenças significativas entre a frequência referida pelas instituições hospitalares e não hospitalares, sobre o preparo do ambiente, estas foram relacionadas a verificação da data de validade de insumos antes do uso e utilização de insumos com embalagens danificadas, conforme tabela 18, em destaque.

Tabela 18 - Diferença significativa entre instituições hospitalares e outros serviços de acordo com componentes do instrumento de coleta de dados sobre preparo do ambiente para administração de medicações injetáveis segundo frequência referida. São Carlos, SP, 2018.

**Código**	**Prática**	**Grau de Instrução**	**Sempre**		**Quase Sempre**		**Às Vezes**		**Quase Nunca**		**Nunca**	
			**n**	**%**	**n**	**%**	**n**	**n**	**%**	**n**	**%**	**n**
DA3	Verificação da data de validade de insumos antes do uso	Instituições Hospitalares (N=744)	378	50,81	148	19,89	91	**12,23**	78	**10,48**	49	**6,59**
		Outros serviços (N=551)	369	**66,97**	107	19,42	45	8,17	23	4,17	7	1,27
TESTE QUI-QUADRADO: X2=56,19; GL=4; P<0,001												
DA4	Utilização de insumos com embalagens danificadas	Instituições Hospitalares (N=744)	46	6,18	6	0,81	15	2,02	56	**7,53**	621	**83,47**
		Outros serviços (N=551)	52	**9,44**	10	1,81	15	2,72	28	5,08	446	80,94
TESTE QUI-QUADRADO: X2=10,88; GL=4; P=0,028												

Fonte: Dados da pesquisa

Nota: GL: Grau de liberdade; GL: Grau de liberdade; P: valor-p; Valor-P referente ao teste de Mann-Whitney para comparação dos valores entre os 2 grupos

Quanto ao domínio preparo das medicações, as diferenças relacionaram-se com a identificação de frascos multidoses e salinização de CVP, conforme tabela 19, em destaque.

Tabela 19 - Diferença significativa entre instituições hospitalares e outros serviços de acordo com componentes do instrumento de coleta de dados sobre preparo de medicações para administração de injetáveis segundo frequência referida. São Carlos, SP, 2018.

**Código**	**Prática**	**Grau de Instrução**	**Sempre**		**Quase Sempre**		**Às Vezes**		**Quase Nunca**		**Nunca**	
			**n**	**%**	**n**	**%**	**n**	**n**	**%**	**n**	**%**	**n**
DB5	Identificação de frascos multidoses (data, volume do diluente, nome do profissional)	Instituições Hospitalares (N=744)	551	74,06	73	9,81	32	4,30	30	**4,03**	58	7,80
		Outros serviços (N=551)	418	75,86	44	7,99	25	4,54	8	1,45	56	**10,16**
TESTE QUI-QUADRADO: X2=10,55; GL=4; P=0,032												
DB9	Salinização de cateteres venosos periféricos	Instituições Hospitalares (N=744)	564	**75,81**	101	13,58	59	7,93	12	1,61	8	1,08
		Outros serviços (N=551)	329	59,71	69	12,52	40	7,26	32	5,81	81	**14,70**
TESTE QUI-QUADRADO: X2=114,25; GL=4; P<0,001												

Fonte: Dados da pesquisa

Nota: GL: Grau de liberdade; GL: Grau de liberdade; P: valor-p; Valor-P referente ao teste de Mann-Whitney para comparação dos valores entre os 2 grupos

Houve diferença significativa entre os serviços hospitalares e outros serviços no domínio sobre administração medicamentosa para HM com água e sabão ou solução alcoólica/solução padronizada antes da administração medicamentosa, armazenamento inapropriado de oclusores de CVP para reutilização, desobstrução forçada de CVP, desinfecção de oclusores de dispositivos intravenosos com álcool a 70% previamente a administração medicamentosa, antissepsia da pele do paciente com álcool a 70% para administração de medicações SC, quebra da técnica *no touch* para administração de injetáveis, utilização da mesma seringa, com troca da agulha, para administração de injetável no mesmo paciente, infusão com solução fisiológica 0,9% de dispositivo intravenoso após administração medicamentosa, treinamento para manipulação de agulhas e cateteres com dispositivos de segurança, administração de medicações com lesões de pele nas mãos, uso de luvas para administração de medicações em diferentes situações, curativo estéril para fixação/manutenção dos CVP e troca deste antes do aparecimento de sinais de flebite. Tais dados são destacados na tabela 20.

Tabela 20 - Diferença significativa entre instituições hospitalares e outros serviços de acordo com componentes do instrumento de coleta de dados sobre administração de injetáveis segundo frequência referida. São Carlos, SP, 2018.

(continua)

Código	Prática	Grau de Instrução	Sempre		Quase Sempre		Às Vezes		Quase Nunca		Nunca	
			n	%	n	%	n	n	%	n	%	n
DC1	Higienização das mãos com água e sabão antes da administração de medicação endovenosa	Instituições Hospitalares (N=744)	476	63,98	159	**21,37**	69	9,27	16	2,15	24	3,23
		Outros serviços (N=551)	447	**81,13**	57	10,34	23	4,17	8	1,45	16	2,90
TESTE QUI-QUADRADO: X2=48,66; GL=4; P<0,001												
DC2	Higienização das mãos com solução alcoólica/solução padronizada antes da administração de medicação endovenosa	Instituições Hospitalares (N=744)	511	**68,68**	142	**19,09**	60	8,06	19	2,55	12	1,61
		Outros serviços (N=551)	343	62,25	77	13,97	78	**14,16**	24	4,36	29	5,26
TESTE QUI-QUADRADO: X2=34,32; GL=4; P<0,001												
DC5	Armazenamento inapropriado de oclusores de cateteres periféricos para reutilização	Instituições Hospitalares (N=744)	17	2,28	47	6,32	82	11,02	86	**11,56**	512	68,82
		Outros serviços (N=551)	13	2,36	24	4,36	54	9,80	42	7,62	418	**75,86**
TESTE QUI-QUADRADO: X2=9,83; GL=4; P=0,043												

Tabela 20 - Diferença significativa entre instituições hospitalares e outros serviços de acordo com componentes do instrumento de coleta de dados sobre administração de injetáveis segundo frequência referida. São Carlos, SP, 2018.

(continuação)

Código	Prática	Grau de Instrução	Sempre		Quase Sempre		Às Vezes		Quase Nunca		Nunca	
			**n**	**%**	**n**	**%**	**n**	**n**	**%**	**n**	**%**	**n**
DC6	Desobstrução forçada com solução fisiológica 0,9% de cateteres venosos periféricos	Instituições Hospitalares (N=744)	67	9,01	95	12,77	216	**29,03**	138	18,55	228	30,65
		Outros serviços (N=551)	50	9,07	50	9,07	116	21,05	91	16,52	244	**44,28**
TESTE QUI-QUADRADO: X2=28,62; GL=4; P<0,001												
DC8	Desinfecção de oclusores de dispositivos intravenosos com álcool a 70% previamente a administração medicamentosa	Instituições Hospitalares (N=744)	473	**63,58**	116	15,59	68	9,14	38	5,11	49	6,59
		Outros serviços (N=551)	275	49,91	63	11,43	51	9,26	36	6,53	126	**22,87**
TESTE QUI-QUADRADO: X2=77,42; GL=4; P<0,001												
DC10	Antissepsia da pele do paciente com álcool a 70% para administração de medicações subcutâneas	Instituições Hospitalares (N=744)	690	**92,74**	22	2,96	13	1,75	5	0,67	14	1,88
		Outros serviços (N=551)	487	88,38	20	3,63	9	1,63	7	1,27	28	**5,08**
TESTE QUI-QUADRADO: X2=12,34; GL=4; P=0,015												
DC12	Quebra da técnica *no touch* para administração de injetáveis	Instituições Hospitalares (N=744)	26	3,49	23	3,09	72	9,68	143	**19,22**	480	64,52
		Outros serviços (N=551)	31	5,63	15	2,72	54	9,80	75	13,61	376	**68,24**
TESTE QUI-QUADRADO: X2=10,00; GL=4; P=0,040												

Tabela 20 - Diferença significativa entre instituições hospitalares e outros serviços de acordo com componentes do instrumento de coleta de dados sobre administração de injetáveis segundo frequência referida. São Carlos, SP, 2018.

(continuação)

**Código**	**Prática**	**Grau de Instrução**	**Sempre**		**Quase Sempre**		**Às Vezes**		**Quase Nunca**		**Nunca**	
			**n**	**%**	**n**	**%**	**n**	**n**	**%**	**n**	**%**	**n**
DC13	Utilização da mesma seringa, com troca da agulha, para administração de injetável no mesmo paciente	Instituições Hospitalares (N=744)	27	3,63	14	1,88	37	**4,97**	48	**6,45**	618	**83,06**
		Outros serviços (N=551)	43	**7,80**	15	2,72	20	3,63	25	4,54	448	81,31
TESTE QUI-QUADRADO: X2=14,68; GL=4; P=0,005												
DC15	Infusão com solução fisiológica 0,9% de dispositivo intravenoso após administração medicamentosa	Instituições Hospitalares (N=744)	540	**72,58**	105	14,11	60	8,06	21	2,82	18	2,42
		Outros serviços (N=551)	325	58,98	60	10,89	58	10,53	18	3,27	90	**16,33**
TESTE QUI-QUADRADO: X2=87,15; GL=4; P<0,001												
DC17	Treinamento para manipulação de agulhas e cateteres com dispositivos de segurança	Instituições Hospitalares (N=744)	119	**15,99**	114	**15,32**	218	29,30	188	25,27	105	14,11
		Outros serviços (N=551)	49	8,89	46	8,35	135	24,50	163	29,58	158	**28,68**
TESTE QUI-QUADRADO: X2=62,67; GL=4; P<0,001												
DC18	Administração de medicações com lesões de pele nas mãos	Instituições Hospitalares (N=744)	18	2,42	27	3,63	95	12,77	177	**23,79**	427	57,39
		Outros serviços (N=551)	22	3,99	16	2,90	83	15,06	97	17,60	333	**60,44**
TESTE QUI-QUADRADO: X2=10,48; GL=4; P=0,033												

Tabela 20 - Diferença significativa entre instituições hospitalares e outros serviços de acordo com componentes do instrumento de coleta de dados sobre administração de injetáveis segundo frequência referida. São Carlos, SP, 2018.

(continuação)

Código	Prática	Grau de Instrução	Sempre		Quase Sempre		Às Vezes		Quase Nunca		Nunca	
			n	%	n	%	n	n	%	n	%	n
DC19	Uso de luvas para administração de medicações intramusculares	Instituições Hospitalares (N=744)	510	**68,55**	61	8,20	69	9,27	50	6,72	54	7,26
		Outros serviços (N=551)	337	61,16	36	6,53	52	9,44	66	**11,98**	60	**10,89**
TESTE QUI-QUADRADO: X2=18,33; GL=4; P=0,001												
DC20	Uso de luvas para administração de medicações subcutâneas	Instituições Hospitalares (N=744)	465	**62,50**	64	8,60	72	9,68	70	9,41	73	9,81
		Outros serviços (N=551)	305	55,35	37	6,72	41	7,44	80	**14,52**	88	**15,97**
TESTE QUI-QUADRADO: X2=22,78; GL=4; P<0,001												
DC21	Uso de luvas para administração de medicações endovenosas	Instituições Hospitalares (N=744)	565	75,94	73	**9,81**	42	5,65	37	**4,97**	27	3,63
		Outros serviços (N=551)	477	**86,57**	31	5,63	22	3,99	10	1,81	11	2,00
TESTE QUI-QUADRADO: X2=24,68; GL=4; P<0,001												
DC23	Curativo estéril para fixação/manutenção dos cateteres venosos periféricos	Instituições Hospitalares (N=744)	382	**51,34**	94	12,63	80	10,75	84	11,29	104	13,98
		Outros serviços (N=551)	260	47,19	54	9,80	56	10,16	60	10,89	121	**21,96**
TESTE QUI-QUADRADO: X2=15,09; GL=4; P=0,005												

Tabela 20 - Diferença significativa entre instituições hospitalares e outros serviços de acordo com componentes do instrumento de coleta de dados sobre administração de injetáveis segundo frequência referida. São Carlos, SP, 2018.

(conclusão)

**Código**	**Prática**	**Grau de Instrução**	**Sempre**		**Quase Sempre**		**Às Vezes**		**Quase Nunca**		**Nunca**	
			**n**	**%**	**n**	**%**	**n**	**n**	**%**	**n**	**%**	**n**
DC24	Troca de acesso venoso periférico, conforme padronização do serviço, antes do aparecimento de sinais de flebite	Instituições Hospitalares (N=744)	559	**75,13**	123	**16,53**	40	5,38	15	2,02	7	0,94
		Outros serviços (N=551)	379	68,78	56	10,16	29	5,26	20	3,63	67	**12,16**
TESTE QUI-QUADRADO: X2=83,83; GL=4; P<0,001												

Fonte: Dados da pesquisa

Nota: GL: Grau de liberdade; GL: Grau de liberdade; P: valor-p; Valor-P referente ao teste de Mann-Whitney para comparação dos valores entre os 2 grupos

No Domínio sobre cuidados após administração medicamentosa, a diferença significativa entre instituições hospitalares e não-hospitalares ocorreu para o reencape de agulha após administração medicamentosa, separação entre agulha e corpo da seringa para o descarte após medicar pacientes, ciência sobre situação vacinal contra doenças potencialmente passíveis de transmissão ocupacional e sobre fluxo de atendimento pós exposição percutânea no local de trabalho.

Estas diferenças podem ser observadas na tabela 21, em destaque.

Tabela 21 - Diferença significativa entre instituições hospitalares e outros serviços de acordo com componentes do instrumento de coleta de dados sobre cuidados após administração medicamentosa segundo frequência referida. São Carlos, SP, 2018.

(continua)

Código	Prática	Grau de Instrução	Sempre		Quase Sempre		Às Vezes		Quase Nunca		Nunca	
			**n**	**%**	**n**	**%**	**n**	**n**	**%**	**n**	**%**	**n**
DD1	Reencape de agulha após administração medicamentosa	Instituições Hospitalares (N=744)	30	4,03	35	4,70	78	**10,48**	91	**12,23**	510	68,55
		Outros serviços (N=551)	33	5,99	24	4,36	39	7,08	42	7,62	413	**74,95**
TESTE QUI-QUADRADO: X2=15,01; GL=4; P=0,005												
DD4	Separação entre agulha e corpo da seringa para o descarte após medicar pacientes	Instituições Hospitalares (N=744)	134	**18,01**	68	**9,14**	81	10,89	86	11,56	375	50,40
		Outros serviços (N=551)	69	12,52	20	3,63	42	7,62	39	7,08	381	**69,15**
TESTE QUI-QUADRADO: X2=49,41; GL=4; P<0,001												
DD5	Ciência sobre situação vacinal contra doenças potencialmente passíveis de transmissão ocupacional, como a hepatite B	Instituições Hospitalares (N=744)	436	58,60	108	14,52	105	14,11	68	**9,14**	27	3,63
		Outros serviços (N=551)	360	**65,34**	74	13,43	66	11,98	20	3,63	31	5,63
TESTE QUI-QUADRADO: X2=20,66; GL=4; P<0,001												

Tabela 21 - Diferença significativa entre instituições hospitalares e outros serviços de acordo com componentes do instrumento de coleta de dados sobre cuidados após administração medicamentosa segundo frequência referida. São Carlos, SP, 2018.

(conclusão)

**Código**	**Prática**	**Grau de Instrução**	**Sempre**		**Quase Sempre**		**Às Vezes**		**Quase Nunca**		**Nunca**	
			**n**	**%**	**n**	**%**	**n**	**n**	**%**	**n**	**%**	**n**
DD6	Ciência sobre fluxo de atendimento pós exposição percutânea com material perfurocortante no seu local de trabalho	Instituições Hospitalares (N=744)	309	41,53	111	14,92	153	**20,56**	102	13,71	69	9,27
		Outros serviços (N=551)	257	**46,64**	85	15,43	84	15,25	62	11,25	63	11,43

TESTE QUI-QUADRADO: X2=9,80; GL=4; P=0,044

Fonte: Dados da pesquisa

Nota: GL: Grau de liberdade; GL: Grau de liberdade; P: valor-p; Valor-P referente ao teste de Mann-Whitney para comparação dos valores entre os 2 grupos

A partir do coeficiente de relação de Spearman, quanto à idade (n=1271), obteve-se significativa correlação inversamente proporcional nos domínios "preparo do ambiente" (-0,1905, P<0,0001), "preparo das medicações" (r= -0,06158; P= 0,0282), "administração medicamentosa" (r= -0,09526; P=0,0007) e "cuidados após medicações injetáveis" (-0,11108, P<0,0001) e em todo o instrumento QBMI (r= -0,13797; P<0,0001), ou seja, quanto maior a idade, menor a pontuação obtida e, consequentemente, melhor a prática referida nestes domínios/instrumento.

Já no tocante a correlação entre tempo de atuação (n=1295) e os domínios do instrumento QBMI, houve significativa correlação inversamente proporcional para condutas referentes ao preparo do ambiente (r=-0,05639; P= 0,0425) e cuidados após administração (r= -0,05986; P= 0,0312), sendo assim, quanto maior o tempo de atuação, menor o escore nestes domínios, ou, melhor a conduta para o preparo de medicações e cuidados após administração medicamentosa.

Após a utilização do instrumento para coleta de dados, ele foi avaliado quanto à sua consistência interna (α de Cronbach) para medir a confiabilidade do QBMI, no total e nos domínios, apresentando melhor consistência interna para o total (α=0,790).

Os dados referentes a esta etapa da pesquisa foram publicados (sob financiamento da Coordenação de Aperfeiçoamento de Pessoal de Nível Superior (CAPES) na revista da Escola de Enfermagem da USP, volume 54, em 2020 (disponível em http://www.scielo.br/scielo.php?script=sci_arttext&pid=S0080-62342020000100499&lng=en&nrm=iso).

5.2 DESENVOLVIMENTO

5.2.1 **Curso "Boas Práticas com Injetáveis: ações para o controle de infecção": planejamento, elaboração, validação e revisão das ferramentas educativas**

5.2.1.1 Elaboração das ferramentas educativas

A fim de subsidiar o desenvolvimento das ferramentas educativas e anteriormente a criação do texto-base (APÊNDICE I) foi elaborado um esquema contando com os componentes que deveriam emergir no curso, conforme análise contextual:

Figura 2 – Esquema sobre os componentes das Boas Práticas com Injetáveis a serem abordados no material educativo proposto. São Carlos, SP, 2020.

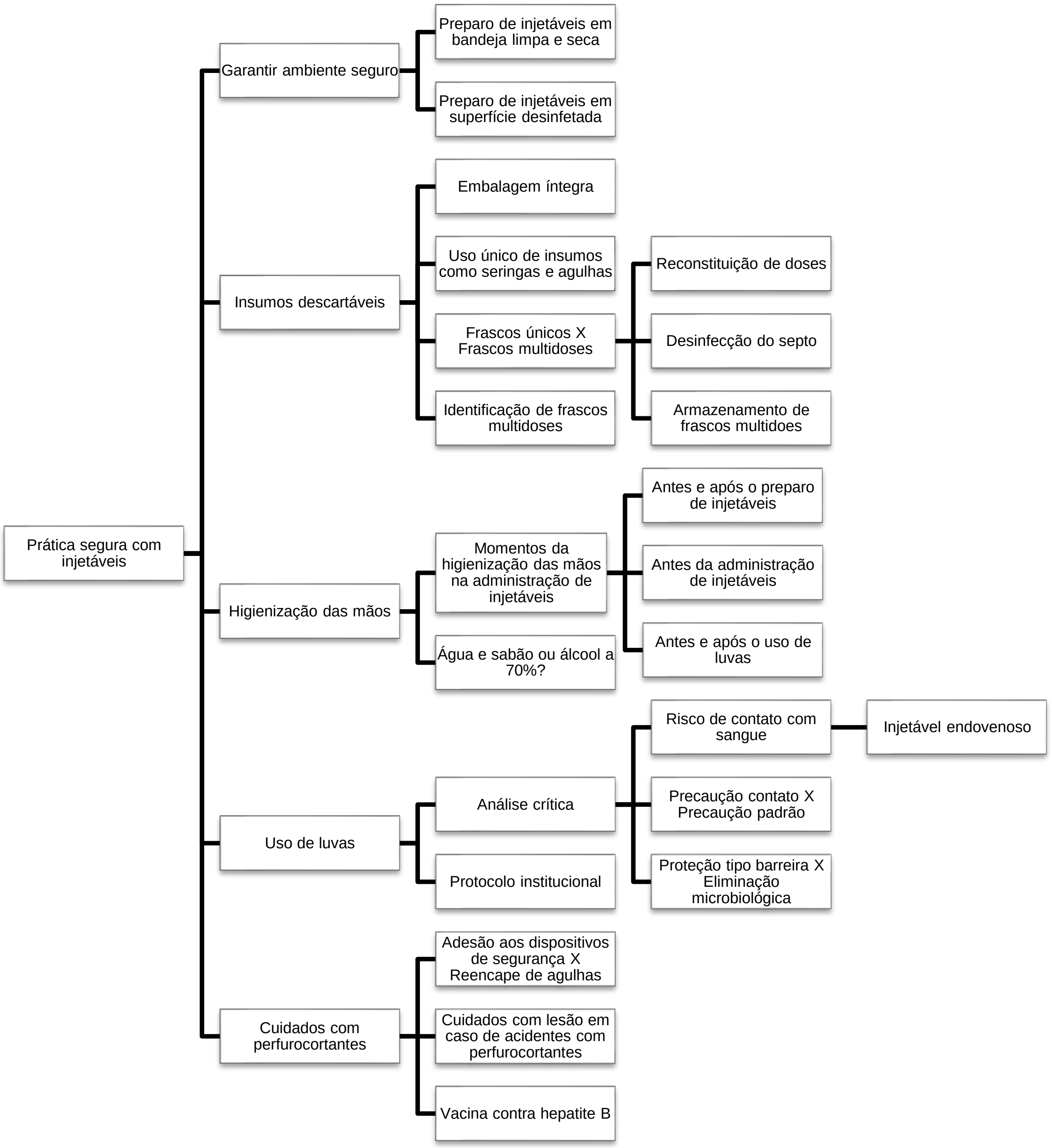

Fonte: Elaborado pela autora

Tendo-se em vista as orientações fornecidas pelo PoCA, a TAS e orientações de Filatro e Cairo (2015), objetivou-se dividir o conteúdo em duas ferramentas, um *ebook* e um *podcast*, conforme tratado a seguir.

5.2.1.1 A construção do ebook “Boas Práticas com Injetáveis: Ações para o Controle de Infecção”

Em sua primeira versão, o ebook subdividia o título em dois subtítulos: “A escolha dos insumos utilizados: qual a influência no controle de infecção?” e “Higienização das mãos: elemento de destaque da prática com injetáveis!”, já no modelo padronizado pelo PoCA (figura 3), em dez páginas, em PDF.

Figura 3 - Capa e primeira página do ebook “Boas Práticas com Injetáveis: ações para o controle de infecção: elaboração e validação por conteúdo”. São Carlos, SP, 2020.

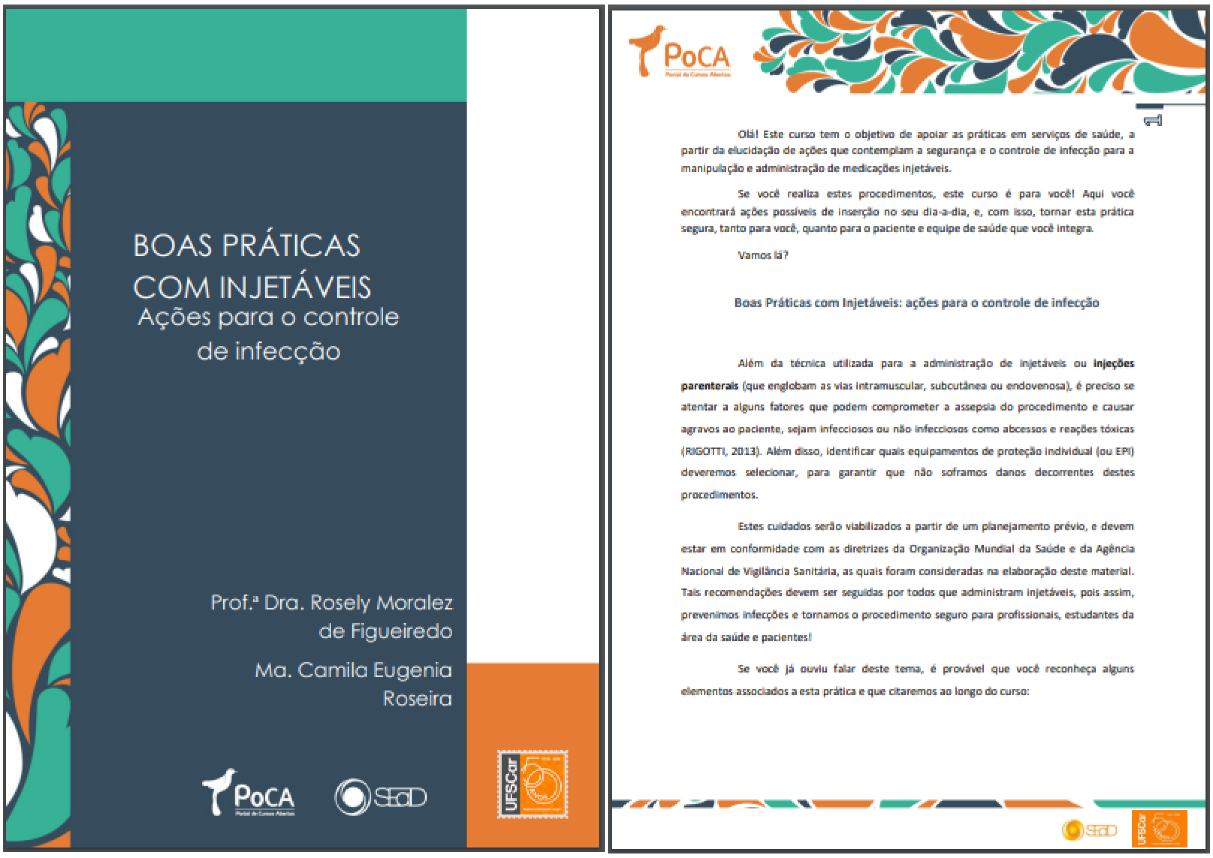

Fonte: Elaborado pela autora

Na primeira página, havia a apresentação do material textual, convidando o participante para explorar o mesmo, e após o título, foi discorrido sobre os fatores associados a quebra da barreira asséptica relacionada a administração de injetáveis e os agravos decorrentes desta.

Sequencialmente, tratou-se dos elementos que constituem a administração de medicações injetáveis, acompanhadas de imagens e denominações usuais, a saber:

cateteres intravenosos agulhado e flexível, dispositivo de segurança acoplado em agulha hipodérmica, dânula e dupla-via.

Na página três, em “A escolha dos insumos utilizados: qual a influência no controle de infecção?” foi tratado o controle do ambiente para o manuseio seguro de insumos utilizados na administração de medicações injetáveis, a importância de não reutilizar insumos de uso único, desinfecção e cuidados de armazenamento, frascos para reconstituição de dose única/multidose, e imagens diferenciando a ampola e o frasco de diluente, frasco-ampola e ampola de solução medicamentosa e oclusor tipo *luer lock*.

Na página seis, foi dedicada uma seção para a HM contemplando as etapas para administração de injetáveis, uso de água e sabão ou álcool a 70% e uso de luvas (análise crítica para a proteção e observação desta enquanto recurso complementar à higienização das mãos).

As imagens foram adquiridas a partir do acervo de fotografias iStock (https://www.istockphoto.com/br), com conteúdo Royalty-Free em que, para o presente trabalho, optou-se pela licença padrão, ou uso de fotografias realizadas e editadas pela autora.

As referências utilizadas para a elaboração do conteúdo (BRASIL, 2017; FERRONI *et al*., 2014; GRAZIANO *et al*., 2012; RIGOTTI, 2012; ORGANIZAÇÃO MUNDIAL DA SAÚDE, 2010) ocuparam as páginas sete e oito, enquanto a página nove finalizou com um resumo constando o objetivo do curso.

5.2.1.2 A construção do podcast “Boas Práticas com Medicações Injetáveis: Subsídios para o Autocuidado”

O processo de gravação e edição da primeira versão do *podcast*, também, foi realizado pela pesquisadora.

O *podcast*, em sua primeira versão, possuía 4’52’’ e foi gravado a partir do aplicativo para Android Gravador de Som & Voz – ASR, em formato MP3, com auxílio de fone de ouvido.

Para a gravação, o texto roteirizado foi ensaiado a fim de se adequar a fala, visando clareza e coesão na comunicação oral, cronometrando o tempo, antes da

apresentação definitiva, fazendo-se as edições necessárias pelo *Audacity*, *software* livre de edição digital de áudio, e seu conteúdo foi transcrito, posteriormente.

Para o áudio, foi adquirido uma música, também sob licença Creative Commons, a partir do domínio https://filmmusic.io/ (Funny, por MusicLFiles).

Esta primeira versão do *podcast* incluía a apresentação da pesquisadora e um convite inicial à reflexão sobre a avaliação crítica na utilização de luvas de procedimento ao administrar injetáveis.

Além disto, o conteúdo do podcast, também, versou sobre a importância da integridade cutânea das mãos dos profissionais para administrar injetáveis e como agir caso estiver com a mesma não-íntegra.

A prevenção de acidentes com perfurocortantes também foi abordada, bem como quais os cuidados iniciais em caso deste tipo de evento.

A transcrição do áudio (constando em quatro páginas) constava das citações e referências utilizadas durante a construção do mesmo (BRASIL, 2017; CONSELHO REGIONAL DE ENFERMAGEM DE SÃO PAULO, 2010; GRAZIANO *et al*., 2012; MONCAIO; FIGUEIREDO, 2009; O'GRADY *et al*., 2011; SÃO PAULO, 2014; ORGANIZAÇÃO MUNDIAL DA SAÚDE, 2009, 2010).

5.2.1.3 Questões avaliativas do conteúdo do curso e questões para interação interparticipantes no fórum

Para a avaliação do conteúdo abordado no curso, foram elaboradas cinco questões obrigatórias com respostas do tipo múltipla-escolha, conforme o modelo estrutural abaixo:

Figura 4 - Modelo estrutural das questões obrigatórias para conclusão do curso "Boas Práticas com Injetáveis: ações para o controle de infecção". São Carlos, SP, 2020.

Número da questão a ser avaliada
Serão 5, no total

1. Segundo a prescrição médica, a técnica de enfermagem Ana, deverá administrar uma ampola de dipirona monossódica (500mg/ml) diluída em 20 ml de solução fisiológica 0,9% (SF 0,9%), via endovenosa. Para a diluição da medicação, há ampolas contendo 10 ml e frascos de 100 e 250 ml de SF 0,9%. Pensando no controle de infecção é correto que Ana:

Cada alternativa é precedida por uma letra. Serão cinco alternativas para cada questão

a) Selecione o frasco de 100 ml para aspirar o total de diluente (20 ml de SF 0,9%) e armazene o restante do frasco para diluições futuras (para o mesmo paciente).

Conforme sugestão da plataforma que contemplará o curso, os feedbacks formativos devem ter "comentários para questões corretas e incorretas, numa linguagem dialogada com os cursistas "

Feedback: Poxa, vamos tentar de novo? Utilizar frascos com grandes quantidades para diluir medicações in bolus pode predispor este volume restante às contaminações, e levar danos aos pacientes que receberem as doses subsequentes

b) Selecione 2 ampolas de SF 0,9% para diluir a dipirona, descartando os resíduos em seguida.

Feedback: Muito bem!

c) Compartilhe o conteúdo de algum frasco com grande volume de SF 0,9% para fazer a diluição da medicação deste paciente e utilizar o restante para diluir medicações de outros pacientes.

Feedback: Poxa, vamos tentar de novo? Utilizar frascos com grandes quantidades para diluir medicações in bolus pode predispor este volume restante às contaminações, e levar danos aos pacientes que receberem as doses subsequentes.

d) Diluir em um frasco de 100 ml de SF 0,9%, uma vez que não há dose unitária com o volume de diluente prescrito.

Feedback: Poxa, vamos tentar de novo? Utilizar frascos com grandes quantidades para diluir medicações in bolus pode predispor este volume restante às contaminações, e levar danos aos pacientes que receberem as doses subsequentes

e) Diluir com uma ampola de SF 0,9% (10 ml), pois, é o volume unitário disponível.

Feedback: Poxa, vamos tentar de novo? Esta opção não contempla a prescrição médica.

Comentário geral, contemplando a justificativa e referência sobre a alternativa correta

Comentário: está correta a alternativa b. Para garantir a diluição conforme prescrição médica (com 20 ml de SF 0,9%) e prevenir contaminação da solução, e, consequentemente, de todas as doses a partir desta ação, podendo ocasionar danos ao paciente (BRASIL, 2017)

Fonte: Elaborado pela autora

As questões foram elaboradas a partir do referencial teórico utilizado para a apresentação do conteúdo do *ebook* e *podcast* (BRASIL, 2017; CONSELHO REGIONAL DE ENFERMAGEM DE SÃO PAULO, 2010; FERRONI *et al.*, 2014; MONCAIO; FIGUEIREDO, 2009; O'GRADY *et al.*, 2011; ORGANIZAÇÃO MUNDIAL DA SAÚDE, 2010, 2014).

A primeira questão abordou a seleção de apresentação de diluente (ampola/frasco) para administração de medicações via EV a partir do controle de infecção frente ao procedimento.

Na segunda questão, o participante foi convidado a refletir sobre três premissas: a quem a HM protege quando o profissional/estudante higieniza as mãos antes da inserção do cateter intravenoso; o uso de luvas enquanto complemento à HM ao realizar

o procedimento; a quem a HM protege quando o profissional/estudante higieniza as mãos após manipulação do cateter intravenoso.

Na terceira questão, o participante foi convidado a preencher seis espaços em branco (com as palavras “nidação”, “funcionalidade”, “proibido”, “estéreis”, “*flushing*” e “baixa”) em um período que relacionava a realização de *flushing* a partir de uma baixa pressão com a manutenção do cateter intravenoso e prevenção da colonização do mesmo por microrganismos, utilizando-se seringas com esterilidade preservada e reiterando a proibição do reuso de dispositivos descartáveis mesmo para o próprio paciente.

A ocorrência de acidentes com perfurocortante foi abordada na quarta questão avaliativa, em que o participante foi convidado a refletir sobre as premissas: análise crítica para utilização de luvas para realização de injeção IM, relação entre risco de infecção e esquema vacinal do profissional (hepatite B), notificação do evento ao serviço e importância deste para redução de risco a saúde do profissional.

A quinta questão tratou de uma assertiva cujo participante deveria julgar se verdadeira ou falsa sobre a utilização de álcool a 70% enquanto solução desinfetante e sua utilização em lesão por perfurocortante.

Ressalta-se que a cada alternativa selecionada pelo participante, o comentário de “*feedback*” o parabenizava com uma mensagem de “Muito Bem!” enquanto o comentário que acusava a alternativa como errada continha uma mensagem corrigindo a informação equivocada, iniciada por “Poxa, vamos tentar de novo?”.

Já as duas questões disparadoras de discussão (abertas) para o fórum, não tinham caráter avaliativo ou tutoria para moderação, para as quais, buscou-se incentivar o compartilhamento de experiências a partir de vivências em serviços de saúde com acidente com perfurocortante e treinamentos para manuseio de injetáveis com perfurocortantes a partir do olhar dos participantes do curso.

5.2.1.2 Validação por conteúdo das ferramentas educativas

Para a validação por conteúdo, 18 sujeitos foram convidados via eletrônica no dia primeiro de setembro de 2020 para participarem desta etapa da pesquisa, via e-mail, o qual continha, a linhas gerais, em que o material educativo consistia e como seria disponibilizado (via portal aberto, sem tutoria), solicitando confirmação da disponibilidade para envio do material.

No total, treze sujeitos responderam positivamente ao convite. Em oito de setembro do mesmo ano, foram encaminhados, via e-mail: formulário para preenchimento (com as orientações instrucionais e já contemplando as questões avaliativas do conteúdo e para o fórum) acessado ao clicar em *link* (APÊNDICE J) e o material educativo em sua primeira versão (*ebook*, *podcast* e sua transcrição), como anexo.

Ao findar o prazo para validação (em 29/09), dez avaliadoras retornaram o instrumento de validação preenchido.

5.2.1.2.1 Caracterização das avaliadoras

Todas as avaliadoras eram do sexo feminino, com idade variando entre 24 e 57 anos (36,1±9,7), 40% (4/10) possuíam mestrado, 20% (2/10) doutorado, 30% (3/10) especialização e 10% (1/10) alunas de graduação (a qual realizava iniciação científica na área).

Sobre a atuação destas, 30% (3/10) atuavam como enfermeiras assistenciais, 20% (2/10) como enfermeira do Serviço de Controle de Infecção Hospitalar (SCIH), 20% (2/10) docentes universitárias, 10% (1/10) como docente de curso técnico de enfermagem, 10% (1/10) doutoranda em Ciências da Saúde e 10% (1/10) como aluna de graduação de enfermagem.

5.2.1.2.2 Processo de validação do ebook “Boas Práticas com Injetáveis: Ações para o Controle de Infecção

O *ebook* “Boas Práticas com Injetáveis: ações para o controle de infecção” deveria apresentar fatores associados ao ambiente seguro e o cuidado com os insumos para preparo de injetáveis, como a manutenção de limpeza de superfície de preparo; insumos -uso único, integridade da embalagem- desinfecção e cuidados de armazenamento de frascos para reconstituição de dose única/multidose, momentos para a HM de acordo com as etapas para administração de injetáveis, uso de água e sabão ou álcool a 70%; uso de luvas (análise crítica para a proteção).

De forma geral, o *ebook* foi avaliado como adequado por todas as avaliadoras, considerando-se seus objetivos, estrutura e relevância, conforme tabela a seguir:

Tabela 22 - Análise descritiva dos escores do ebook “Boas Práticas com Injetáveis: ações para o controle de infecção” aos objetivos, segundo o Instrumento de Validação de Conteúdo Educativo em Saúde. São Carlos, SP, 2020.

**Domínio**	**Máximo**	**Mínimo**	**Média(±dp)**	**Mediana**	**Moda**
Objetivo	10	8	9,7(±0,68)	10	10
Estrutura/Apresentação	20	18	19,6(±0,84)	20	20
Relevância	6	4	5,8(±0,63)	6	6
**Total**	36	30	35,1(±2,02)	36	36

Fonte: Dados da pesquisa

As tabelas subsequentes apresentam a frequência relativa de concordância por gradação de adequabilidade, segundo cada item por domínios do IVCES.

Tabela 23 - Frequência relativa sobre concordância das avaliadoras sobre a adequabilidade do ebook “Boas Práticas com Injetáveis: ações para o controle de infecção” aos objetivos, segundo o Instrumento de Validação de Conteúdo Educativo em Saúde. São Carlos, SP, 2020.

**Item**	**Adequado (%)**	**Parcialmente Adequado (%)**	**Inadequado (%)**
Contempla tema proposto	100	0	0
Adequado ao processo de ensino-aprendizagem	100	0	0
Esclarece dúvidas sobre o tema abordado	100	0	0
Proporciona reflexão sobre o tema	80	20	0
Incentiva mudança de comportamento	90	10	0

Fonte: Dados da pesquisa

No quadro a seguir, pode-se observar as adequações realizadas no *ebook*, a fim de contemplar às sugestões realizadas pelas avaliadoras:

Quadro 4 - Adequações realizadas no *ebook* "Boas Práticas com Injetáveis: ações para o controle de infecção" para atender às sugestões das avaliadoras sobre a adequação aos objetivos da ferramenta educativa. São Carlos, SP, 2020.

**Sugestões realizadas pelas avaliadoras**	**Adequações realizadas no ebook "Boas Práticas com Injetáveis: ações para o controle de infecção: elaboração e validação por conteúdo"**
"Citar mais exemplos, facilita a reflexão sobre a execução da prática" **(E5)**	"Talvez você tenha pensado em uma manipulação insegura dos insumos (por exemplo, manipular seringas e agulhas sem higienizar as mãos ou utilização de solução injetável previamente contaminada que não foi descartada oportunamente) uma antissepsia ineficaz previamente à administração de uma medicação intramuscular, ou ainda, a não identificação prévia de sinais locais que sugiram flebite em uma punção venosa já instalada" (p.1)<br>"Equipos e dispositivos complementares devem ser trocados a cada troca de cateter venoso periférico, caso não haja restrição de acordo com a solução administrada (por exemplo, para administração de hemocomponentes, a troca destes deve ser a cada bolsa), quebra de integridade do produto ou suspeita de contaminação deste ou do sistema, o que enseja troca imediata (BRASIL, 2017) "<br>"Você sabia que a manutenção de uma assistência segura se inicia com o planejamento de nossas ações? Por exemplo, ao selecionarmos os insumos que utilizaremos durante uma administração de medicação injetável, poderemos inserir ações das Boas Práticas em nosso dia a dia" (p.5)
"Sugiro incluir uma imagem de *luer slip* (página 5); avaliar a possibilidade de substituir a palavra "nidificação" (página 4)" **(E7)**	Incluída uma imagem de conexão do tipo *luer slip* ao lado da imagem de exemplo de conexão *luer lock* (p.9)<br><br>Substituída a palavra "nidificação" por colonização (p.7)

Fonte: Dados da pesquisa

Já sobre a adequação desta ferramenta educativa enquanto estrutura, a concordância das juízas está na tabela 24.

Tabela 24 - Frequência relativa sobre concordância das avaliadoras sobre a adequabilidade do ebook "Boas Práticas com Injetáveis: ações para o controle de infecção" a estrutura, segundo o Instrumento de Validação de Conteúdo Educativo em Saúde. São Carlos, SP, 2020.

**Item**	**Adequado (%)**	**Parcialmente Adequado (%)**	**Inadequado (%)**
Linguagem adequada ao público-alvo	100	0	0
Linguagem apropriada ao material educativo	100	0	0
Linguagem interativa, permitindo envolvimento ativo no processo educativo	80	20	0
Informações corretas	100	0	0
Informações objetivas	100	0	0
Informações esclarecedoras	100	0	0
Informações necessárias	90	10	0
Sequência lógica das ideias	90	10	0
Tema atual	100	0	0
Tamanho do texto adequado	100	0	0

Fonte: Dados da pesquisa

Foram adequações realizadas a fim de contemplar as sugestões das avaliadoras:

Quadro 5 - Adequações realizadas no *ebook* "Boas Práticas com Injetáveis: ações para o controle de infecção: elaboração e validação por conteúdo" para atender às sugestões das avaliadoras sobre a adequação a estrutura da ferramenta educativa. São Carlos, SP, 2020.

(continua)

**Sugestões realizadas pelas avaliadoras**	**Adequações realizadas no ebook "Boas Práticas com Injetáveis: ações para o controle de infecção: elaboração e validação por conteúdo"**
"Material muito bem-feito, parabéns! Acredito que para fins estéticos mesmo, seria interessante separar as informações que estão logo após a capa em uma página separada do início do material. Outro ponto que fiquei pensando, quando é falado sobre os materiais necessários para administração de injetáveis, validade, embalagem, uso único...fala-se sobre a flebite. Não seria importante acrescentar algo falando de que também é importante analisar o sítio da punção? Às vezes, o material está com validade adequada, limpo, íntegro, escolhido e preparado de acordo com as boas práticas, porém, punciona-se um local em que já havia sinais flogísticos (...)" (E4)	Como havia um limite de páginas, não foi possível realizar a modificação do local da apresentação do material educativo de seu conteúdo informativo<br><br>Foi colocado em uma caixa informativa, na página 1:<br>"Por falar em flebite, você sabe o que é e quais os seus sinais?<br>A flebite é uma irritação que ocorre na parede da veia, que pode ser mecânica (ocasionada por cateteres venosos de calibres maiores, geralmente), química (em decorrência do próprio medicamento infundido), relacionada às condições clínicas do paciente, bacteriana (quando há quebra na técnica asséptica) ou pós-infusão, quando surge após 48 de remoção do acesso venoso periférico (BRASIL, 2017, INFUSION NURSES SOCIETY, 2016).<br>São sinais/sintomas desta inflamação: dor, sensibilidade, eritema, calor, inchaço, endurecimento, purulência ou cordão venoso palpável (INFUSION NURSES SOCIETY, 2016)"
"Linguagem: informal e interativa somente em alguns trechos, sugiro padronizar pela interatividade em todos os parágrafos; sequência lógica das ideias: a higienização das mãos deve ser o primeiro tema abordado, visto sua importância e necessidade de realização antes da execução de qualquer procedimento" (E5)	A fim de padronizar interatividade, foram inseridas perguntas ao longo do ebook:<br>"Você identifica quais fatores podem comprometer a administração injetável e que possam causar estes danos ao paciente?" (p.1)<br>"Você percebe a importância das Boas Práticas na administração de injetáveis para a manutenção do dispositivo, conforto e segurança dos pacientes?" (p.5)<br>"Você sabia que a manutenção de uma assistência segura se inicia com o planejamento de nossas ações?" (p.5)<br>"Você sabe quais as implicações desta ação?" (p.6)<br>"Você sabe quais ações podem auxiliar a tornar o manuseio de frascos multidoses seguro?" (p.7)<br>A seção sobre Higienização das mãos passou a ser o segundo tópico abordado, após a introdução ao assunto, na página 2.

Quadro 4 - Adequações realizadas no *ebook* “Boas Práticas com Injetáveis: ações para o controle de infecção: elaboração e validação por conteúdo” para atender às sugestões das avaliadoras sobre a adequação a estrutura da ferramenta educativa. São Carlos, SP, 2020.

(conclusão)

**Sugestões realizadas pelas avaliadoras**	**Adequações realizadas no ebook “Boas Práticas com Injetáveis: ações para o controle de infecção: elaboração e validação por conteúdo”**
“(...) 1) pág 3: seria apropriado citar que a desinfecção de superfície/badeja pode ser realizada com álcool 70% ou desinfetante padronizado na instituição? 2) pág 5: é necessário citar que os oclusores podem ser utilizados em dânulas ou dupla via (o 2 parágrafo se refere apenas as dânulas). 3) pág 7: a figura 3 não cita a administração das demais medicações injetáveis (IM, SC), seria indicado adicionar? 4) Falta a referência completa do Van Tuong." (E.6)	Manteve-se a sugestão de limpeza de balcão e bandeja com álcool a 70% visto eficiência e disponibilidade deste, no serviço.<br><br>Inserido “dupla-via no referido parágrafo, p.8<br><br>A figura 3 tornou-se figura 1, visto referir-se aos momentos de higienização das mãos e inseriu-se as demais vias de administração (IM e SC)<br>Completou-se a referência citada.
“Avaliar a possibilidade de inserir no material algum "lembrete" sobre as questões éticas que envolvem a administração de medicamentos, exemplo COFEN 311 ou outros”. (E7)	Inserido um quadro convidando o participante a visualizar o link para a referência: CONSELHO REGIONAL DO ESTADO DE SÃO PAULO. Código de Ética e Principais Legislações para o Exercício da Enfermagem. São Paulo. 2018. Disponível em < https://portal.coren-sp.gov.br/wp-content/uploads/2018/11/Codigo-de-etica.pdf
“Tema atual, penso que talvez mais informações que impactem sobre a importância da manipulação correta dos injetáveis, números relacionados a infecção e também senti falta de informações sobre tempo de troca dos dispositivos (conectores) para prevenção de infecção” (E10)	Inserido o trecho: “Embora o risco de infecção de corrente sanguínea associada ao cateter periférico seja um evento raro, sua ocorrência já foi associada a microrganismos gram-positivos (58%), gram-negativo (35,8%), *Candida spp* (6.2%), dentre outros. Além disso, identificou-se que pacientes com hemocultura positiva vieram a óbito dentro de 30 dias do diagnóstico, os quais apresentaram proporção maior de infecção por *Staphylococcus aureus* em relação aos sobreviventes (SATO et al, 2017)”. (p.6)<br>Inseridas recomendações sobre trocas de dispositivos e oclusores para prevenção de infecção, segundo Brasil (2017) (pp. 4-5)

Fonte: Dados da pesquisa

Além disto, modificou-se figura que abordava os momentos de HM a fim de atender a sugestão da avaliadora E9, aumentando seu tamanho a fim de garantir acessibilidade.

A figura supracitada pode ser visualizada a partir da imagem, a seguir:

Figura 5 - Adequação de figura após validação de conteúdo do ebook "Boas Práticas com Injetáveis: ações para o controle de infecção" a relevância, segundo o Instrumento de Validação de Conteúdo Educativo em Saúde. São Carlos, SP, 2020.

<table>
<tr><th>Figura pré-validação</th><th>Figura pós-validação</th></tr>
<tr><td>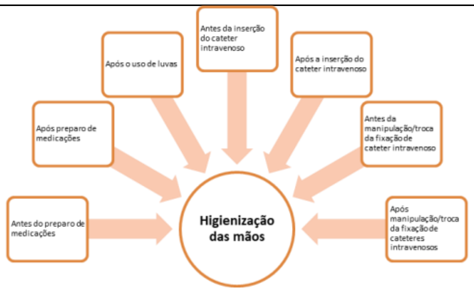
</td><td>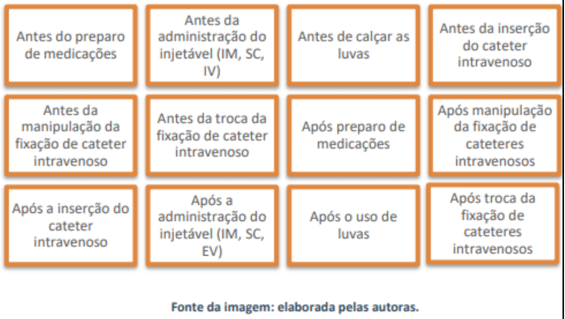
</td></tr>
</table>

Fonte: Elaborado pela autora

A concordância sobre a relevância do *ebook* encontra-se na tabela 25.

Tabela 25 - Frequência relativa sobre concordância das avaliadoras sobre a adequabilidade do ebook "Boas Práticas com Injetáveis: ações para o controle de infecção" a relevância, segundo o Instrumento de Validação de Conteúdo Educativo em Saúde. São Carlos, SP, 2020.

Item	Adequado (%)	Parcialmente Adequado (%)	Inadequado (%)
Estimula o aprendizado	90	10	0
Contribui para o conhecimento na área	100	0	0
Desperta interesse pelo tema	90	10	0

Fonte: Dados da pesquisa

Sobre a relevância, apenas uma avaliadora deixou sugestão:

> Como disse acredito que para mudança de comportamento esse material não atingiria o objetivo, sugiro colocar algo mais provocativo ao profissional que está lendo no intuito dele fazer uma reflexão sobre as práticas dele. Por exemplo uma tabela para ele preencher de *como eu faço* e depois ao final ele comparar com uma tabela de *como devo fazer*. Depois para ele escrever por exemplo três coisas que ele mudaria na sua prática após as informações recebidas (E10).

Contudo, tendo em vista o limite de páginas e por ser um curso sem tutoria, não foi possível inserir exercícios conforme os sugeridos pela avaliadora.

O ebook validado está, conforme modelo do PoCA, no Apêndice K.

5.2.1.2.3 Processo de validação do podcast "Boas Práticas com Medicações Injetáveis: subsídios para o autocuidado"

Reitera-se que o *podcast* "Boas Práticas com Medicações Injetáveis: subsídios para o autocuidado" foi a ferramenta eleita para abordar os conteúdos sobre cuidados com perfurocortantes: adesão aos dispositivos de segurança, primeiros cuidados à lesão, vacina contra hepatite B e importância do conhecimento do Protocolo Pós-Exposição.

A análise descritiva referente a cada domínio do IVCES, aplicado para a validação deste material educativo encontra-se na tabela 26, enquanto as tabelas 27, 28 e 29 apresentam a frequência relativa da concordância por gradação de adequabilidade, segundo cada item por domínio do IVCES.

Tabela 26 - Análise descritiva dos escores do podcast "Boas Práticas com Medicações Injetáveis: subsídios para o autocuidado" segundo o Instrumento de Validação de Conteúdo Educativo em Saúde. São Carlos, SP, 2020.

Domínio	Máximo	Mínimo	Média(±dp)	Mediana	Moda
Objetivo	10	8	9,8(±0,63)	10	10
Estrutura/Apresentação	20	19	19,9(±0,32)	20	20
Relevância	6	4	5,8(±0,63)	6	6
**TOTAL**	36	31	35,5(±1,6)	36	36

Fonte: Dados da pesquisa

Tabela 27 - Frequência relativa da concordância das avaliadoras sobre a adequabilidade do podcast "Boas Práticas com Medicações Injetáveis: subsídios para o autocuidado" aos objetivos, segundo o Instrumento de Validação de Conteúdo Educativo em Saúde. São Carlos, 2020.

Item	Adequado (%)	Parcialmente Adequado (%)	Inadequado (%)
Contempla tema proposto	100	0	0
Adequado ao processo de ensino-aprendizagem	100	0	0
Esclarece dúvidas sobre o tema abordado	100	0	0
Proporciona reflexão sobre o tema	90	10	0
Incentiva mudança de comportamento	90	10	0

Fonte: Dados da pesquisa

Houve apenas uma sugestão referente aos objetivos desta ferramenta educativa: "Também não sei o quanto o material é adequado para mudança de

comportamento. Talvez sugeriria mais perguntas durante o *podcast* para despertar mais reflexão" (E10).

A fim de se adequar a sugestão da avaliadora, foram inseridos os seguintes questionamentos: "E sobre a prevenção de acidentes com perfurocortantes? Você sabe quais as ações necessárias?" e "Como está sua carteira de vacinação? Completa?".

Já sobre a adequação da ferramenta educativa enquanto estrutura, a concordância das juízas está na tabela 28.

Tabela 28 - Frequência relativa sobre concordância das avaliadoras sobre a adequabilidade do podcast "Boas Práticas com Medicações Injetáveis: subsídios para o autocuidado" a estrutura, segundo o Instrumento de Validação de Conteúdo Educativo em Saúde. São Carlos, 2020.

Item	Adequado (%)	Parcialmente Adequado (%)	Inadequado (%)
Linguagem adequada ao público-alvo	100	0	0
Linguagem apropriada ao material educativo	100	0	0
Linguagem interativa, permitindo envolvimento ativo no processo educativo	90	10	0
Informações corretas	100	0	0
Informações objetivas	100	0	0
Informações esclarecedoras	100	0	0
Informações necessárias	100	0	0
Sequência lógica das ideias	100	0	0
Tema atual	100	0	0
Tamanho do texto adequado	100	0	0

Fonte: Dados da pesquisa

As adequações realizadas estão sumarizadas no quadro a seguir:

Quadro 5 - Adequações realizadas no podcast “Boas Práticas com Medicações Injetáveis: subsídios para o autocuidado” para atender às sugestões das avaliadoras sobre a adequação a estrutura da ferramenta educativa. São Carlos, SP, 2020.

Sugestões realizadas pelas avaliadoras	Adequações realizadas no podcast “Boas Práticas com Medicações Injetáveis: subsídios para o autocuidado”
“No momento em que começa a falar sobre a prevenção de acidentes com perfurocortantes acho que isso foi realizado de forma meio repentina, acredito que seja necessária uma frase para fazer uma ligação”. (E4)	Antes da gravação sobre prevenção de acidente com perfurocortantes, foi inserida uma questão para reflexão “E sobre a prevenção de acidentes com perfurocortantes? Você sabe quais as ações necessárias?”
“"Boa dicção e qualidade da voz durante a reprodução do áudio. O podcast faz a apresentação do assunto e do locutor logo no início, traz informações de forma clara e objetiva, utilizando linguagem adequada. Seria indicado falar a data?<br>Em relação ao reencape de agulhas: pode ser realizado em situações em que o descarpack não esteja disponível, nessas situações pode-se realizar o reencape passivo, ou seja, utilizar apenas uma única mão". (E6)	Não foi inserida a data pois o material ficará disponível para realização via PoCA por dois anos, apenas.<br>Sobre o reencape passivo, este não foi abordado por compreender-se enquanto habilidade técnica e pelo limite para reprodução do áudio (cinco minutos)

Fonte: Dados da pesquisa

A adequabilidade do podcast à relevância, segundo itens do IVCES está na tabela 29.

Tabela 29 - Frequência relativa da concordância das avaliadoras sobre a adequabilidade do podcast "Boas Práticas com Medicações Injetáveis: subsídios para o autocuidado" a relevância, segundo o Instrumento de Validação de Conteúdo Educativo em Saúde. São Carlos, 2020.

Item	Adequado (%)	Parcialmente Adequado (%)	Inadequado (%)
Estimula o aprendizado	90	10	0
Contribui para o conhecimento na área	100	0	0
Desperta interesse pelo tema	90	0	0

Fonte: Dados da pesquisa

Como sugestão, emergiu: "Acredito que para estimular o aprendizado e despertar interesse do aluno deva ter algo mais provocativo à relevância do tema" (E10).

Partindo das modificações supracitadas, compreendeu-se que a inserção de questionamentos realizados de forma direta ao participante, tornaram o *podcast* mais provocativo, neste sentido.

A fim de adequar o *podcast* ao tempo sugerido, foi gravado novamente o conteúdo pela pesquisadora, conforme escaleta a seguir e tendo-se por base um roteiro elaborado para esta ferramenta educativa, bem como utilizando-se os mesmos aplicativos e softwares para gravação e edição do podcast em sua primeira versão.

A escaleta do podcast validado está no quadro 6, enquanto a transcrição do se encontra no Apêndice L.

Quadro 6 - Escaleta do podcast “Boas Práticas com Medicações Injetáveis: subsídios para o autocuidado” pós validação do conteúdo pelas avaliadoras. São Carlos, SP, 2020.

**Início**	**Sequência**	**Áudio**	**Duração**
00:00	Vinheta de abertura	Membro da Equipe (PoCA)	09’’
00:09	Apresentação do conteúdo	Fala da pesquisadora	10’’
00:19	Apresentação da pesquisadora	Fala da pesquisadora	05’’
00:24	Apresentação do objetivo do áudio	Fala da pesquisadora	15’’
00:39	Uso de luvas para administração de medicações injetáveis: análise crítica	Fala da pesquisadora	01’12’’
01:57	Cuidados com presença de lesões nas mãos para administração de medicações injetáveis	Fala da pesquisadora	30’’
02:27	Prevenção de acidentes com perfurocortantes	Fala da pesquisadora	49’’
03:06	Cuidados em caso de acidentes com perfurocortante	Fala da pesquisadora	01’54’’
05:00	Encerramento	Fala da pesquisadora	08’’

Fonte: Dados da pesquisa

5.2.1.2.4 Processo de validação do questionário para certificação e questões do fórum do curso “Boas Práticas com Medicações Injetáveis: ações para o controle de infecção”

Para validação tanto do questionário de avaliação do conteúdo abordado pelo curso quanto para as questões do fórum, para estimular interação entre os participantes, foi avaliado o IVC de cada questão quanto à clareza e pertinência.

De forma geral, todas as questões estavam claras e pertinentes ou precisavam de pequena revisão para atingir estas qualidades.

Atendendo ao questionamento da avaliadora E9, foi inserida a referência em todos os comentários em formato de *feedback*.

Todos os feedbacks contemplando as respostas corretas passaram a reforçar positivamente as escolhas, conforme sugerido pela juíza E6.

Além disto, modificou-se a abordagem dos *feedbacks* das alternativas incorretas (“A utilização de frases como ‘Vamos tentar novamente?’ Ou ‘Essa afirmação não está correta, vamos tentar novamente?’ são maneiras de mudar a forma de abordagem” (E3).

“[...] "poxa, vamos tentar de novo? utilizar frascos com grandes quantidades para diluir medicações *in bolus* pode predispor este volume restante às contaminações” (E5).

A primeira questão atingiu IVC=1 para clareza e pertinência, contudo suas alterações realizadas (referências inseridas no *feedback* e comentário positivo para as alternativas corretas) ao passo que as alterações nas redações do *feedback* conforme sugerido pelas avaliadoras encontram-se no quadro 7.

Quadro 7 - Adequação da redação do feedback da primeira questão avaliativa do curso "Boas Práticas com Injetáveis", São Carlos, SP, 2020.

**Redação inicial**	**Adequação realizada**
a) Selecione o frasco de 100 ml para aspirar o total de diluente (20 ml de SF 0,9%) e armazene o restante do frasco para diluições futuras (para o mesmo paciente).<br>Feedback: Poxa, vamos tentar de novo? Utilizar frascos com grandes quantidades para diluir medicações *in bolus* pode predispor este volume restante às contaminações, e levar danos aos pacientes que receberem as doses subsequentes	a) Selecione o frasco de 100 ml para aspirar o total de diluente (20 ml de SF 0,9%) e armazene o restante do frasco para diluições futuras (para o mesmo paciente).<br>Feedback: Vamos tentar de novo? Utilizar frascos com grandes quantidades para diluir medicações *in bolus* pode predispor este volume restante às contaminações (BRASIL, 2017; ORGANIZAÇÃO MUNDIAL DA SAÚDE, 2010).

Fonte: Dados da pesquisa

A segunda questão, a qual abordava a HM foi modificada a fim de atender à sugestão da avaliadora (embora tenha atingido IVC=1 para clareza e pertinência): “Acredito que o termo "é correto o que se afirma" deva vir antes das afirmativas, logo após explicar a importância da higienização das mãos, ou algo, ‘selecione as afirmativas com a informação correta’” (E4); “Sugestão para a questão: ’É correto o que se afirma nos itens:’ ou ‘Assinale as alternativas que contenham os itens corretos:’” (E6).

Quadro 8 - Adequação da redação da segunda questão avaliativa do curso "Boas Práticas com Injetáveis", São Carlos, SP, 2020.

Redação inicial	Adequação realizada
A higienização das mãos é primordial para a redução de infecção em serviços de saúde, pois, remove os microrganismos a partir do emprego de água e sabão ou solução alcoólica. É correto considerar que, ao realizar uma medicação endovenosa:<br>I. Deve-se higienizar as mãos antes da inserção do cateter venoso a fim de prevenir algum dano infeccioso no paciente.<br>II. Como, obrigatoriamente, deve-se utilizar luvas de procedimento para realizar o procedimento, é facultativa a higienização das mãos após a retirada deste equipamento de proteção individual.<br>III. Deve-se higienizar as mãos após a manipulação de cateteres venosos periféricos pois protege ao profissional ou estudante que realizou esta ação.<br>É correto o que se afirma:	A higienização das mãos é primordial para a redução de infecção em serviços de saúde, pois, remove os microrganismos a partir do emprego de água e sabão ou solução alcoólica. Considere as afirmações a seguir:<br>I. Deve-se higienizar as mãos antes da inserção do cateter venoso a fim de prevenir algum dano infeccioso no paciente.<br>II. Como, obrigatoriamente, deve-se utilizar luvas de procedimento para realizar o procedimento, é facultativa a higienização das mãos após a retirada deste equipamento de proteção individual.<br>III. Deve-se higienizar as mãos após a manipulação de cateteres venosos periféricos para prevenir a contaminação das mãos daquele que realizou esta ação.<br>Assinale as alternativas que contenham as afirmações corretas:

Fonte: Dados da pesquisa

A terceira questão, em que requeria que lacunas fossem preenchidas com palavras referentes ao *flushing* e proibição ao reuso de insumos descartáveis possuiu IVC= 1 para clareza e para pertinência.

Foram sugestões das avaliadoras para esta questão: “Está ótima a questão, mas para ficar ainda mais compreensível, antes da frase com as lacunas para completar, colocaria algo do tipo: Escolha a opção que complete corretamente a afirmativa abaixo” (E4) e “Sugestão para a questão: “3. Complete o parágrafo com as palavras corretas: sugestão para o início do parágrafo: “_____________ é o termo utilizado para a administração de solução fisiológica...’” (E6).

A adequação a esta questão está no quadro 9.

Quadro 9 - Adequação da redação da terceira questão avaliativa do curso "Boas Práticas com Injetáveis", São Carlos, SP, 2020.

Redação inicial	Adequação realizada
3. Diz-se ___________________ a administração de solução fisiológica com seringa de 10 ml para gerar uma ___________ pressão no lúmen do cateter intravenoso, a fim de manter sua integridade e _____________, prevenir obstrução e ___________ de microrganismos. Deve-se realizar o procedimento antes e após cada administração endovenosa. É importante utilizar seringas _______________ pois o reuso é _______________ mesmo se tratando de procedimento no mesmo paciente.	3. Complete o parágrafo com as palavras corretas:<br><br>_______________ é o termo utilizado para a administração de solução fisiológica com seringa de 10 ml para gerar uma _______________ pressão no lúmen do cateter intravenoso, a fim de manter sua integridade e _______________, prevenir obstrução e _______________de microrganismos. Deve-se realizar o procedimento antes e após cada administração endovenosa. É importante utilizar seringas _______________pois o reuso é _______________mesmo se tratando de procedimento no mesmo paciente.

Fonte: Dados da pesquisa

Foi realizada adequação à redação do *feedback* da questão supracitada, conforme sugerido pela avaliadora E5: “feedback: "(...) estão são descartáveis (...); alterar para (...) estes são descartáveis (...)”.

Foi também inserida a referência utilizada, conforme apontado pela avaliadora E9 que fez este questionamento geral “Os feedbacks não possuem as referências?”

Estas modificações constam no quadro 10.

Quadro 10 - Adequação da redação do feedback da terceira questão avaliativa do curso "Boas Práticas com Injetáveis", São Carlos, SP, 2020.

Redação inicial	Adequação realizada
O flushing é um procedimento que deve suceder toda medicação injetável. Deve ser realizado com seringa de 10 ml, assim, exerce uma baixa pressão no lúmen do cateter, prevenido danificações no cateter, sem prejuízo na verificação de resistência. O flushing visa remover resíduos do cateter, prevenindo a nidação (ou fixação) de microrganismos. Para garantir a esterilidade e segurança deste procedimento, deve-se utilizar insumos estéreis e descartá-los em seguida, pois, seringas e agulhas são insumos descartáveis, de uso único e não podem ser reutilizadas, mesmo se tratando do mesmo paciente.	O flushing é um procedimento que deve suceder toda medicação injetável. Deve ser realizado com seringa de 10 ml, assim, exerce uma baixa pressão no lúmen do cateter, prevenido danificações no cateter, sem prejuízo na verificação de resistência. O flushing visa remover resíduos, como depósitos de fibrina, do lúmen do cateter, prevenindo a colonização por microrganismos. Para garantir a esterilidade e segurança deste procedimento, deve-se utilizar insumos estéreis e descartá-los em seguida, pois, seringas e agulhas são insumos de uso único e não podem ser reutilizadas, mesmo se tratando do mesmo paciente (BRASIL, 2017; FERRONI *et al*, 2014).

Fonte: Dados da pesquisa

A quarta questão, que abordava sobre acidente ocupacional com perfurocortante, embora tenha recebido IVC igual 1 para clareza e pertinência, recebeu sugestão para melhora da redação:

> "No item 'II) Caso ele esteja com o esquema vacinal contra hepatite B(...)'; sugiro incluir a palavra 'completo': 'II) caso ele esteja com o esquema vacinal completo contra hepatite b (...)'; faltou 'ponto final' ao final da redação dos itens III e IV; comentário final: "corrente sanguíne", faltou a letra a" (E5).

A mesma questão também recebeu sugestão de outra avaliadora: "Sugestão para a questão (caso o *podcast* também seja modificado): "ao reencapar a agulha já utilizada com as duas mãos" (E6).

A primeira sugestão foi atendida, porém, como optou-se por não abordar o reencape passivo no *podcast*, a segunda sugestão não pode ser contemplada, ficando ao final, redigida conforme quadro 11:

Quadro 11 - Adequação da redação da quarta questão avaliativa do curso "Boas Práticas com Injetáveis", São Carlos, SP, 2020.

Redação inicial	Adequação realizada
Ao realizar uma medicação intramuscular em uma Unidade de Pronto Atendimento (UPA), Fernando acaba perfurando sua mão, ao reencapar a agulha já utilizada. Como ele é recém-contratado, está receoso de sofrer alguma sanção ao reportar o ocorrido ao serviço, já que, na ocasião, não estava utilizando luvas de procedimento.<br>Analise as colocações a seguir:<br>I) É obrigatório o uso de luvas para qualquer injeção intramuscular.<br>II) Caso ele esteja com o esquema vacinal contra hepatite B, não há risco algum neste evento acidental.<br>III) O evento deve ser reportado e notificado ao serviço, pois, há riscos à saúde do profissional<br>IV) O evento deve ser reportado e notificado ao serviço, pois, há riscos à saúde do profissional e do paciente que recebeu a injeção<br>É correto o que se afirma em:	Ao realizar uma medicação intramuscular em uma Unidade de Pronto Atendimento (UPA), Fernando acaba perfurando sua mão, ao reencapar a agulha já utilizada. Como ele é recém-contratado, está receoso de sofrer alguma sanção ao reportar o ocorrido ao serviço, já que, na ocasião, não estava utilizando luvas de procedimento.<br>Analise as colocações a seguir:<br>I) É obrigatório o uso de luvas para qualquer injeção intramuscular.<br>II) Caso ele esteja com o esquema vacinal completo contra hepatite B, não há risco algum neste evento acidental.<br>III) O evento deve ser reportado e notificado ao serviço, pois, há riscos à saúde do profissional.<br>IV) O evento deve ser reportado e notificado ao serviço, pois, há riscos à saúde do profissional e do paciente que recebeu a injeção.<br>É correto o que se afirma em:

Fonte: Dados da pesquisa

No que concerne à quinta questão (utilização de álcool a 70% enquanto desinfetante e cuidado a lesão por acidente com perfurocortante, esta atingiu IVC para clareza e para pertinência igual a 1.

Para esta questão, foram sugeridas nova redação: "A versatilidade da solução alcoólica enquanto desinfetante e solução antisséptica de" (E2); “sugestão de nova redação para a alternativa c: Esta afirmação é parcialmente verdadeira, visto que não se deve empregar solução alcoólica para a limpeza de bandejas, já que não abrange microrganismos que possam estar nestes materiais” (E5).

A modificação realizada na quinta questão pode ser observada no quadro 12.

Quadro 12 - Adequação da redação da alternativa ‘c’ da quinta questão avaliativa do curso "Boas Práticas com Injetáveis", São Carlos, SP, 2020.

Sugestão realizada pelas avaliadoras	Adequação realizada
c) Esta afirmação é parcialmente verdadeira, visto que não se deve empregar solução alcoólica para a limpeza de bandejas, já que não abrange microrganismos que possam estar nestes recursos.	c) Esta afirmação é parcialmente verdadeira, visto que não se deve empregar solução alcoólica para a limpeza de bandejas, já que não abrange microrganismos que possam estar nestes materiais

Fonte: Dados da pesquisa

As questões validadas para a certificação dos participantes podem ser lidas no “Apêndice M”.

Com relação às questões disparadoras de discussão, a primeira “Você já experienciou, algum acidente com perfurocortante, durante sua prática em serviços de saúde? Como isso influenciou a forma como você encara a prática com injetáveis?” atingiu IVC igual a 1 para clareza e para pertinência, sendo modificada sua redação, conforme sugerido pela avaliadora E1: “Você já experienciou, algum acidente com perfurocortante, durante sua prática em serviços de saúde? Se sim, esse fato lhe causou algum impacto?”.

A segunda questão “Você costuma realizar treinamentos para manuseio de injetáveis com dispositivos de segurança? Conte um pouco sobre sua experiência com estes recursos” atingiu IVC igual a 1 para clareza e pertinência. Para tanto, atendeu-se a sugestão de, ao invés de “realizar treinamentos” utilizar-se “participar de treinamentos”, conforme sugerido por E7.

Destaca-se sugestão da avaliadora E7, de que, por tratar-se de curso assíncrono, o fórum poderia ser subutilizado, já que não teria devolutiva do tutor, contudo,

optou-se por mantê-lo a fim de que as instruções para inserção de cursos na plataforma escolhida (PoCA) fossem atendidas.

5.3 IMPLEMENTAÇÃO

A implementação do material educativo no PoCA ocorreu com auxílio da equipe especializada do referido portal, o qual foi finalizado, após registro do curso na Pró-Reitoria de Extensão (Proc. N° 23112.015764/2020-90) em agosto de 2020, como atividade de extensão, em formato EAD, sem tutoria.

O início do curso ocorreu em 20 de novembro de 2020, local em que permanecerá ativo por dois anos, na plataforma, contudo, durante este tempo, o material educativo (*ebook* e *podcast*) poderão ser baixados e armazenados em nuvem, ou, no caso do *ebook* e transcrição do áudio, impressos para consulta.

Este curso (que requer conexão com *internet*) pode ser acessado a partir de navegadores *Web* (https://cursos.poca.ufscar.br/course/view.php?id=79) ou de aplicativo para celulares do *Moodle.*

A página inicial do PoCA (https://poca.ufscar.br/), plataforma que acolheu este curso, pode ser visualizada na figura a seguir:

Figura 6 - Página inicial do PoCA, acessada via navegador Google Chrome. São Carlos, SP, 2021.

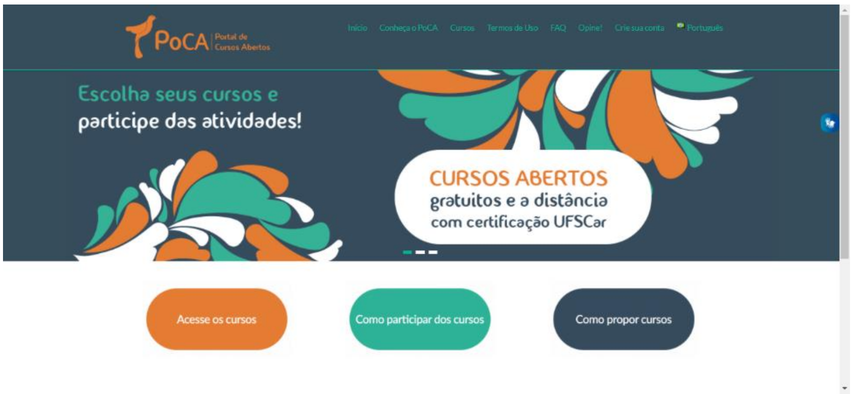

Fonte: Portal de Cursos Abertos da Universidade Federal de São Carlos

Através do link “acesse os cursos”, o participante é direcionado a uma página de cadastro para confirmação da conta, via e-mail pessoal. Posteriormente a este acesso, ele terá acesso a todos os cursos disponíveis na plataforma.

O curso “Boas Práticas com Medicações Injetáveis: ações para o controle de infecção”, inicialmente, esteve dentro dos “cursos em destaque” e, posteriormente, “saúde”, visualizado após realização de login na plataforma:

Figura 7 - Página inicial do curso "Boas Práticas com Injetáveis: ações para o controle de infecção". São Carlos, SP, 2021.

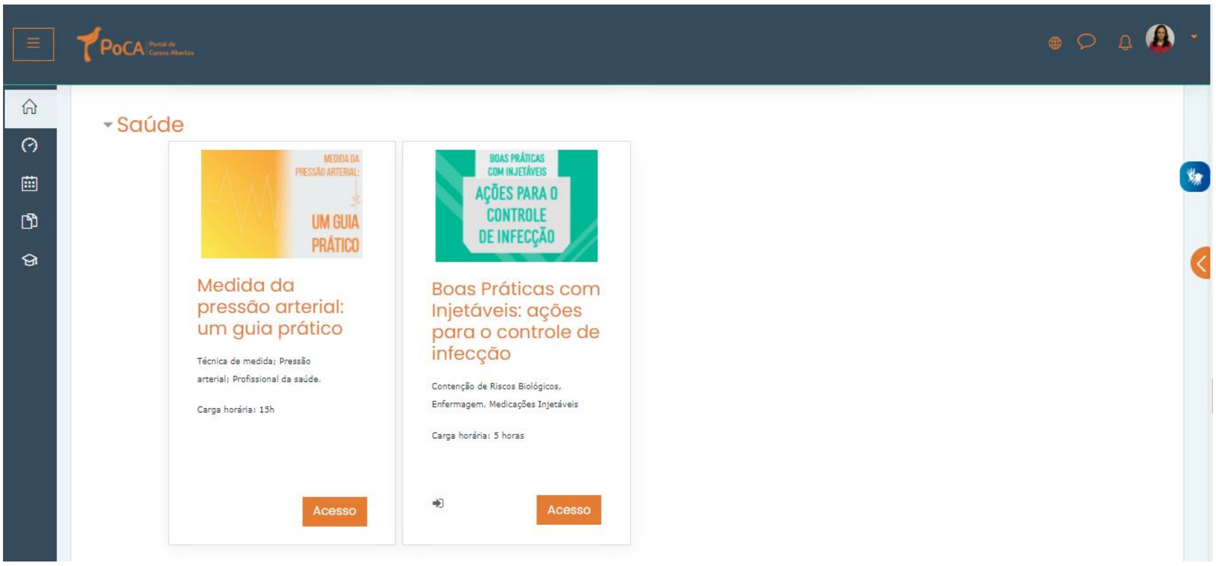

Fonte: Portal de Cursos Abertos da Universidade Federal de São Carlos

Ao clicar em “acesso”, o usuário conhece o curso através da aba “Apresentação”, que engloba o título, nomes e fotos das autoras, descrição, objetivos e unidade do curso, carga horária, possibilidade de certificado, e reafirma o compromisso do aluno, que deve acessar, estudar e realizar o curso.

Na aba superior, ao clicar na imagem referente ao perfil, o participante pode acessar ao painel do curso, perfil, notas, mensagens, preferências do site, notificações (preferências, por exemplo, *pop up* ou *e-mail* de acordo com as ações no curso) e preferência de idioma do portal (inglês, português ou espanhol).

À esquerda, ao clicar nos ícones fixos o estudante pode navegar pela plataforma, acessando, por exemplo, à seção do curso, página inicial, participantes do curso e calendário.

A aba “navegação” permite ao participante acessar a página do site e dos cursos que está inscrito, sendo acessada após clicar na seta, à direita.

A interface do curso pode ser observada nas figuras a seguir:

Figura 8 - Interface da apresentação do curso "Boas Práticas com Injetáveis: ações para o controle de infecção".

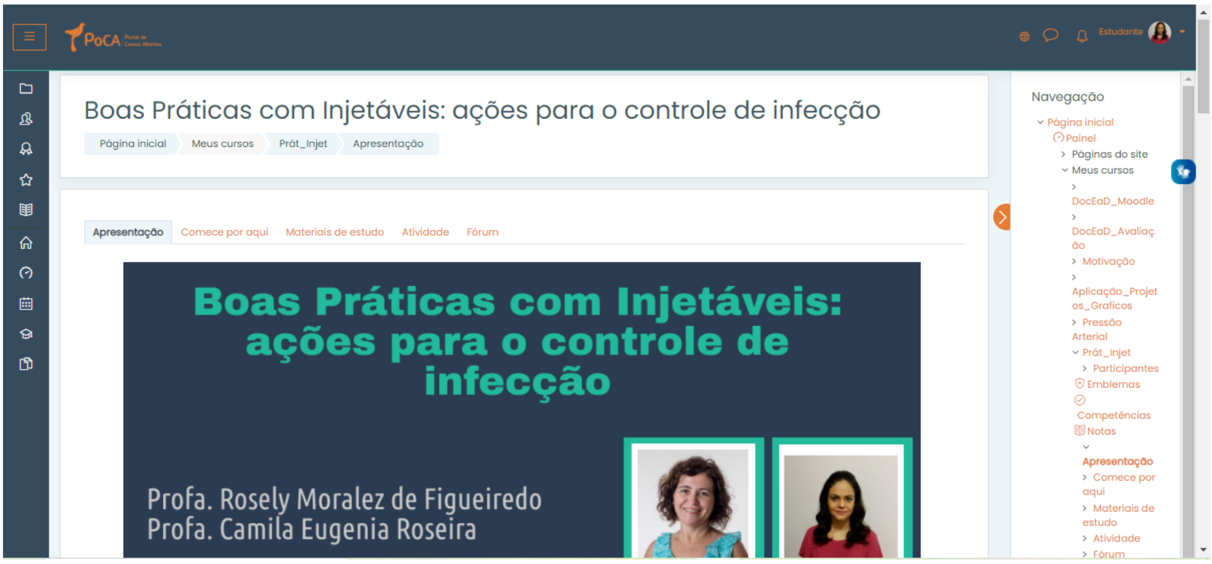

Fonte: Portal de Cursos Abertos da Universidade Federal de São Carlos

No que concerne à aba "comece aqui", esta contempla mensagem de boas-vindas ao participante, direcionando o mesmo para o guia do curso ou materiais de estudo:

Figura 9 - Página do curso "Boas Práticas com Injetáveis: ações para o controle de infecção".

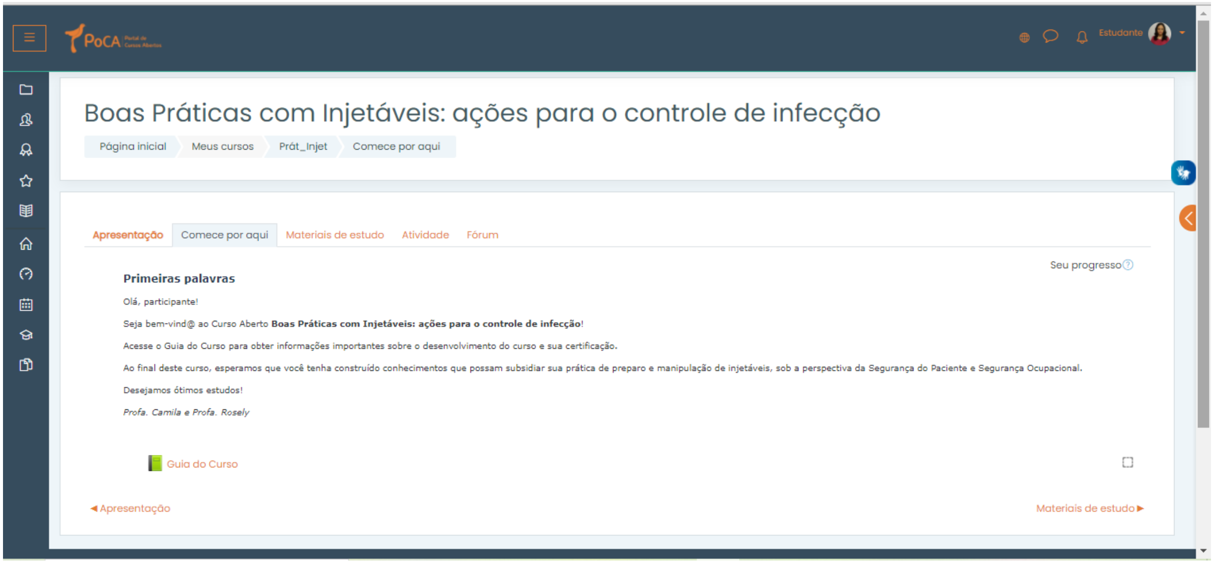

Fonte: Portal de Cursos Abertos da Universidade Federal de São Carlos

A ação concluída pelo participante é indicada a partir de uma marca na caixa com borda pontilhada, a frente do item, em questão. No caso da figura 8, esta caixa aponta que não houve visualização do guia do curso, enquanto na figura 9, referente à página

“materiais de estudo” a marca aponta que tanto a página do ebook quanto a página do podcast foram visualizadas pelo participante:

Figura 10 - Página do curso "Boas Práticas com Injetáveis: ações para o controle de infecção".

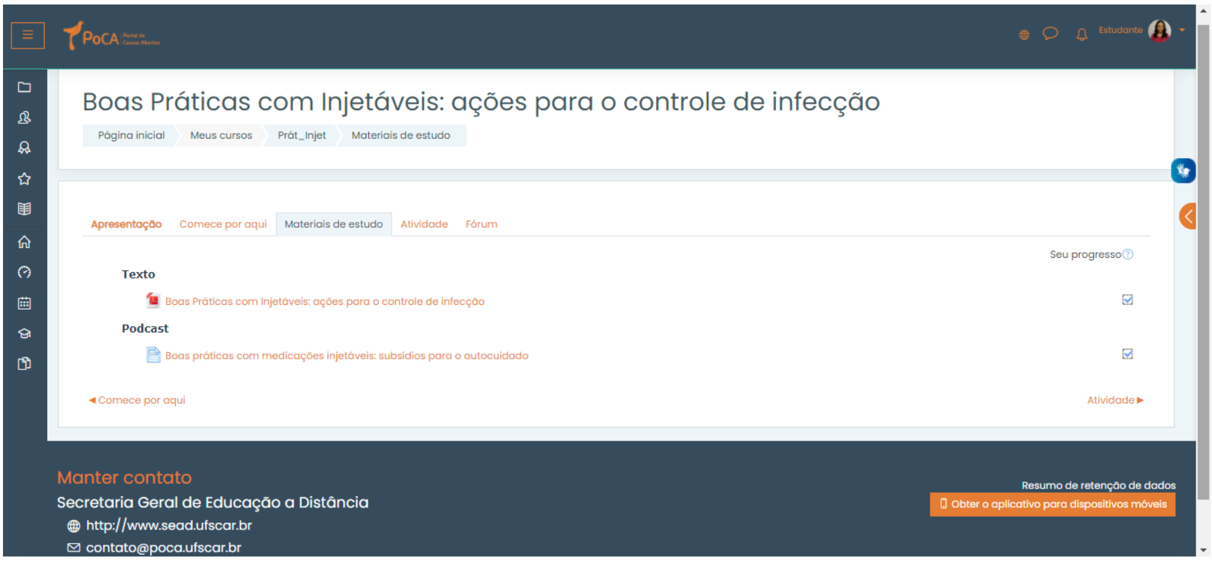

Fonte: Portal de Cursos Abertos da Universidade Federal de São Carlos

Ao clicar no ícone referente ao *ebook* “Boas Práticas com Injetáveis: ações para o controle de infecção”, conforme figura 10, o participante acessa ao *ebook*, em formato PDF, tendo a possibilidade de imprimir ou baixar o mesmo para visualizações futuras.

Figura 11 - Página do curso "Boas Práticas com Injetáveis: ações para o controle de infecção".

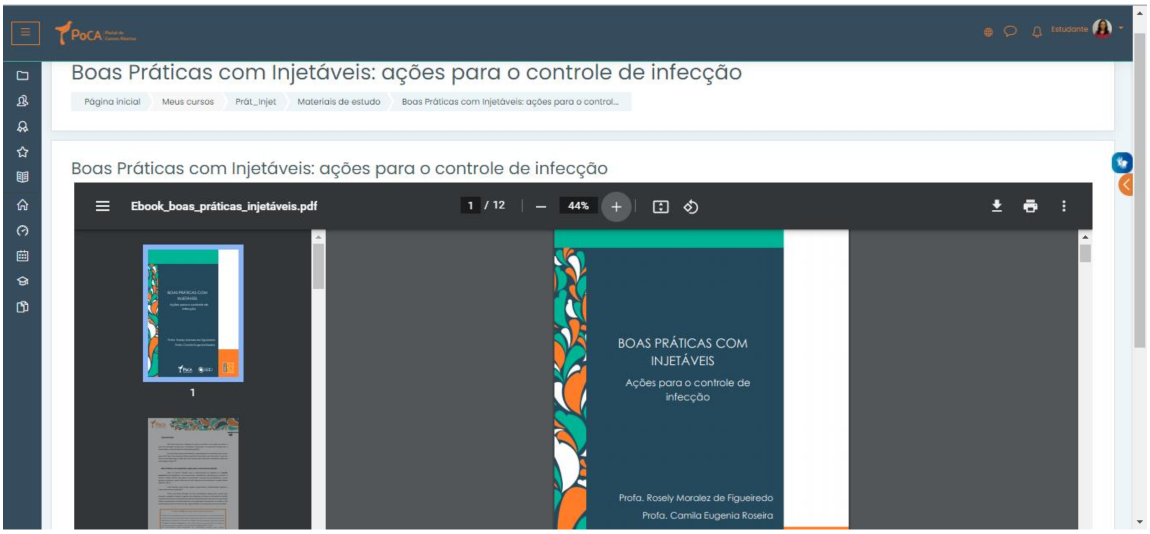

Fonte: Portal de Cursos Abertos da Universidade Federal de São Carlos

Na página referente ao *podcast* intitulado “Boas práticas com medicações injetáveis: subsídios para o autocuidado” (figura 11), o participante tem acesso ao áudio do *podcast*, gravado em MP3 (figura 12) e a transcrição do mesmo (figura 13), e às referências utilizadas para a elaboração do conteúdo deste e sua música:

Figura 12 - Página do curso "Boas Práticas com Injetáveis: ações para o controle de infecção".

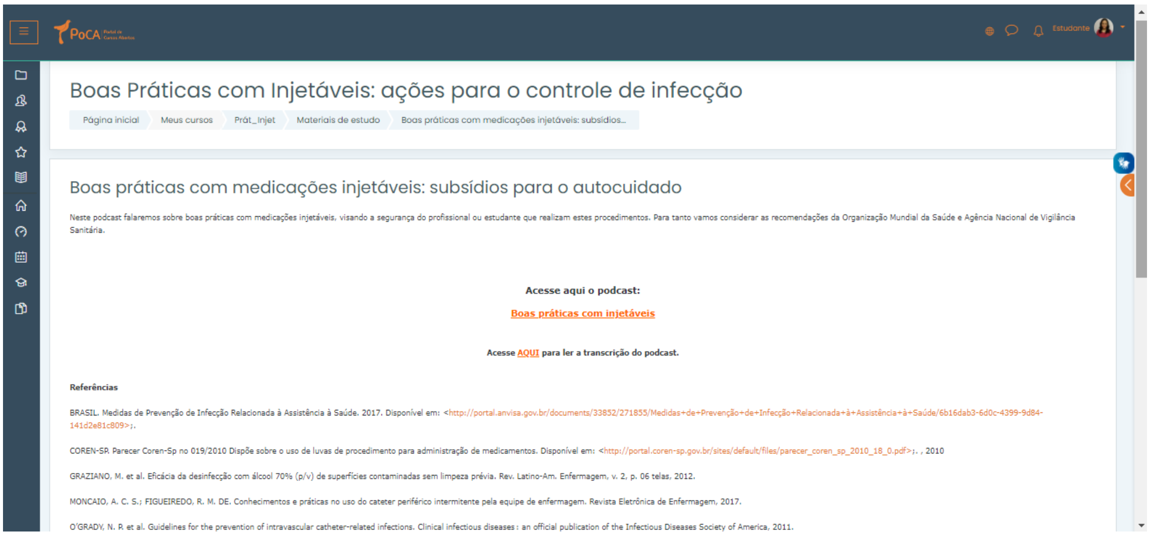

Fonte: Portal de Cursos Abertos da Universidade Federal de São Carlos

Figura 13 - Página do curso "Boas Práticas com Injetáveis: ações para o controle de infecção".

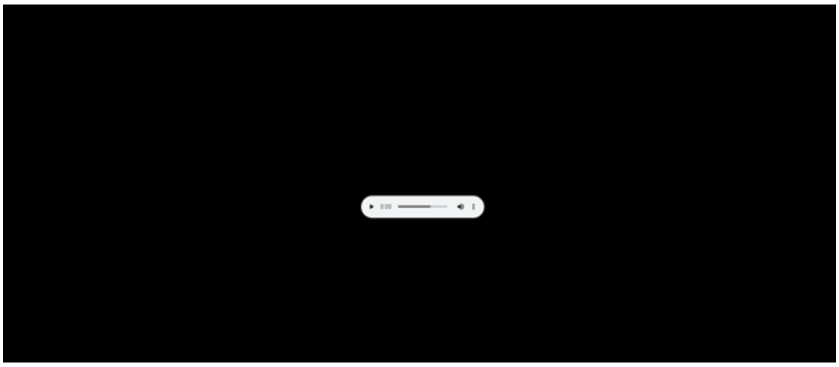

Fonte: Portal de Cursos Abertos da Universidade Federal de São Carlos

Figura 14 - Página do curso "Boas Práticas com Injetáveis: ações para o controle de infecção".

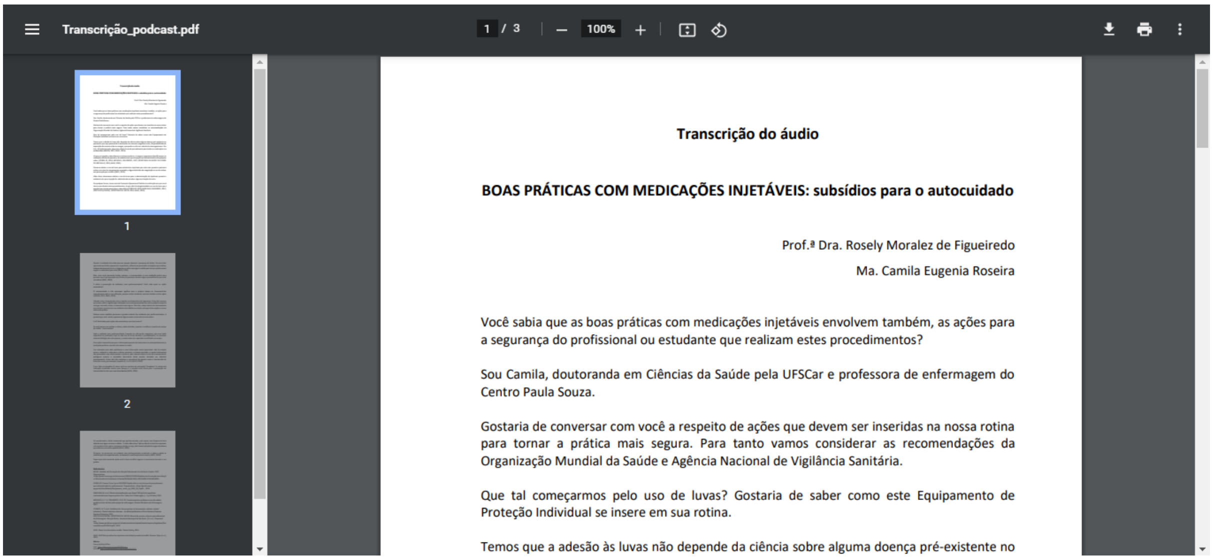

Fonte: Portal de Cursos Abertos da Universidade Federal de São Carlos

Já a aba "Atividades" (figura 14) contemplou o acesso ao questionário (para emissão de certificado) e pesquisa de satisfação da plataforma.

Figura 15 - Página do curso "Boas Práticas com Injetáveis: ações para o controle de infecção".

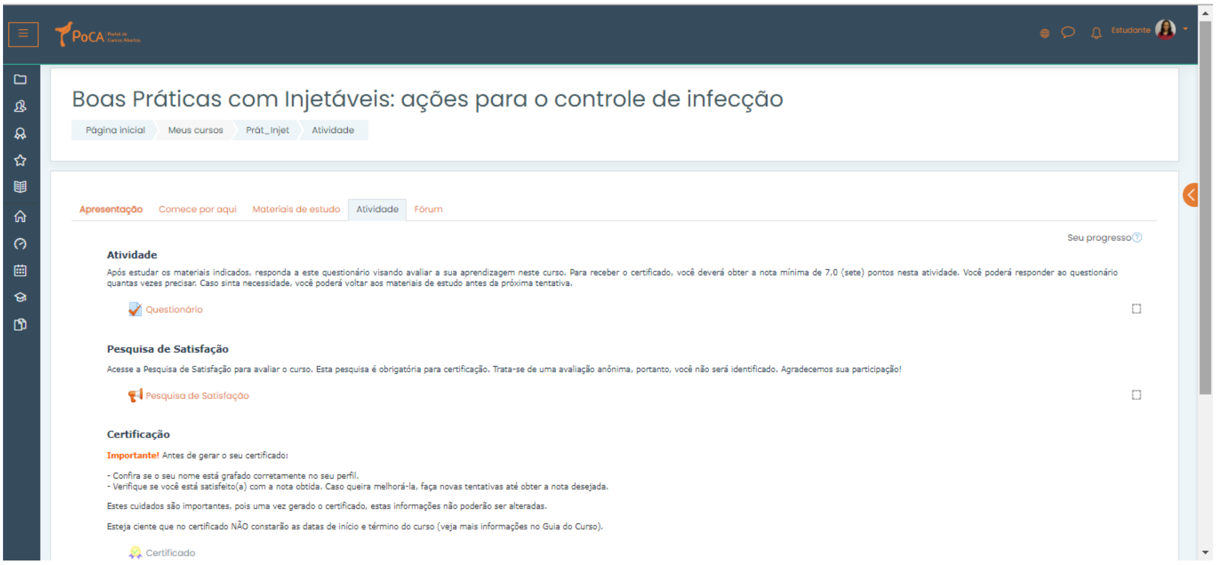

Fonte: Portal de Cursos Abertos da Universidade Federal de São Carlos

O questionário (figura 15), com cada página equivalente a uma questão, oferecia possibilidade de revisão, e o participante tem direito ao certificado (com carga horária de cinco horas) após acertar 70% deste (nota igual ou superior a 7). Os *feedbacks* e a correção do questionário poderão ser vistos nas figuras subsequentes.

Figura 16 - Página do curso "Boas Práticas com Injetáveis: ações para o controle de infecção".

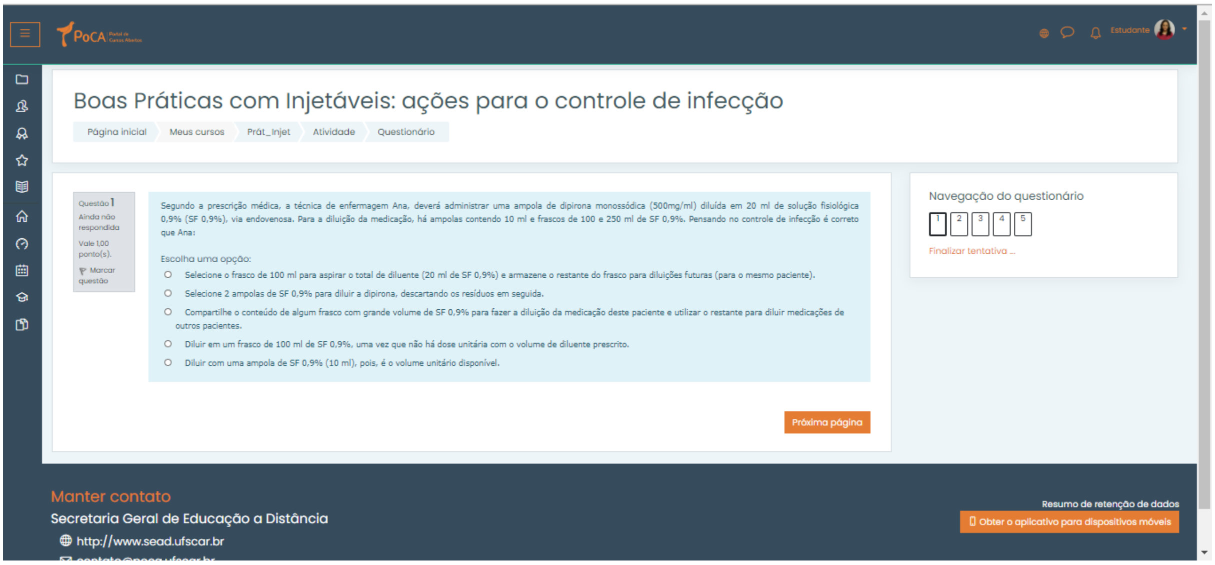

Fonte: Portal de Cursos Abertos da Universidade Federal de São Carlos

Figura 17 - Página do curso "Boas Práticas com Injetáveis: ações para o controle de infecção".

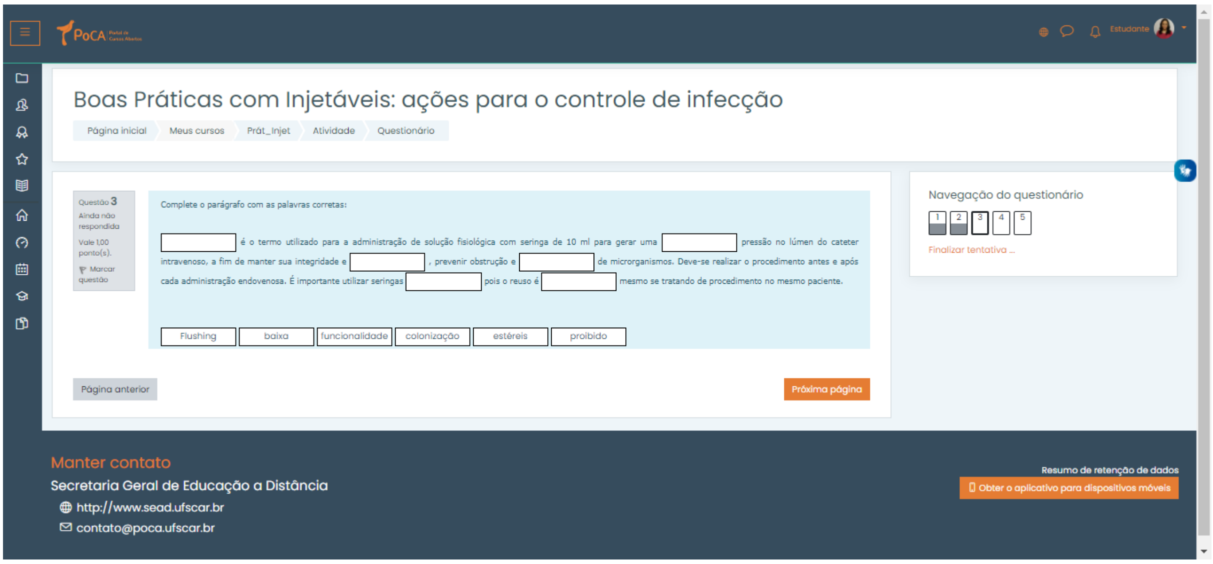

Fonte: Portal de Cursos Abertos da Universidade Federal de São Carlos

Figura 18 - Página do curso "Boas Práticas com Injetáveis: ações para o controle de infecção".

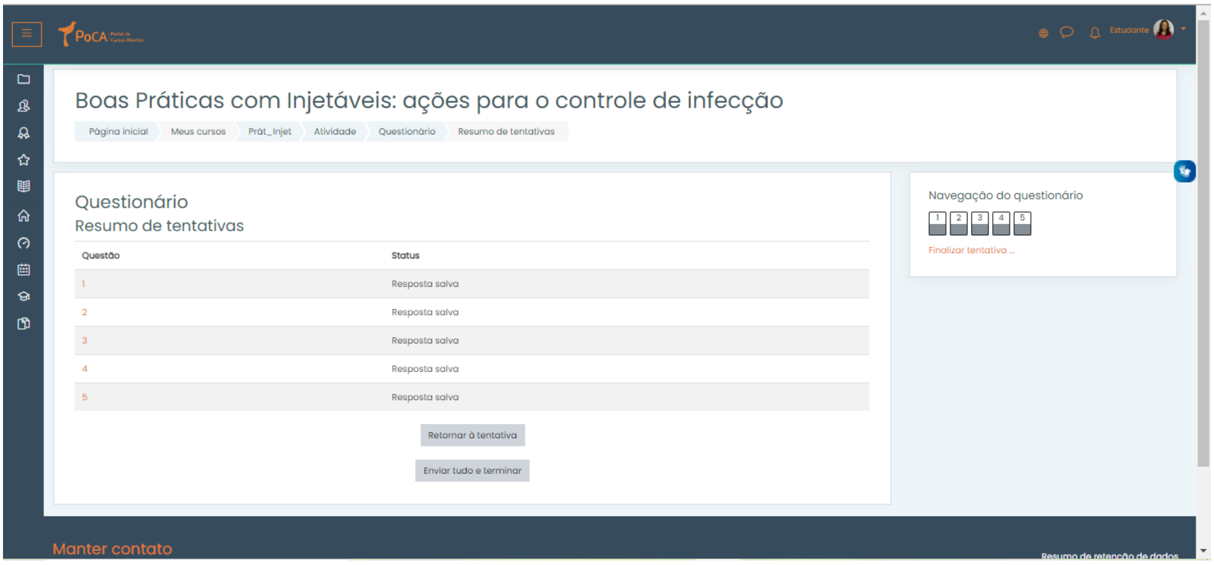

Fonte: Portal de Cursos Abertos da Universidade Federal de São Carlos

Figura 19 - Página do curso "Boas Práticas com Injetáveis: ações para o controle de infecção".

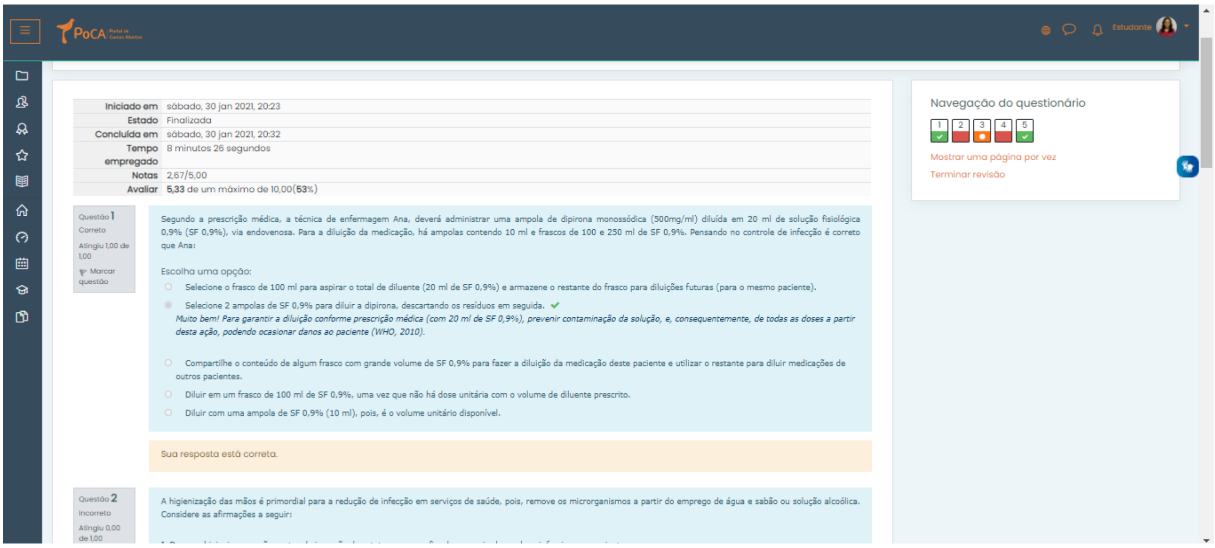

Fonte: Portal de Cursos Abertos da Universidade Federal de São Carlos

Figura 20 - Página do curso "Boas Práticas com Injetáveis: ações para o controle de infecção".

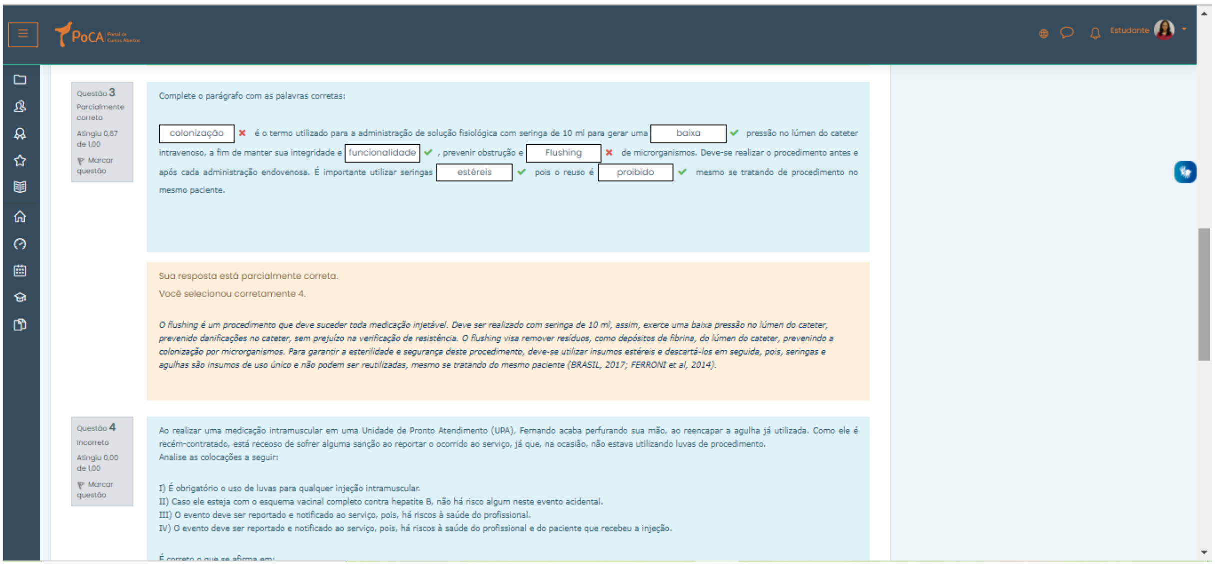

Fonte: Portal de Cursos Abertos da Universidade Federal de São Carlos

Figura 21 - Página do curso "Boas Práticas com Injetáveis: ações para o controle de infecção".

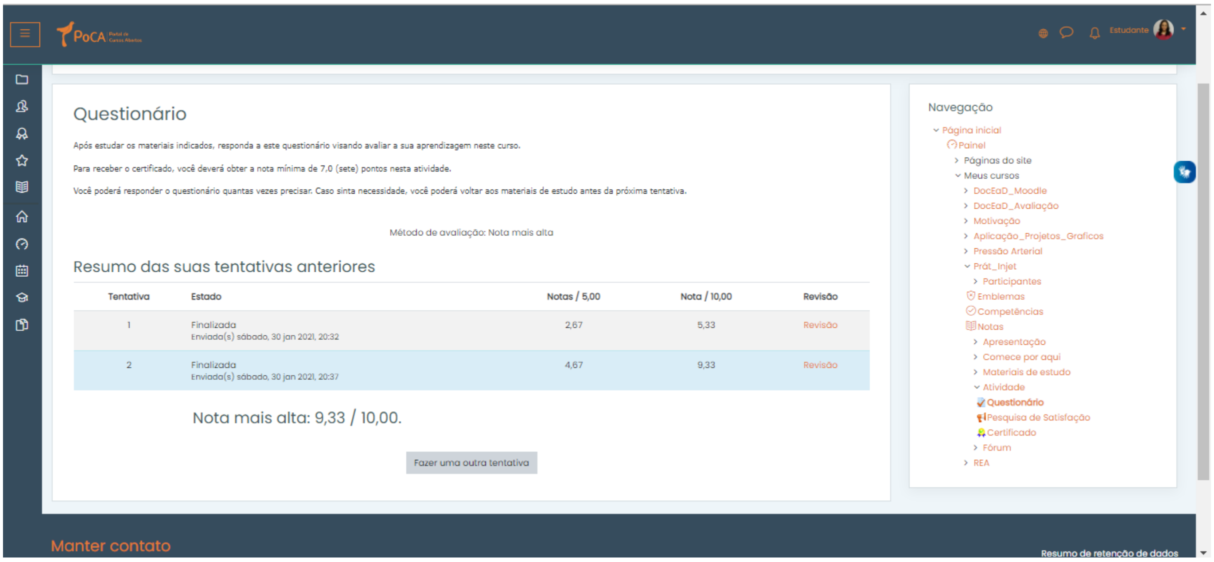

Fonte: Portal de Cursos Abertos da Universidade Federal de São Carlos

Para a emissão do certificado, o participante, além de realizar a atividade, deveria acessar ao *link* para esta ação:

Figura 22 - Página do curso "Boas Práticas com Injetáveis: ações para o controle de infecção".

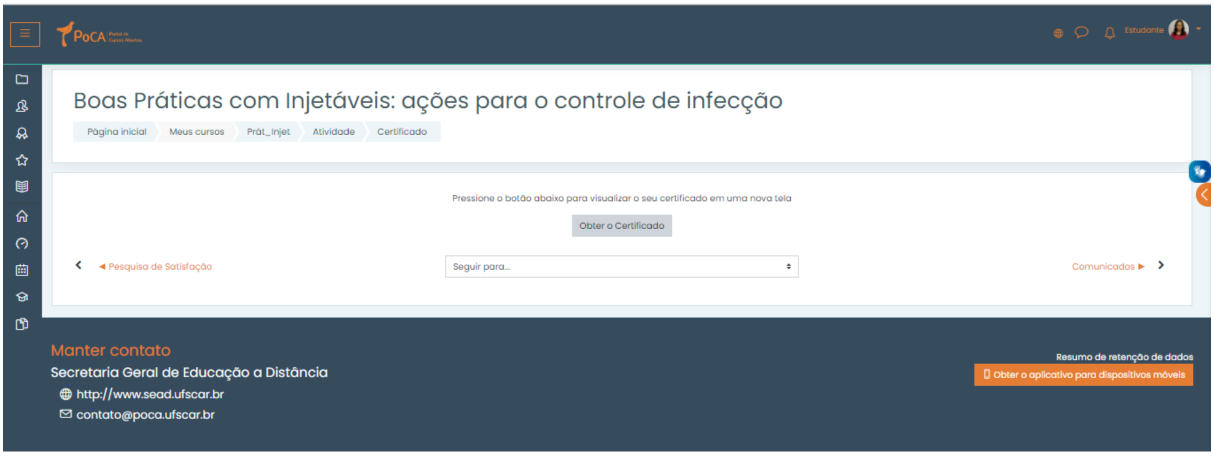

Fonte: Portal de Cursos Abertos da Universidade Federal de São Carlos

Na aba “Fórum”, o participante tem acesso aos comunicados e mensagens de fórum, ou seja, neste local, ele acessaria o *link* para participar da pesquisa para deixar a percepção do curso e sua contribuição respondendo às questões validadas para este fim. Elas serão apresentadas a seguir:

Figura 23 - Página do curso "Boas Práticas com Injetáveis: ações para o controle de infecção".

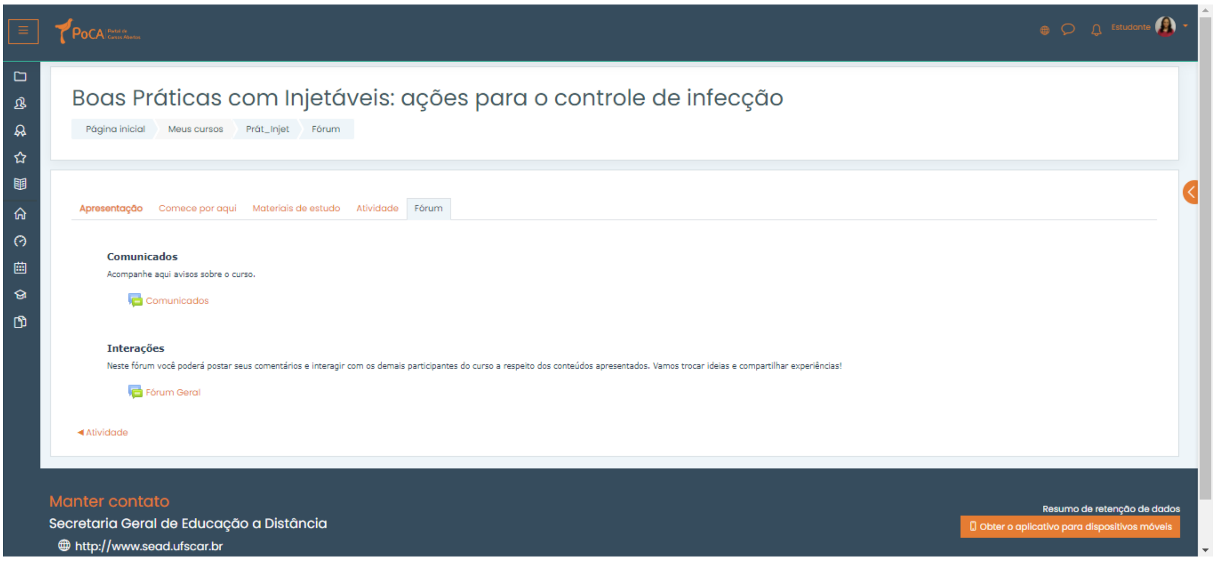

Fonte: Portal de Cursos Abertos da Universidade Federal de São Carlos

Figura 24 - Página do curso "Boas Práticas com Injetáveis: ações para o controle de infecção".

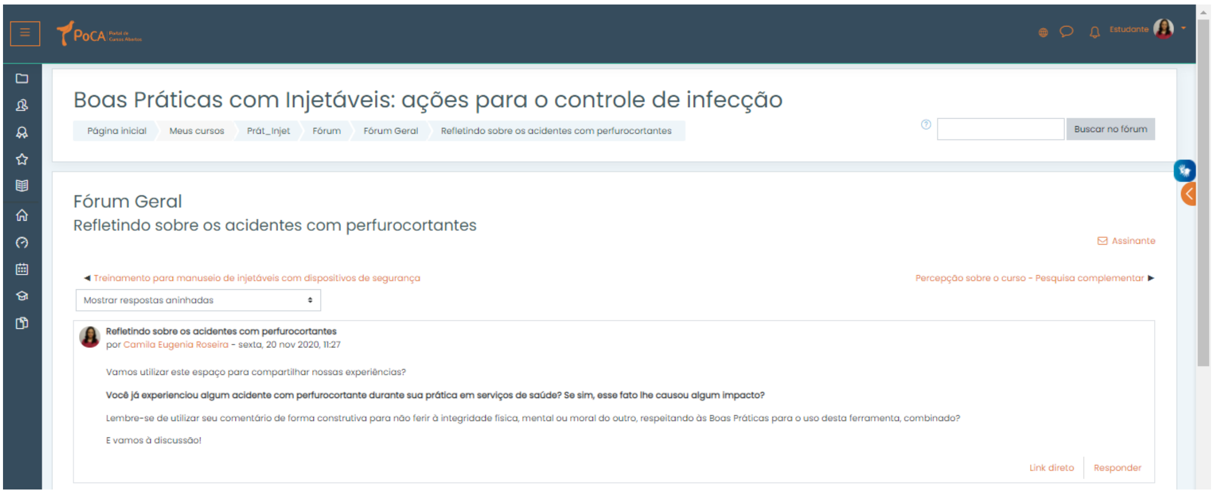

Fonte: Portal de Cursos Abertos da Universidade Federal de São Carlos

Figura 25 - Página do curso "Boas Práticas com Injetáveis: ações para o controle de infecção".

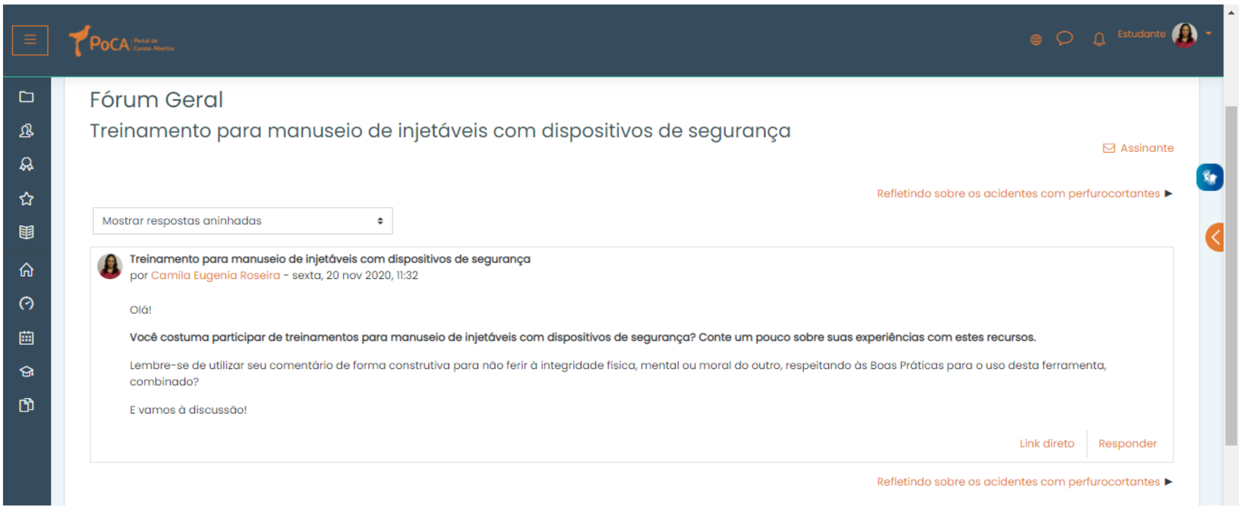

Fonte: Portal de Cursos Abertos da Universidade Federal de São Carlos

Ressalta-se que foi adquirida uma licença aberta, Creative Commons, de forma a tornar o material reprodutível, respeitando-se alguns direitos autorais, contemplando assim, a licença adquirida pelo material de terceiros (neste caso, da música utilizada para o *podcast*) utilizado neste material educativo, permitindo aos usuários copiar, traduzir, modificar, mas citando a fonte original, sem utilizar o mesmo para fins comerciais, sinalizando a escolha das autoras tanto no *ebook*, quanto na página do *podcast*:

Figura 26 - Licença do material educativo do curso "Boas Práticas com Injetáveis".

O podcast *Boas práticas com medicações injetáveis:subsídios para o autocuidado* de Camila Eugenia Roseira está licenciado com uma Licença Creative Commons - Atribuição-CompartilhaIgual 4.0 Internacional.

Fonte: Portal de Cursos Abertos da Universidade Federal de São Carlos

A fim de viabilizar a autonomia do sujeito e para que este pudesse explorar o curso conforme sua disponibilidade e volia, exceto pelo número de acertos para a emissão do certificado, não havia atividades obrigatórias.

Até o dia 30/01/21, o curso havia recebido 227 auto-inscrições, 134 concluíram o questionário (dos quais, quatro não atingiram a pontuação necessária), 118 acessaram à página do podcast, 134 acessaram o *ebook*.

Ressalta-se que não é possível identificar quantos participantes, efetivamente, pertenciam à enfermagem, visto ser um curso aberto e que não havia obrigatoriedade desta resposta no cadastro.

5.4 AVALIAÇÃO

A fim de identificar a percepção da população-alvo sobre o curso, foi disponibilizado, na própria plataforma dois campos com os convites para participarem da pesquisa, no fórum e nos comunicados do curso, bem como foram convidados aqueles que concluíram ao curso e que não responderam à pesquisa.

Para recrutar sujeitos, foram disponibilizados, também, os *links* para o acesso ao curso e para a pesquisa em redes sociais e plataformas de ensino.

O convite para a participação na pesquisa deixado no fórum, a fim de deixar a percepção sobre o curso, encontra-se a seguir:

Figura 27 - Convite deixado na plataforma para participar da pesquisa sobre a percepção do curso "Boas Práticas com Injetáveis".

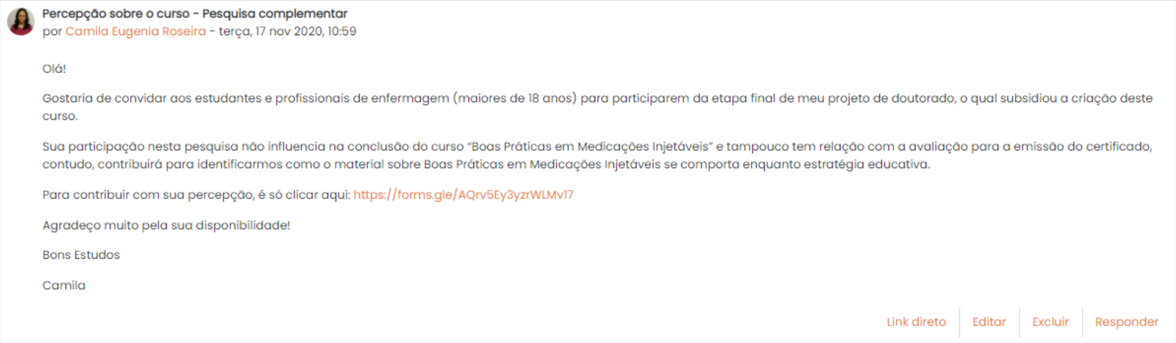
Percepção sobre o curso - Pesquisa complementar
por Camila Eugenia Roseira - terça, 17 nov 2020, 10:59

Olá!

Gostaria de convidar aos estudantes e profissionais de enfermagem (maiores de 18 anos) para participarem da etapa final de meu projeto de doutorado, o qual subsidiou a criação deste curso.

Sua participação nesta pesquisa não influencia na conclusão do curso "Boas Práticas em Medicações Injetáveis" e tampouco tem relação com a avaliação para a emissão do certificado, contudo, contribuirá para identificarmos como o material sobre Boas Práticas em Medicações Injetáveis se comporta enquanto estratégia educativa.

Para contribuir com sua percepção, é só clicar aqui: https://forms.gle/AQrv5Ey3yzrWLMv17

Agradeço muito pela sua disponibilidade!

Bons Estudos

Camila

Link direto | Editar | Excluir | Responder

Fonte: Portal de Cursos Abertos da Universidade Federal de São Carlos

5.4.1 Construção do Instrumento de Coleta de Dados

O questionário para identificação da percepção da população-alvo sobre o curso "Boas Práticas com Injetáveis: ações para o controle de infecção" foi construído com base em instrumentos utilizados em avaliação de cursos realizados em plataformas, em modalidade à distância e indicadores da qualidade destes (GALDINO; MERCADO, 2018; MARTINS; ZERBINI, 2015; PIMENTEL; PINTO; MERCADO, 2010; PRADO; VAZ; ALMEIDA, 2011) e objetivava identificar a percepção da população-alvo sobre o curso "Boas Práticas com Medicações Injetáveis: ações para o controle de infecção".

O questionário tipo múltipla-escolha poderia ser respondido a partir de uma escala de gradação de concordância e foi disponibilizado a partir do Google Forms (APÊNDICE N), conforme sugerido pela equipe do PoCA e a fim de não conflitar com a pesquisa de satisfação da plataforma.

O *link* de acesso para a participação da pesquisa foi divulgado juntamente com o *link* de acesso ao curso através de redes sociais, aplicativos de mensagens instantâneas, plataformas de comunicação e em formato de mensagem aos inscritos no curso que houvessem concluído ao mesmo e visualizado ao *ebook* e *podcast*/transcrição, na forma de convite e em formato de "Comunicado" e no campo "Fórum Geral" na página do curso.

5.4.2 Caracterização dos Participantes da Pesquisa

Neste período, embora 118 participantes tenham concluído o questionário, 30 não visualizaram o *podcast* e dois não visualizaram o *ebook*, ou seja, 85 haviam visualizado

o material do curso e realizado o questionário.

Sobre o acesso ao *link* da pesquisa (https://forms.gle/AQrv5Ey3yzrWLMv17) recebeu respostas de 51sujeitos, contudo, após exclusão daqueles que não haviam realizado o curso (25), que haviam realizado a pesquisa primeiro e o curso após (quadro), que não acessaram a todo o material do curso – página do *ebook*, página do *podcast* e questionário – (quatro) ou não aceitaram a utilização de suas respostas enquanto dados de pesquisa (um), 17 pessoas contemplaram esta avaliação.

Dentre as 17 pessoas, 82,4% eram do sexo feminino, com idade entre 18 e 57 anos (30,7±10,8), mediana de 31 e moda de 33. Destes, 94,12% provenientes do estado de São Paulo e o restante de Dundo (Angola).

Sobre o dispositivo utilizado para acessar o curso, o computador foi o mais utilizado (70, 59%), seguido pelo uso de computador e celular (17,65%).

Já sobre a atuação na área, 35,29% (6/17) afirmaram ser enfermeiros, 17,65% (3/17) referiram ser técnicos de enfermagem, 5,88% (1/17) auxiliar de enfermagem; 17,65% (3/17) eram alunos de pós-graduação em enfermagem, 11,76% (2/17) alunos de curso de graduação de enfermagem e 41,18% (7/17) alunos de curso técnico de enfermagem.

5.4.3 A percepção dos participantes sobre o curso “Boas práticas com Injetáveis: ações para o controle de infecção”

A frequência relativa de concordância dos participantes é abordada na tabela 30

Tabela 30 - Frequência relativa segundo gradação de concordância da percepção da população alvo sobre o curso "Boas Práticas com Injetáveis: ações para o controle de infecção". São Carlos, SP, 2021.

(continua)

Item	Concordo totalmente		Concordo parcialmente		Não concordo, nem discordo		Discordo parcialmente		Discordo totalmente	
	**%**	**n**	**%**	**n**	**%**	**n**	**%**	**n**	**%**	**n**
O conteúdo abordado pelo material educativo está relacionado ao objetivo do curso	100,00	17	0	0	0	0	0	0	0	0
O conteúdo abordado pelo curso é relevante para a prática com injetáveis em serviços de saúde	100,00	17	0	0	0	0	0	0	0	0
O conteúdo abordado é útil para os participantes do curso	100,00	17	0	0	0	0	0	0	0	0
O conteúdo é exposto de forma clara e coerente	100,00	17	0	0	0	0	0	0	0	0
A quantidade de carga horária sugerida é suficiente para abordar ao conteúdo	82,35%	14	17,65	3	0	0	0	0	0	0
O material educativo possui linguagem clara, objetiva e dialógica	100,00	17	0	0	0	0	0	0	0	0
O curso possui uma interface simples e de fácil manuseio	94,12	16	5,88	1	0	0	0	0	0	0
O curso apresenta-se de forma atrativa	82,24	15	11,76	2	0	0	0	0	0	0
A sequência do conteúdo está organizada de forma lógica	94,12	16	5,88	1	0	0	0	0	0	0
O participante é estimulado a desenvolver um papel ativo e independente durante o manuseio do material do curso	82,35	14	17,65%	3	0	0	0	0	0	0
O participante é estimulado a refletir criticamente sobre a prática com injetáveis em serviços de saúde	100,00	17	0	0	0	0	0	0	0	0

Tabela 30 – Frequência relativa segundo gradação de concordância da percepção da população alvo sobre o curso "Boas Práticas com Injetáveis: ações para o controle de infecção". São Carlos, SP, 2021.

(continuação)

Item	Concordo totalmente		Concordo parcialmente		Não concordo, nem discordo		Discordo parcialmente		Discordo totalmente	
	**%**	**n**	**%**	**n**	**%**	**n**	**%**	**n**	**%**	**n**
O curso instiga para o aprofundamento teórico do tema	94,12	16	5,88	1	0	0	0	0	0	0
O material educativo do texto "Boas Práticas com Injetáveis: ações para o controle de infecção" está distribuído de forma harmônica no espaço limitado à página (texto e imagens)	100,00	17	0	0	0	0	0	0	0	0
O material educativo do texto "Boas Práticas com Injetáveis: ações para o controle de infecção" está distribuído de forma harmônica no espaço limitado à página (texto e imagens)	100,00	17	0	0	0	0	0	0	0	0
O material educativo do texto "Boas Práticas com Injetáveis: ações para o controle de infecção" possui texto de fácil leitura	100,00	17	0	0	0	0	0	0	0	0
O material educativo do texto "Boas Práticas com Injetáveis: ações para o controle de infecção" transmite a mensagem de forma clara e concisa	100,00	17	0	0	0	0	0	0	0	0
As imagens do material educativo do texto "Boas Práticas com Injetáveis: ações para o controle de infecção" são significativas para a aprendizagem	100,00	17	0	0	0	0	0	0	0	0
Os links disponibilizados para acesso complementam o material do curso	100,00	17	0	0	0	0	0	0	0	0
O áudio do podcast "Boas práticas com medicações injetáveis: subsídios para o autocuidado" apresenta fluidez sonora e boa dicção	94,12	16	5,88	1	0	0	0	0	0	0

Tabela 30 – Frequência relativa segundo gradação de concordância da percepção da população alvo sobre o curso "Boas Práticas com Injetáveis: ações para o controle de infecção". São Carlos, SP, 2021.

(conclusão)

**Item**	**Concordo totalmente**		**Concordo parcialmente**		**Não concordo, nem discordo**		**Discordo parcialmente**		**Discordo totalmente**	
O fórum possibilita interação entre os participantes do curso	64,71	11	11,76	2	23,53	4	0	0	0	0
O fórum favorece o aprendizado e o compartilhamento de conhecimento entre os participantes	70,59	12	11,76	2	17,64	3	0	0	0	0
A atividade proposta para emissão do certificado é pertinente com os conteúdos trabalhados no material sobre Boas Práticas com Injetáveis	100,00	17	0	0	0	0	0	0	0	0
A atividade proposta para emissão do certificado proporciona a relação entre a teoria e a prática com injetáveis	94,12	16	5,88	1	0	0	0	0	0	0
A atividade proposta para a emissão do certificado é motivadora e estimula a resolução com entusiasmo	82,35	14	11,76	2	5,88	1	0	0	0	0
As ferramentas, como áudio, links e carregamento de imagens apresentam boa funcionalidade	88,24	15	0	0	5,88	1	5,88	1	0	0

Fonte: Dados da pesquisa

Como sugestões de melhoria para o curso, emergiram: melhorias na plataforma devido a oscilações, principalmente na aba atividade, durante a mudança de questões por 5,88% (1/17) e inserção de vídeo no material por 11,76% (2/17).

6 DISCUSSÃO DOS RESULTADOS

6.1 PRÁTICA COM INJETÁVEIS: UMA ANÁLISE CONTEXTUAL

6.1.1 Questionário para Identificação das Boas Práticas com Medicações Injetáveis: Sobre seu Processo de Construção e Validação

Para o processo de construção do QBMI, este utilizou referências relevantes para o tema, uma vez que contemplou estudos que ofereciam um contexto sobre como a prática com injetáveis ocorre, a nível nacional (MONCAIO; FIGUEIREDO, 2009; NEGELISKII, 2015; PUGLIESE *et al*., 2010) e diretrizes que orientam a prática com injetáveis (BRASIL, 2016; ORGANIZAÇÃO MUNDIAL DA SAÚDE, 2010).

Em sua construção inicial, o QBMI era composto por quatro domínios, os quais dividiam-se da seguinte forma: cinco itens pertencentes ao domínio “Preparo do ambiente”, sete no domínio “Preparo de Medicações”, 23 itens no domínio “Administração medicamentosa” e sete no “Cuidados após Administração Medicamentosa”.

Todos os especialistas que o validaram apresentavam amplo conhecimento sobre o tema, contemplando experiências em diferentes sítios de atuação (abrangendo ambiente acadêmico e assistencial).

O número total de especialistas esteve de acordo com a quantidade sugerida por Coluci, Alexandre e Milani (2015), que sugerem entre cinco e 10 especialistas para a validação por conteúdo de instrumentos construídos.

O convite a pessoas que não atuavam no estado de São Paulo ocorreu para que não atingisse potenciais participantes da coleta de dados da *survey*, visto que ele foi distribuído pelo Coren-SP, aos profissionais inscritos.

Após o processo de validação, o QBMI passou por pequenas modificações, uma vez que o IVC de todos os itens estavam acima de 0,78 e as sugestões foram

acolhidas tendo-se em foco o objetivo primário da elaboração do material educativo, ou seja, o controle de infecção associado à prática com medicações injetáveis.

Para tanto, deve-se considerar a inversão da pontuação dos seguintes componentes: DA4, DB1, DB2, DB4, DB7, DB8, DC3, DC4, DC5, DC6, DC7, DC9, DC12, DC13, DC14, DC16, DC18, DC22, DD1, DD3, DD4 e DD7.

Já para os componentes “DC10, DC19, DC20”, a inversão da pontuação deve-se sujeitar às recomendações do serviço em questão, uma vez que a antissepsia de pele para administração de injetável via SC e o uso de luvas para injetáveis via IM e SC devem ocorrer de acordo com as recomendações do serviço.

Neste sentido, reitera-se que DC10, DC19 e DC20 não foram invertidos quanto à desejabilidade da frequência, mantendo o mesmo padrão que os demais componentes, pois, não foi identificado consenso sobre as práticas (antissepsia de pele para administração de injetável SC, uso de luvas para injetáveis via IM e SC), segundo busca na literatura, uma vez que a maioria dos participantes deste estudo referiu a prática com certa frequência e não se buscou conhecer o contexto em que estas ações ocorriam, entende-se que há necessidade de maiores estudos para conhecer a influência destas condutas na prática (APÊNDICE F).

Além disso, visto a duplicação da mesma pergunta para os componentes DC3 e DC9, sugere-se a exclusão de DC9, ficando ao final, um instrumento com 44, conforme apêndice G, no qual encontram-se instruções de preenchimento deste questionário.

A elaboração da pontuação foi um facilitador na interpretação da prática referida sobre medicações injetáveis, em que, quanto mais próximo da pontuação mínima (44 pontos), melhor o desempenho.

Desta forma, o QBMI mostrou-se uma ferramenta abrangente, com potencialidade para mensurar o que se pretende, ou seja, identificar a prática referida de profissionais de enfermagem sobre práticas com injetáveis, uma vez que seu conteúdo se mostrou válido para a coleta de dados (COLUCI; ALEXANDRE; MILANI, 2015), tal qual foi sua contribuição para a análise contextual que subsidiou o curso “Boas Práticas com Injetáveis: ações para o controle de infecção”.

6.1.2 Prática Referida de Profissionais de Enfermagem com Medicações Injetáveis

De forma geral, as injeções inseguras (presentes em práticas como reuso de seringas ou agulhas) podem resultar, além de abcessos e reações tóxicas, em transmissão de diversos patógenos como fungos, vírus e bactérias, acarretando doenças como aids, hepatite B e C, a partir da exposição a contaminantes presentes de forma direta, como no dispositivo utilizado ou no frasco de algum medicamento aspirado (ORGANIZAÇÃO MUNDIAL DA SAÚDE, 2010).

Metanálise conduzida entre 2010 e 2017 focada no uso de medicações injetáveis em países de média e baixa renda, identificou que uma a cada 20 injeções é potencialmente insegura (ADEWUYI; AUTA, 2019).

De forma geral, 96,5% dos indivíduos reportaram utilização de insumos (seringa e agulha) estéreis, ou seja, removidos imediatamente do invólucro antes do procedimento, sendo uma prática menos comum em locais como Paquistão (86%), Comores (90,3%) e Afeganistão (90,9%), cujas prevalências de injeção insegura atingiram 14,0%, 9,7% e 9,1%, respectivamente (ADEWUYI; AUTA, 2019).

Segundo dados da América Latina e do Caribe, a maior prevalência de medicações injetáveis esteve relacionada ao maior poder aquisitivo (35,3%) e aqueles indivíduos com ao menos educação secundária (35,1%), da mesma forma que a maior prevalência ao acesso aos equipamentos estéreis também correspondia ao maior índice de riqueza (94,1% contra 95,2% daqueles com menor poder aquisitivo) e aqueles com ao menos educação secundária (97,4%) comparado a 94,7% (ADEWUYI; AUTA, 2019).

É importante salientar que, independentemente da função, tipo de serviço ou grau de instrução, as Boas Práticas com Medicações injetáveis devem ser contempladas por todos aqueles que executam a prática.

Considerando-se que as opções de resposta “sempre”, “quase sempre”, “às vezes” e “quase nunca” sugerem que a mesma ocorra com alguma frequência, ao contrário de “nunca” (KOSSOVER-SMITH *et al.*, 2017), foi realizada a somatória dessas respostas a fim de identificar a ocorrência ou não da referida prática para realizar a discussão do presente trabalho.

Embora a maioria dos participantes tenha referido a prática com injetáveis dentro das condutas seguras, encontrou-se componentes referidos por uma pequena parcela que demonstra relevância temática enquanto prática insegura, como ao reutilizar seringas para mais de um paciente, semelhante ao estudo desenvolvido por Kossover-Smith *et al*. (2017).

Além disso, ressalta-se que tanto profissionais da saúde quanto usuários do sistema de saúde e o ambiente, estão em risco frente a algumas práticas relatadas, mesmo que em menor frequência: reuso de insumos para a injeção, reprocessamento de seringas e agulhas não estéreis e HM inadequada.

Agregam-se às condutas inadequadas que podem ocasionar danos ao profissional, o reencape de agulhas, manipulação do perfurocortante já utilizado, caixa de descarte distante do local de sua utilização e HM deficiente, enquanto para a comunidade ou outros profissionais de saúde, descarte de perfurocortante realizado de forma insegura e o próprio reuso de seringas e agulhas (ORGANIZAÇÃO MUNDIAL DA SAÚDE, 2010).

Assim como sugerido por Filatro e Cairo (2015), a etapa inicial da elaboração de um material educativo deve contar com a análise contextual, a qual viabilizou a definição dos objetivos educacionais, após diagnóstico das necessidades de aprofundamento teórico, caracterização do público e o levantamento de potencialidades e restrições que possam emergir, corroborando com a TAS (MOREIRA, 2011b).

6.1.2.1 A Higienização das Mãos Durante a Prática com Medicações Injetáveis

Neste trabalho, os profissionais referiram alta frequência para HM, seja com água e sabão ou com solução alcoólica, contudo, não houve total inserção desta prática na rotina laboral.

Sobre a solução de escolha, é interessante ressaltar que na comparação estatística entre serviços, a HM com água e sabão antes da administração de medicação EV apresentou maior frequência de “sempre” nos serviços não hospitalares e de “quase sempre” nos hospitalares, enquanto a prática com solução alcoólica apresentou maior frequência de “às vezes” nos serviços não hospitalares e “sempre” e “quase sempre” nos hospitalares.

Sobre a relação entre uso de luvas e higienização das mãos, 98,92% profissionais afirmaram higienizar as mãos com alguma frequência antes e após o uso deste EPI, dado contrário a um estudo desenvolvido na Jordânia que identificou que o uso de luvas foi relacionado a uma menor frequência de HM no tocante à administração de injetáveis (AL-RAWAJFAH; TUBAISHAT, 2017).

A alta adesão referida à HM neste estudo, também, é contrária àquela encontrada em estudo observacional iraniano, cuja HM era precária, em setor emergencial (MUSAVI GHAHFAROKHI; MASOUDIYEKTA; KHAJEALI, 2019) e a encontrada por Van Toung *et al*. (2017) que identificou apenas 63,1% de adesão à esta prática antes da administração de injetável e 61,8% após.

Estudo desenvolvido na Jordânia demostrou que 73,9% das administrações de injetáveis foi realizada sem HM, sendo as injeções SC as que tiveram esta prática mais deficiente no momento pós procedimento (AL-RAWAJFAH; TUBAISHAT, 2017).

Para tanto, deve-se considerar, também, que estudos que avaliam a declaração do sujeito (abordagem utilizada para o presente trabalho) estão expostos à desejabilidade social, em que ele pode responder às questões conforme conduta socialmente aceita e não suas ações reais, o que pode sugerir uma menor adesão às práticas seguras.

Como exemplo cita-se um estudo observacional desenvolvido em ambiente cirúrgico, em que 41% dos profissionais higienizaram as mãos com solução alcoólica e apenas 19,4% após o procedimento (FODA; ELSHAER; SULTAN, 2018), ou seja, frequência muito inferior à encontrada em estudos referidos, como o realizado neste trabalho.

6.1.2.2 Preparo do Ambiente para Administração de Injetáveis

A maioria dos participantes da pesquisa afirmaram limpar superfície e bandeja para o preparo de medicamentos, em que, aqueles que referiram maior titulação enquanto nível médio, obtiveram maior frequência de “sempre” para esta prática.

O número encontrado neste trabalho foi substancialmente maior do que o encontrado por Mota *et al.* (2016), em que, apenas 28,4% limparam bancadas para realização de preparo de medicações.

Sobre os insumos utilizados para a administração medicamentosa, 95,68% referiram verificar a data de validade antes do uso com alguma frequência, enquanto 82,39% afirmaram nunca utilizar insumos cujas embalagens estivessem danificadas, apresentando maior frequência de “sempre” no nível médio, de “quase sempre” na graduação e de “quase nunca” na pós-graduação.

A proporção de pessoas que referiram verificar a validade das medicações e atentar-se para a integridade de embalagens foi muito maior do que a identificada em estudo observacional (MOTA *et al.*, 2016) em que apenas 10,3% verificaram integridade das embalagens.

Como práticas contrárias à segurança com injetáveis identificadas, foram citadas, pela literatura: presença de equipamentos para infusão EV e de seringas e agulhas fora da embalagem em salas cirúrgicas observadas (FODA; ELSHAER; SULTAN, 2018; VAN TUONG et al., 2017).

Em estudo desenvolvido por Van Tuong et al (2017), apesar da maioria dos profissionais terem passado por treinamento sobre injeção segura e reconhecer a disponibilidade de recursos materiais para uso, no serviço, houve relatos de profissionais que não checavam a integridade do invólucro para utilização dos insumos estéreis.

Ao observar todas as condutas referentes ao domínio "preparo do ambiente", verificou-se que houve correlação significativa entre idade (r=00,191; P<0,001) e tempo de atuação (r=00,563; P<0,001) demonstrando que quanto maior a idade e o tempo de atuação, menor o escore de preparo do ambiente e, consequentemente, melhor conduta, achados contrários ao do trabalho de Van Tuong *et al.* (2017) quando relacionou estes fatores à melhor conduta segura com injetáveis.

6.1.2.3 O Preparo das Medicações Injetáveis: uma Prática Asséptica

O compartilhamento de frascos de solução fisiológica para reconstituição de doses e utilização de frascos multidoses para dois pacientes ou mais foram práticas referidas por participantes da pesquisa.

Estas ações merecem atenção, visto que, embora não proibido, a utilização de frascos multidoses requer cuidados para prevenção e controle de infecções.

Condutas inadequadas sobre esta prática foram observadas no estudo de Kossover-Smith et al (2017) em que 24,1% profissionais afirmaram completar dose utilizando a mesma seringa do paciente para aspirar conteúdo do frasco multidose e 13,2% afirmaram utilizar frascos de solução salina intravenosa para diluir medicações de mais de um paciente, o que pode comprometer a esterilidade da solução e predispor a transmissão de patógenos pela via EV.

Para a limpeza do diafragma de frascos multidoses de medicação, 93,82% relataram realizar esta com alguma frequência, enquanto 27,03% afirmaram a utilização da mesma seringa para aspiração de medicamentos de frascos multidoses, apresentando maior frequência em relação ao trabalho de Kossover-Smith *et al.* (2017).

Já a desinfecção do septo antes da aspiração do conteúdo foi referida pela maioria dos profissionais, o que contrariou o achado de Van Tuong *et al.* (2017).

No tocante aos frascos e ampolas de medicação, 93,44% referiram realizar a limpeza com álcool a 70% com alguma frequência, antes da aspiração.

A frequência referida para desinfecção de frascos ou frascos ampolas demonstrou-se maior em relação a um estudo cearense, cujo objeto de estudo foi a injeção IM (MOTA *et al.*, 2016).

Sobre o uso de ampolas de vidro, pode haver contaminação da solução por micropartículas ou contaminações por metais durante a abertura das mesmas (que podem ser aspiradas e injetadas causando lesões decorrentes de embolias e reações a corpo estranho) ou causar acidente por perfurocortante ao profissional (expondo-o ao risco biológico decorrente da porta de entrada para bactérias e vírus) (CARRARETTO *et al.*, 2011).

Sistemas de abertura para o manuseio de ampolas foram desenvolvidos a fim de minimizar estes eventos. Para reduzir o risco potencial de fonte microbiológica para o paciente, deve-se proceder à desinfecção desta com álcool a 70% antes da abertura, gazes ou abridores para ampolas[2], e a abertura a partir do anel de abertura ou único ponto de abertura, mantendo-a inclinada a 45°, apoiando ambos os polegares no sentido oposto ao ponto de abertura e envolvendo a parte superior da ampola com o indicador, pressionando, posteriormente (CARRARETTO *et al.*, 2011).

Sobre a utilização da mesma agulha ou seringa para o preparo de medicações injetáveis, quando indagado sua ocorrência para o mesmo paciente, 20,23% afirmaram positivamente, em alguma frequência, já para pacientes diferentes, 3,09% fizeram esta referência, porcentagem maior do que aquela identificada por Van Tuong *et al.* (2017).

[2] Russell SH – Glass ampules – another approach. Anesth Analg, 1994;78:816 e Tiefenthaler W, Gimpl S, Wechselberger G et al. – Touch sensitivity with sterile standard surgical gloves and single-use protective gloves. Anaesthesia, 2006;61:959-961.

6.1.2.4 Administração de Injetáveis: os Riscos Presentes nos Detalhes

A realização da salinização intermitente foi referida por 93,13% dos participantes desta pesquisa, chamando a atenção a reutilização da mesma seringa para salinização dos cateteres periféricos de pacientes diferentes com alguma frequência referida por 3,47%.

A realização de *flushing* (infusão de solução salina ou SF 0,9% com a finalidade de limpar o cateter venoso) e *lock* (infusão de volume pré-estabelecido no período que o cateter não estiver sendo utilizado) para a manutenção da permeabilidade do CVP previnem sua obstrução, mal funcionamento, e potencial colonização para microrganismos, reduzindo o risco de infecção de corrente sanguínea (FERRONI et al, 2015).

Houve referência a reutilização da mesma seringa, com troca da agulha para administração de medicamento no mesmo paciente com alguma frequência, foi referida por 17,68%, enquanto para pacientes diferentes, atingiu 1,85%.

Os relatos sobre reutilização destes insumos diferem do encontrado em estudo desenvolvido em ambiente cirúrgico (FODA; ELSHAER; SULTAN, 2018). E se assemelha ao encontrado em estudo de Kossover-Smith et al (2017) em que 3% dos enfermeiros participantes da pesquisa confirmaram o reuso de seringas para mais de um paciente.

De forma geral, ações como o reuso de seringas ou agulhas podem resultar, além de abcessos e reações tóxicas, em transmissão de diversos patógenos como fungos, vírus e bactérias, causando doenças como aids, hepatite B e C, a partir da exposição a contaminantes presentes de forma direta, como no dispositivo utilizado ou no frasco de algum medicamento aspirado (ORGANIZAÇÃO MUNDIAL DA SAÚDE, 2010).

Ações referentes a manutenção da funcionalidade e prevenção de infecção e agravos decorrentes da obstrução e deposição de resíduos medicamentosos, bem como coágulos, também foi abordada pelo QBMI.

A realização de *flushing* e *lock* podem ser medidas importantes para a prevenção destas ocorrências.

Revisão integrativa (GOOSSENS, 2015) condensou recomendações pertinentes ao *flushing* e *lock* de acessos venosos periféricos, dentre os quais, citam-

se: não exercer força no êmbolo contra resistência da seringa, visto risco de lesionar o cateter, preferindo-se técnica de fluxo pulsátil, antes e após cada administração medicamentosa (impede a fixação de depósito da substância/fibrina e após porque previne o acúmulo destas no lúmen prevenindo a ligação de microrganismos na sua parede interna.

Sobre a heparina, é desencorajado seu para este fim, visto que, embora a probabilidade de indução à hemorragia iatrogênica seja rara, pode haver erro na dose, induzir trombocitopenia a partir da hipersensibilidade e estímulo à formação de biofilme pelo *S. aureus*, contaminação pelo manuseio de frasco multidose e incompatibilidade com outras drogas (GOOSSENS, 2015; SHANKS et al., 2006).

Trabalho que buscou identificar a taxa de incidência e o uso de *flushing* na prevenção das obstruções de CVP em enfermaria de uma clínica médica em Portugal (BRAGA *et al.*, 2018), a partir de entrevista com 22 enfermeiros, análise documental e observação participante (110 pacientes com AVP) até alta médica/óbito/transferência), identificou uma incidência cumulativa de 50% de obstrução do cateter venoso e a maioria ocorreu nas primeiras 48 horas (66%).

A realização do *flushing* (entre 3 e 10 ml de SF 0,9%) no trabalho supracitado, tinha por barreira: tempo para realização do cuidado, grau de dependência dos pacientes e o excesso de trabalho de forma que não se estabeleça uma padronização quanto a este cuidado. Os autores indicam ainda a indisponibilidade de informações como volume do *flushing* a ser administrado, em norma disponível naquele serviço.

A reutilização de oclusores de cateteres venosos periféricos atingiu 50,89% das respostas referidas desta pesquisa, enquanto o armazenamento destes, com alguma frequência, em mesas de cabeceira, bolsos de jaleco/avental ou outros locais para posterior reutilização foi afirmado por 28,19% dos profissionais que participaram desta pesquisa.

Já a reutilização de oclusores de equipo ou dupla via, em alguma frequência, alcançou 32,36%. A desinfecção dos oclusores com solução a base de álcool antes da infusão de medicamento foi relatada por 86,49%.

Sobre conectores, estudo desenvolvido no interior de Minas Gerais (Brasil), considerou uma medida adequada o armazenamento de conectores a separação destes por paciente, mantidos em recipiente seco e tampado, pois, a instituição em questão não havia adotado a recomendação da Anvisa até aquele momento (coleta

de dados entre setembro e dezembro de 2017), e ainda assim, identificou que 30,6% destes haviam sido armazenados em local inapropriado, como “pregadas” no equipo/suporte de frasco de solução venosa, bolso do avental do profissional ou mesmo na mesa de cabeceira do paciente, dividindo o recipiente com conectores de diferentes pacientes (ALVES *et al.,* 2019).

No presente estudo, sobre a antissepsia de pele com álcool a 70%, 96,76% afirmaram esta prática com alguma frequência para administração de injetáveis via SC enquanto 98,84% o fizeram para via IM.

Após a antissepsia, 66,1% dos profissionais negaram tocar a área que realizaram antissepsia.

Estudo observacional com injetáveis identificou que 91,1% das injeções foram administradas após desinfecção cutânea (VAN TUONG *et al*, 2017).

Estudo desenvolvido em ambiente cirúrgico identificou que a antissepsia de pele ocorreu em cerca da metade dos casos de punção observadas (FODA; ELSHAER; SULTAN, 2018).

Sobre a antissepsia da pele com álcool a 70% para administração de medicações IM (exceto vacinas) observou-se maior frequência de “quase sempre” para aqueles com até 10 anos de atividade laboral e de “às vezes” e “quase nunca” para aqueles com mais de 10 anos.

A antissepsia da pele do paciente com álcool a 70% para administração de medicações SC (exceto vacinas), assim como a realização de lavagem do acesso venoso periférico intermitente com SF 0,9% após administração de medicamentos e de curativo estéril para fixação/manutenção dos cateteres venosos periféricos apresentaram maior frequência de “nunca” nos serviços não hospitalares e de “sempre” nos hospitalares.

Sobre a antissepsia de pele para injeção SC, revisão da literatura abordou técnica e insumos necessários para a realização da mesma, contudo, não havia homogeneidade sobre esta necessidade: enquanto alguns referenciavam este passo, outros não o faziam (REICHEMBACH; MEIER; ASCHIDAMINI, 2005).

A antissepsia de pele é estratégia reconhecida por reduzir a microbiota local a partir de agentes germicidas, sendo o álcool a 70% a solução mais utilizada e recomendada para tal procedimento. Contudo, não elimina toda a microbiota existente e a partir da perfuração da pele por uma agulha há a possibilidade de microrganismos

da camada mais profunda da pele deslocar-se pelos tecidos adjacentes (FERREIRA *et al*., 2009).

Estudos realizados, incluindo um com inoculação de *Staphylococcus aureus* que descreviam desde a quantidade de microrganismos necessários para a ocorrência de infecção até a não relação entre infecção e antissepsia de pele, afirma que há necessidade de ensaios clínicos randomizados de boa qualidade nesta área do conhecimento, sendo um tema controverso (FERREIRA *et al*., 2009).

Quanto ao uso de luvas: 91,2% afirmaram utilizar este EPI para administração de medicações intramusculares, enquanto 87,57% fizeram a mesma afirmativa para SC e 97,07% para EV.

Indagados sobre o uso de luvas apenas para pacientes sabidamente com HIV, HCV ou HBV, 87,95% referiram a prática com alguma frequência.

Sobre o uso de luvas para administração de medicações intramusculares obteve-se maior frequência de “sempre” para o nível médio e graduação e de “nunca” para pós-graduação, para SC houve maior frequência de “sempre” no nível médio e graduação e de “quase nunca” e “nunca” na pós-graduação, enquanto para administração de medicamentos por via EV apenas para paciente sabidamente acometido com vírus como HIV, hepatites B ou C obteve maior frequência de “sempre” no nível médio e de “nunca” para graduação e pós-graduação.

Dentre os EPIs para a prática com injetáveis, o uso de máscara, proteção ocular e avental não se faz necessário, a menos que haja probabilidade de respingo de sangue (ORGANIZAÇÃO MUNDIAL DA SAÚDE, 2010).

As luvas têm seu uso orientado para redução da contaminação nas mãos dos profissionais da saúde por sangue ou fluidos corporais.

Sugere-se o uso, ainda, em casos de precaução por contato e em situais de surto devendo ser associado a HM, para garantir total proteção às mãos do profissional, já que se pode entrar em contato com microrganismos ou até mesmo contaminar-se durante a remoção deste equipamento (ORGANIZAÇÃO MUNDIAL DA SAÚDE, 2009).

A decisão pelo seu uso requer avaliação do risco de contaminação a partir do procedimento a ser realizado (ORGANIZAÇÃO MUNDIAL DA SAÚDE, 2009).

A alta referência ao uso de luvas para a prática de injeções EV é substancialmente maior do que a identificada em estudo observacional do Vietnã (VAN TUONG *et al*, 2017).

Estudo desenvolvido em unidade pediátrica, que considerou o uso de luvas pertinente diante de medicações intramusculares em pediatria, identificou que houve baixa adesão a este EPI, no momento da administração medicamentosa (SOUZA *et al.*, 2018).

Ao comparar (a partir da diferença significativa) o tempo de atuação profissional, tanto o uso de luvas para administração medicamentosa via intramuscular quanto SC obtiveram maior frequência de “sempre” no grupo com atuação até 10 anos e de “às vezes”, “quase nunca” e “nunca” para o de mais de 10 anos, enquanto para a via EV, atingiu maior frequência de “sempre” naquele com até 10 anos e de “às vezes” no grupo com mais de 10 anos.

O uso de luvas para administração de medicamentos IM e SC obteve maior frequência de “quase nunca” e “nunca” nos serviços não hospitalares e de “sempre” nos hospitalares.

Já para administrar medicamentos por via EV evidenciou-se para a frequência de “sempre” nos serviços não hospitalares e de “quase sempre” e “quase nunca” nos hospitalares.

A adesão referida às luvas para realização de medicação EV mostrou-se maior do que o encontrado, observacionalmente, em ambiente cirúrgico (FODA; ELSHAER; SULTAN, 2018).

Este EPI é importante como método de barreira para prevenção de acidentes com fluido corporal ou sangue, contudo, ainda há, na literatura, apontamentos para a não adesão deste no momento do acidente (NOUETCHOGNOU *et al.*, 2016).

Estudo iraniano identificou que, no setor emergencial, a adesão às medidas de segurança alcançou 63,3% para injeção intramuscular e 86,7% para injeções intravenosas, sem diferença estatística na comparação entre idade, gênero, grau de instrução e experiência de trabalho para as adesões (MUSAVI GHAHFAROKHI; MASOUDIYEKTA; KHAJEALI, 2019).

Já com relação a injeção intramuscular, a partir da observação direta (327 observações), foi identificado que não houve uso de luvas em nenhuma delas em ambiente pediátrico (MOTA *et al.*, 2016).

É sabido que a adesão às luvas depende do risco de contato com sangue e fluidos corporais, sendo obrigatório para a realização de injeções EV e ficando a critério de protocolos quanto às injeções SC ou IM, dependendo da avaliação crítica

do profissional (CONSELHO REGIONAL DE ENFERMAGEM DE SÃO PAULO, 2010; SECRETARIA DE ESTADO DA SAÚDE, 2016).

Em caso de pacientes em precaução por contato, o uso de luvas sempre se fará necessário. A presença ou não de doenças transmissíveis por sangue ou outros fluidos não interferem na decisão do uso deste EPI.

Contudo, deve-se considerar que a não adesão a este EPI pode viabilizar a transmissão destas para o profissional.

Ressalta-se que o uso de luvas não é associado à prevenção de transmissão cruzada de microrganismos.

Seu uso é correto quando há troca de luvas precedendo e sucedendo a higienização das mãos, quando o EPI é descartado após o procedimento e não é reutilizado ou reprocessado, e para manutenção de sua integridade, sugere-se evitar utilização de cremes e loções a base de petrolatos (ORGANIZAÇÃO MUNDIAL DA SAÚDE, 2009).

Segundo boletim epidemiológico de 2018 (MINISTÉRIO DA SAÚDE, 2018) a hepatite C foi responsável pela maior parte das mortes em decorrência de hepatites virais no Brasil (75,8%) seguida da hepatite B (21,4%) entre 2000 e 2016.

A hepatite C foi ainda a terceira maior causa de transplante hepático, entre 1999 e 2017, de 587.821 casos confirmados de hepatites virais, 218.257 (37,1%) eram hepatite B enquanto 200.839 (34,2%) correspondia a hepatite C, em que a região sudeste se relacionava a 35,2% de hepatite B e 60,9% de hepatite C.

Sobre o HIV, em 2018, foram diagnosticados 43.941 novos casos e 37.161 casos de aids, e, entre 2000 e 2019, 125.144 gestantes foram notificadas com HIV (BRASIL, 2019).

Paralelamente a isto, estudo indiano realizado com graduandos de enfermagem que avaliava o conhecimento e conduta frente à hepatite B identificou que 13,7% destes reconheciam o uso de método de barreira como estratégia preventiva à doença, enquanto 29,5% o faziam quanto ao uso de precaução padrão. Contudo, 79,8% dos estudantes relataram utilizar luvas antes do manuseio do paciente (CHHABRA *et al.*, 2019).

Ressalta-se que mesmo quando ocorre exposição a pouca quantidade de sangue, há probabilidade de transmissão de doenças, e, conforme revisão integrativa, o risco de transmissão de vírus pela corrente sanguínea é maior para hepatite B, seguido pela C e HIV: a cada 1000 acidentes com pacientes infectados, 300

profissionais se infectarão pelo HBV, enquanto para HCV a soroconversão é 30 para 1000 e HIV 30 para 1000 (COOKE; STEPHENS, 2017).

Sobre curativo estéril de CVP e troca deste antes da presença de sinais flebite, a maioria dos participantes referiram estas práticas, a última com maior frequência de “sempre” para aqueles atuantes em instituições hospitalares.

Estudo transversal, ao observar curativos de acessos venosos periféricos, identificou que 35,5% encontravam-se molhados e 53% com sujidade (sangue/medicação) no curativo e no extensor 0 dupla via (ALVES *et al.*, 2019).

Ressalta-se que estudo brasileiro que avaliou sinais de flebite em pacientes com AVP identificou que os sinais deste evento, geralmente, foram observados ao redor do curativo realizado com fita microporosa ou esparadrapo (ALVES *et al*., 2019).

Complicações decorrentes do uso de CVP não ocorrem exclusivamente em ambiente hospitalar, como observado na atenção domiciliar, no Japão, em que 15 de 20 pacientes cuidados pela enfermagem domiciliária apresentaram complicações, dentre as quais, infiltração SC, obstrução do cateter e perda do dispositivo, complicações não correlacionadas com idade ou tempo de permanência para a incidência de complicações (NAKAYAMA *et al.*, 2018).

Ao comparar, estatisticamente, os escores de “administração medicamentosa” bem como o escore total do instrumento, observou-se que foram menores nos serviços hospitalares, em relação ao comparativo com “outros serviços”.

Sobre a correlação entre variáveis numéricas verificou-se que houve correlação significativa de forma que, quanto maior a idade e menor o tempo de atuação, menor o escore referente à administração de injetáveis e, consequentemente, melhor conduta.

É importante salientar que fatores como qualificação profissional e quantidade de injeções administradas pelo profissional não demonstraram estar associadas com melhores práticas com injetáveis, segundo a literatura (VAN TUONG *et al.*, 2017).

6.1.2.5 Profissional de Enfermagem e o Cuidado com sua Segurança ao Manipular Perfurocortantes

Quando indagados sobre treinamento para manipulação de agulhas e cateteres com dispositivos de segurança, 79,69% afirmaram participar com alguma

frequência, apresentando maiores frequências de “nunca” nos serviços não hospitalares e de “quase sempre” e “sempre” nos hospitalares.

Comparativamente, entre os graus de instrução, observou-se diferença significante com maior frequência de “sempre” para o nível médio, de “nunca” para graduação e de “quase nunca” na pós-graduação.

São dispositivos para a segurança durante a manipulação de agulhas hipodérmicas: seringas com sistema manual retrátil (puxam a agulha para dentro do corpo da seringa após sua utilização) e agulha de segurança que possui capa rígida integrada para protege-la após sua utilização, e, embora possam encarecer o preço da agulha, o uso de agulhas com estes dispositivos demonstrou valor econômico ao reduzir acidentes e também, a partir do achado de que os acidentes podem acarretar prejuízos à saúde mental dos profissionais (COOKE; STEPHENS, 2017).

O treinamento é um fator associado a melhor aquisição de conhecimento, o que tende a corresponder de forma positiva com a prática segura com injetáveis ($p > 0,05$), e pode reduzir os acidentes com perfurocortantes ($X2 = 6,39$; $p <0,05$) (VAN TUONG *et al.*, 2017).

Sobre o reencape de agulhas (em que 28,73% afirmaram reencapar agulhas com alguma frequência) houve diferença estatística na frequência de “nunca” no nível médio, de “às vezes” na graduação e de “quase nunca” na pós-graduação.

O reencape de agulhas é uma prática presente no ambiente laboral de profissionais de enfermagem. Contudo, há estudos que relatam esta prática em menor escala, como estudo observacional que identificou o descarte após uso de quase a totalidade dos perfurocortantes utilizados, e quase 10% realizou a prática com apenas uma mão (FODA; ELSHAER; SULTAN, 2018).

Destaca-se que o uso de dispositivos de segurança é eficaz na prevenção de acidentes com perfurocortantes, como visto em revisão integrativa, que sugere que o uso de dispositivos de segurança que cobre a agulha, após o procedimento, diminui o risco dos acidentes com perfurocortantes pelos profissionais da saúde entre 43,4% e 100% quando comparados aos dispositivos convencionais (COOKE; STEPHENS, 2017).

Na comparação para avaliação de diferença estatística entre os tempos de trabalho, “Com que frequência costuma informar-se sobre o fluxo de atendimento pós-exposição percutânea com material perfurocortante no seu local de trabalho?” apresentou maior frequência de “nunca” no grupo com até 10 anos de atuação e

“sempre” naquele com mais de 10 anos, enquanto os cuidados inapropriados com lesão decorrente de acidente ocupacional com perfurocortante, apresentou maior frequência de “nunca” para até 10 anos na área e de “quase nunca”, “quase sempre” e “sempre”, quando mais de 10 anos.

O Brasil se encontra entre os países que adotam legislação para acidentes com perfurocortantes, ao passo que estudo brasileiro identificou, dentre as justificativas para a adoção mais lenta da adesão a estes dispositivos as lacunas de monitoramento da adoção desta prática (COOKE; STEPHENS, 2017).

Já sobre o transporte de materiais como agulhas e seringas usadas realizado de forma desprotegida até o local de descarte foi referido por 61,39%. Comparativamente, observou-se maior frequência de “sempre” nos grupos referentes ao nível médio e graduação e de “quase nunca” na pós-graduação.

O transporte do material contaminado é muito importante, pois, quanto mais próxima do local de execução da prática, menor a probabilidade de acidente com perfurocortante.

Estudo nigeriano identificou que 23,4% dos acidentes relatados tinha o momento do descarte como de ocorrência, e 32,1% dos dispositivos já haviam sido utilizados no paciente (OLUWATOSIN; OLADAPO; ASUZU, 2016).

Estudo indiano (SRIRAM, 2019) que teve por objeto acidentes com perfurocortantes entre profissionais de saúde de um hospital de ensino, em diferentes setores, identificou que entre 1525 funcionários (médicos e profissionais de enfermagem) 10,81% dos profissionais haviam sido expostos a acidentes com perfurocortante, a maioria havia ocorrido em enfermaria, tendo sido causados por agulhas hipodérmicas, 40% durante o procedimento de medicações injetáveis e em 98% dos casos houve perfuração cutânea, contudo, apenas 82% dos profissionais procuraram tratamento após o acidente (65% utilizavam luvas).

Estudo desenvolvido em Youndé, capital dos Camarões (NOUETCHOGNOU *et al.*, 2016) identificou a partir de questionário auto-aplicado, que a maioria dos profissionais reconheciam a possibilidade de transmissão sanguínea do vírus HIV e HBV, contudo, 11,33% desconsideraram a vacinação de prestadores de cuidado como medida preventiva à transmissão de patógenos por corrente sanguínea frente exposição ocupacional a sangue e fluidos corpóreos (apenas 33,67% tinham o esquema vacinal completo, contra hepatite B).

A preocupação em adquirir informações sobre sua situação vacinal contra doenças potencialmente passíveis de transmissão ocupacional, como a hepatite B foi referida por 95,52%.

Ao considerar como adequado que o profissional realize a busca de informações vacinais contra hepatite B e fluxo de atendimento após acidente com perfurocortante, buscou-se identificar não apenas o quanto os profissionais buscam esta atualização, mas também, de forma indireta, a sua ciência quanto a disponibilidade e eficácia da vacina, rotineiramente solicitada em processo de contratação de profissionais da saúde e existência de medidas disponíveis em protocolos institucionais de atendimento à vítima de acidente ocupacional, o que foi demonstrado positivamente, uma vez que a maioria dos participantes apontou alguma frequência para esta conduta.

Outro estudo desenvolvido na Índia (CHHABRA *et al.*, 2019) que contou com 183 enfermeiros dentre os participantes da pesquisa e buscou avaliar conhecimento e atitude sobre hepatite B em uma instituição de saúde, relatou que a vacina contra hepatite B passou a ser obrigatória em novembro de 2018 e encontrou após questionário auto-aplicado em coleta de dados entre julho e outubro de 2018 que menos da metade os profissionais de enfermagem possuíam esquema vacinal completo ou tinham conhecimento sobre a doença e reconheciam a vacinação como melhor método profilático contra a hepatite B (86,9% destes eram cientes da disponibilidade da vacina contra hepatite B), contudo, a maioria sabia da obrigatoriedade da notificação compulsória relativa à doença.

Sobre o cuidado com lesões em decorrência de acidente com perfurocortante, 59,61% afirmaram tomar conhecimento de que pessoas da equipe de enfermagem após sofrer acidente com perfurocortante espremem a área afetada e colocam álcool a 70% para desinfetar a região, em que houve maior frequência de “nunca” para até 10 anos na área e de “quase nunca”, “quase sempre” e “sempre”, quando mais de 10 anos.

O cuidado inadequado com lesões decorrentes de acidentes com perfurocortante está presente na literatura. Estudo com estudantes de enfermagem de Uttarakhand (Índia) coletou 26,2% relatos de sujeitos que afirmaram ter sofrido ou testemunhado o acidente com perfurocortante, em que, embora a maioria tenha seguido ao protocolo de lavar a área com água e sabão e relatar o ocorrido à instituição, apenas 62,4% não espremeram o ferimento (CHHABRA *et al.*, 2019),

semelhante a 55,6% de enfermeiros de Kerala (Índia), que realizaram a limpeza do local da lesão com água e sabão (MADHAVAN *et al.*, 2019).

Estudo iraniano identificou que o cuidado com a área da lesão após acidente com perfurocortante englobou água, sabão e antisséptico (SOBATI; MASOUDI, 2017). Já um trabalho de Camarões, identificou que 80% limparam a lesão com água corrente e 74,5% aplicaram solução antisséptica (NOUETCHOGNOU *et al.*, 2016).

É preciso avaliar lesões nas mãos para prevenir acidentes que possam ocasionar respingos sobre a área lesionada, como observado no estudo de Nouetchognou (2016), em que 41% de acidentes com sangue e fluidos corporais envolveram respingo de sangue em pele lesionada.

Salienta-se que o tamanho da lesão não deve ser fator associado à realização de notificação pós-exposição, conforme acreditava 43,6% de enfermeiros de um estudo que comparava as atitudes frente a acidentes perfurocortantes (MADHAVAN *et al.*, 2019), uma vez que, ao contrário de algumas doenças cuja transmissão por corrente sanguínea depende do patógeno, volume e tipo de exposição sanguínea, patógenos como HBV, HCV, HIV podem ser transmitidos mesmo quando há ausência de contaminação visível por sangue (ORGANIZAÇÃO MUNDIAL DA SAÚDE, 2010).

A ausência de sistema de notificação e o desconhecimento do procedimento de notificação são possíveis justificativas para a não notificação de acidentes com perfurocortantes (FODA; ELSHAER; SULTAN, 2018).

Salienta-se que o mesmo estudo supracitado que identificou estes fatores, em entrevista com chefia da unidade de controle de infecção, os autores (FODA; ELSHAER; SULTAN, 2018) relatam que foi salientado a existência de protocolos sobre a prática com injetáveis, treinamento para manuseio de resíduos e protocolo pós-exposição ocupacional.

Complementarmente aos fatores para a não notificação de acidentes com perfurocortantes por parte do profissional acometido, citam-se: acidente com material estéril ou limpo, sobrecarga de trabalho, hepatites B e C, pelo paciente fonte (SOBATI; MASOUDI, 2017), protocolos complicados ou pouco claros, a crença de que o evento pode refletir negativamente em sua prática-padrão (COOKE; STEPHENS, 2017), paciente fonte não tinha doenças transmissíveis (ALAY *et al.*, 2017).

A exposição aos perfurocortantes foram responsáveis por 37,39% dos casos de hepatites B e C em trabalhadores da saúde, no mundo, enquanto o CDC estima o gasto (entre profilaxia pós-exposição e tratamento a longo e curto prazo para infecções transmitidas pela corrente sanguínea, absenteísmo, indenização e processos judiciais) entre US$71 e US$5,000 (COOKE; STEPHENS, 2017), o que sugere a importância necessária frente aos eventos que podem envolver perfurocortantes como protagonistas de acidentes ocupacionais entre profissionais de saúde.

Revisão integrativa destaca que a ocorrência deste tipo de acidente pode desencadear prejuízos mentais, bem como estresse pós-traumático (COOKE; STEPHENS, 2017).

Estudo identificou que o reencape de agulhas esteve presente em 29% dos acidentes envolvendo exposição percutânea, enquanto aqueles da área da enfermagem apresentaram melhores atitudes para o cuidado com as lesões. Para esta parcela do estudo, a administração de injetáveis (EV, IM e SC) esteve dentre as atividades mais predisponentes ao acidente – 26% (MADHAVAN *et al.*, 2019).

Dentre as estratégias para equipamentos de gerenciamento de resíduos, estão as seringas descartáveis, o uso de seringas com dispositivo de prevenção ao reuso e de proteção de lesão por perfurocortante (MORISSON *et al.*, 2016).

Já achados referentes aos acidentes com perfurocortantes em ambiente cirúrgico de Alexandria, apontaram que entre 61,3% relatos de acidentes com perfurocortantes ocorreu com a maioria destes já utilizadas (80%), e menos da metade estavam utilizando luvas, sendo quase 30% relacionado ao momento do descarte, chamando a atenção para a reincidência do evento para com o mesmo indivíduo (FODA; ELSHAER; SULTAN, 2018).

São destacados como fatores modificáveis para a ocorrência de acidentes com perfurocortantes: sobrecarga de trabalho e os procedimentos realizados de forma inadequada, como o reencape de agulhas para o descarte com perfurocortantes (COOKE; STEPHENS, 2017).

É preciso considerar que conhecimento sobre determinado tema, como é o caso de injeção segura, pode não configurar uma prática dentro destes padrões, conforme identificado no estudo de Van Tuong *et al*. (2017) e de Alay *et al.* (2017), em que, segundo dados coletados pelos prontuários dos profissionais, 78% utilizavam luvas, enquanto durante entrevista, esse número aumentou para 98%.

De qualquer forma, reitera-se que embora estudos observacionais sejam mais fidedignos do que aqueles que contam com autorrelato estes são mais factíveis para a aquisição de respostas de muitos profissionais, mesmo não sendo considerado o padrão-ouro, o que enseja cuidado para realização de maiores inferências frente ao tema.

Outro ponto importante é o reconhecimento de que treinamentos, atualizações e sensibilização do profissional da saúde que estejam sob risco deste tipo de acidente e que realizam a manipulação de injetáveis, é uma ferramenta que muitos estudos sugerem para prevenir estes tipos de agravos e tornar estes procedimentos mais seguros para todos os atores envolvidos (KOSSOVER-SMITH *et al.*, 2017; NOUETCHOGNOU *et al.*, 2016).

Além dos pontos aqui apresentados, deve-se considerar que a adesão às Boas Práticas enseja não apenas esforços do profissional, mas também a disponibilidade de recursos materiais na instituição (ESTEQUI *et al.*, 2020) ou ainda à logística desta para oportunizar a pronta entrega e utilização de insumos como itens para a realização de *flushing.*

Estudo realizado no interior paulista relatou que a dispensação de medicamentos via EV era disponibilizada em *kits*, os quais não contavam com os insumos necessários para a realização de *flushing* (requerendo solicitação manual do profissional) e, relacionaram que este fator poderia estar associado a uma baixa adesão desta prática (FERREIRA *et al.*, 2021).

Sobre a utilização do QBMI, neste estudo, o instrumento mostrou-se confiável quando em sua totalidade (α=0,790). Sugere-se sua utilização em diferentes cenários a fim de identificar como ele se configura em outros cenários.

6.2 CURSO “BOAS PRÁTICAS COM INJETÁVEIS: AÇÕES PARA O CONTROLE DE INFECÇÃO”: DO DESENVOLVIMENTO À IMPLEMENTAÇÃO

As tecnologias digitais da informação e comunicação são estratégias potencializadoras da aprendizagem no desenvolvimento de ambientes de aprendizagem (WANDERLEY *et al.*, 2018).

O AVA é considerado uma das TIC mais utilizadas no ensino em enfermagem (NORONHA, 2018), seja em ambientes acadêmicos (CASTILHO *et al.*, 2020; PARULLA *et al.*, 2020), principalmente enquanto apoio a disciplinas

semipresenciais, ou como educação continuada (FRUTUOSO et al., 2019) tem-se configurado de forma positiva, na área da saúde, uma vez que permite a autonomia do sujeito, uma relação dialógica e o aprofundamento de temas (FRUTUOSO et al., 2019).

Para tanto, o PoCA (UNIVERSIDADE FEDERAL DE SÃO CARLOS, c2020) mostrou-se uma ferramenta viável, por possibilitar que a população-alvo pudesse ter acesso ao conteúdo de forma gratuita, além de aumentar a visibilidade ao curso.

Como ferramenta de disseminação de conteúdo aplicada ao AVA, a utilização de plataformas de cursos abertos de alcance massivo são estratégias úteis para a acessibilidade de conteúdos de ensino que demandam conexão à *internet*, e contemplam recursos que variam desde textos para leitura, em formato PDF, simulações, tutoriais, animações, *podcasts* e vídeos e referências para leitura e que são oferecidos de forma gratuita ou com baixo custo, visando à autonomia do sujeito, tendo por vantagem, ser utilizados para atualização profissional ou mesmo como complemento às atividades em sala de aula (PARULLA *et al.*, 2020; SWIGART; LIANG, 2016).

Em sua revisão integrativa, Silveira e Cogo (2017) identificaram enquanto tecnologias educativas na enfermagem, entre os anos 2006 e 2015, cursos online abordando administração medicamentosa, AVA sobre punção venosa e hipertextos abordando tanto punção venoso quanto habilidades de enfermagem em geral.

Em geral, cursos focando a administração de medicações ou a punção venosa abordam alunos de graduação em enfermagem (FROTA *et al.*, 2015; GADIOLI *et al.*, 2018; LEE; QUINN, 2019; METTIÄINEN *et al.*, 2014) receberam avaliação satisfatória enquanto estratégias educativas, seja pelo corpo de juízes, seja pela população-alvo, ou mesmo descrição de etapas de construção destas ferramentas, pelas autoras.

A literatura aponta para estratégias educativas pautadas na aprendizagem significativa, e, o quanto deve-se contextualizar a elaboração destes às necessidades educacionais presentes. Como exemplo cita-se curso a distância sobre Suporte Básico de Vida para acadêmicos e profissionais de enfermagem, o qual contemplou a construção e validação de um jogo educativo simulado em suporte básico de vida (COSTA *et al.*, 2018).

Para tanto, considerar as diretrizes de Filatro e Cairo (2015) a fim de produzir material educativo com qualidade comunicacional visando a elaboração do

presente curso, possibilitou contextualizar etapas importantes e que consideram o referencial teórico escolhido (TAS), visto que as etapas também contemplam uma análise inicial da população-alvo sobre o conteúdo e fluência digital, visando um material com linguagem acessível, clara e dialogada promovendo reflexões e novos significados sobre o tema.

6.2.1 O Processo de Validação e Revisão do Material Educativo do Curso "Boas Práticas com Injetáveis: Ações para o Controle de Infecção"

AS avaliadoras, além de possuírem conhecimento acerca do conteúdo relevante para as Boas Práticas com Injetáveis, possuía variedade no que concerne ao campo de atuação, sendo possível observar a concordância entre diferentes atores: especialistas, profissionais assistências, docentes e discente.

O *ebook* mostrou-se adequado segundo as avaliadoras, sendo considerado totalmente adequado (de acordo com o IVCES) pela maioria destas.

Ao disponibilizar parte do conteúdo em formato de texto para mídia impressa, pode-se garantir uma maior mobilidade e flexibilidade deste conteúdo, que pode ser baixado para guarda em formato PDF (versão portável) ou impresso, ou seja, pode ser utilizado em diversos ambientes, podendo o aluno aprofundar-se em tópicos de seu interesse a partir da leitura das referências (FILATRO; CAIRO, 2015).

Sobre os objetivos, as melhorias visaram adequar o material enquanto ferramenta para reflexão sobre o tema (80% de concordância) e para incentivar a mudança de comportamento (90%). Para tal, foram inseridos exemplos contextualizando as Boas Práticas com Injetáveis e a rotina com esta prática e inserção de imagem.

As imagens são consideradas os primeiros elementos a chamar a atenção em um conteúdo educacional, por serem percebidas anteriormente ao texto e pela semelhança com aquilo que designam, diferentemente do texto, cujas palavras podem não ter semelhança com aquilo que designa, sendo mais facilmente lembradas, tornando a comunicação mais efetiva, ao passo que fotografias intencionam reproduzir imagens reais (FILATRO; CAIRO, 2015).

Já as melhorias enquanto estrutura, conforme resultados do IVCES, as mudanças visaram tornar a linguagem mais interativa para permitir o envolvimento ativo no processo educativo (80% de concordância) e reestruturar de forma que a

sequência de assuntos se tornasse lógica (90% de concordância) com informações necessárias (90%).

A fim de melhorar o material enquanto estrutura, foram inseridos quadro informativo explicando termo utilizado e sugestão de leitura, bem como outros elementos pertinentes para a prática, padronização da linguagem utilizada, fazendo-se uso de mais questionamentos no decorrer do texto, e modificação textual de seção, tornando a sequência mais lógica.

Para Filatro e Cairo (2015), o desenvolvimento de conteúdo educacional com recursos midiáticos assemelha-se à simulação de diálogo didático real adaptado a partir da comunicação assíncrona entre docentes e estudantes, e as autoras consideram, ainda, que a produção de conteúdo educacionais possui um consenso referente às características para garantia de sua qualidade.

Possibilitar um diálogo didático é, assim como observado por Filatro e Cairo (2015) e Moreira (2011) garantir que a aprendizagem ocorra efetivamente, pois, é possibilitar a interação entre o conteúdo aprendido e o aluno.

Sobre o processo de elaboração e validação do *podcast* "Boas Práticas com Medicações Injetáveis: subsídios para o autocuidado", este também se apresentou de forma adequada, conforme resultado do IVCES, porém, sugestões das avaliadoras foram consideradas a fim de melhorar esta ferramenta.

O uso de *podcasts*, passível de acesso via *internet*, pode ter seu conteúdo baixado ou executado via navegador *web* e podem ser executados conforme ritmo de aprendizado do usuário, podendo ser ouvido ao mesmo tempo em que ele realiza outras atividades (FILTATRO; CAIRO, 2015).

No tocante às questões para avaliação e disparadoras de discussão, estas também foram validadas e, embora o IVC tenha sido adequada, foi realizado pequenas mudanças nos mesmos.

Destaca-se aqui a importância em não utilizar mesmos exemplos nas questões avaliadoras a fim de permitir ao aluno refletir sobre o conteúdo que ele processou e não apenas memorizar, tal como ocorre na aprendizagem exclusivamente mecânica (MOREIRA, 2011a).

Como tanto textos, quanto imagens e sons, são originados a partir de esforços humanos, os quais são protegidos por legislação vigente, é primordial respeitá-los durante a produção de conteúdo, portanto, foi dado os créditos (ação

obrigatória) conforme desejo dos autores (FILATRO; CAIRO, 2015) quando utilizados no presente material educativo.

Após a revisão do material educativo, ele seguiu para inserção na plataforma pela equipe técnica e, posteriormente a isto, foi coletada a percepção sobre o curso de usuários da plataforma que o realizaram.

6.3 AVALIAÇÃO DO CURSO “BOAS PRÁTICAS COM INJETÁVEIS: AÇÕES PARA O CONTROLE DE INFECÇÃO” CONFORME PERCEPÇÃO DA POPULAÇÃO-ALVO

Cursos abertos como o objeto aqui descrito, podem ser avaliados, de acordo com o modelo de Kikpatrick para identificar o quanto efetivo eles se apresentam, e este processo pode ocorrer através níveis: reação, aprendizado, comportamento e resultados (KIRKPATRICK; KIRKPATRICK, 2006).

Ressalta-se que todos os níveis são importantes e impactam nos níveis subsequentes. Para tanto, a avaliação aqui descrita refere-se ao primeiro nível, em que se mensura a reação (KIRKPATRICK; KIRKPATRICK, 2006), ou seja, buscou-se identificar como os participantes reagiram ao curso “Boas práticas com injetáveis: ações para o controle de infecção” conforme percepção da população-alvo, como perceberam este e se se sentiram satisfeitos.

Sobre a percepção da população-alvo a respeito do curso, de forma geral, este apresentou uma aceitabilidade satisfatória.

O dispositivo mais utilizado para acesso ao curso foi o computador, enquanto a atuação dentro da área da enfermagem contemplou estudantes (maioria, alunos de curso técnico de enfermagem, com 41,18%) e profissionais de enfermagem (maioria destes enfermeiros, 35,29%), com a maioria sendo do sexo feminino.

Este dado foi semelhante ao encontrado por Antunes et al (2020), cuja maior parte dos participantes utilizaram *notebook* para realizar as disciplinas *online*, sendo do sexo feminino.

Sobre o conteúdo educacional, este deve ser relevante para o sistema e deve atender ao nível de desenvolvimento e interesse do público-alvo, relacionar-se com questões da vida cotidiana, permitindo a construção de conhecimentos e habilidades que perpassem a memorização e compreensão: devendo objetivar o

desenvolvimento da argumentação, crítica e solução de problemas (FILATRO; CAIRO, 2015).

De forma unânime, houve concordância sobre o conteúdo abordado ser relevante para a prática com injetáveis, útil para os participantes e estar com linguagem clara, objetiva, coerente e dialógica.

Quanto a interface do curso, mais de 90% dos participantes desta etapa da pesquisa consideraram-na totalmente simples e de fácil manuseio, enquanto 82,24% concordaram totalmente que é atrativo.

Sobre o ebook “Boas Práticas com Injetáveis: ações para o controle de infecção” os participantes consideram, por unanimidade, que está escrito de forma clara, com texto de fácil leitura, as imagens são significativas para a aprendizagem e que os *links* complementam o curso.

Questionar sobre o quão significativo as informações e imagens foram para o participante do curso intencionou compreender o quanto o sujeito considerou o conhecimento importante, de acordo com seu ponto de vista, é outro fator relacionado à TAS: atribuição pessoal de significado para aquele conteúdo processado, ou seja, a aprendizagem aqui ocorre de forma ativa, dinâmica e intencional (LEMOS, 2006).

Sobre o *podcast* “Boas práticas com medicações injetáveis: subsídios para o autocuidado", 94,22% dos participantes concordaram totalmente que este apresenta fluidez sonora e boa dicção.

Neste ponto, cabe salientar a importância imagética no aprendizado, em que 11,76% afirmaram sentir falta de vídeos no curso.

Assim como enfatizado por Filatro e Cairo (2015), é um desafio tanto a escolha de mídias para subsidiar o conteúdo a ser trabalhado quanto a melhor tecnologia para veiculá-la.

A utilização de *podcast* enquanto recurso tecnológico para a educação profissional e tecnológica tem sido considerada e suas potencialidades expostas, estando a efetividade enquanto ferramenta para aprendizagem significativa relacionada ao seu contexto de aplicação (CORADINI; BORGES; DUTRA, 2020).

Coradini, Borges e Dutra (2020) ressaltam que os estudantes considerados nativos digitais já interagem com elementos que transcendem os textos, como animações, imagens e áudios, cujo acesso a partir de dispositivos móveis favorece a aquisição a grande quantidade de informações disponibilizadas pela internet.

Tem-se que o *podcast* é um tipo de ferramenta midiática em formato de áudio, distribuído digitalmente (em mp3, por exemplo) e que pode ser ouvido sob demanda, ou seja, quando e como o interlocutor quiser e através de qualquer dispositivo. Contudo, trata-se de uma tecnologia ainda em ascensão.

O primeiro agregador do *Podcast* enquanto ferramenta de oralidade foi desenvolvido pelo próprio criador desta, Adam Curry, denominado *iPodder*, o qual foi, posteriormente, disponibilizado em código aberto, o que Freire (2017) considera importante fato do uso da ferramenta enquanto estratégia educacional, cuja primeira utilização data de 2004, contudo, o mesmo autor ressalta o quanto, no Brasil, esta tecnologia educativa ainda é pouco explorada na educação formal, cujo início ocorreu em 2006.

Sobre a transcrição do áudio, trata-se de um recurso para acessibilidade, o qual busca, assim como afirmado por Freire (2017) manter parcialmente a oralidade a partir da reprodução textual de acordo com a fluência do falante.

Tratou-se de um *podcast* expositivo de duração curta (entre um e cinco minutos), a fim de apresentar conteúdo e explicar conceito, não foi associado a imagens ou visualizações dinâmicas, a fim de serem menos pesados, ou seja, possibilitando liberdade de movimentos e sem atenção visual, gravado a partir da voz física da própria pesquisadora (FILATRO; CAIRO, 2015).

Já com relação ao fórum, este ponto foi o mais divergente na gradação de concordância dos participantes, visto que, embora a maioria considere que esta ferramenta possibilita a interação e o compartilhamento de conhecimento entre os participantes do grupo, em algum grau, alguns participantes responderam a este questionamento de forma neutra (23,53% e 17,64%, respectivamente).

Este achado vai de encontro com sugestão de avaliadora do presente trabalho, que apontou a baixa viabilidade deste por tratar-se de curso sem tutoria.

Além disto, assemelha-se e a curso AVA sobre Diabetes Mellitus focado em estudantes de enfermagem, o qual recebeu baixa interatividade (CASTILHO *et al.*, 2020), enquanto há relatos que apontam o quanto fóruns são bem aceitos no papel de compartilhamento de saberes em cursos com tutorias focado em educação continuada com equipe de enfermagem (FRUTUOSO *et al.,* 2019).

De fato, cursos abertos que ocorrem sem a presença de um tutor ainda representa um desafio, visto trabalho de Antunes *et al* (2020) que apontou que 57,2% dos alunos demonstraram preferência por disciplinas *on-line* que utilizam estratégias

tanto de leituras e atividades individuais quanto fóruns de interação e vídeos, enquanto a opção sem a presença de um tutor foi manifestada por 25,5%.

A interação entre participantes é um fator que está associado a melhor efetividade de cursos abertos, bem como o uso de mais de uma ferramenta abordando o conteúdo (LONGHINI *et al.*, 2021)

A atividade para emissão do certificado foi unanimemente reconhecida como pertinente ao conteúdo teórico abordado ao passo que 94,12% concordaram totalmente que esta proporciona relação teórico-prática e 82,35% a consideram motivadora e que estimula sua resolução com entusiasmo (5,88% responderam a esta questão de forma neutra).

Pensar sobre a questão da motivação ao abordar o AVA é considerar que os fatores motivacionais do sujeito interferem na retenção do conteúdo, o que está de acordo com a Teoria de Ausubel, em que, a motivação influencia o aprendizado, pois, é tida como algo espontâneo capaz de produzir satisfação, o que estimulará a continuidade do processo de aprendizagem (KIEFER e PILATTI, 2014).

Estudo que objetivou identificar fatores relacionados à motivação de estudantes de graduação de cursos da área da saúde de uma instituição privada do estado de São Paulo para a realização de disciplinas on-line (por exemplo, biossegurança, gestão em saúde e vigilância sanitária) ambientada pelo Moodle que disponibilizava material textual, em PDF, com avaliação a partir de dez questões de múltipla escolha com cinco alternativas e possuía fórum de interação, identificou que tanto a predisposição para fazer algum curso a distância, quanto a possibilidade de local adequado para estudos e conciliação destes e uso de outros sites influenciam positivamente a motivação dos alunos em disciplinas on-line (ANTUNES et al, 2020).

Para atingir a esta emoção, buscou-se estimular a interação com o aluno a partir do uso da linguagem familiar e procurando relacionar a mesma com elementos que já estivessem consolidados em sua estrutura cognitiva, avaliação contendo situações semelhantes às exemplificadas no decorrer do material (mas não as mesmas, conforme sugerido pela TAS), oferecimento de certificado, implementação do curso em uma plataforma aberta, em que o aluno é responsável pelo gerenciamento do tempo, e, consequentemente, reconheça o seu empenho para a finalização, ou seja a volição.

Para a avaliação da aprendizagem, em consonância com Kiefer e Pilatti (2014) ao citar Faria (1989), foram elaboradas questões pautadas em solução

problema, considerando-se situações que o participante poderia se deparar em sua prática com injetáveis, contudo, não foram utilizados os mesmos exemplos dados no decorrer do material educativo, seja o *ebook* ou o *podcast*, conforme sugerido por Moreira (2011), mas, entendendo-se que o participante poderia relacionar com aquilo que já tivera experienciado, de acordo com sua realidade, tornando a aprendizagem significativa para o mesmo (KIEFER e PILATTI, 2014) opondo-se, assim, a aprendizagem puramente mecânica.

Sobre a funcionalidade do áudio, *links* e carregamento de imagens, 88,24% concordaram que esta apresenta-se boa, somando-se a isso observação de participante que relatou oscilações na plataforma durante a realização de atividade.

Dificuldades de acesso ou para conexão de *internet* são fatores que podem funcionar como barreira a conclusão do curso pelo participante (LONGHINI *et al.*, 2021)

Assim como enfatizado por Antunes *et al.* (2020), a aprendizagem requer mais do que o acesso a um dispositivo compatível com TIC: requer que o AVA possibilite ao aluno o desenvolvimento de motivação.

Para Filatro e Cairo (2015), as pessoas desenvolvem padrões de comportamento em relação à preferência ao processo de aprendizagem a partir de suas experiências na infância e adolescência. Este fator pode ser encarado como um desafio na educação da população adulta, ou seja, não sendo possível atingir a todas as preferências, combinar mídias foi uma alternativa para acolher a este desafio.

Os resultados da implementação desta TIC corroboram com Fetterman *et al*. (2017), que afirmam as potencialidades destes ambientes enquanto desenvolvimento da autonomia dos participantes, motivação para a aprendizagem e capacitação profissional, contudo, no presente caso, não foi possível confirmar sua potencialidade enquanto ferramenta para interação entre participantes, sendo importante enquanto complementar às demais metodologias de ensino.

O uso desta ferramenta vem de encontro a maior disseminação do conhecimento de informática que se tem tornado inerente a profissão da enfermagem, no que concerne ao domínio técnico mínimo (FETTERMANN *et al.*, 2017)

Desta forma, ao elaborar material educativo sobre Boas Práticas com Medicações Injetáveis validado e com boa aceitabilidade inicial, focando profissionais e estudantes de enfermagem, visando o controle de infecção, se corrobora com o

apontamento de Antunes (2020) sobre a importância da qualificação da força de trabalho subsidiada pela TIC, sendo um evento que está em crescimento na era digital.

Ressalta-se que é preciso identificar, também o quanto o curso se mostra efetivo a partir da avaliação da aprendizagem, comportamento e resultados (KIRKPATRICK; KIRKPATRICK, 2006), visto que a avaliação da reação, embora importante para a motivação do sujeito em realizar o curso, não avalia retenção do conteúdo.

6.4 LIMITAÇÕES DO ESTUDO

Como limitação do estudo, cita-se que o trabalho considerou a realidade de uma parcela dos profissionais e não de outras regiões do Brasil.

Além disso, a escolha da implementação do material educativo em uma plataforma aberta, embora contemple uma necessidade global pode não atingir a todos os contextos de educação em enfermagem, visto requerer habilidade técnica em informática e dispositivos que sejam compatíveis.

Já sobre a percepção da população-alvo ressalta-se que, embora ofereça subsídios para identificar como ela reage ao mesmo, e se este se material se mostra motivador, não avalia a retenção de conhecimento, o que demandaria outros estudos, a fim mensurá-lo.

6.5 CONTRIBUIÇÕES PARA A ENFERMAGEM

A partir do diagnóstico situacional das práticas com injetáveis, poder-se-á compreender e comparar como esta se dá nos diferentes cenários, ou ainda, utilizar o instrumento originário desta para configurar a prática em serviços de saúde ou escolas de enfermagem.

De forma geral, o curso sobre “Boas Práticas com Injetáveis: ações para o controle de Infecção” pode ser utilizado como ferramenta complementar para a educação em enfermagem em ambientes formais ou informais de forma gratuita.

Os materiais de apoio, *ebook* e *podcast*/transcrição podem ser baixados e armazenados para visualização posterior ou ainda utilizados como material de apoio para docentes de cursos técnicos ou de graduação, ou mesmo para atualização profissional.

7 CONSIDERAÇÕES FINAIS

A análise contextual foi uma importante etapa para a elaboração do curso "Boas Práticas com Injetáveis: ações para o controle de infecção", assim como proposto pela TAS de Ausubel, pois, possibilitou identificar o quanto o tema não é estranho a esta população (primordial para identificação do conhecimento prévio), bem como confirmar a relevância que a utilização de mídias e tecnologias de apoio poderiam beneficiar o refinamento de práticas ainda não totalmente inseridas na rotina destes profissionais, visto a colaboração de um número significativo de profissionais que aceitaram participar da pesquisa e o perfil demográfico que apontou a afinidade com utilização de computadores para tal, acolhendo profissionais de diferentes faixas etárias e experiências acadêmicas e laborais.

De forma geral, a conduta com injetáveis mostrou-se dentro do preconizado, contudo, embora presentes em um número pequeno de respondentes, percebeu-se a necessidade de aprimoramento teórico frente ao tema visando a segurança do paciente e do profissional para o controle de infecção.

Exemplo é a não há unanimidade no uso de luvas para medicações EV, o que é amplamente recomendado e obrigatório, pois envolve grande risco a exposição biológica.

Dado que foi pouco referido, mas, merece destaque pela relevância e alto risco evidenciado é o compartilhamento de seringas para salinização de cateteres de pacientes diferentes, demonstrando reuso de insumos descartáveis e alto risco de transmissão de patógenos em caso de um dos pacientes apresentar infecções transmissíveis por corrente sanguínea.

Priorizou-se, então, a abordagem destes tópicos no material educativo sobre Boas Práticas com Injetáveis, que, a partir de uma linguagem dialogada viabiliza a reflexão crítica de ações nos serviços de saúde que oportunizam o controle de infecção a partir de uma análise crítica.

Para a validação do material educativo por conteúdo, este apresentou-se válido, visto alta pontuação do IVCES e IVC, contudo, optou-se por realizar pequenas modificações no material, tendo por base as orientações provenientes destas devolutivas pelas avaliadoras.

Após devolutiva do questionário abordando a percepção de participantes do curso (população-alvo), observou-se uma aceitação satisfatória tanto do material

em si quanto do questionário para avaliação do conteúdo, ao passo que o fórum se configurou como a ferramenta potencialmente menos atrativa.

Contudo, ressalta-se a necessidade da aplicação deste material em outros cenários a fim de configurá-lo relevante para outros contextos que não a busca individual, por exemplo, enquanto complemente ao ensino formal e para atualização em serviços de saúde, frente ao tema, e mesmo a partir da visão de pessoas que estejam atuando em outras regiões do país e contextos.

8 CONCLUSÕES

A partir de questionário estruturado e validado para este fim, foi possível identificar o conhecimento prévio que profissionais da saúde possuem sobre a prática com injetáveis, bem como a fluência digital para o acesso a ferramenta educativa abordando este tema.

Constou-se que a maior parte das condutas referidas por estes profissionais estão configuradas dentro das Boas Práticas com Medicações Injetáveis, contudo, há inadequações que chamam à atenção pela relevância temática, como compartilhamento de frascos multidoses, reuso de insumos de uso único entre pacientes, treinamentos com dispositivos de segurança para perfurocortantes infrequente e falta de adesão às luvas para a realização de medicações EV.

Partindo disto, construiu-se e validou-se (com utilização do IVCES e IVC) uma estratégia educativa para profissionais e estudantes de enfermagem em formato AVA, constando de ferramentas como *ebook*, *podcast*, fórum e questionário para emissão de certificado visando às boas práticas na administração de medicações injetáveis.

Este curso foi avaliado quanto a percepção de participantes auto-inscritos, em que se confirmou, a partir do olhar destes, que o curso é uma estratégia válida enquanto ferramenta educativa para a reflexão sobre o tema ao passo que contém informações pertinentes e motivadoras, contudo, o fórum não se mostrou, de forma completa, como ferramenta para interação entre participantes.

Desta forma, trata-se de importante ferramenta a complementar a prática com injetáveis, sendo de valia para serviços de saúde e em ambientes de ensino, tanto para estudantes de graduação quanto de curso técnico, além de estimular que

novas ferramentas sobre os diversos assuntos pertinentes à área sejam implementadas e validadas, utilizando-se TIC como apoio.

REFERÊNCIAS

ABATE, Karen S. The effect of podcast lectures on nursing students' knowledge retention and application. **Nursing Education Perspectives**, v. 34, n. 3, p. 1820185, 2013.

ABEDIN, Tasnima et al. YouTube as a source of useful information on diabetes foot care. **Diabetes research and clinical practice**, v. 110, n. 1, p. e10e4, 2015.

ADEWUYI, Emmanuel O.; AUTA, Asa. Medical injection and access to sterile injection equipment in low0and middle0income countries: a meta0analysis of Demographic and Health Surveys (2010–2017). **International health**, v. 12, n. 5, p. 3880394, 2020.

ALAY et al. Evaluation of Injuries Involving Exposure to Blood and Body Fluids Among Hospital Healthcare Workers. **Mediterranean Journal of Infection Microbes and Antimicrobials**, v. 6, 2017.

AL-RAWAJFAH, Omar M.; TUBAISHAT, Ahmad. A concealed observational study of infection control and safe injection practices in Jordanian governmental hospitals. **American journal of infection control**, v. 45, n. 10, p. 112701132, 2017.

ALVES, Diego Alcântara et al. Avaliação das condutas de punção e manutenção do cateter intravenoso periférico. **Revista de Enfermagem do Centro-Oeste Mineiro**, v. 9, 2019.

ARNOLD, Sandi et al. Notes from the field: hepatitis C transmission from inappropriate reuse of saline flush syringes for multiple patients in an acute care general hospital—Texas, 2015. **MMWR. Morbidity and mortality weekly report**, v. 66, n. 9, p. 258, 2017.

BASKIN, Jacquelyn L. et al. Management of occlusion and thrombosis associated with long-term indwelling central venous catheters. **The Lancet**, v. 374, n. 9684, p. 1590169, 2009.

BELELA-ANACLETO, Aline Santa Cruz; PETERLINI, Maria Angélica Sorgini; PEDREIRA, Mavilde da Luz Gonçalves. Hand hygiene as a caring practice: a reflection on professional responsibility. **Revista brasileira de enfermagem**, v. 70, n. 2, p. 4420445, 2017.

BINSWANGER, Ingrid A. et al. High prevalence of abscesses and cellulitis among community-recruited injection drug users in San Francisco. **Clinical infectious diseases**, v. 30, n. 3, p. 5790581, 2000.

BRAGA, Luciene Muniz et al. Taxa de incidência e o uso do flushing na prevenção das obstruções de cateter venoso periférico. **Texto & Contexto-Enfermagem**, v. 27, n. 4, 2018.

Brasil. Agência Nacional de Vigilância Sanitária. **Medidas de Prevenção de Infecção Relacionada à Assistência à Saúde**. Brasília: Anvisa, 2017.

BRASIL. **Programa Nacional de prevenção e controle de infecções relacionadas à assistência à saúde (201602020)**. Brasília: Anvisa, 2016.

BRASIL. Ministério da Saúde. Secretaria de Vigilância em Saúde. **Boletim Epidemiológico**: HIV/Aids. 2010.

BÜCHNER, Ané et al. Nosocomial outbreak of hepatitis B virus infection in a pediatric hematology and oncology unit in South Africa: Epidemiological investigation and measures to prevent further transmission. **Pediatric blood & cancer**, v. 62, n. 11, p. 191401919, 2015.

CAMERINI, Flavia Giron; SILVA, Lolita Dopico da. Segurança do paciente: análise do preparo de medicação intravenosa em hospital da rede sentinela. **Texto & Contexto-Enfermagem**, v. 20, n. 1, p. 41049, 2011.

CARLSON, Abigail L.; PERL, Trish M. Health care workers as source of hepatitis B and C virus transmission. **Clinics in liver disease**, v. 14, n. 1, p. 1530168, 2010.

CARRARETTO, Antônio Roberto et al. Glass ampoules: risks and benefits. **Revista Brasileira de Anestesiologia**, v. 61, n. 4, p. 5170521, 2011.

CARVALHO, Juliana; ARAGÃO, Isabella. Infografia: conceito e prática. **InfoDesign-Revista Brasileira de Design da Informação**, v. 9, n. 3, p. 1600177, 2012.

CASTILHO, William Lima de et al. Educational module in a virtual learning environment on Diabetes Mellitus. **Enfermería Global**, v. 19, n. 3, p. 3750388, 2020.

CHHABRA, Disksha et al. Knowledge, attitude, and practice study on hepatitis B among medical and nursing undergraduate students of an apex healthcare institute at Uttarakhand foothills: A descriptive analysis. **Journal of family medicine and primary care**, v. 8, n. 7, p. 2354, 2019.

COGO, Ana Luísa; PERRY, Gabriela; SANTOS, Marlise B. Produção de material digital para o ensino de enfermagem. **Revista Novas Tecnologias na Educação**, v. 13, n. 2, 2015.

COLUCI, Marina Zambon Orpinelli; ALEXANDRE, Neusa Maria Costa; MILANI, Daniela. Construção de instrumentos de medida na área da saúde. **Ciência & Saúde Coletiva**, v. 20, p. 9250936, 2015.

CONOVER, William Jay. **Practical Nonparametric Statistics**. New York: [s.n.], 1999.

CONSELHO REGIONAL DE ENFERMAGEM DE SÃO PAULO. **Parecer nº014/2010**. Dispõe sobre o uso de luvas de procedimento para a administração de medicamentos. 2010.

COOKE, Catherine E.; STEPHENS, Jennifer M. Clinical, economic, and humanistic burden of needlestick injuries in healthcare workers. **Medical Devices (Auckl)**, v. 10, p. 225, 2017.

CORADINI, Neirimar Humberto Kochhan; BORGES, Aurélio Ferreira; DUTRA, Charles Emerick Medeiros. Tecnologia educacional podcast na educação profissional e tecnológica. **Revista eletrônica científica ensino interdisciplinar**, v. 6, n. 16, p. 2160230, 2020.

COSTA, Isabel Karolyne Fernandes et al. Development of a virtual simulation game on basic life support. **Revista da Escola de Enfermagem da USP**, v. 52, 2018.

D'AQUINO, Maria; DE SOUZA, Rogério Marques. Hipodermóclise ou via subcutânea. **Revista Hospital Universitário Pedro Ernesto**, v. 11, n. 2, 2012.

ESCOBAR, Bolívar Teston de. Infográficos de saúde em EAD na UNASUS0UFMA: um enfoque de gestão de design. 2018.

ESTEQUI, Jeanine Geraldi et al. Boas práticas na manutenção do cateter intravenoso periférico. **Enfermagem em Foco**, v. 11, n. 1, p. 10–14, 2020.

FARIAS, Quitéria Larissa Teodoro et al. Implicações das tecnologias de informação e comunicação no processo de educação permanente em saúde. **Revista Eletrônica de Comunicação, Informação e Inovação em Saúde**, v. 4, n. 11, 2017.

FERNANDES, Stéfani Martins; HENN, Leonardo Guedes; KIST, Liane Batistela. O ensino a distância no Brasil: alguns apontamentos. **Research, Society and Development**, v. 9, n. 1, p. e21911551, 1 jan. 2020.

FERREIRA, Adriano Menis et al. Antissepsia na administração de injetáveis: necessidade ou ritualismo?. **Rev. enferm. UERJ**, p. 1300133, 2009.

FERREIRA, Giovanna da Silva et al. Boas práticas na administração de medicamentos endovenosos. **The Brazilian Journal of Infectious Diseases**, v. 25, 2021.

FERRONI, Agnès et al. Pulsative flushing as a strategy to prevent bacterial colonization of vascular access devices. **Medical Devices (Auckl)**, v. 7, p. 379, 2014.

FETTERMANN, Fernanda Almeida et al. Potencialidades e fragilidades dos ambientes virtuais de aprendizagem no ensino em enfermagem: revisão integrativa. **Journal of Health Informatics**, v. 9, n. 4, 2017.

FILATRO, Andrea; CAIRO, Sabrina. **Produção de Conteúdo Educacionais**. 1. ed. São Paulo: Saraiva, 2015.

FLEISS, Joseph L.; LEVIN, Bruce; PAIK, Myunghee Cho. **Statistical Methods for Rates and Proportions**. 3. ed. Hoboken, NJ: John Wiley & Sons, 2003.

FODA, Nermine Mohamed Tawfik; ELSHAER, Noha Selim Mohamed; SULTAN, Yasmine Hussein Mohamed. Safe injection procedures, injection practices, and needlestick injuries among health care workers in operating rooms. **Alexandria Journal of Medicine**, v. 54, n. 1, p. 85092, 2018.

FROTA, Natasha Marques et al. Construção de uma tecnologia educacional para o ensino de enfermagem sobre punção venosa periférica. **Revista Gaúcha de Enfermagem**, v. 34, n. 2, p. 29036, 2013.

FROTA, Natasha Marques et al. Validation of educational hypermedia about peripheral venipuncture. **Texto & Contexto-Enfermagem**, v. 24, n. 2, p. 3530361, 2015.

FRUTUOSO, Isabela Schumaher et al. Creation of a virtual learning environment in intensive care. **Revista de Enfermagem UFPE**, v. 13, n. 5, 2019.

GADIOLI, Bárbara et al. Construction and validation of a virtual learning object for the teaching of peripheral venous vascular semiology. **Escola Anna Nery**, v. 22, n. 4, 2018.

GALDINO, Danielle Patricia; MERCADO, Luis Paulo Leopoldo. AVALIAÇÃO DE MATERIAL DIDÁTICO EM VÍDEO PARA EDUCAÇÃO A DISTÂNCIA: um estudo de caso do curso de Especialização do Programa Nacional de formação em Administração Pública. **Educação em Foco**, p. 2280263, 2017.

GOOSSENS, Godelieve Alice. Flushing and locking of venous catheters: available evidence and evidence deficit. **Nursing research and practice**, v. 2015, 2015.

GRAZIANO, Maurício Uchikawa et al. Eficácia da desinfecção com álcool 70% (p/v) de superfícies contaminadas sem limpeza prévia. **Revista Latino0Americana de Enfermagem**, Ribeirão Preto , v. 21, n. 2, p. 6180623, Abr. 2013.

HATCHER, L. **A Step-by-Step Approach to Using the SAS System for Factor Analysis and Structural Equation Modeling**. Cary, NC: SAS Institute Inc, 1994.

HUTIN, Yvan et al. Best infection control practices for intradermal, subcutaneous, and intramuscular needle injections. **Bulletin of the World Health Organization**, v. 81, p. 4910500, 2003.

TRELHA, Edna Yukimi Itakussu et al. Elaboração de vídeo educativo sobre uso da malha compressiva após queimadura. **Revista Brasileira de Queimaduras**, v. 13, n. 4, p. 2360239, 2014.

KIRKPATRICK, James D.; KIRKPATRICK, Donald L. **Evaluating Training Programs:** The Four Levels. 3a ed. San Francisco: Berrett0Koehler Publishers, 2006.

KOBAYASHI, Rika Miyahara; DE ARAÚJO, Gabriela Denise. Avaliação do treinamento mediado por tecnologias educacionais: revisão integrativa. **Journal of Health Informatics**, v. 11, n. 3, 2019.

KOSSOVER-SMITH, Rachel A. et al. One needle, one syringe, only one time? A survey of physician and nurse knowledge, attitudes, and practices around injection safety. **American journal of infection control**, v. 45, n. 9, p. 101801023, 2017.

LEE, Seung Eun; QUINN, Brenna L. Incorporating medication administration safety in undergraduate nursing education: A literature review. **Nurse education today**, v. 72, p. 77083, 2019.

LEITE, Sarah de Sá et al. Construction and validation of an Educational Content Validation Instrument in Health. **Revista brasileira de enfermagem**, v. 71, p. 163501641, 2018.

LEMOS, Evelyse dos Santos. A Aprendizagem Significativa: estratégias facilitadoras e avaliação. **Série-Estudos-Periódico do Programa de Pós-graduação em Educação da UCDB**, 2006.

LIMA, Marília Brito de et al. Construção e validação de vídeo educativo para orientação de pais de crianças em cateterismo intermitente limpo. **Revista da Escola de Enfermagem da USP**, v. 51, 2017.

JESSICA, Longhini et al. What knowledge is available on massive open online courses in nursing and academic healthcare sciences education? A rapid review. **Nurse Education Today**, p. 104812, 2021.

MADHAVAN, Anitha et al. Comparison of knowledge, attitude, and practices regarding needle0stick injury among health care providers. **Journal of family medicine and primary care**, v. 8, n. 3, p. 840, 2019.

MAGEROTE, Nelissa de Paula et al. Associação entre flebite e retirada de cateteres intravenosos periféricos. **Texto & Contexto-Enfermagem**, v. 20, n. 3, p. 4860492, 2011.

MÄNNISTÖ, Merja et al. Digital collaborative learning in nursing education: A systematic review. **Scandinavian journal of caring sciences**, v. 34, n. 2, p. 2800292, 2020.

MARCONI, Marina de Andrade; LAKATOS, Eva Maria. **Fundamentos de metodologia científica**. 7. ed. São Paulo: Atlas, 2010.

MARTINS, Lara Barros; ZERBINI, Thaís. Evidências de validade de instrumentos de reações no ensino superior à distância. **Estudos e Pesquisas em Psicologia**, v. 15, n. 1, p. 1160134, 2015.

METTIÄINEN, Sari et al. Web course on medication administration strengthens nursing students' competence prior to graduation. **Nurse Education in Practice**, v. 14, n. 4, p. 3680373, 2014.

MONCAIO, Ana Carolina Scarpel; FIGUEIREDO, Rosely Moralez de. Conhecimentos e práticas no uso do cateter periférico intermitente pela equipe de enfermagem. **Revista Eletrônica de Enfermagem**, v. 11, n. 3, 2009.

MOREIRA, Marco Antonio. **Aprendizagem significativa: a teoria e textos complementares**. 1 ed. São Paulo: Editora Livraria da Física. 2011a.

MOREIRA, Marco Antonio. Aprendizagem Significativa: Um Conceito Subjacente. **Aprendizagem Significativa em Revista**, v.1, n.3, 2011b.

MOREIRA, Marco Antonio; MASINI, Elcie F Salzano. **Aprendizagem Significativa: a teoria de Deivid Ausubel**. São Paulo: Centauro, 2001.

MOTA, Rafaela De Oliveira et al. Preparo de medicamentos administrados via intramuscular na pediatria: atuação da equipe de enfermagem. **Cogitare Enfermagem**, v. 21, p. 1–9, 2016.

MOTAMEDIFAR, Mohammad; ASKARIAN, Mehrdad. The prevalence of multidose vial contamination by aerobic bacteria in a major teaching hospital, Shiraz, Iran, 2006. **American journal of infection control**, v. 37, n. 9, p. 7730777, 2009.

MUSAVI GHAHFAROKHI, Motahareh; MASOUDIYEKTA, Leila; KHAJEALI, Nasrin. The level of safety standards in implementing therapeutic and caring procedures by emergency department personnel. **Nursing open**, v. 6, n. 1, p. 109, 2019.

NAKAYAMA, Naomi et al. Implementation of intravenous drip infusion therapy with peripheral venous catheters and the incidence of related complications in home0based medical care settings in Japan. **Annals of Nutrition and Metabolism**, v. 73, p. 1000105, 2018.

NEGELISKII, Christian. **Efeito de uma intervenção educativa com profissionais de enfermagem acerca da segurança do paciente na administração de medicamentos injetáveis**. 2015. Tese (Doutorado em Enfermagem) – Programa de Pós-graduação em Enfermagem, Universidade Federal do Rio Grande do Sul, Rio Grande do Sul, 2015.

NEGELISKII, Christian; LAUTERT, Liana. O preparo e administração de medicamentos injetáveis: a banalização deste cuidado de enfermagem. **Revista Sul0Brasileira de Enfermagem**, v. 23, n. 6, p. 17, 2017.

NORONHA, Márcia Pessoa de Sousa. Tecnologias digitais educacionais utilizadas nas práticas pedagógicas no ensino em Enfermagem. 2018.

NOUETCHOGNOU, Julienne Stéphanie et al. Accidental exposures to blood and body fluids among health care workers in a Referral Hospital of Cameroon. **BMC research notes**, v. 9, n. 1, p. 106, 2016.

NUNNALLY, Jum. **Psychometric Theory**. 2 ed. New York: McGraw-Hill, 1978.

O'GRADY, Naomi. et al. Guidelines for the prevention of intravascular catheter0related infections. **Clinical infectious diseases**, v. 52, n. 9, p. e1620e193, 2011.

OLIVEIRA, Tainã Clarissa Santos da Silva de et al. Preparo e administração de medicamentos por via subcutânea: os saberes da equipe de enfermagem. **Revista Enfermagem Atual In Derme**, v. 87, n. 25, 2019.

OLUWATOSIN, O. Abimbola; OLADAPO, M. Moyosola; ASUZU, Michael C. Needlestick injuries among health care workers in Ondo State, Nigeria. **International Journal of Medicine and Public Health**, v. 6, n. 1, 2016.

ORGANIZAÇÃO PAN0AMERICANA DA SAÚDE. **Uso de Luvas**. Folha Informativa,n.6, 2010. Disponível em: < https://www.paho.org/bra/index.php?option=com_docman&view=download&alias=4980folha0informativa0uso0luvas08&category_slug=seguranca0do0paciente0970&Itemid=965>. Acesso em 21 abr 2021.

ORGANIZAÇÃO MUNDIAL DA SAÚDE. **WHO guideline on the use of safety-engineered syringes for intramuscular, intradermal and subcutaneous injections in health care settings**. World Health Organization, 2016.

ORGANIZAÇÃO MUNDIAL DA SAÚDE. **Glove Use.** Information Leaflet. Patient Safety, 2009.

ORGANIZAÇÃO MUNDIAL DA SAÚDE. **WHO best practices for injections and related procedures toolkit**. Geneva, 2010.

PARULLA, Cibele Duarte et al. Nursing assessment: the elaboration and development of a massive open online course. **Revista Gaúcha de Enfermagem**, v. 41, n. SPE, 2020.

PEREIRA, Júlio Cesar Rodrigues. **Análise de Dados Qualitativos – Estratégias Metodológicas para as Ciências da Saúde, Humanas e Sociais**. São Paulo: EDUSP, 1999.

PERZ, Joseph F. et al. Case-control study of hepatitis B and hepatitis C in older adults: Do healthcare exposures contribute to burden of new infections?. **Hepatology**, v. 57, n. 3, p. 9170924, 2013.

PERZ, Joseph F. et al. US outbreak investigations highlight the need for safe injection practices and basic infection control. **Clinics in liver disease**, v. 14, n. 1, p. 1370151, 2010.

PIMENTEL, Fernando Silvio Cavalcante; PINTO, Anamelea de Campos; MERCADO, Luis Paulo Leopoldo. **Indicadores para avaliação de cursos em EAD**. In: MACHADO, Glaucio José Couri. Educação e ciberespaço: estudos, propostas e desafios. Aracaju: Virtus, 2010. p. 208–235.

PINTO, Thais da Rocha Cicero et al. Educational animation about home care with premature newborn infants. **Revista Brasileira de Enfermagem**, v. 71, p. 160401610, 2018.

PRADO, Cláudia; VAZ, Débora Rodrigues; ALMEIDA, Denise Maria de. Teoria da aprendizagem significativa: elaboração e avaliação de aula virtual na plataforma Moodle. **Revista Brasileira de Enfermagem**, v. 64, n. 6, p. 11401121, 2011.

PUGLIESE, Gina et al. Injection practices among clinicians in United States health care settings. **American Journal of Infection Control**, v. 38, n. 10, p. 7890798, 2010.

RAMOS, Susana Maria Sardinha Vieira. **Prevenção e controlo da infeção na preparação e administração de medicamentos endovenosos**. 2013. Dissertação (Mestrado em Infecção em Cuidados de Saúde) – Universidade Católica Portuguesa, Lisboa, 2013.

REICHEMBACH, Mitsy Tânnia; MEIER, Marineli Joaquim; ASCHIDAMINI, Ione Maria. Administração de medicamentos por via subcutânea: convenção ou controvérsia para a enfermagem?. **Revista Brasileira de Enfermagem**, v. 58, n. 5, p. 6020606, 2005.

RIGOTTI, Marcelo Alessandro. **Segurança microbiológica na abertura de ampolas com ênfase no procedimento de desinfecção**. 2012. Dissertação (Mestrado em Ciências) – Programa de Enfermagem Fundamental, Universidade de São Paulo. Ribeirão Preto, 2012.

RODRIGUES JUNIOR, Jânio Cavalcanti et al. Construção de vídeo educativo para a promoção da saúde ocular em escolares. **Texto & Contexto0Enfermagem**, v. 26, n. 2, 2017.

ROSA, Bruna Vanessa Costa da et al. Desenvolvimento e validação de tecnologia educativa audiovisual para famílias e pessoas com colostomia por câncer. **Texto & Contexto 0 Enfermagem.**, Florianópolis, v. 28, e20180053, 2019.

SÃO PAULO. Secretaria Municipal da Saúde. **Manual de normas, rotinas e procedimentos de Enfermagem:** Atenção Básica. 2 ed. São Paulo: Secretaria Municipal da Saúde, 2014. Disponível em: <http://www.prefeitura.sp.gov.br/cidade/secretarias/upload/saude/arquivos/legislacao/NormaseRotinas02102015.pdf>. Acesso em 21 abr 2021.

SÃO PAULO. Secretaria Municipal da Saúde. Recomendações sobre o uso de luvas em serviços de saúde, 2016. Disponível em: < https://www.cristofoli.com/biosseguranca/wp0content/uploads/2018/10/CVE_USAO_LUVAS.pdf>. Acesso em 21 abr 2021.

SATO, Akihiro et al. Peripheral venous catheter0related bloodstream infection is associated with severe complications and potential death: a retrospective observational study. **BMC Infectious Diseases**, v. 17, n. 1, p. 106, 2017.

SCHUTIJSER, Bernadette et al. Nurse compliance with a protocol for safe injectable medication administration: comparison of two multicentre observational studies. **BMJ open**, v. 8, n. 1, 2018.

SEXSON, Kathryn; LINDAUER, Allison; HARVATH, Theresa A. Administration of subcutaneous injections.**The American Journal of Nursing**, v. 117, n. 5, p. S70S10, 2017.

SIEGEL, Sidney; CASTELLAN, John. **Estatística Não-Paramétrica para as Ciências do Comportamento.** 2. Ed. Editora Penso. 2006.

SHANKS, Robert et al. Catheter lock solutions influence staphylococcal biofilm formation on abiotic surfaces. **Nephrology Dialysis Transplantation**, v. 21, n. 8, p. 224702255, 2006.

SILVA, Natiele Favarão da et al. Construção e validação de um vídeo educativo sobre a reflexologia podal. **Revista Eletrônica de Enfermagem**, v. 19, 2017.

SOBATI, Abolfazl et al. Understanding the factors associated with nonreporting of needlestick injuries in nurses at Imam Khomeini Hospital Complex, Tehran 2016: A case study. **Annals of Tropical Medicine and Public Health**, v. 10, n. 3, p. 651, 2017.

SOUSA, Alana Tamar Oliveira de et al. A utilização da teoria da aprendizagem significativa no ensino da Enfermagem. **Revista Brasileira de Enfermagem**, v. 68, n. 4, p. 7130722, 2015.

SRIRAM, Shyamkumar. Study of needle stick injuries among healthcare providers: Evidence from a teaching hospital in India. **Journal of Family Medicine and Primary Care**, v. 8, n. 2, p. 599, 2019.

STEINKULLER, Fozia et al. Outpatient infection prevention: a practical primer. In: **Open Forum Infectious Diseases**. US: Oxford University Press, v. 5, n. 5, 2018.

STREINER, David L. Starting at the beginning: an introduction to coefficient alpha and internal consistency. **Journal of Personality Assessment**, v. 80, n. 1, p. 990103, 2003.

STREINER, David L.; NORMAN, Geoffrey R. **Health Measurement Scales**: A Practical Guide to Their Development and Use. Oxford: Oxford University Press, 1995.

STRUCHINER, Miriam; GIANNELLA, Taís R. **Novas tecnologias de informação e comunicação na formação de recursos humanos em saúde**. In: MONTEIRO, S., and VARGAS, E. orgs. Educação, comunicação e tecnologia

educacional: interfaces com o campo da saúde [online]. Rio de Janeiro: Editora FIOCRUZ, 2006, pp. 1290140.

SWIGART, Valerie; LIANG, Zhan. Digital resources for nursing education: Open courseware and massive open online courses. **International Journal of Nursing Sciences**, v. 3, n. 3, p. 3070313, 2016.

TABACHNICK, Barbara G. FIDELL, Linda S. Using Multivariate Statistics. 5ed. California, 2007.

THOMAS, Larissa Scheeren; FONTANA, Rosane Teresinha. Uso das Tecnologias de Informação e Comunicação como meio educacional na saúde: revisão integrativa. **Research, Society and Development**, v. 9, n. 10, p. e98691093210e9869109321, 2020.

UNIVERSIDADE FEDERAL DE SÃO CARLOS. Secretaria Geral de Educação a Distância. **Portal de Cursos Abertos**. Disponível em: <https://poca.ufscar.br/>. Acesso em 21 abr 2021.

VAN TUONG, Phan et al. Assessment of injection safety in Ha Dong general hospital, Hanoi, in 2012. **F1000Research**, v. 6, 2017.

WANDERLEY, Tatiana Peres Santana Porto et al. Docência em saúde: tempo de novas tecnologias da informação e comunicação. **Revista Eletrônica de Comunicação, Informação e Inovação em Saúde**, v. 12, n. 4, 2018.

APÊNDICE A – Termo de Consentimento Livre e Esclarecido encaminhado aos juízes para validação do Questionário para Identificação das Boas Práticas com Medicações Injetáveis

UNIVERSIDADE FEDERAL DE SÃO CARLOS
DEPARTAMENTO DE ENFERMAGEM
PROGRAMA DE PÓS-GRADUAÇÃO EM ENFERMAGEM

TERMO DE CONSENTIMENTO LIVRE E ESCLARECIDO
(Resolução 466/2012 do CNS)
BOAS PRÁTICAS EM MEDICAÇÕES INJETÁVEIS: UMA INTERVENÇÃO EDUCATIVA PARA A EQUIPE DE ENFERMAGEM NO ESTADO DE SÃO PAULO

O (a) Senhor (a) está sendo convidado (a) para participar da pesquisa “Boas práticas em medicações injetáveis: uma intervenção educativa para a equipe de enfermagem no estado de São Paulo”. O qual justifica-se pela literatura científica, que aponta para a necessidade emergente de estratégias que otimizem a adesão às boas práticas de medicações injetáveis e tendo em vista a indisponibilidade de trabalhos que demonstrem este cenário no estado de São Paulo, almeja-se apresentar um diagnóstico situacional referente ao tema e a partir deste construir um material educativo e disponibilizá-lo para todos os profissionais de enfermagem.

O objetivo deste estudo é desenvolver e validar uma estratégia educativa para profissionais de enfermagem visando às boas práticas na administração de medicações injetáveis. O (a) senhor (a) foi selecionado (a) por ter experiências clínicas na área de controle de infecção relacionada à assistência à saúde, ou tecnologia da informação e comunicação aplicada à enfermagem, possuir publicação ou pesquisar sobre o tema, ter conhecimento sobre a construção de questionários ou atuar profissionalmente como enfermeiro. Sua participação é voluntária e consiste em responder formulário em anexo a fim de validar o instrumento por conteúdo para posterior coleta de dados utilizando o mesmo com equipe de enfermagem no Estado de São Paulo, e a qualquer momento o (a) senhor (a) pode desistir de participar e retirar seu consentimento. A sua recusa não trará nenhum prejuízo na sua relação com o pesquisador ou com a instituição que forneceu os dados. Em caso de esclarecimentos quanto à pesquisa, poderá entrar em contato com a pesquisadora pelo telefone ou e-mail abaixo deste termo, a qualquer momento.

A validação deste questionário enviado eletronicamente, aborda questões referentes à rotina de trabalho em diferentes serviços de saúde envolvendo boas praticas de medicações injetáveis, ou seja, medidas que minimizam o risco de infecção relacionada à assistência à saúde e um breve questionário pessoal no qual deve informar seu nível de instrução, tempo de atuação na área e de serviço em que atua. O tempo utilizado para coleta dos dados será de aproximadamente 45 minutos.

Suas respostas serão tratadas de forma anônima e confidencial em qualquer fase do estudo. Quando for necessário exemplificar determinada situação, sua privacidade será assegurada. Os dados coletados poderão ter seus resultados divulgados em eventos, revistas e/ou trabalhos científicos.

O preenchimento destes questionários não oferece risco imediato ao (a) senhor (a), porém considera-se a possibilidade de um risco subjetivo, pois algumas perguntas podem levar a sentir-se desconfortável, evocar sentimentos ou lembranças desagradáveis, sentir-se cansado após responder o questionário. Caso algumas dessas possibilidades ocorram, o senhor (a) poderá optar pela suspensão imediata de sua participação nesta pesquisa.

O senhor (a) não terá nenhum custo ou compensação financeira ao participar do estudo. Contudo, ressalta-se que a sua participação auxiliará a conhecer o contexto atual das boas práticas de medicações injetáveis no estado de São Paulo, o qual embasará uma estratégia educativa a ser divulgado eletronicamente, ao qual o (a) senhor (a) terá acesso assim que publicado. Há direito a indenização por qualquer tipo de dano resultante da sua participação na pesquisa.

O (a) senhor (a) receberá uma via deste termo, rubricada em todas as páginas por você e pelo pesquisador, onde consta o telefone e o endereço do pesquisador principal.

Declaro que entendi os objetivos, riscos e benefícios de minha participação na pesquisa e concordo em participar. O pesquisador me informou que o projeto foi aprovado pelo Comitê de Ética em Pesquisa em Seres Humanos da UFSCar que funciona na Pró-Reitoria de Pesquisa da Universidade Federal de São Carlos, localizada na Rodovia Washington Luiz, Km. 235 0 Caixa Postal 676 0 CEP 13.5650905 - São Carlos - SP – Brasil. Fone (16) 335108028. Endereço eletrônico: cephumanos@ufscar.br

Endereço para contato

Pesquisador Responsável: Camila Eugenia Roseira

Endereço: UFSCar, Rod. Washington Luís, Km 235, São Carlos/SP

Contato telefônico: (16) 335108338. e-mail: roseira.camila@gmail.com

Local e data: ____________________________________

___________________________ ___________________________

Nome do Pesquisador Assinatura do Pesquisador

APÊNDICE B – Termo de Consentimento Livre e Esclarecido encaminhado aos participantes da pesquisa que responderam ao Questionário para Identificação das Boas Práticas com Medicações Injetáveis

UNIVERSIDADE FEDERAL DE SÃO CARLOS
DEPARTAMENTO DE ENFERMAGEM
PROGRAMA DE PÓS-GRADUAÇÃO EM ENFERMAGEM

TERMO DE CONSENTIMENTO LIVRE E ESCLARECIDO
(Resolução 466/2012 do CNS)

BOAS PRÁTICAS EM MEDICAÇÕES INJETÁVEIS: UMA INTERVENÇÃO EDUCATIVA PARA A EQUIPE DE ENFERMAGEM NO ESTADO DE SÃO PAULO

O (a) Senhor (a) está sendo convidado (a) para participar da pesquisa “Boas práticas em medicações injetáveis: uma intervenção educativa para a equipe de enfermagem no estado de São Paulo”. O qual justifica-se pela literatura científica, que aponta para a necessidade emergente de estratégias que otimizem a adesão às boas práticas de medicações injetáveis e tendo em vista a indisponibilidade de trabalhos que demonstrem este cenário no estado de São Paulo, almeja-se apresentar um diagnóstico situacional referente ao tema e a partir deste construir um material educativo e disponibilizá-lo para todos os profissionais de enfermagem.

O objetivo deste estudo é desenvolver e validar uma estratégia educativa para profissionais de enfermagem visando às boas práticas na administração de medicações injetáveis. O (a) senhor (a) foi selecionado (a) por ser profissional da área da enfermagem e possuir registro ativo no conselho regional do estado de São Paulo. Sua participação é voluntária, isto é, a qualquer momento o (a) senhor (a) pode desistir de participar e retirar seu consentimento. A sua recusa não trará nenhum prejuízo na sua relação com o pesquisador ou com a instituição que forneceu os dados. Em caso de esclarecimentos quanto à pesquisa, poderá entrar em contato com a pesquisadora pelo telefone ou e-mail abaixo deste termo, a qualquer momento.

A coleta de dados será composta por este questionário validado por especialistas, enviado eletronicamente, o qual aborda questões referentes à sua rotina de trabalho envolvendo boas práticas de medicações injetáveis, ou seja, medidas que minimizam o risco de infecção relacionada à assistência à saúde e um breve questionário pessoal no qual deve informar sua categoria profissional, nível de instrução, tempo de atuação na área e de serviço em que atua. O tempo utilizado para coleta dos dados será de aproximadamente trinta minutos.

Suas respostas serão tratadas de forma anônima e confidencial, desta forma, não será divulgado seu nome em qualquer fase do estudo. Quando for necessário exemplificar determinada situação, sua privacidade será assegurada. Os dados coletados poderão ter seus resultados divulgados em eventos, revistas e/ou trabalhos científicos.

O preenchimento destes questionários não oferece risco imediato ao (a) senhor (a), porém considera-se a possibilidade de um risco subjetivo, pois algumas perguntas podem levar a sentir-se desconfortável, evocar sentimentos ou lembranças desagradáveis, sentir-se cansado após responder o questionário. Caso algumas dessas possibilidades ocorram, o senhor (a) poderá optar pela suspensão imediata de sua participação nesta pesquisa.

O senhor (a) não terá nenhum custo ou compensação financeira ao participar do estudo. Contudo, ressalta-se que a sua participação auxiliará a conhecer o contexto atual das boas práticas de medicações injetáveis no estado de São Paulo, o qual embasará uma estratégia educativa a ser divulgado eletronicamente, ao qual o (a) senhor (a) terá acesso assim que publicado. Há o direito a indenização por qualquer tipo de dano resultante da sua participação na pesquisa.

O (a) senhor (a) receberá uma via deste termo, rubricada em todas as páginas por você e pelo pesquisador, onde consta o telefone e o endereço do pesquisador principal

Declaro que entendi os objetivos, riscos e benefícios de minha participação na pesquisa e concordo em participar. O pesquisador me informou que o projeto foi aprovado pelo Comitê de Ética em Pesquisa em Seres Humanos da UFSCar que funciona na Pró-Reitoria de Pesquisa da Universidade Federal de São Carlos, localizada na Rodovia Washington Luiz, Km. 235 - Caixa Postal 676 - CEP 13.565-905 - São Carlos - SP – Brasil. Fone (16) 3351-8028. Endereço eletrônico: cephumanos@ufscar.br

Endereço para contato

Pesquisador Responsável: Camila Eugenia Roseira
Endereço: UFSCar, Rod. Washington Luís, Km 235, São Carlos/SP
Contato telefônico: (16) 3351-8338. e-mail: roseira.camila@gmail.com

Local e data: ____

______________________________ ______________________________

Nome do Pesquisador Assinatura do Pesquisador

APÊNDICE C - Termo de Consentimento Livre e Esclarecido encaminhado aos avaliadores para validação do material educativo do curso “Boas Práticas com Injetáveis: ações para o controle de infecção”

UNIVERSIDADE FEDERAL DE SÃO CARLOS
DEPARTAMENTO DE ENFERMAGEM
PROGRAMA DE PÓS GRADUAÇÃO EM ENFERMAGEM

TERMO DE CONSENTIMENTO LIVRE E ESCLARECIDO
(Resolução 466/2012 do CNS)
BOAS PRÁTICAS EM MEDICAÇÕES INJETÁVEIS: UMA INTERVENÇÃO EDUCATIVA PARA A EQUIPE DE ENFERMAGEM NO ESTADO DE SÃO PAULO

O (a) Senhor (a) está sendo convidado (a) para participar da pesquisa “Boas práticas em medicações injetáveis: uma intervenção educativa para a equipe de enfermagem no estado de São Paulo”. O qual justifica-se pela literatura científica, que aponta para a necessidade emergente de estratégias que otimizem a adesão às boas práticas de medicações injetáveis e tendo em vista a indisponibilidade de trabalhos que demonstrem este cenário no estado de São Paulo, almeja-se apresentar um diagnóstico situacional referente ao tema e a partir deste construir um material educativo e disponibilizá-lo para todos os profissionais de enfermagem.

O objetivo deste estudo é desenvolver e validar uma estratégia educativa para profissionais de enfermagem visando às boas práticas na administração de medicações injetáveis. O (a) senhor (a) foi selecionado (a) por ter experiências clínicas na área de controle de infecção relacionada à assistência à saúde, ou tecnologia da informação e comunicação aplicada à enfermagem, possuir publicação ou pesquisar sobre o tema, ter conhecimento sobre a construção de questionários ou atuar profissionalmente como enfermeiro. Sua participação é voluntária e consiste em avaliar este material educativo quanto ao conteúdo, sugerindo modificações e realizando observações conforme julgar pertinente. A qualquer momento o (a) senhor (a) pode desistir de participar e retirar seu consentimento. A sua recusa não trará nenhum prejuízo na sua relação com o pesquisador ou com a instituição que forneceu os dados. Em caso de esclarecimentos quanto à pesquisa, poderá entrar em contato com a pesquisadora pelo telefone ou e-mail abaixo deste termo, a qualquer momento.

A validação deste material educativo enviado eletronicamente, o qual aborda questões referentes às boas praticas de medicações injetáveis a partir de um diagnóstico situacional realizado previamente no estado de São Paulo, do qual participaram profissionais de enfermagem deste estado, objetiva o aperfeiçoamento e a implantação de medidas que minimizam o risco de infecção relacionada à assistência à saúde e há um breve questionário pessoal no qual deve informar seu nível de instrução, tempo de atuação na área e de serviço em que atua. O tempo utilizado para coleta dos dados será de aproximadamente 45 minutos.

Suas respostas serão tratadas de forma anônima e confidencial em qualquer fase do estudo, sendo sua privacidade assegurada. Os dados coletados poderão ter seus resultados divulgados em eventos, revistas e/ou trabalhos científicos.

A avaliação deste material não oferece risco imediato ao (a) senhor (a), porém considera-se a possibilidade de um risco subjetivo, pois algumas perguntas podem levar a sentir-se desconfortável, evocar sentimentos ou lembranças desagradáveis, sentir-se cansado após responder o questionário. Caso algumas dessas possibilidades ocorram, o senhor (a) poderá optar pela suspensão imediata de sua participação nesta pesquisa.

O senhor (a) não terá nenhum custo ou compensação financeira ao participar do estudo. Contudo, ressalta-se que a sua participação auxiliará a conhecer o contexto atual das boas práticas de medicações injetáveis no estado de São Paulo, o qual embasará uma estratégia educativa a ser divulgado eletronicamente, ao qual o (a) senhor (a) terá acesso assim que publicado. Há direito a indenização por qualquer tipo de dano resultante da sua participação na pesquisa.

O (a) senhor (a) receberá uma via deste termo, rubricada em todas as páginas por você e pelo pesquisador, onde consta o telefone e o endereço do pesquisador principal.

Declaro que entendi os objetivos, riscos e benefícios de minha participação na pesquisa e concordo em participar. O pesquisador me informou que o projeto foi aprovado pelo Comitê de Ética em Pesquisa em Seres Humanos da UFSCar que funciona na Pró-Reitoria de Pesquisa da Universidade Federal de São Carlos, localizada na Rodovia Washington Luiz, Km. 235 0 Caixa Postal 676 0 CEP 13.5650905 0 São Carlos 0 SP – Brasil. Fone (16) 335108028. Endereço eletrônico: cephumanos@ufscar.br

Endereço para contato

Pesquisador Responsável: Camila Eugenia Roseira
Endereço: UFSCar, Rod. Washington Luís, Km 235, São Carlos/SP
Contato telefônico: (16) 335108338. e-mail: roseira.camila@gmail.com
Local e data: ____

__________________________ Nome do Pesquisador

__________________________ Assinatura do Pesquisador

APÊNDICE D Termo de Consentimento Livre e Esclarecido encaminhado a população-alvo para avaliar a percepção do curso "Boas Práticas com Injetáveis: ações para o controle de infecção"

UNIVERSIDADE FEDERAL DE SÃO CARLOS

DEPARTAMENTO DE ENFERMAGEM

PROGRAMA DE PÓS GRADUAÇÃO EM ENFERMAGEM

TERMO DE CONSENTIMENTO LIVRE E ESCLARECIDO

(Resolução 466/2012 do CNS)

BOAS PRÁTICAS EM MEDICAÇÕES INJETÁVEIS: UMA INTERVENÇÃO EDUCATIVA PARA A EQUIPE DE ENFERMAGEM NO ESTADO DE SÃO PAULO

Prezado(a), o senhor(a) está sendo convidado(a) para participar da pesquisa "Boas práticas em medicações injetáveis: uma intervenção educativa para a equipe de enfermagem no estado de São Paulo". Esta pesquisa se justifica pela literatura científica, que aponta para a necessidade emergente de estratégias que aperfeiçoem a adesão às boas práticas de medicações injetáveis. O objetivo deste estudo é desenvolver e validar uma estratégia educativa para profissionais de enfermagem visando às boas práticas na administração de medicações injetáveis. O material educativo anteriormente citado refere-se ao curso aberto "Boas Práticas em Medicações Injetáveis". O curso "Boas Práticas em Medicações Injetáveis" aborda as boas práticas de medicações injetáveis a partir de um diagnóstico situacional realizado previamente no estado de São Paulo, do qual participaram profissionais de enfermagem deste estado, e objetiva o aperfeiçoamento e a implantação de medidas que minimizam o risco de infecção relacionada à assistência à saúde. O (a) senhor(a) foi selecionado(a) por ser um(a) aluno(a) do curso "Boas Práticas em Medicações Injetáveis", ter mais de 18 anos e ser profissional ou estudante de enfermagem. Sua participação é voluntária e consiste em preencher um questionário abordando seus dados sociodemográficos e sua percepção sobre o curso em questão. O tempo utilizado para responder ao questionário será de aproximadamente 45 minutos. A qualquer momento o (a) senhor(a) pode desistir de participar e retirar seu consentimento. A sua recusa não trará nenhum prejuízo na sua relação com o pesquisador ou com a instituição. Em caso de esclarecimentos quanto à pesquisa, poderá entrar em contato com a pesquisadora pelo telefone ou e-mail abaixo deste termo, a qualquer momento. Ressalta-se que a sua participação nesta pesquisa não influencia na conclusão do curso "Boas Práticas em Medicações Injetáveis" e tampouco tem relação com a avaliação para a emissão do certificado. Suas respostas serão tratadas de forma anônima e confidencial em qualquer fase do estudo, sendo sua privacidade assegurada. Os dados coletados poderão ter seus resultados divulgados em eventos, revistas e/ou trabalhos científicos. O preenchimento destes questionários não oferece risco imediato ao (a) senhor (a), porém considera-se a possibilidade de um risco subjetivo, pois algumas perguntas podem levar a sentir-se desconfortável, evocar sentimentos ou lembranças desagradáveis, sentir-se cansado após responder o questionário. Caso algumas dessas possibilidades ocorram, o senhor (a) poderá optar pela suspensão imediata de sua participação nesta pesquisa. O senhor (a) não terá nenhum custo ou compensação financeira ao participar do estudo. Contudo, ressalta-se que a sua participação auxiliará na reflexão sobre como o material sobre Boas Práticas em Medicações Injetáveis se comporta enquanto estratégia educativa segundo a percepção dos profissionais ou estudantes da área da enfermagem. Há direito a indenização por qualquer tipo de dano comprovadamente resultante da sua participação na pesquisa. Ao assinalar eletronicamente que aceita participar da pesquisa, o (a)

senhor (a) receberá por e-mail uma via assinada e rubricada deste termo onde consta o telefone e o endereço do pesquisador principal, podendo tirar suas dúvidas sobre o projeto e sua participação, agora ou a qualquer momento. Declaro que entendi os objetivos, riscos e benefícios de minha participação na pesquisa e concordo em participar. O pesquisador me informou que o projeto foi aprovado pelo Comitê de Ética em Pesquisa em Seres Humanos da UFSCar que funciona na Pró Reitoria de Pesquisa da Universidade Federal de São Carlos, localizada na Rodovia Washington Luiz, Km. 235 0 Caixa Postal 676 0 CEP 13.5650905 0 São Carlos 0 SP – Brasil.

Fone (16) 335108028. Endereço eletrônico: cephumanos@ufscar.br

Endereço para contato

Pesquisador Responsável: Camila Eugenia Roseira

Endereço: UFSCar, Rod. Washington Luís, Km 235, São Carlos/SP

Contato telefônico: (16) 335108338. e-mail: roseira.camila@gmail.com

APÊNDICE E – Versão do Questionário para Identificação das Boas Práticas com Medicações Injetáveis encaminhado ao comitê de juízes para validação

Orientações de preenchimento para os juízes

O(a) senhor(a) está sendo convidado a participar, na qualidade de juiz, do questionário de coleta de dados do projeto intitulado "Boas práticas em medicações injetáveis: uma intervenção educativa para a equipe de enfermagem no estado de São Paulo" com o objetivo de desenvolver e validar uma estratégia educativa para profissionais de enfermagem visando às boas práticas na administração de medicações injetáveis, sendo este questionário voltado para a equipe de enfermagem registrada pelo Conselho de Enfermagem do Estado de São Paulo cujos dados embasarão a elaboração deste material. O instrumento de coleta de dados é composto pelos domínios: Preparo do Ambiente, Preparo das Medicações, Administração Medicamentosa e Cuidados após Administração Medicamentosa. O referencial utilizado para elaboração dos itens deste questionário bem como de sua estrutura, está ao final deste, em referências. **O padrão de resposta que os participantes responderão para cada item será assinalando entre "Sempre", "Muito frequente", "Não realizo a atividade", "Poucas vezes" e "Nunca".**

Após responder o breve questionário abaixo com relação às questões pessoais para caracterização do comitê de juízes, solicita-se que, primeiramente, avalie cada item segundo clareza e pertinência (COLUCI; ALEXANDRE; MILANI, 2015), conforme escala de 1 a 4 abaixo:

1	2	3	4
**Não claro**	Item necessita de grande revisão para ser claro	Item necessita de pequena revisão para ser claro	Item claro
**Não pertinente**	Item necessita de grande revisão para ser relevante	Item necessita de pequena revisão para ser relevante	Item pertinente

Clareza: avaliar se os itens são redigidos de modo que o conceito esteja compreensível e se expresse adequadamente ao que se espera medir.

Pertinência: avaliar se os itens refletem os conceitos envolvidos, se são relevantes e adequados para atingir objetivos almejados.

Questionário Pessoal

Categoria profissional: ( ) enfermeiro ( ) Outro _______________________________
Qual seu maior grau de instrução?
( ) especialização _______________________
( ) mestrado ___________________________
( ) doutorado __________________________
( ) Outra: ___________________

Há quanto tempo atua na área? ( ) 0-5 anos ( ) 6-10 anos ( ) 11-20 anos ( ) 21 a 30 anos ( ) 30 anos ou mais

Em qual tipo de serviço você atua? (pode ser assinalado mais de um)
( ) Hospital
( ) ambulatórios/Centros de especialidades
( ) Unidades de Pronto Atendimento (UPA)
( ) Atenção básica/Atenção primária (UBS/USF, etc)
( ) outros serviços não hospitalares
( ) escolas técnicas
( ) faculdades/universidades
( ) outros. Quais?_________________________________

Sua atividade principal na enfermagem é de caráter:
( ) assistencial
( ) gerencial/administrativa
( ) ensino

Domínio 1: PREPARO DO AMBIENTE										
ITENS	Clareza				Pertinência				Sugestão de nova redação	Observação
Com que frequência você desinfeta balcão para preparo das medicações antes do início de suas atividades com álcool a 70% ou outras substâncias padronizadas em seu serviço?	1	2	3	4	1	2	3	4		
Com que frequência você higieniza as bandejas de preparo e administração de medicações injetáveis a cada preparo destas?	1	2	3	4	1	2	3	4		
Com que frequência você verifica a data de validade de materiais utilizados para administração medicamentosa antes de sua utilização?	1	2	3	4	1	2	3	4		
Com que frequência você utiliza materiais para administração medicamentosa cuja embalagem esteja aberta?	1	2	3	4	1	2	3	4		
Com que frequência você higieniza as mãos antes e após o preparo de medicações injetáveis?	1	2	3	4	1	2	3	4		
Domínio 2: PREPARO DAS MEDICAÇÕES										
ITENS	Clareza				Pertinência				Sugestão de nova redação	Observação
Com que frequência você higieniza as mãos, com água e sabonete ou solução alcóolica, antes da administração de medicação endovenosas?	1	2	3	4	1	2	3	4		
Com que frequência você compartilha frascos de soros (de 100 a 1000ml) no preparo de medicações no posto de enfermagem para reconstituição de doses medicamentosas?	1	2	3	4	1	2	3	4		
Com que frequência você compartilha o mesmo frasco com multidoses para dois ou mais pacientes?	1	2	3	4	1	2	3	4		

Com que frequência você realiza desinfecção com álcool a 70% das tampas de frascos multidoses antes da aspiração do medicamento a ser administrado?	1	2	3	4	1	2	3	4		
Com que frequência você utiliza a mesma agulha para aspirar medicações de frascos multidoses?	1	2	3	4	1	2	3	4		
Com que frequência você utiliza medicações de frascos que constem data de abertura do mesmo, volume de diluente (se aplicável), nome do profissional que realizou a abertura e reconstituiu a medicação?	1	2	3	4	1	2	3	4		
Com que frequência você realiza assepsia de frascos e ampolas de medicação antes de aspirá-la?	1	2	3	4	1	2	3	4		
Domínio 3: ADMINISTRAÇÃO MEDICAMENTOSA										
ITENS	Clareza				Pertinência				Sugestão de nova redação	Observação
Com que frequência você saliniza cateteres venosos periféricos intermitentes com solução fisiológica ou heparinizada?	1	2	3	4	1	2	3	4		
Com que frequência você utiliza a mesma seringa para sorolização ou heparinização de cateteres venosos periféricos em diferentes pacientes?	1	2	3	4	1	2	3	4		
Com que frequência você reutiliza a mesma tampinha, de fechamento de dupla via, em cateteres venosos periféricos após administração medicamentosa por esta via?	1	2	3	4	1	2	3	4		
Com que frequência você armazena tampinha de cateter venoso periférico em mesas de cabeceira, bolsos de jaleco/avental ou outros locais para posterior reutilização desta?	1	2	3	4	1	2	3	4		
	1	2	3	4	1	2	3	4		

Com que frequência você realiza a desobstrução de cateteres venosos periféricos com a infusão forçada de soro fisiológico?										
Com que frequência realiza desinfecção de conectores com álcool a 70% antes da punção venosa periférica?	1	2	3	4	1	2	3	4		
Com que frequência você reutiliza agulhas ou seringas para preparo de medicações do mesmo paciente?	1	2	3	4	1	2	3	4		
Com que frequência você reutiliza agulhas ou seringas para preparo de medicações de pacientes diferentes?	1	2	3	4	1	2	3	4		
Com que frequência você reutiliza seringas para salinização de pacientes diferentes?	1	2	3	4	1	2	3	4		
Com que frequência você realiza assepsia da pele do paciente com álcool a 70% para administrar medicações subcutâneas?	1	2	3	4	1	2	3	4		
Com que frequência você realiza assepsia da pele do paciente para realizar medicações, que não vacinas, pela via intramuscular?	1	2	3	4	1	2	3	4		
Com que frequência você toca a área da pele do paciente em que já realizou assepsia antes da administração do medicamento injetável?	1	2	3	4	1	2	3	4		
Com que frequência você reutiliza seringas modificando a agulha desta para administração medicamentosa injetável no mesmo paciente ou em pacientes diferentes?	1	2	3	4	1	2	3	4		
Com que frequência você realiza lavagem do acesso venoso após administração de medicamentos com SF0,9%?	1	2	3	4	1	2	3	4		
Com que frequência você reutiliza materiais descartáveis como tampinhas de equipos ou polifix?	1	2	3	4	1	2	3	4		
Com que frequência você recebe treinamento para manipulação de agulhas e cateteres com dispositivos de segurança?	1	2	3	4	1	2	3	4		

Com que frequência você realiza ações de enfermagem com a integridade de suas mãos prejudicadas?	1	2	3	4	1	2	3	4		
Com que frequência você utiliza luvas para administrar medicações intramusculares?	1	2	3	4	1	2	3	4		
Com que frequência você utiliza luvas para administrar medicações subcutâneas?	1	2	3	4	1	2	3	4		
Com que frequência você utiliza luvas para administrar medicamentos por via endovenosa?	1	2	3	4	1	2	3	4		
Com que frequência você utiliza luvas para administrar medicamentos via endovenosa apenas para paciente sabidamente acometidos com vírus como HIV, hepatites B ou C?	1	2	3	4	1	2	3	4		
Com que frequência realiza curativo estéril para manutenção dos cateteres de infusão medicamentosa?	1	2	3	4	1	2	3	4		
Com que frequência realiza troca de acesso venoso periférico programada (entre 72 e 96 horas) antes dos sinais de flebite local?	1	2	3	4	1	2	3	4		
Domínio 4: CUIDADOS APÓS ADMINISTRAÇÃO MEDICAMENTOSA										
ITENS	Clareza				Pertinência				Sugestão de nova redação	Observação
Com que frequência você reencapa agulhas após realizar medicações no paciente antes de descartar os resíduos?	1	2	3	4	1	2	3	4		
Com que frequência você higieniza as mãos antes e após a colocação de luvas para administração medicamentosa?	1	2	3	4	1	2	3	4		
Com que frequência você transporta material como seringas e agulhas utilizadas desprotegidas até local adequado de descarte?	1	2	3	4	1	2	3	4		
	1	2	3	4	1	2	3	4		

Com que frequência você separa a agulha do corpo da seringa após medicar pacientes para o descarte dos resíduos?										
Com que frequência você se preocupa em vacinar-se contra doenças potencialmente passíveis de transmissão ocupacional, como a hepatite B?	1	2	3	4	1	2	3	4		
Com que frequência você procura serviço de saúde ocupacional após exposição percutânea com material perfurocortante em até 72 horas após o acidente?	1	2	3	4	1	2	3	4		
Com que frequência, após sofrer acidente com perfurocortante você julga adequado espremer a área afetada e colocar álcool a 70% para desinfetar a região para diminuir a probabilidade de infecção de patógenos transmitidos por via sanguínea?	1	2	3	4	1	2	3	4		

Agora, avalie cada domínio quanto à abrangência, concordando com esta quando cada domínio foi adequadamente contemplado pelo conjunto de itens (COLUCI; ALEXANDRE; MILANI, 2015).

	Abrangência		Comentário
Domínio 1: PREPARO DO AMBIENTE	Concordo	Discordo	
Domínio 2: PREPARO DAS MEDICAÇÕES			
Domínio 3: ADMINISTRAÇÃO MEDICAMENTOSA			
Domínio 4: CUIDADOS APÓS ADMINISTRAÇÃO MEDICAMENTOSA			

Você considera que a linguagem empregada no questionário é adequada para os participantes da pesquisa?

Há alguma sugestão ou observação a fazer, considerando-se o questionário de forma geral?

REFERÊNCIAS

BRASIL (BR). Agência Nacional de Vigilância Sanitária. Programa Nacional de prevenção e controle de infecções relacionadas à assistência à saúde (201602020). Brasília, DF, 2016.

COLUCI, MZO; ALEXANDRE, NMC; MILANI, D. Construção de instrumentos de medida na área da saúde. Ciênc. saúde coletiva, Rio de Janeiro, v. 20, n. 3, p. 9250936, Mar. 2015. Disponível em <http://www.scielo.br/scielo.php?script=sci_arttext&pid=S141308123201500030092 5&lng=en&nrm=iso>. Acesso em 16/04/2017.

MONCAIO ACS, FIGUEIREDO RM. Conhecimentos e práticas no uso do cateter periférico intermitente pela equipe de enfermagem. **Rev. Eletr. Enf.** [Internet], v.11, n.3, p.620-7,2009. Disponível em <http://www.fen.ufg.br/revista/v11/n3/v11n3a20.htm>

NEGELISKII, C. **Efeitos de uma intervenção educativa com profissionais de enfermagem acerca da segurança do paciente na administração de medicamentos injetáveis**. Tese (doutorado). Universidade Federal do Rio Grande do Sul.144f. 2015.

PUGLIESI G, GOSNELL C, BARTLEY JM, ROBINSON S. Injection practices among clinicians in United States health care settings. **American Journal of Infection Control**, v. 38 n. 10, p789098; 2010.

WORLD HEALTH ORGANIZATION. **WHO best practices for injections and related procedures toolkit**. Geneva, Suiça, 2010.

WORLD HEALTH ORGANIZATION. **Patient Safety Curriculum Guide Multi-Professional Edition**. 2011.

APÊNDICE F – Recomendações presentes na literatura para as Boas Práticas com Medicações Injetáveis, conforme conteúdo dos itens do Questionário para Identificação das Boas Práticas com Medicações Injetáveis

Recomendações presentes na literatura para as Boas Práticas com Medicações Injetáveis, conforme conteúdo dos itens do Questionário para Identificação das Boas Práticas com Medicações Injetáveis
Limpeza de balcão para preparo das medicações
Comentário: Deve-se considerar que as medicações devem ser preparadas em área limpa, sem contaminação por sangue e fluidos corporais, em superfícies limpas, antes do início de preparo de injetáveis e sempre que houver contaminação por sangue e fluidos corpóreos, deve-se proceder à limpeza com solução alcoólica a 70% e aguardar a secagem espontânea (ORGANIZAÇÃO MUNDIAL DA SAÚDE, 2010)
Limpeza de bandejas a cada preparo de medicações injetáveis
Comentário: antes do início de preparo de injetáveis e sempre que houver contaminação por sangue e fluidos corpóreos, deve-se proceder à limpeza com solução alcoólica a 70% e aguardar a secagem espontânea (ORGANIZAÇÃO MUNDIAL DA SAÚDE, 2010)
Verificação da data de validade de insumos antes do uso
Comentário: devem ser descartados insumos cuja validade está vencida, ou a embalagem esteja úmida, furada, danificada (ORGANIZAÇÃO MUNDIAL DA SAÚDE, 2010)
Utilização de insumos com embalagens danificadas
Comentário: esta prática configura utilização de equipamentos não estéreis (injeção insegura). Deve-se utilizar um novo disposto descartável a cada procedimento, certificando-se de que haja integridade do invólucro que funciona como barreira protetiva (ORGANIZAÇÃO MUNDIAL DA SAÚDE, 2010)
Higienização das mãos antes e após o preparo de medicações injetáveis
Comentário: está relacionada a redução e prevenção de transmissão de infecção, deve ser realizada durante o preparo de material injetável (ORGANIZAÇÃO MUNDIAL DA SAÚDE, 2010)
Compartilhamento de frascos de solução fisiológica para reconstituição de doses medicamentosas
Comentário: não é recomendado o uso de bolsas de soro (100 ml) para reconstituição de doses de medicamento, para injeção de rotina tampouco seringas preenchidas para diluição medicamentosa, visto alta probabilidade de contaminação (BRASIL, 2017; ORGANIZAÇÃO MUNDIAL DA SAÚDE, 2010)
Utilização de frascos multidoses para dois ou mais pacientes
Comentário: deve-se evitar o uso de frascos multidose, pois, há alta probabilidade de contaminação quando a técnica asséptica é frágil, só é permitido quando não há outra alternativa e de ocorrer da seguinte forma: deixar, preferencialmente, um frasco multidose para casa paciente, com o nome do mesmo, armazenamento longe de contaminantes por spray ou respingos. O frasco deve ser descartado se: a esterilidade ou conteúdo foi comprometido, estiver fora do prazo de validade, após 24 horas de abertura, e acessá-lo com novos dispositivos estéreis, sempre 0 ou conforme recomendação do fabricante (ORGANIZAÇÃO MUNDIAL DA SAÚDE, 2010)
Desinfecção do septo de frascos multidoses pré0aspiração

Comentário: deve-se realizar a limpeza do diafragma (septo) do frasco com álcool a 70% antes de perfurá-lo, permitindo sua secagem prévia (ORGANIZAÇÃO MUNDIAL DA SAÚDE, 2010)
Utilização mesma agulha para aspiração de medicações de frascos multidoses
Comentário: deve-se utilizar um novo dispositivo descartável a cada procedimento, incluindo reconstituição de dose medicamentosa, ou seja, acessar o frasco com novo conjunto de seringa e agulha a cada utilização, não deixando a agulha no mesmo (ORGANIZAÇÃO MUNDIAL DA SAÚDE, 2010)
Identificação de frascos multidoses (data, volume do diluente, nome do profissional)
Comentário: após reconstituição ou data de abertura, deve-se rotular o frasco com os seguintes dados: data e horário do preparo, tipo e volume do diluente (s/n), concentração final (s/n), data de validade após reconstituição (s/n), nome e assinatura do profissional após reconstituição (ORGANIZAÇÃO MUNDIAL DA SAÚDE, 2010)
Limpeza septo de frascos e ampolas pré-aspiração
Comentário: deve-se realizar a limpeza do topo do frasco com álcool a 70% antes de perfura-lo, permitindo sua secagem prévia (ORGANIZAÇÃO MUNDIAL DA SAÚDE, 2010); há artigo que relata que esta é uma prática desnecessária (HUTIN *et al.*, 2003)
Reutilização de agulhas ou seringas para preparo de medicação do mesmo paciente
Comentário: deve-se utilizar um novo disposto descartável a cada procedimento, deve-se acessar ao frasco com nova seringa e agulha, mesmo que seja para o mesmo paciente (ORGANIZAÇÃO MUNDIAL DA SAÚDE, 2010)
Reutilização de agulhas ou seringas para preparo de medicação de pacientes diferentes
Comentário: deve-se utilizar um novo disposto descartável a cada procedimento. Não utilizar seringa ou agulha para mais de um paciente (ORGANIZAÇÃO MUNDIAL DA SAÚDE, 2010)
Salinização de cateteres venosos periféricos
Comentário: deve ocorrer antes e após a administração medicamentosa. O valor de cinco ml, embora possa reduzir o volume de fibrina e as drogas precipitadas, deve passar pela avaliação clínica do paciente, bem como o tipo de solução infundida, que pode demandar maiores volumes. Quando antes de cada administração medicamentosa juntamente com a aspiração para garantir a funcionalidade do cateter e prevenção de complicações com seringa de 10 ml para gerar baixa pressão no lúmen, previne a mistura de medicamentos incompatíveis, garante o funcionamento do cateter e previne complicações. (BRASIL, 2017)
Higienização das mãos com água e sabão antes da administração medicamentosa
Comentário: as mãos devem ser higienizadas antes e após a inserção de cateteres e ao manipular o dispositivo. Deve ocorrer com água e sabonete líquido quando visivelmente sujas, contaminadas com sangue ou fluidos corporais: antes e após tocar o sítio de inserção do cateter, antes e após a inserção, remoção manipulação ou troca de curativo (BRASIL, 2017). Este é um mecanismo de redução de exposição e prevenção de transmissão de patógenos (ORGANIZAÇÃO MUNDIAL DA SAÚDE, 2010)
Higienização das mãos com solução alcoólica/solução padronizada antes da administração de medicamentos via endovenosa
Comentário: utilizar preparação alcoólica (entre 60 e 80%) quando as mãos não estiverem visivelmente sujas: antes e após tocar o sítio de inserção do cateter, antes e após a inserção, remoção, manipulação ou troca de curativo (BRASIL, 2017). Este é um

mecanismo de redução de exposição e prevenção de transmissão de patógenos (ORGANIZAÇÃO MUNDIAL DA SAÚDE, 2010)
Compartilhamento de seringa para salinização de cateteres venosos periféricos em diferentes pacientes
Comentário: deve-se utilizar um novo disposto descartável a cada procedimento. Não utilizar seringa ou agulha para mais de um paciente (ORGANIZAÇÃO MUNDIAL DA SAÚDE, 2010)
Reutilização de oclusores de dupla via de cateteres periféricos
Comentário: deve-se descartar oclusores das dânulas a cada a cada uso e utilizar oclusores estéreis (BRASIL, 2017)
Armazenamento inapropriado de oclusores de cateteres periféricos para reutilização
Comentário: deve-se descartar oclusores das dânulas a cada a cada uso e utilizar oclusores estéreis (BRASIL, 2017)
Desobstrução forçada com solução fisiológica 0,9% de cateteres venosos periféricos
Comentário: em caso de resistência deve-se utilizar seringa de 10 ml para gerar baixa pressão no lúmen do cateter e avaliar fatores de resistência. Sugere-se a sequência flushing pulsátil (mais efetivo na remoção de depósito sólido, quando em comparação com o contínuo) imediatamente a cada uso, fechar clamp e desconectar a seringa para minimizar o refluxo de sangue pós desconexão da seringa uso (BRASIL, 2017)
Desobstrução forçada com heparina de cateteres venosos periféricos
Comentário: não se deve forçar desobstrução de cateteres venosos periféricos e recomenda-se realizar flushing com solução fisiológica 0,9% (BRASIL, 2017)
Desinfecção de oclusores de dispositivos intravenosos com álcool a 70% previamente a administração medicamentosa
Comentário: deve-se realizar desinfecção dos conectores a cada acesso ou manipulação a partir de solução antisséptica a base de álcool, com fricção mecânica por 5015 segundos (BRASIL, 2017)
Reutilização de seringa para salinização de acesso venoso periférico de pacientes diferentes
Comentário: deve-se utilizar um novo disposto descartável a cada procedimento. Não utilizar seringa ou agulha para mais de um paciente (ORGANIZAÇÃO MUNDIAL DA SAÚDE, 2010)
Antissepsia da pele do paciente com álcool a 70% para administração de medicações subcutâneas
Comentário: embora a OMS afirme que não é necessário, preconizando-se realização de limpeza com água e sabão (ORGANIZAÇÃO MUNDIAL DA SAÚDE, 2010), bem como estudo que sugeriu que durante a perfuração com agulha, a maior parte das bactérias que adentram ao tecido da flora da pele do sujeito não são patogênicas, e estão em número menor do que o necessário para desencadear reação infecciosa com formação de pus, a partir de estudo com usuários de drogas injetáveis via subcutânea ou intramuscular, não identificando diferença significativa entre abcesso ou celulite entre grupos de indivíduos que realizaram ou não a limpeza prévia da área (BINSWANGER *et al.*, 2000). Contudo, as orientações esclarecem que há fragilidades, uma vez que as boas práticas referentes à injeções subcutâneas, intradérmicas e intramusculares possuem documentação técnica limitada (HUTIN *et al.*, 2003). Há relatos de que, mesmo não havendo necessidade, deve-se realizar a antissepsia de pele com álcool a 70% e aguardar o mesmo secar, por ser uma prática comum (OLIVEIRA *et al.*, 2019; SEXSON;

LINDAUER; HARVATH, 2017), havendo sugestão desta para hipodermóclise (D'AQUINO; SOUZA, 2012)
Antissepsia da pele do paciente com álcool a 70% para administração de medicações intramusculares
Comentário: deve-se realizar a antissepsia da pele com solução alcoólica para administração de medicamentos intramusculares (ORGANIZAÇÃO MUNDIAL DA SAÚDE, 2010)
Quebra da técnica *no touch* para administração de injetáveis
Comentário: deve-se aplicar a técnica do *no touch* no sítio de inserção do cateter após realização de antissepsia, ou calçar luvas estéreis, se necessário, deve-se aguardar a secagem antes da palpação local (BRASIL, 2017; O'GRADY et al., 2011)
Utilização da mesma seringa, com troca da agulha, para administração de injetável no mesmo paciente
Comentário: compartilhamento de seringa entre pacientes pode oferecer risco para transmissão de patógenos, não se deve ainda reutilizar a mesma seringa utilizando outra agulha (ORGANIZAÇÃO MUNDIAL DA SAÚDE, 2010)
Utilização da mesma seringa, com troca da agulha, para administração de injetável em pacientes diferentes
Comentário: não se deve, ainda, reutilizar a mesma seringa utilizando outra agulha (ORGANIZAÇÃO MUNDIAL DA SAÚDE, 2010)
Infusão com solução fisiológica 0,9% de dispositivo intravenoso após administração medicamentosa
Comentário: deve ser realizado imediatamente a cada uso. Devem ser utilizadas frascos de dose única ou seringas preenchidas com soluções com cloreto de sódio 0,9% para *flushing* ou *lock* do cateter (BRASIL, 2017)
Reutilização de insumos descartáveis (oclusores, dupla via)
deve-se descartar oclusores das dânulas a cada a cada uso e utilizar oclusores estéreis (BRASIL, 2017)
Treinamento para manipulação de agulhas e cateteres com dispositivos de segurança
Comentário: a educação e o treinamento de profissionais da saúde responsáveis pela inserção ou manutenção dos cateteres está entre as recomendações para prevenir infecções relacionadas aos mesmos e manter diretrizes atualizadas sobre o tema, em fácil acesso (O'GRADY et al., 2011), o treinamento é uma estratégia preventiva ao acidente com perfurocortante que empodera o profissional para uso de dispositivos de segurança que previnem a reutilização destes insumos (ORGANIZAÇÃO MUNDIAL DA SAÚDE, 2010)
Administração de medicações com lesões de pele nas mãos
Comentário: deve-se evitar ministrar injeções em caso da pele estar com a integridade cutânea prejudicada por infecção local ou outras injúrias como dermatite, corte ou lesão cutânea, devendo-se proceder à cobertura de pequenos cortes. Em presença de lesões, deve-se evitar higienizar as mãos com solução alcoólica em casos de pele não integra, procedendo-se a higienização das mãos com água e sabonete antisséptico ou sabão, água corrente e toalha de papel de uso único em caso de contato da lesão com fluidos corporais ou sangue (ORGANIZAÇÃO MUNDIAL DA SAÚDE, 2010)
Uso de luvas para administração de medicações intramusculares
Comentário: não é necessário utilizar luvas para administração de injeção intramuscular, exceto se a pele do profissional ou paciente não estiver íntegra, como em casos de

eczema, queimadura ou infecção cutânea (MONCAIO; FIGUEIREDO, 2009; SÃO PAULO, 2016; ORGANIZAÇÃO MUNDIAL DA SAÚDE, 2010). O Coren-SP orienta sobre a necessidade de protocolos institucionais a fim da padronização do processo de administração de medicamentos (COREN0SP, 2010)
Uso de luvas para administração de medicações subcutâneas
Comentário: não é necessário utilizar luvas para administração de injeção subcutânea, exceto se a pele do profissional ou paciente estiver não estiver íntegra, como em casos de eczema, queimadura ou infecção cutânea (SÃO PAULO, 2016; ORGANIZAÇÃO MUNDIAL DA SAÚDE, 2010, ORGANIZAÇÃO PAN0AMERICANA DA SAÚDE, 2010). Para a administração de hipodermóclise há a sugestão de uso de luvas durante o procedimento (D'AQUINO; SOUZA, 2012)
Uso de luvas para administração de medicações endovenosas
Comentário: deve-se utilizar luvas de procedimento para inserção de cateteres vasculares periféricos (O'GRADY *et al.*, 2011)
Uso de luvas para administração de medicações endovenosas apenas para paciente sabidamente acometidos com vírus como HIV, hepatites B ou C
Comentário: deve-se usar luvas de procedimento para redução da contaminação nas mãos dos profissionais da saúde por sangue ou fluidos corporais, sugere-se, ainda, seu uso, ainda em casos de precaução por contato e em situais de surto (ORGANIZAÇÃO MUNDIAL DA SAÚDE, 2009)
Curativo estéril para fixação/manutenção dos cateteres venosos periféricos
Comentário: utilizar fitas adesivas podem ser facilmente contaminadas por microrganismos patogênicos, portanto, não devem ser utilizadas como coberturas ou para estabilização do cateter, uma vez que o curativo objetiva proteger o sítio de punção, minimizar a possibilidade de infecção e prevenir a movimentação do dispositivo com dano ao vaso, o mesmo deve ser protegido com cobertura impermeável para não molhar durante o banho, reduzindo a entrada de microrganismos pelo sítio do cateter como cobertura, pois, podem ser facilmente contaminadas por microrganismos patogênicos (BRASIL, 2017). Em caso de utilização de gaze e fita adesiva estéril (rolos não estéreis podem ser facilmente contaminados com microrganismos patogênicos) podem ser utilizados quando a previsão do acesso for menor que 48h, em caso de sangramento ou diaforese, pois aumenta risco de perda do acesso durante troca, os demais casos, deve-se priorizar cobertura estéril transparente (BRASIL, 2017; O'GRADY et al., 2011)
Troca de acesso venoso periférico, conforme padronização do serviço, antes do aparecimento de sinais de flebite
Comentário: rotineiramente, não deve ser trocado em período inferior a 96h, devendo ser removido em caso de instalação emergencial, contaminação, complicação ou mau funcionamento. Deve-se avaliar o sítio de inserção do cateter e área ao redor quanto a rubor, edema, secreção e valorizar queixas dolorosas ou de parestesia ao palpar curativo intacto, a cada quatro horas ou dependendo da criticidade do paciente. Se gaze e fita adesiva estéril, trocar a cada 48h, se cobertura estéril transparente, trocar a cada sete dias, havendo sujidade, umidade ou soltura, qualquer cobertura deve ser trocada (BRASIL, 2017). Visualização sugerida: a cada 4h, se em terapia intensiva, sedado ou com déficit cognitivo: entre 1 e 2h, pediátrico: no mínimo 2 vezes no turno, em unidade de internação: uma vez por turno. Deve haver remoção do cateter assim que não houver medicação prescrita nas últimas 24h, se suspeita de contaminação, complicação ou mau funcionamento e deve ser trocado quando instalado em situação emergencial com

comprometimento da técnica asséptica, assim que possível (BRASIL, 2017). O uso prolongado de cateteres venosos periféricos pode desencadear flebite, e é raramente associado com o desenvolvimento de infecção de corrente sanguínea (O'GRADY et al., 2011)
Reencape de agulha após administração medicamentosa
Comentário: não se deve reencapar agulhas após sua utilização, se necessário, proceder ao reencape com uma mão (ORGANIZAÇÃO MUNDIAL DA SAÚDE, 2010)
Higieniza das mãos antes e após o uso de luvas para administração medicamentosa
Comentário: a higienização das mãos deve ocorrer antes e após a administração de medicamento injetável (ORGANIZAÇÃO MUNDIAL DA SAÚDE, 2010) ao passo que o uso de luvas não substitui a higienização das mãos (BRASIL, 2017)
Transporte desprotegido, até local de descarte, de seringas e agulhas contaminados
Comentário: descarte imediato do material utilizado no local utilizado, em caixa coletora de perfurocortante, utilização de dispositivo de segurança (por exemplo, capa protetora para agulhas pós0utilização, dispositivo que retraia a agulha após procedimento) e sistemas intravenosos que não utilizam agulhas, podem ser utilizados (ORGANIZAÇÃO MUNDIAL DA SAÚDE, 2010)
Separação entre agulha e corpo da seringa para o descarte após medicar pacientes
Comentário: deve-se proceder ao descarte conjunto do dispositivo, evitando-se a separação (ORGANIZAÇÃO MUNDIAL DA SAÚDE, 2010)
Ciência sobre situação vacinal contra doenças potencialmente passíveis de transmissão ocupacional, como a hepatite B
Comentário: vacina contra hepatite B deve ser administrada em todo profissional da saúde a fim de reduzir o risco de infecção por HBV em caso de acidente percutâneo, protegendo também o paciente (ORGANIZAÇÃO MUNDIAL DA SAÚDE, 2010)
Ciência sobre fluxo de atendimento pós exposição percutânea com material perfurocortante no seu local de trabalho
Comentário: a literatura está documentada sobre a transmissão de patógenos para os profissionais durante acidentes ocupacionais com perfurocortantes, risco aumentado quando a pessoa exposta não recebe profilaxia pós exposição, deve-se após acidente com material perfurocortante, registrar o acidente e iniciar profilaxia pós-exposição o mais precocemente (primeiras 72 horas), seguindo o protocolo institucional – POP (ORGANIZAÇÃO MUNDIAL DA SAÚDE, 2010)
Cuidados inapropriados com lesão decorrente de acidente ocupacional com perfurocortante
Comentário: em caso de acidente perfurocortante ou com respingo em pele intacta ou não, deve-se lavar a área com água e sabão, imediatamente, se contato com os olhos, irrigar os mesmos com água corrente ou solução salina, por 15 minutos, em caso de contato com nariz ou boca, deve-se enxaguar os mesmos algumas vezes com água, assoar o nariz e limpar a área afetada com água ou solução salina (ORGANIZAÇÃO MUNDIAL DA SAÚDE, 2010)

Referências

BINSWANGER, Ingrid A. et al. High prevalence of abscesses and cellulitis among community0recruited injection drug users in San Francisco. **Clinical infectious diseases**, v. 30, n. 3, p. 5790581, 2000.

Brasil. Agência Nacional de Vigilância Sanitária. **Medidas de Prevenção de Infecção Relacionada à Assistência à Saúde**. Brasília: Anvisa, 2017.

CONSELHO REGIONAL DE ENFERMAGEM DE SÃO PAULO. **Parecer nº014/2010**. Dispõe sobre o uso de luvas de procedimento para a administração de medicamentos. 2010.

D'AQUINO, Maria; DE SOUZA, Rogério Marques. Hipodermóclise ou via subcutânea. **Revista Hospital Universitário Pedro Ernesto**, v. 11, n. 2, 2012.

HUTIN, Yvan et al. Best infection control practices for intradermal, subcutaneous, and intramuscular needle injections. **Bulletin of the World Health Organization**, v. 81, p. 4910500, 2003.

MONCAIO, Ana Carolina Scarpel; FIGUEIREDO, Rosely Moralez de. Conhecimentos e práticas no uso do cateter periférico intermitente pela equipe de enfermagem. **Revista Eletrônica de Enfermagem**, v. 11, n. 3, 2009.

ORGANIZAÇÃO PAN0AMERICANA DA SAÚDE. **Uso de Luvas**. Folha Informativa,n.6, 2010. Disponível em: < https://www.paho.org/bra/index.php?option=com_docman&view=download&alias=4980folha0informativa0uso0luvas08&category_slug=seguranca0do0paciente0970&Itemid=965>. Acesso em 21 abr 2021.

O'GRADY, Naomi. et al. Guidelines for the prevention of intravascular catheter0related infections. **Clinical infectious diseases**, v. 52, n. 9, p. e1620e193, 2011.

OLIVEIRA, Tainã Clarissa Santos da Silva de et al. Preparo e administração de medicamentos por via subcutânea: os saberes da equipe de enfermagem. **Revista Enfermagem Atual In Derme**, v. 87, n. 25, 2019.

SÃO PAULO. Secretaria Municipal da Saúde. **Recomendações sobre o uso de luvas em serviços de saúde**. 2016. Disponível em: < https://www.cristofoli.com/biosseguranca/wp0content/uploads/2018/10/CVE_USAO_LUVAS.pdf>. Acesso em 21 abr 2021.

SEXSON, Kathryn; LINDAUER, Allison; HARVATH, Theresa A. Administration of subcutaneous injections. **AJN The American Journal of Nursing**, v. 117, n. 5, p. S70S10, 2017.

ORGANIZAÇÃO MUNDIAL DA SAÚDE. **Glove Use.** Information Leaflet. Patient Safety, 2009.

ORGANIZAÇÃO MUNDIAL DA SAÚDE. **WHO best practices for injections and related procedures toolkit**. Geneva, 2010.

APÊNDICE G – Questionário para Identificação das Boas Práticas com Medicações Injetáveis: versões para aplicação em contextos de saúde

QUESTIONÁRIO SOBRE BOAS PRÁTICAS COM MEDICAÇÕES INJETÁVEIS (QBMI) – VERSÃO DO AVALIADOR

Instruções de preenchimento:

Você deverá escolher uma única alternativa para cada pergunta.

Considerando as oportunidades em que as atividades descritas ocorrem na sua prática, assinale a alternativa que melhor retrate a frequência com que você realiza ou observa as mesmas.

Oportunidade deve ser compreendida como a ocorrência de determinada atividade durante sua prática, por exemplo, supervisores e docentes devem considerar a frequência com que realiza esta atividade quando esta ocorre, mesmo que esta não faça parte de suas atribuições diárias.

- <u>Sempre</u>: se esta atividade ocorre em cerca de 100% das oportunidades;
- <u>Quase sempre</u>: se esta atividade ocorre em cerca de 75% das oportunidades;
- <u>Às vezes</u>: se esta atividade ocorre em cerca de 50% das oportunidades;
- <u>Quase nunca</u>: se esta atividade ocorre em cerca de 25% das oportunidades;
- <u>Nunca</u>: se esta atividade não costuma ocorrer durante as oportunidades.

Ao final, realize a somatória dos pontos de acordo com o espaço assinalado.

A pontuação varia entre 44 e 220: **quanto mais próxima de 44, mais adequada estará a prática com injetáveis.**

<u>Atenção</u>!

A pontuação das questões abaixo deve ser invertida, caso a instituição a qual você pertence orientar o uso corriqueiro de luvas para administração de injetáveis via subcutânea e intramuscular e antissepsia de pele antes da administração de medicações via subcutânea:

23. Com que frequência realiza a antissepsia da pele do paciente com álcool a 70% para administração de medicações subcutâneas (exceto vacinas)?

32. Com que frequência utiliza luvas para administrar medicações intramusculares?

33. Com que frequência utiliza luvas para administrar medicações subcutâneas?

Para estas questões, considerar: Sempre = 1; Quase sempre= 2; Às vezes=3; Quase nunca =4; Nunca= 5.

PREPARO DO AMBIENTE	Sempre	Quase Sempre	Às Vezes	Quase nunca	Nunca
1. Com que frequência higieniza o balcão para preparo das medicações, antes do início de suas atividades, com álcool a 70% ou outras soluções padronizadas em seu serviço?	1	2	3	4	5
2. Com que frequência higieniza as bandejas usadas no preparo e administração de medicações injetáveis antes de cada uso?	1	2	3	4	5
3. Com que frequência verifica a data de validade de materiais utilizados para administração de medicamentos antes de seu uso (por exemplo, seringas, agulhas)?	1	2	3	4	5
4. Com que frequência utiliza materiais para administração de medicamentos cujas embalagens estejam danificadas (por exemplo, tampinhas de equipo, cateteres e agulhas)?	5	4	3	2	1
5. Com que frequência higieniza as mãos antes e após o preparo de medicações injetáveis?	1	2	3	4	5
**PREPARO DAS MEDICAÇÕES**	**Sempre**	**Quase Sempre**	**Às Vezes**	**Quase nunca**	**Nunca**
6. Com que frequência compartilha frascos de soros (de 100 a 1000 mL) para reconstituição de doses de medicamentos?	5	4	3	2	1
7. Com que frequência utiliza frascos multidoses para dois ou mais pacientes?	5	4	3	2	1

**8. Com que frequência realiza desinfecção com álcool a 70% das tampas de frascos multidoses antes da aspiração do medicamento a ser administrado?**	**1**	**2**	**3**	**4**	**5**
**9. Com que frequência utiliza a mesma agulha para aspirar medicações de frascos multidoses?**	**5**	**4**	**3**	**2**	**1**
**10. Com que frequência identifica os frascos multidoses disponíveis na sua unidade com data de abertura, volume de diluente (se aplicável) e nome do profissional que reconstituiu a medicação?**	**1**	**2**	**3**	**4**	**5**
**11. Com que frequência realiza limpeza de frascos e ampolas de medicação com álcool a 70% antes de aspirá-la?**	**1**	**2**	**3**	**4**	**5**
**12. Com que frequência reutiliza agulhas ou seringas para preparo de medicações do mesmo paciente?**	**5**	**4**	**3**	**2**	**1**
**13. Com que frequência reutiliza agulhas ou seringas para preparo de medicações de pacientes diferentes?**	**5**	**4**	**3**	**2**	**1**
**ADMINISTRAÇÃO MEDICAMENTOSA**	**Sempre**	**Quase Sempre**	**Às Vezes**	**Quase nunca**	**Nunca**
**14. Com que frequência realiza a salinização de cateteres venosos periféricos intermitentes?**	**1**	**2**	**3**	**4**	**5**
**15. Com que frequência higieniza as mãos, com água e sabonete antes da administração de medicação endovenosa?**	**1**	**2**	**3**	**4**	**5**
**16. Com que frequência higieniza as mãos, com solução alcoólica ou soluções padronizadas, antes da administração de medicação endovenosa?**	**1**	**2**	**3**	**4**	**5**

**17. Com que frequência utiliza a mesma seringa para salinização de cateteres venosos periféricos em diferentes pacientes?**	**5**	**4**	**3**	**2**	**1**
**18. Com que frequência reutiliza a mesma tampinha, de fechamento de dupla via, em cateteres venosos periféricos?**	**1**	**2**	**3**	**4**	**5**
**19. Com que frequência armazena tampinha de cateter venoso periférico em mesas de cabeceira, bolsos de jaleco/avental ou outros locais para posterior reutilização desta?**	**5**	**4**	**3**	**2**	**1**
**20. Com que frequência realiza a desobstrução de cateteres venosos periféricos com a infusão forçada de soro fisiológico?**	**5**	**4**	**3**	**2**	**1**
**21. Com que frequência realiza a desobstrução de cateteres venosos periféricos com a infusão forçada de solução de heparina?**	**5**	**4**	**3**	**2**	**1**
**22. Com que frequência realiza desinfecção de conectores (dupla via, torneirinha ou injetor lateral de equipo) com álcool a 70% antes de administrar os medicamentos?**	**1**	**2**	**3**	**4**	**5**
**23. Com que frequência realiza a antissepsia da pele do paciente com álcool a 70% para administração de medicações subcutâneas (exceto vacinas)?**	**1**	**2**	**3**	**4**	**5**
**24. Com que frequência realiza antissepsia da pele do paciente com álcool a 70% para administração de medicações intramusculares (exceto vacinas) ?**	**1**	**2**	**3**	**4**	**5**
**25. Com que frequência toca a área da pele do paciente, onde já realizou antissepsia, antes da administração do medicamento injetável?**	**5**	**4**	**3**	**2**	**1**
**26. Com que frequência utiliza a mesma seringa, trocando apenas a agulha, para administração de medicamentos no mesmo paciente?**	**5**	**4**	**3**	**2**	**1**

**27. Com que frequência utiliza a mesma seringa, trocando apenas a agulha, para administração de medicamentos em pacientes diferentes?**	**5**	**4**	**3**	**2**	**1**
**28. Com que frequência realiza lavagem do acesso venoso periférico intermitente com SF 0,9% após administração de medicamentos?**	**1**	**2**	**3**	**4**	**5**
**29. Com que frequência reutiliza materiais descartáveis como tampinhas de equipos ou dupla via?**	**5**	**4**	**3**	**2**	**1**
**30. Com que frequência recebe treinamento para manipulação de agulhas e cateteres com dispositivos de segurança?**	**1**	**2**	**3**	**4**	**5**
**31. Com que frequência realiza administração de medicações com lesões de pele nas mãos?**	**5**	**4**	**3**	**2**	**1**
**32. Com que frequência utiliza luvas para administrar medicações intramusculares?**	**5**	**4**	**3**	**2**	**1**
**33. Com que frequência utiliza luvas para administrar medicações subcutâneas?**	**5**	**4**	**3**	**2**	**1**
**34. Com que frequência utiliza luvas para administrar medicamentos por via endovenosa?**					
**35. Com que frequência utiliza luvas para administrar medicamentos por via endovenosa apenas para paciente sabidamente acometidos com vírus como HIV, hepatites B ou C?**	**5**	**4**	**3**	**2**	**1**
**36. Com que frequência realiza curativo estéril para fixação/manutenção dos cateteres venosos periféricos?**	**1**	**2**	**3**	**4**	**5**
**37. Com que frequência realiza troca de acesso venoso periférico, conforme padronização do serviço, antes do aparecimento de sinais de flebite local?**	**1**	**2**	**3**	**4**	**5**

CUIDADOS APÓS ADMINISTRAÇÃO MEDICAMENTOSA	Sempre	Quase Sempre	Às Vezes	Quase nunca	Nunca
**38. Com que frequência reencapa agulhas após realizar medicações?**	**5**	**4**	**3**	**2**	**1**
**39. Com que frequência higieniza as mãos antes e após o uso de luvas para administração de medicamentos?**	**1**	**2**	**3**	**4**	**5**
**40. Com que frequência transporta materiais usados, como seringas e agulhas, desprotegidos até o local de descarte?**	**5**	**4**	**3**	**2**	**1**
**41. Com que frequência separa a agulha do corpo da seringa para o descarte após medicar pacientes?**	**5**	**4**	**3**	**2**	**1**
**42. Com que frequência procura informar-se sobre sua situação vacinal contra doenças potencialmente passíveis de transmissão ocupacional, como a hepatite B?**	**1**	**2**	**3**	**4**	**5**
**43. Com que frequência costuma informar-se sobre o fluxo de atendimento pós exposição percutânea com material perfurocortante no seu local de trabalho?**	**1**	**2**	**3**	**4**	**5**
**44. Com que frequência observa e/ou toma conhecimento de que pessoas da equipe de enfermagem após sofrer acidente com perfurocortante espremem a área afetada e colocam álcool a 70% para desinfetar a região?**	**5**	**4**	**3**	**2**	**1**
**Pontuação Total:**					

QUESTIONÁRIO SOBRE BOAS PRÁTICAS COM MEDICAÇÕES INJETÁVEIS (QBMI) – VERSÃO DO AVALIADOR

Instruções de preenchimento:

Você deverá escolher uma única alternativa para cada pergunta.

Considerando as oportunidades em que as atividades descritas ocorrem na sua prática, assinale a alternativa que melhor retrate a frequência com que você realiza ou observa as mesmas.

Oportunidade deve ser compreendida como a ocorrência de determinada atividade durante sua prática, por exemplo, supervisores e docentes devem considerar a frequência com que realiza esta atividade quando esta ocorre, mesmo que esta não faça parte de suas atribuições diárias.

- Sempre: se esta atividade ocorre em cerca de 100% das oportunidades;
- Quase sempre: se esta atividade ocorre em cerca de 75% das oportunidades;
- Às vezes: se esta atividade ocorre em cerca de 50% das oportunidades;
- Quase nunca: se esta atividade ocorre em cerca de 25% das oportunidades;
- Nunca: se esta atividade não costuma ocorrer durante as oportunidades.

Ao final, será realizada a somatória dos pontos de acordo com o espaço assinalado.

A pontuação varia entre 44 e 220: **quanto mais próxima de 44, mais adequada estará a prática com injetáveis.**

PREPARO DO AMBIENTE	Sempre	Quase Sempre	Às Vezes	Quase nunca	Nunca
1. Com que frequência higieniza o balcão para preparo das medicações, antes do início de suas atividades, com álcool a 70% ou outras soluções padronizadas em seu serviço?					
2. Com que frequência higieniza as bandejas usadas no preparo e administração de medicações injetáveis antes de cada uso?					
3. Com que frequência verifica a data de validade de materiais utilizados para administração de medicamentos antes de seu uso (por exemplo, seringas, agulhas)?					
4. Com que frequência utiliza materiais para administração de medicamentos cujas embalagens estejam danificadas (por exemplo, tampinhas de equipo, cateteres e agulhas)?					
5. Com que frequência higieniza as mãos antes e após o preparo de medicações injetáveis?					
**PREPARO DAS MEDICAÇÕES**	**Sempre**	**Quase Sempre**	**Às Vezes**	**Quase nunca**	**Nunca**
6. Com que frequência compartilha frascos de soros (de 100 a 1000 mL) para reconstituição de doses de medicamentos?					
7. Com que frequência utiliza frascos multidoses para dois ou mais pacientes?					

**8. Com que frequência realiza desinfecção com álcool a 70% das tampas de frascos multidoses antes da aspiração do medicamento a ser administrado?**					
**9. Com que frequência utiliza a mesma agulha para aspirar medicações de frascos multidoses?**					
**10. Com que frequência identifica os frascos multidoses disponíveis na sua unidade com data de abertura, volume de diluente (se aplicável) e nome do profissional que reconstituiu a medicação?**					
**11. Com que frequência realiza limpeza de frascos e ampolas de medicação com álcool a 70% antes de aspirá-la?**					
**12. Com que frequência reutiliza agulhas ou seringas para preparo de medicações do mesmo paciente?**					
**13. Com que frequência reutiliza agulhas ou seringas para preparo de medicações de pacientes diferentes?**					
**ADMINISTRAÇÃO MEDICAMENTOSA**	**Sempre**	**Quase Sempre**	**Às Vezes**	**Quase nunca**	**Nunca**
**14. Com que frequência realiza a salinização de cateteres venosos periféricos intermitentes?**					
**15. Com que frequência higieniza as mãos, com água e sabonete antes da administração de medicação endovenosa?**					
**16. Com que frequência higieniza as mãos, com solução alcoólica ou soluções padronizadas, antes da administração de medicação endovenosa?**					

**17. Com que frequência utiliza a mesma seringa para salinização de cateteres venosos periféricos em diferentes pacientes?**					
**18. Com que frequência reutiliza a mesma tampinha, de fechamento de dupla via, em cateteres venosos periféricos?**					
**19. Com que frequência armazena tampinha de cateter venoso periférico em mesas de cabeceira, bolsos de jaleco/avental ou outros locais para posterior reutilização desta?**					
**20. Com que frequência realiza a desobstrução de cateteres venosos periféricos com a infusão forçada de soro fisiológico?**					
**21. Com que frequência realiza a desobstrução de cateteres venosos periféricos com a infusão forçada de solução de heparina?**					
**22. Com que frequência realiza desinfecção de conectores (dupla via, torneirinha ou injetor lateral de equipo) com álcool a 70% antes de administrar os medicamentos?**					
**23. Com que frequência realiza a antissepsia da pele do paciente com álcool a 70% para administração de medicações subcutâneas (exceto vacinas)?**					
**24. Com que frequência realiza antissepsia da pele do paciente com álcool a 70% para administração de medicações intramusculares (exceto vacinas) ?**					
**25. Com que frequência toca a área da pele do paciente, onde já realizou antissepsia, antes da administração do medicamento injetável?**					
**26. Com que frequência utiliza a mesma seringa, trocando apenas a agulha, para administração de medicamentos no mesmo paciente?**					

**27. Com que frequência utiliza a mesma seringa, trocando apenas a agulha, para administração de medicamentos em pacientes diferentes?**					
**28. Com que frequência realiza lavagem do acesso venoso periférico intermitente com SF 0,9% após administração de medicamentos?**					
**29. Com que frequência reutiliza materiais descartáveis como tampinhas de equipos ou dupla via?**					
**30. Com que frequência recebe treinamento para manipulação de agulhas e cateteres com dispositivos de segurança?**					
**31. Com que frequência realiza administração de medicações com lesões de pele nas mãos?**					
**32. Com que frequência utiliza luvas para administrar medicações intramusculares?**					
**33. Com que frequência utiliza luvas para administrar medicações subcutâneas?**					
**34. Com que frequência utiliza luvas para administrar medicamentos por via endovenosa?**					
**35. Com que frequência utiliza luvas para administrar medicamentos por via endovenosa apenas para paciente sabidamente acometidos com vírus como HIV, hepatites B ou C?**					
**36. Com que frequência realiza curativo estéril para fixação/manutenção dos cateteres venosos periféricos?**					
**37. Com que frequência realiza troca de acesso venoso periférico, conforme padronização do serviço, antes do aparecimento de sinais de flebite local?**					

CUIDADOS APÓS ADMINISTRAÇÃO MEDICAMENTOSA	Sempre	Quase Sempre	Às Vezes	Quase nunca	Nunca
38. Com que frequência reencapa agulhas após realizar medicações?					
39. Com que frequência higieniza as mãos antes e após o uso de luvas para administração de medicamentos?					
40. Com que frequência transporta materiais usados, como seringas e agulhas, desprotegidos até o local de descarte?					
41. Com que frequência separa a agulha do corpo da seringa para o descarte após medicar pacientes?					
42. Com que frequência procura informar-se sobre sua situação vacinal contra doenças potencialmente passíveis de transmissão ocupacional, como a hepatite B?					
43. Com que frequência costuma informar-se sobre o fluxo de atendimento pós exposição percutânea com material perfurocortante no seu local de trabalho?					
44. Com que frequência observa e/ou toma conhecimento de que pessoas da equipe de enfermagem após sofrer acidente com perfurocortante espremem a área afetada e colocam álcool a 70% para desinfetar a região?					
Pontuação Total:					

APÊNDICE H – Questionário para Identificação das Boas Práticas com Medicações Injetáveis disponibilizado na plataforma SurveyMonkey aos participantes da pesquisa

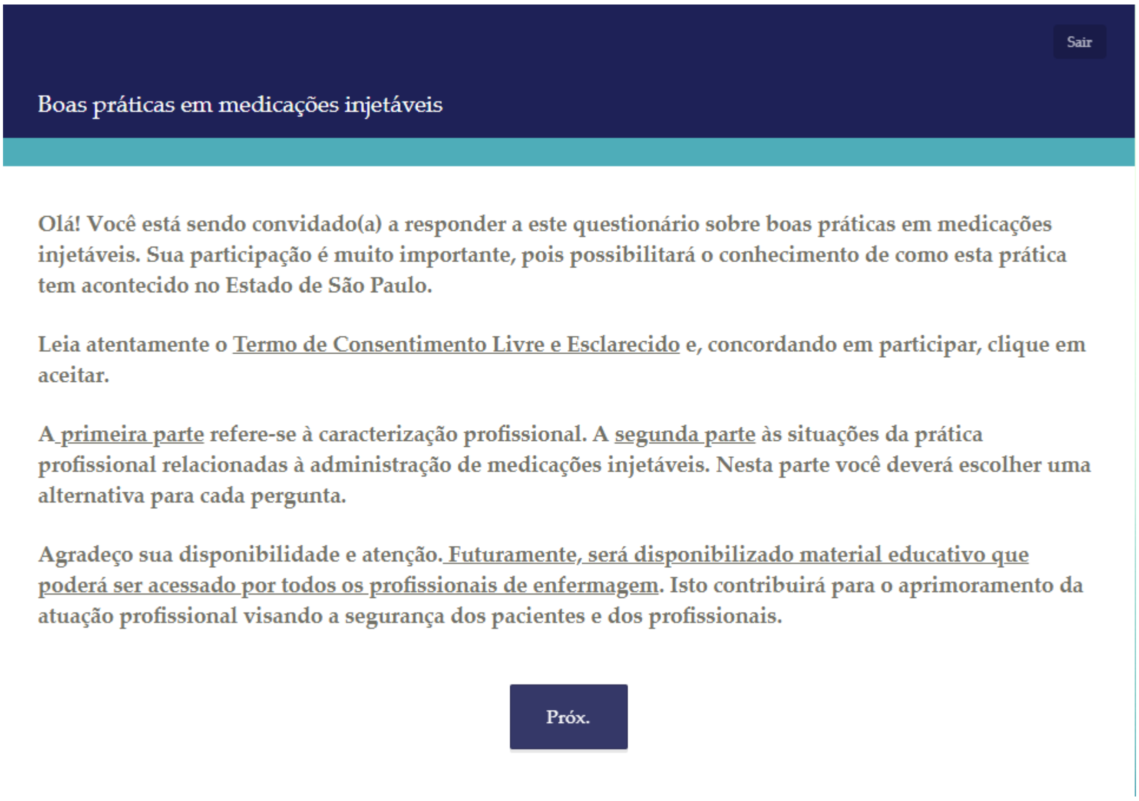

Sair

Boas práticas em medicações injetáveis

Olá! Você está sendo convidado(a) a responder a este questionário sobre boas práticas em medicações injetáveis. Sua participação é muito importante, pois possibilitará o conhecimento de como esta prática tem acontecido no Estado de São Paulo.

Leia atentamente o Termo de Consentimento Livre e Esclarecido e, concordando em participar, clique em aceitar.

A primeira parte refere-se à caracterização profissional. A segunda parte às situações da prática profissional relacionadas à administração de medicações injetáveis. Nesta parte você deverá escolher uma alternativa para cada pergunta.

Agradeço sua disponibilidade e atenção. Futuramente, será disponibilizado material educativo que poderá ser acessado por todos os profissionais de enfermagem. Isto contribuirá para o aprimoramento da atuação profissional visando a segurança dos pacientes e dos profissionais.

Próx.

Sair

Boas práticas em medicações injetáveis

TERMO DE CONSENTIMENTO LIVRE E ESCLARECIDO

(Resolução 466/2012 do CNS)

BOAS PRÁTICAS EM MEDICAÇÕES INJETÁVEIS: INTERVENÇÃO EDUCATIVA PARA A EQUIPE DE ENFERMAGEM NO ESTADO DE SÃO PAULO

*

Caro(a) Senhor(a), o Sr(a) está sendo convidado(a) para participar da pesquisa "Boas práticas em medicações injetáveis: intervenção educativa para a equipe de enfermagem no estado de São Paulo", que tem como objetivo desenvolver e validar uma estratégia educativa para profissionais de enfermagem visando às boas práticas na administração de medicações injetáveis. O(a) senhor(a) foi selecionado(a) por ser profissional da área da enfermagem e possuir registro ativo no conselho regional do estado de São Paulo.

Sua participação é voluntária, isto é, a qualquer momento o(a) senhor(a) pode desistir de participar e retirar seu consentimento. A sua recusa não trará nenhum prejuízo na sua relação com o pesquisador ou com a instituição que forneceu os dados. Em caso de esclarecimentos quanto à pesquisa, poderá entrar em contato com a pesquisadora pelo telefone ou e-mail abaixo deste termo, a qualquer momento.

A coleta de dados será composta por este questionário validado por especialistas, enviado eletronicamente, o qual aborda questões referentes à sua rotina de trabalho envolvendo a administração de medicações injetáveis e um breve questionário pessoal no qual deve informar sua categoria profissional, nível de instrução, tempo de atuação na área e de serviço em que atua. O tempo utilizado para coleta dos dados será de aproximadamente trinta minutos.

Suas respostas serão tratadas de forma anônima e confidencial, desta forma, não será divulgado seu nome em qualquer fase do estudo. Quando for necessário exemplificar determinada situação, sua privacidade será assegurada. Os dados coletados poderão ter seus resultados divulgados em eventos, revistas e/ou trabalhos científicos.

Os riscos presentes em sua participação são mínimos, e frente a qualquer desconforto ou cansaço o questionário poderá deixar de ser respondido, sem nenhum prejuízo ou perda para você, e posteriormente retomado, caso seja de seu interesse.

Você não terá nenhum custo ou compensação financeira ao participar do estudo. Contudo, destaca-se que sua participação auxiliará a conhecer o contexto atual das boas práticas de medicações injetáveis no estado de São Paulo, o qual embasará uma estratégia educativa a ser divulgado eletronicamente, ao qual o(a) senhor(a) terá acesso assim que publicado.

O(a) senhor(a) receberá uma via assinada deste termo, por você e pelo pesquisador, onde consta o telefone e o endereço do pesquisador principal.

Declaro que entendi os objetivos, riscos e benefícios de minha participação na pesquisa e concordo em participar.

O pesquisador me informou que o projeto foi aprovado pelo Comitê de Ética em Pesquisa em Seres Humanos da UFSCar que funciona na Pró-Reitoria de Pesquisa da Universidade Federal de São Carlos, localizada na Rodovia Washington Luiz, Km. 235 - Caixa Postal 676 - CEP 13.565-905 - São Carlos - SP – Brasil. Fone (16) 3351-8028. Endereço eletrônico: cephumanos@ufscar.br

Endereço para contato

Pesquisadora Responsável: Camila Eugenia Roseira

Endereço: UFSCar, Rod. Washington Luís, Km 235, São Carlos/SP

Contato telefônico: (16) 3351-8338. e-mail: roseira.camila@gmail.com

- ◯ Aceito
- ◯ Não aceito

Anter. Próx.

Desenvolvido pela SurveyMonkey

Sair

Boas práticas em medicações injetáveis

Caracterização Profissional

Nesta primeira parte, você é convidado a contar um pouco sobre você.

* Categoria profissional

- [ ] Enfermeiro
- [ ] Auxiliar de enfermagem
- [ ] Técnico de enfermagem
- [ ] Outro (especifique)

* Qual a sua idade?

* Qual seu maior grau de instrução?

- [ ] Graduação
- [ ] Nível médio
- [ ] Pós graduação
- [ ] Outro (especifique)

* Há quanto tempo atua na área?

- ( ) 0-5 anos
- ( ) 21-30 anos
- ( ) 6-10 anos
- ( ) 30 anos ou mais
- ( ) 11-20 anos

* Em qual tipo de serviço você atua? Pode assinalar mais de uma resposta.

- [ ] Hospital
- [ ] Escola técnica de enfermagem
- [ ] Ambulatório/Centro de especialidades
- [ ] Faculdades/Universidades
- [ ] Unidades de Pronto Atendimento
- [ ] Atendimento pré-hospitalar
- [ ] Atenção Primária à Saúde/Atenção Básica
- [ ] Outro (especifique)

* Sua atividade principal na enfermagem é de caráter (pode assinalar mais de uma resposta):

- [ ] Assistencial
- [ ] Ensino
- [ ] Gerencial/Administrativa
- [ ] Outro (especifique)

Anter. | Próx.

Desenvolvido pela

Sair

Boas práticas em medicações injetáveis

PARTE 2

Esta parte se refere às situações da prática da administração de medicações injetáveis. Você deverá escolher uma única alternativa para cada pergunta.

Considerando as oportunidades em que as atividades descritas ocorrem na sua prática, assinale a alternativa que melhor retrate a frequência com que você realiza ou observa as mesmas.

Atenção: oportunidade deve ser compreendida como a ocorrência de determinada atividade durante sua prática, por exemplo, supervisores e docentes devem considerar a frequência com que realiza esta atividade quando esta ocorre, mesmo que esta não faça parte de suas atribuições diárias.

· **Sempre**: se esta atividade ocorre em cerca de 100% das oportunidades.

· **Quase sempre**: se esta atividade ocorre em cerca de 75% das oportunidades.

· **Às vezes**: se esta atividade ocorre em cerca de 50% das oportunidades.

· **Quase nunca**: se esta atividade ocorre em cerca de 25% das oportunidades.

· **Nunca**: se esta atividade não costuma ocorrer durante as oportunidades.

Anter. Próx.

Desenvolvido pela
SurveyMonkey
Veja como é fácil criar um questionário.

Sair

Boas práticas em medicações injetáveis

ORGANIZAÇÃO DO AMBIENTE

Esta seção refere-se às ações como higienização de bancadas, bandejas, identificação de materiais, para o posterior preparo das medicações injetáveis. Aqui, você deve selecionar uma resposta para cada item.

* Com que frequência higieniza o balcão para preparo das medicações, antes do início de suas atividades, com álcool a 70% ou outras soluções padronizadas em seu serviço?

- Sempre
- Quase sempre
- Às vezes
- Quase nunca
- Nunca

* Com que frequência higieniza as bandejas usadas no preparo e administração de medicações injetáveis antes de cada uso?

- Sempre
- Quase sempre
- Às vezes
- Quase nunca
- Nunca

* Com que frequência verifica a data de validade de materiais utilizados para administração de medicamentos antes de seu uso (por exemplo, seringas, agulhas)?

- Sempre
- Quase sempre
- Às vezes
- Quase nunca
- Nunca

* Com que frequência utiliza materiais para administração de medicamentos cujas embalagens estejam danificadas (por exemplo, tampinhas de equipo, cateteres e agulhas)?

- Sempre
- Quase sempre
- Às vezes
- Quase nunca
- Nunca

* Com que frequência higieniza as mãos antes e após o preparo de medicações injetáveis?

- Sempre
- Quase sempre
- Às vezes
- Quase nunca
- Nunca

Anter. Próx.

Desenvolvido pela
SurveyMonkey
Veja como é fácil criar um questionário.

Sair

Boas práticas em medicações injetáveis

PREPARO DAS MEDICAÇÕES

Esta seção refere-se à manipulação de materiais e medicamentos utilizados para medicações injetáveis, como frascos de soros, medicações multidoses e agulhas. Aqui, você deve selecionar uma resposta para cada item.

* Com que frequência compartilha frascos de soros (de 100 a 1000 mL) para reconstituição de doses de medicamentos?

- Sempre
- Quase sempre
- Às vezes
- Quase nunca
- Nunca

* Com que frequência utiliza frascos multidoses para dois ou mais pacientes?

- Sempre
- Quase sempre
- Às vezes
- Quase nunca
- Nunca

* Com que frequência realiza desinfecção com álcool a 70% das tampas de frascos multidoses antes da aspiração do medicamento a ser administrado?

- Sempre
- Quase sempre
- Às vezes
- Quase nunca
- Nunca

* Com que frequência utiliza a mesma agulha para aspirar medicações de frascos multidoses?

- Sempre
- Quase sempre
- Às vezes
- Quase nunca
- Nunca

* Com que frequência identifica os frascos multidoses disponíveis na sua unidade com data de abertura, volume de diluente (se aplicável) e nome do profissional que reconstituiu a medicação?

- Sempre
- Quase sempre
- Às vezes
- Quase nunca
- Nunca

* Com que frequência realiza limpeza de frascos e ampolas de medicação com álcool a 70% antes de aspirá-la?

- Sempre
- Quase sempre
- Às vezes
- Quase nunca
- Nunca

* Com que frequência reutiliza agulhas ou seringas para preparo de medicações do mesmo paciente?

- Sempre
- Quase Sempre
- Às vezes
- Quase nunca
- Nunca

* Com que frequência reutiliza agulhas ou seringas para preparo de medicações de pacientes diferentes?

- Sempre
- Quase sempre
- Às vezes
- Quase nunca
- Nunca

Anter. Próx.

Sair

Boas práticas em medicações injetáveis

ADMINISTRAÇÃO MEDICAMENTOSA

Esta seção refere-se à administração de medicamentos propriamente dita, desde a higienização prévia das mãos à salinização de cateteres venosos periféricos. Aqui, você deve selecionar uma resposta para cada item.

* Com que frequência realiza a salinização de cateteres venosos periféricos intermitentes?

- Sempre
- Quase sempre
- Às vezes
- Quase nunca
- Nunca

* Com que frequência higieniza as mãos, com água e sabonete antes da administração de medicação endovenosa?

- Sempre
- Quase sempre
- Às vezes
- Quase nunca
- Nunca

* Com que frequência higieniza as mãos, com solução alcoólica ou soluções padronizadas, antes da administração de medicação endovenosa?

- Sempre
- Quase sempre
- Às vezes
- Quase nunca
- Nunca

* Com que frequência utiliza a mesma seringa para salinização de cateteres venosos periféricos em diferentes pacientes?

- Sempre
- Quase sempre
- Às vezes
- Quase nunca
- Nunca

* Com que frequência reutiliza a mesma tampinha, de fechamento de dupla via, em cateteres venosos periféricos?

- Sempre
- Quase sempre
- Às vezes
- Quase nunca
- Nunca

* Com que frequência armazena tampinha de cateter venoso periférico em mesas de cabeceira, bolsos de jaleco/avental ou outros locais para posterior reutilização desta?

- ◯ Sempre
- ◯ Quase sempre
- ◯ Às vezes
- ◯ Quase Nunca
- ◯ Nunca

* Com que frequência realiza a desobstrução de cateteres venosos periféricos com a infusão forçada de soro fisiológico?

- ◯ Sempre
- ◯ Quase sempre
- ◯ Às vezes
- ◯ Quase nunca
- ◯ Nunca

* Com que frequência realiza a desobstrução de cateteres venosos periféricos com a infusão forçada de solução de heparina?

- ◯ Sempre
- ◯ Quase sempre
- ◯ Às vezes
- ◯ Quase nunca
- ◯ Nunca

* Com que frequência realiza desinfecção de conectores (dupla via, torneirinha ou injetor lateral de equipo) com álcool a 70% antes de administrar os medicamentos?

- ◯ Sempre
- ◯ Quase sempre
- ◯ Às vezes
- ◯ Quase nunca
- ◯ Nunca

* Com que frequência reutiliza a mesma seringa para salinização de acesso venoso periférico de pacientes diferentes?

- ◯ Sempre
- ◯ Quase sempre
- ◯ Às vezes
- ◯ Quase nunca
- ◯ Nunca

* Com que frequência realiza a antissepsia da pele do paciente com álcool a 70% para administração de medicações subcutâneas (exceto vacinas)?

- ◯ Sempre
- ◯ Quase sempre
- ◯ Às vezes
- ◯ Quase nunca
- ◯ Nunca

* Com que frequência realiza antissepsia da pele do paciente com álcool a 70% para administração de medicações intramusculares (exceto vacinas) ?

- Sempre
- Quase sempre
- Às vezes
- Quase nunca
- Nunca

* Com que frequência toca a área da pele do paciente, onde já realizou antissepsia, antes da administração do medicamento injetável?

- Sempre
- Quase sempre
- Às vezes
- Quase nunca
- Nunca

* Com que frequência utiliza a mesma seringa, trocando apenas a agulha,para administração de medicamentos no mesmo paciente?

- Sempre
- Quase sempre
- Às vezes
- Quase nunca
- Nunca

* Com que frequência utiliza a mesma seringa, trocando apenas a agulha, para administração de medicamentos em pacientes diferentes?

- Sempre
- Quase sempre
- Às vezes
- Quase nunca
- Nunca

* Com que frequência realiza lavagem do acesso venoso periférico intermitente com SF 0,9% após administração de medicamentos?

- Sempre
- Quase sempre
- Às vezes
- Quase nunca
- Nunca

* Com que frequência reutiliza materiais descartáveis como tampinhas de equipos ou dupla via?

- Sempre
- Quase sempre
- Às vezes
- Quase nunca
- Nunca

* Com que frequência recebe treinamento para manipulação de agulhas e cateteres com dispositivos de segurança?

- Sempre
- Quase sempre
- Às vezes
- Quase nunca
- Nunca

* Com que frequência realiza administração de medicações com lesões de pele nas mãos?

- Sempre
- Quase sempre
- Às vezes
- Quase nunca
- Nunca

* Com que frequência utiliza luvas para administrar medicações intramusculares?

- Sempre
- quase sempre
- Às vezes
- Quase nunca
- Nunca

* Com que frequência utiliza luvas para administrar medicações subcutâneas?

- Sempre
- Quase sempre
- Às vezes
- Quase nunca
- Nunca

* Com que frequência utiliza luvas para administrar medicamentos por via endovenosa?

- Sempre
- Quase sempre
- Às vezes
- Quase nunca
- Nunca

* Com que frequência utiliza luvas para administrar medicamentos por via endovenosa apenas para paciente sabidamente acometidos com vírus como HIV, hepatites B ou C?

- Sempre
- Quase sempre
- Às vezes
- Quase nunca
- Nunca

* Com que frequência realiza curativo estéril para fixação/manutenção dos cateteres venosos periféricos?

- Sempre
- Quase sempre
- Às vezes
- Quase nunca
- Nunca

* Com que frequência realiza troca de acesso venoso periférico, conforme padronização do serviço, antes do aparecimento de sinais de flebite local?

- Sempre
- Quase sempre
- Às vezes
- Quase nunca
- Nunca

Anter. Próx.

Desenvolvido pela
SurveyMonkey
Veja como é fácil criar um questionário.

Sair

Boas práticas em medicações injetáveis

CUIDADOS APÓS ADMINISTRAÇÃO MEDICAMENTOSA

Esta seção refere-se às situações pós administração de medicações injetáveis, desde a higienização das mãos até o transporte de resíduos gerados. Aqui, você deve selecionar uma resposta para cada item.

* Com que frequência reencapa agulhas após realizar medicações?

- Sempre
- Quase sempre
- Às vezes
- Quase nunca
- Nunca

* Com que frequência higieniza as mãos antes e após o uso de luvas para administração de medicamentos?

- Sempre
- Quase sempre
- Às vezes
- Quase nunca
- Nunca

* Com que frequência transporta materiais usados, como seringas e agulhas, desprotegidos até o local de descarte?

- Sempre
- Quase sempre
- Às vezes
- Quase nunca
- Nunca

* Com que frequência separa a agulha do corpo da seringa para o descarte após medicar pacientes?

- Sempre
- Quase sempre
- Às vezes
- Quase nunca
- Nunca

* Com que frequência procura informar-se sobre sua situação vacinal contra doenças potencialmente passíveis de transmissão ocupacional, como a hepatite B?

- Sempre
- Quase sempre
- Às vezes
- Quase nunca
- Nunca

* Com que frequência costuma informar-se sobre o fluxo de atendimento pós exposição percutânea com material perfurocortante no seu local de trabalho?

- ◯ Sempre
- ◯ Quase sempre
- ◯ Às vezes
- ◯ Quase nunca
- ◯ Nunca

* Com que frequência observa e/ou toma conhecimento de que pessoas da equipe de enfermagem após sofrer acidente com perfurocortante espremem a área afetada e colocam álcool a 70% para desinfetar a região?

- ◯ Sempre
- ◯ Quase sempre
- ◯ Às vezes
- ◯ Quase nunca
- ◯ Nunca

Anter. Próx.

Desenvolvido pela
SurveyMonkey
Veja como é fácil criar um questionário.

Sair

Boas práticas em medicações injetáveis

VOCÊ FINALIZOU ESTE QUESTIONÁRIO!

OBRIGADA PELA SUA PARTICIPAÇÃO

Anter. Concluído

Desenvolvido pela
SurveyMonkey
Veja como é fácil criar um questionário.

APÊNDICE I – Texto-base que subsidiou o conteúdo do curso "Boas Práticas com Injetáveis: ações para o controle de infecção"

A prática segura com injetáveis requer planejamento de ações que transcendem o momento da realização de uma injeção, seja ela intramuscular, subcutânea ou endovenosa, visto que demanda cuidados com o ambiente em que serão preparadas, a seleção de insumos necessários e a adesão a comportamentos já amplamente divulgados em áreas da saúde, como o uso de luvas e higienização das mãos.

Mas, quando e por que aplicar estes cuidados? Em quais etapas as mãos devem ser higienizadas? Qual o risco presente em realizar uma administração injetável com insumos que não tem garantia quanto a sua esterilidade e validade? Qual o motivo de realizar o preparo e a administração de injetáveis de acordo com o que sugerem as diretrizes da Organização Mundial da Saúde e Agência Nacional de Vigilância Sanitária? A prática segura protege a quem? Ao usuário do serviço de saúde? Ao profissional ou estudante durante seu estágio e práticas?

Estudante ou profissional da área: é preciso pautar a conduta em diretrizes e recomendações pertinentes para o controle microbiológico e prevenção de infecções, visando a sua segurança, a do usuário do sistema de saúde e a de terceiros.

Pensando no controle do ambiente, recomenda-se a manipulação dos insumos e soluções estéreis em uma superfície limpa (seja balcão ou a própria bandeja) (ORGANIZAÇÃO MUNDIAL DA SAÚDE, 2010). Esta limpeza pode ser feita com solução alcoólica a 70% e é importante a sua utilização, especialmente, em caso de sujidade por fluidos como sangue, pois, sugere-se a eficácia mesmo em presença de material orgânico (GRAZIANO et al., 2012). Ah, aguarde a secagem espontânea e completa da superfície antes de utilizá-la como base para o preparo de injetáveis, garantindo, assim, o tempo de ação para que seja eficiente (ORGANIZAÇÃO MUNDIAL DA SAÚDE, 2010)!

Sobre os insumos utilizados, como as seringas e agulhas, para a realização de medicações injetáveis, independentemente da via, devem estar dentro do prazo de validade, com embalagem íntegra para serem utilizados para um único procedimento e em um único usuário do sistema de saúde (ORGANIZAÇÃO MUNDIAL DA SAÚDE, 2010).

A cada novo procedimento, novos dispositivos devem ser utilizados,

garantindo sua esterilidade. Esta medida é muito importante para a segurança do paciente, pois, a desatenção a estes eventos pode viabilizar a contaminação da solução a ser administrada, ocasionando danos como infecções, abcessos, flebites, dentre outros (ORGANIZAÇÃO MUNDIAL DA SAÚDE, 2010).

Ainda sobre os insumos utilizados para o preparo de medicações, é preciso atentar-se, também, para o tipo dos frascos de soluções utilizadas para a reconstituição de doses. Por exemplo, é preconizado que a diluição de medicamentos ocorra a partir de doses únicas para este fim e que não sejam compartilhadas bolsas de solução para reconstituição de doses medicamentosas (como aquelas com solução fisiológica 0,9% contendo 100 ml). Uma vez que caso ocorra contaminação da solução, todas doses a partir de então estarão contaminadas também podendo ocasionar danos ao paciente (BRASIL, 2017).

Já os frascos multidoses requerem muito cuidado e devem ser utilizados apenas quando não há alternativa e, se possível, para pacientes individualizados (ou seja, lembre-se de identificar sempre o rótulo, com nome do usuário, data e horário de abertura, volume de reconstituição e assine, assim você garante que será utilizada no tempo de validade estabelecido pelo fabricante), mantendo técnica asséptica durante o manuseio e utilizando novos dispositivos sempre que for necessário aspirar a solução (portanto, utilize sempre, nova seringa e agulha a cada aspiração medicamentosa, mesmo que seja dose complementar para o mesmo usuário), após a desinfecção do septo (cuidado a ser ampliado para frascos unidose, também, assim não há risco de contaminar a solução a ser aspirada). Em caso de dúvidas sobre esterilidade ou a forma de manuseio anterior, este frasco deve ser descartado (ORGANIZAÇÃO MUNDIAL DA SAÚDE, 2010)!

Quanto à higienização das mãos, embora haja consciência de sua relevância e comprovação científica para remoção de microrganismos prevenindo e reduzindo a transmissão destes a partir do emprego de água e sabão ou mesmo solução alcoólica (uma substitui a outra, a escolha pode respeitar critérios pessoais, a considerar fatores como tempo de ação, visto que o uso de álcool demanda metade do tempo da higienização das mãos com água e sabão, conforto a partir da qualidade da solução empregada e recursos disponíveis no local, como a beira leito). Os momentos para realização da higienização das mãos, para a prática com injetáveis, são sumarizados a seguir: antes e após o preparo de medicações injetáveis, após uso de luvas (o uso de luvas é complementar à higienização das mãos, não a substitui, já que o uso de luvas

protege apenas o profissional do risco de exposição aos fluidos corporais do usuário ou potenciais microrganismos colonizadores deste, enquanto a higienização das mãos protege a ambos), antes e após a inserção de cateteres ou agulhas, ou mesmo ao manipular dispositivos ou trocar fixadores de cateteres: trata-se de um mecanismo de redução de exposição e prevenção de patógenos! Considere que o uso de luvas não impede que o profissional ou estudante entre em contato com microrganismos, uma vez que microfuros podem estar presentes nas luvas, ou se contamine durante a remoção das luvas, contudo, estes microrganismos são passíveis de remoção pela higienização das mãos que sucederá o procedimento e retirada das luvas (BRASIL, 2017; ORGANIZAÇÃO MUNDIAL DA SAÚDE, 2010).

Ao administrar medicações, independentemente da via, é preciso certificar-se de que a seringa e agulha a serem utilizadas não foram utilizadas em outros pacientes (ORGANIZAÇÃO MUNDIAL DA SAÚDE, 2010), anteriormente: pode parecer um cuidado trivial de ser mencionado, contudo, destaca-se que ao compartilhar seringas entre pacientes por desconhecimento do risco inerente a esta prática insegura, no Texas, houve um surto de hepatite B (VAN TUONG et al., 2017). O reuso de dispositivos é proibido mesmo quando se tratar do mesmo paciente. Deve-se manter o mesmo cuidado quanto ao manuseio asséptico de conectores, como as famosas torneirinhas e oclusores de dânulas, que, antes da manipulação devem passar por desinfecção por solução alcoólica a 70% com tempo de contato entre 5 e 15 segundos e que não devem ser reutilizadas: priorize realizar a troca destes a cada manipulação da via endovenosa (ORGANIZAÇÃO MUNDIAL DA SAÚDE, 2010) .

O uso de luvas é uma importante estratégia para a prevenção de contato a fluidos corporais como o sangue, e seu uso deve ser associado a uma avaliação crítica do profissional ou estudante, pois, é uma proteção física para ele: visto alto risco de exposição, é imprescindível a adesão a este equipamento de segurança para administração de injeções endovenosas (ORGANIZAÇÃO MUNDIAL DA SAÚDE, 2014).

Já para subcutâneas ou intramusculares, é importante identificar as condições clínicas do sujeito (há risco de sangramento associado a distúrbios de coagulação? O paciente está sob precaução por contato em que o uso é necessário? Vivencia-se uma situação de surto?) e também o Protocolo Operacional Padrão para procedimentos, o POP, que envolve procedimentos com injetáveis (a instituição tem em seu protocolo operacional padrão esta recomendação de uso para injeções

subcutâneas e intramusculares?), já que nestes casos, não há obrigatoriedade de uso deste EPI (CONSELHO REGIONAL DE ENFERMAGEM, 2010; MONCAIO; FIGUEIREDO, 2009; SÃO PAULO, 2015; ORGANIZAÇÃO MUNDIAL DA SAÚDE, 2010).

Resumidamente, não se deve atrelar o uso de luvas a doenças, e sim a riscos de exposição aos fluidos que podem ou não ser veículos de microrganismos, uma vez que as luvas fazem parte da precaução0padrão, e que diante de todo procedimento que envolva o risco de exposição a sangue há obrigatoriedade de uso.

E no caso de acidentes com perfurocortantes? Como prevenir a transmissão de doenças como hepatite B, C e o HIV, tornando o manuseio destes dispositivos mais seguro?

Esteja atento e participe ativamente de treinamentos que venham a ocorrer em seu ambiente de trabalho com esta temática ou busque informações e cursos que englobem a segurança para a realização destas práticas, como este que está realizando!

O treinamento é muito importante para a adesão aos dispositivos de segurança, como é o caso de seringas e agulhas com este recurso, que objetivam manter a agulha coberta pela seringa ou por capa protetora após a sua utilização. Esta adesão e as orientações e acolhimento após acidentes, como a profilaxia pós-exposição com material biológico, com eficácia quando administrada nas primeiras 72 horas que sucedem o acidente, podem minimizar agravos decorrentes da manipulação destes dispositivos (ORGANIZAÇÃO MUNDIAL DA SAÚDE, 2010).

A auto0observação antes da realização de procedimentos com injetáveis, bem como de todo o contexto em que esta medicação será administrada também precisam ser considerados. Esteja atento às suas mãos: a presença de lesões quando pequenas e superficiais, podem ser protegidas com o uso de luvas e a higienização das mãos com água e sabão deve ser priorizada em detrimento do álcool tornará a prática mais confortável e segura a prática, enquanto lesões maiores deverão ser avaliadas a fim de prevenir que haja exposição a fluidos durante a realização destes procedimentos (ORGANIZAÇÃO MUNDIAL DA SAÚDE, 2010).

Além disto, não se deve reencapar as agulhas utilizadas antes do descarte tampouco realizar a desconexão manual entre seringa e agulha, visto que muitos acidentes ocorrem em decorrência destas ações (ORGANIZAÇÃO MUNDIAL DA SAÚDE, 2010).

Ressalta-se que não há relação entre acidentes e culpa para a vítima, portanto, o manejo acertado e o apoio institucional são primordiais caso estes venham a ocorrer, pois reduzem tanto o risco de transmissão de patógenos ao profissional ou estudante quanto reduzem a ansiedade decorrente deste evento. Você sabe o que fazer, caso presencie uma lesão por perfurocortante?

Independentemente de perda sanguínea, superficial ou profunda, presença ou não de luvas durante o procedimento, se envolver uma exposição com material, utilizado para administração de injetáveis, como perfurar a pele ao descartar uma agulha utilizada para administrar uma medicação via intramuscular, o evento deve ser reportado e notificado ao serviço, e pode ser necessário que a vítima precise realizar um tratamento ou acompanhamento (o qual pode perdurar cerca de seis meses) (ORGANIZAÇÃO MUNDIAL DA SAÚDE, 2010). Não se trata de uma medida punitiva e sim estratégias para minimizar a ocorrência de agravos a sua saúde, como prevenção de infecções virais (hepatite B, C, HIV podem ser transmitidas a partir de acidentes como o exemplificado, anteriormente) (O'GRADY et al., 2011). Mantenha sua carteira de vacinação sempre atualizada, principalmente contra a hepatite B: trata-se da medida mais eficaz para a prevenção de transmissão deste vírus (ORGANIZAÇÃO MUNDIAL DA SAÚDE, 2010).

E a lesão? Como cuidar? Imediatamente, realize a limpeza com água e sabão. Não aplique álcool ou esprema a lesão, pois pode irritar a pele e aumentar a lesão, e não haverá redução da carga microbiana que adentrou ao tecido epitelial. Portanto, procure manter a calma e adicionar as demais estratégias: acompanhamento, tratamento e notificação (ORGANIZAÇÃO MUNDIAL DA SAÚDE, 2010).

Referências

Brasil. Agência Nacional de Vigilância Sanitária. **Medidas de Prevenção de Infecção Relacionada à Assistência à Saúde**. Brasília: Anvisa, 2017.

CONSELHO REGIONAL DE ENFERMAGEM DE SÃO PAULO. **Parecer nº014/2010**. Dispõe sobre o uso de luvas de procedimento para a administração de medicamentos. 2010.

GRAZIANO, Maurício Uchikawa et al. Eficácia da desinfecção com álcool 70% (p/v) de superfícies contaminadas sem limpeza prévia. **Revista Latino0Americana de Enfermagem**, Ribeirão Preto , v. 21, n. 2, p. 6180623, Abr. 2013.

MONCAIO, Ana Carolina Scarpel; FIGUEIREDO, Rosely Moralez de. Conhecimentos e práticas no uso do cateter periférico intermitente pela equipe de enfermagem. **Revista Eletrônica de Enfermagem**, v. 11, n. 3, 2009.

O'GRADY, Naomi. et al. Guidelines for the prevention of intravascular catheter0related infections. **Clinical infectious diseases**, v. 52, n. 9, p. e1620e193, 2011.

ORGANIZAÇÃO MUNDIAL DA SAÚDE. **Glove Use.** Information Leaflet. Patient Safety, 2009.

ORGANIZAÇÃO MUNDIAL DA SAÚDE. **WHO best practices for injections and related procedures toolkit**. Geneva, 2010.

SÃO PAULO. Secretaria Municipal da Saúde. **Manual de normas, rotinas e procedimentos de Enfermagem:** Atenção Básica. 2 ed. São Paulo: Secretaria Municipal da Saúde, 2014. Disponível em: <http://www.prefeitura.sp.gov.br/cidade/secretarias/upload/saude/arquivos/legislacao/NormaseRotinas02102015.pdf>. Acesso em 21 abr 2021.

VAN TUONG, Phan et al. Assessment of injection safety in Ha Dong general hospital, Hanoi, in 2012. **F1000Research**, v. 6, 2017.

APÊNDICE J – Questionário para validação do material educativo do curso "Boas Práticas com Injetáveis: ações para o controle de infecção" encaminhado às juízas

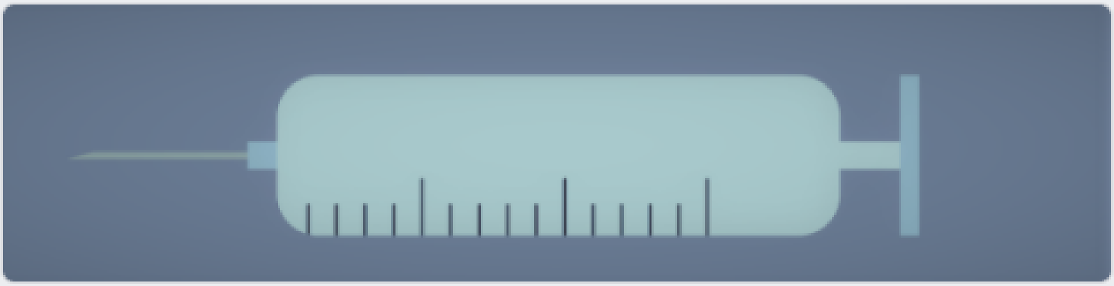

Validação de material educativo: Boas Práticas em Medicações Injetáveis

Olá!

Você está sendo convidado(a) a avaliar o material educativo referente às boas práticas com medicações injetáveis.

Este material estará disponível no Portal de Cursos Abertos - PoCA (poca.ufscar.br). Trata-se de uma plataforma disponibilizada pelo Ambiente Virtual de Aprendizagem do Moodle e que contempla cursos à distância, abertos e gratuitos, sendo administrado por equipes de pedagogia, TI, audiovisual, geral de educação à distância e comissão de avaliação de cursos da Secretaria Geral de Educação a Distância da UFSCar (SEaD-UFSCar).

O material educativo foi elaborado como etapa final de uma tese de doutorado, após devolutiva de questionário auto-aplicado por 1295 profissionais de enfermagem do estado de São Paulo distribuído eletronicamente, no qual foi identificado que, embora haja conhecimento dentro da prática segura com injetáveis, há práticas que demandam um aprofundamento no tema, semelhantemente a realidade exposta pela literatura nacional e internacional, por parte daquele que a executa a ação.
São práticas a serem refletidas: relação entre uso de luvas com as condições clínicas do paciente e risco aos fluidos independentemente de doenças de base, higienização das mãos em todas as etapas do processo com injetáveis para garantir a segurança de todos os envolvidos, proibição ao reuso de seringas e agulhas, cuidados para o profissional frente ao manejo com perfurocortantes (da prevenção de acidentes aos cuidados após lesão).

- Contextualização sobre o curso:

O curso, que contemplará este material educativo, objetiva subsidiar a prática com injetáveis em serviços de saúde, visando o preparo e a manipulação segura de injetáveis de acordo com os princípios de biossegurança, controle e prevenção de infecções relacionadas à assistência em saúde, tendo por foco a equipe de enfermagem.
O material educativo é composto por:
- Ebook (deve englobar fatores associados ao ambiente seguro e o cuidado com os insumos para preparo de injetáveis, como a manutenção de limpeza de superfície de preparo; insumos -uso único, integridade da embalagem- desinfecção e cuidados de armazenamento frascos para reconstituição de dose única/multidose, momentos para a higienização das mãos de acordo com as etapas para administração de injetáveis, uso de água e sabão ou álcool a 70%; uso de luvas (análise crítica para a proteção);
- Podcast (cuidados com perfurocortantes: adesão aos dispositivos de segurança, primeiros cuidados à lesão, vacina contra hepatite B e importância do conhecimento do Protocolo Pós-Exposição);
- 5 questões avaliativas do conteúdo (para emissão do certificado);
- 2 questões disparadoras de discussão (fórum).

Ressalta-se que se trata de um curso sem tutoria, ou seja, prioriza-se o autodidatismo do participante.

- Leia atentamente o Termo de Consentimento Livre e Esclarecido e, concordando em participar, clique em aceitar. Sua participação é muito importante nesta etapa!

- Cada seção será identificada com o componente do material que estará avaliando e contemplará as orientações de preenchimento do instrumento avaliativo.

Agradeço sua disponibilidade e atenção.

*Obrigatório

Endereço de e-mail *

Seu e-mail

Termo de Consentimento Livre e Esclarecido *

TERMO DE CONSENTIMENTO LIVRE E ESCLARECIDO
(Resolução 466/2012 do CNS)

BOAS PRÁTICAS EM MEDICAÇÕES INJETÁVEIS: INTERVENÇÃO EDUCATIVA PARA A EQUIPE DE ENFERMAGEM NO ESTADO DE SÃO PAULO

Caro(a) Senhor(a) ______________________________, o(a) Sr(a) está sendo convidado(a) para participar da pesquisa "Boas práticas em medicações injetáveis: intervenção educativa para a equipe de enfermagem no estado de São Paulo", que tem como objetivo desenvolver e validar uma estratégia educativa para profissionais de enfermagem visando às boas práticas na administração de medicações injetáveis. O(a) senhor(a) foi selecionado(a) por ter experiências clínicas na área de controle de infecção relacionada à assistência à saúde, ou tecnologia da informação e comunicação aplicada à enfermagem, possuir publicação ou pesquisar sobre o tema, ter conhecimento sobre a construção de questionários ou atuar profissionalmente como enfermeiro. Sua participação é voluntária, isto é, a qualquer momento o(a) senhor(a) pode desistir de participar e retirar seu consentimento. A sua recusa não trará nenhum prejuízo na sua relação com o pesquisador ou com a instituição que forneceu os dados. Em caso de esclarecimentos quanto à pesquisa, poderá entrar em contato com a pesquisadora pelo telefone ou e-mail abaixo deste termo, a qualquer momento.

Sua participação consiste em avaliar este material educativo quanto ao conteúdo e aparência, sugerindo modificações e realizando observações conforme julgar pertinente, enviado eletronicamente, o qual aborda questões referentes às boas praticas de medicações injetáveis a partir de um diagnóstico situacional realizado previamente no estado de São Paulo, do qual participaram profissionais de enfermagem deste estado, objetivando o aperfeiçoamento e a implantação de medidas que minimizam o risco de infecção relacionada à assistência à saúde e responder a um breve questionário pessoal no qual deve informar seu nível de instrução, tempo de atuação na área e de serviço em que atua. O tempo utilizado para coleta dos dados será de aproximadamente 45 minutos.

Suas respostas serão tratadas de forma anônima e confidencial, desta forma, não será divulgado seu nome em qualquer fase do estudo. Quando for necessário exemplificar determinada situação, sua privacidade será assegurada. Os dados coletados poderão ter seus resultados divulgados em eventos, revistas e/ou trabalhos científicos.

Os riscos presentes em sua participação são mínimos, e frente a qualquer desconforto ou cansaço o questionário poderá deixar de ser respondido, sem nenhum prejuízo ou perda para você, e posteriormente retomado, caso seja de seu interesse.

Você não terá nenhum custo ou compensação financeira ao participar do estudo. Contudo, destaca-se que sua participação auxiliará a elaboração de ferramenta educativa que poderá ser utilizado na decisão crítica-reflexiva de estratégias visando boas práticas de medicações injetáveis, material divulgado eletronicamente, ao qual o(a) senhor(a) terá acesso assim que publicado.

O(a) senhor(a) receberá uma via assinada deste termo, por você e pelo pesquisador, onde consta o telefone e o endereço do pesquisador principal.

Declaro que entendi os objetivos, riscos e benefícios de minha participação na pesquisa e concordo em participar. O pesquisador me informou que o projeto foi aprovado pelo Comitê de Ética em Pesquisa em Seres Humanos da UFSCar que funciona na Pró-Reitoria de Pesquisa da Universidade Federal de São Carlos, localizada na Rodovia Washington Luiz, Km. 235 - Caixa Postal 676 - CEP 13.565-905 - São Carlos - SP – Brasil. Fone (16) 3351-8028. Endereço eletrônico: cephumanos@ufscar.br

Endereço para contato

Pesquisadora Responsável: Camila Eugenia Roseira
Endereço: UFSCar, Rod. Washington Luis, Km 235, São Carlos/SP
Contato telefônico: (16) 3351-8338. e-mail: roseira.camila@gmail.com

◯ Li o Termo de Consentimento Livre e Esclarecido e aceito participar.

◯ Não aceito

Instrumentos de validação utilizados

Para a validação deste ebook e do podcast será utilizado o INSTRUMENTO DE VALIDAÇÃO DE CONTEÚDO EDUCATIVO EM SAÚDE (IVCES), de autoria de Leite et al. (2018)*: este instrumento generalista se destina à validação de conteúdo educativo em saúde. Constitui-se de dados de identificação do respondente; instruções para preenchimento e itens de avaliação do conteúdo.

Para validação das questões avaliativas do conteúdo do curso e das duas questões do fórum, utilizou-se o instrumento AVALIAÇÃO DA VALIDADE DE CONTEÚDO proposto por Alexandre e Collucci (2015)**.

*LEITE, Sarah de Sá et al . Construção e validação de Instrumento de Validação de Conteúdo Educativo em Saúde. Rev. Bras. Enferm., Brasília , v. 71, supl. 4, p. 1635-1641, 2018 . Disponível em <http://www.scielo.br/scielo.php?script=sci_arttext&pid=S0034-71672018001001635&lng=en&nrm=iso>

**COLUCI, MZO; ALEXANDRE, NMC; MILANI, D. Construção de instrumentos de medida na área da saúde. Ciênc. saúde coletiva, Rio de Janeiro , v. 20, n. 3, p. 925-936, Mar. 2015 . Disponível em <http://www.scielo.br/scielo.php?script=sci_arttext&pid=S1413-81232015000300925&lng=en&nrm=iso>

Nome *

Sua resposta

Idade *

Sua resposta

Maior titulação *

Sua resposta

Ocupação atual *

Sua resposta

Próxima

Página 1 de 4

Nunca envie senhas pelo Formulários Google.

Este conteúdo não foi criado nem aprovado pelo Google. Denunciar abuso - Termos de Serviço - Política de Privacidade

Google Formulários

VALIDAÇÃO DO E-BOOK(pdf) - BOAS PRÁTICAS COM INJETÁVEIS: Ações para o controle de infecção

Antes de responder ao questionário a seguir, sugere-se a leitura do Ebook "BOAS PRÁTICAS COM INJETÁVEIS: Ações para o controle de infecção" em formato PDF encaminhado juntamente com o link de acesso a este instrumento, por e-mail.

O instrumento (IVCES) possui 18 itens com divisões em 3 domínios. Domínio "objetivo" compõe-se dos propósitos, metas ou finalidades que se desejam com a utilização. "Estrutura e apresentação" referem-se à organização geral, estrutura, estratégia, coerência e suficiência das apresentações. Por fim, "relevância" é a característica que avalia o grau de significação do conteúdo educativo apresentado, capacidade do conteúdo em causar impacto, motivação e/ou interesse.

Leia atentamente cada item e pontue conforme seu critério, de acordo com a seguinte valoração:

2 – Adequado
1 – Parcialmente adequado
0 – Inadequado

Após a avaliação dos itens, de cada domínio, há espaço para sugestões e críticas. Caso atribua as notas 0 e 1 escreva nesse local sua justificativa e colaboração para melhoria do material.

- Referências utilizadas para elaboração do Ebook:

BRASIL. Medidas de Prevenção de Infecção Relacionada à Assistência à Saúde. [S.l: s.n.], 2017. Disponível em: <http://portal.anvisa.gov.br/documents/33852/271855/Medidas+de+Prevenção+de+Infecção+Relacionada+à+Assistência+à+Saúde/6b16dab3-6d0c-4399-9d84-141d2e81c809>.

FERRONI, A et al. Pulsative flushing as a strategy to prevent bacterial colonization of vascular access devices. Medical Devices: Evidence and Research, vol. 7, pp. 379– 383, 2014.

GRAZIANO, M. et al. Eficácia da desinfecção com álcool 70% (p/v) de superfícies contaminadas sem limpeza prévia. Rev. Latino-Am. Enfermagem, v. 2, p. 06 telas, 2012.

RIGOTTI, M. A. Segurança microbiológica na abertura de ampolas com ênfase no procedimento de desinfecção. . [S.l: s.n.], 2013. Disponível em: <https://www.teses.usp.br/teses/disponiveis/22/22132/tde-05112012-201527/pt-br.php>.

VAN TUONG, P. et al. Assessment of injection safety in Ha Dong General Hospital, Hanoi, in 2012. F1000Research, 2017.

WHO. WHO Best practices for injections and related procedures toolkit. Geneva, Suíça: [s.n.], 2010.

OBJETIVOS: propósitos, metas ou finalidades

1. Contempla tema proposto *

- ○ 0 – Inadequado
- ○ 1 – Parcialmente adequado
- ○ 2 – Adequado

2. Adequado ao processo de ensino-aprendizagem *

- ○ 0 – Inadequado
- ○ 1 – Parcialmente adequado
- ○ 2 – Adequado

3. Esclarece dúvidas sobre o tema abordado *

- 0 – Inadequado
- 1 – Parcialmente adequado
- 2 – Adequado

4. Proporciona reflexão sobre o tema *

- 0 – Inadequado
- 1 – Parcialmente adequado
- 2 – Adequado

5. Incentiva mudança de comportamento *

- 0 – Inadequado
- 1 – Parcialmente adequado
- 2 – Adequado

Sugestões (justificativa para notas 0 ou 1 e colaboração para melhoria do material):

Sua resposta

ESTRUTURA/APRESENTAÇÃO: organização, estrutura, estratégia, coerência e suficiência

6. Linguagem adequada ao público-alvo *

- 0 – Inadequado
- 1 – Parcialmente adequado
- 2 – Adequado

7. Linguagem apropriada ao material educativo *

- 0 – Inadequado
- 1 – Parcialmente adequado
- 2 – Adequado

8. Linguagem interativa, permitindo envolvimento ativo no processo educativo *

- 0 – Inadequado
- 1 – Parcialmente adequado
- 2 – Adequado

9. Informações corretas *

- 0 – Inadequado
- 1 – Parcialmente adequado
- 2 – Adequado

10. Informações objetivas *

- 0 – Inadequado
- 1 – Parcialmente adequado
- 2 – Adequado

11. Informações esclarecedoras *

- 0 – Inadequado
- 1 – Parcialmente adequado
- 2 – Adequado

12. Informações necessárias *

- 0 – Inadequado
- 1 – Parcialmente adequado
- 2 – Adequado

13. Sequência lógica das ideias *

- 0 – Inadequado
- 1 – Parcialmente adequado
- 2 – Adequado

14. Tema atual *

- 0 – Inadequado
- 1 – Parcialmente adequado
- 2 – Adequado

15. Tamanho do texto adequado *

- 0 – Inadequado
- 1 – Parcialmente adequado
- 2 – Adequado

Sugestões (justificativa para notas 0 ou 1 e colaboração para melhoria do material):

Sua resposta

RELEVÂNCIA: significância, impacto, motivação e interesse

16. Estimula o aprendizado *

- 0 – Inadequado
- 1 – Parcialmente adequado
- 2 – Adequado

17. Contribui para o conhecimento na área *

- 0 – Inadequado
- 1 – Parcialmente adequado
- 2 – Adequado

18. Desperta interesse pelo tema *

- 0 – Inadequado
- 1 – Parcialmente adequado
- 2 – Adequado

Sugestões (justificativa para notas 0 ou 1 e colaboração para melhoria do material):

Sua resposta

Voltar Próxima

Página 2 de 4

Nunca envie senhas pelo Formulários Google.

Este conteúdo não foi criado nem aprovado pelo Google. Denunciar abuso - Termos de Serviço - Política de Privacidade

Google Formulários

VALIDAÇÃO DO PODCAST - BOAS PRÁTICAS COM MEDICAÇÕES INJETÁVEIS: subsídios para o autocuidado

Antes de responder ao questionário a seguir, sugere-se que ouça ao podcast, em formato MP3, e leia a transcrição do áudio, encaminhados juntamente com o link de acesso a este instrumento, por e-mail.

O instrumento (IVCES) possui 18 itens com divisões em 3 domínios. Domínio "objetivo" compõe-se dos propósitos, metas ou finalidades que se desejam com a utilização. "Estrutura e apresentação" referem-se à organização geral, estrutura, estratégia, coerência e suficiência das apresentações. Por fim, "relevância" é a característica que avalia o grau de significação do conteúdo educativo apresentado, capacidade do conteúdo em causar impacto, motivação e/ou interesse.

Leia atentamente cada item e pontue conforme seu critério, de acordo com a seguinte valoração:

2 – Adequado
1 – Parcialmente adequado
0 – Inadequado

Após a avaliação dos itens, de cada domínio, há espaço para sugestões e críticas. Caso atribua as notas 0 e 1 escreva nesse local sua justificativa e colaboração para melhoria do material.

- Referências utilizadas para elaboração do Podcast:

BRASIL. Medidas de Prevenção de Infecção Relacionada à Assistência à Saúde. 2017. Disponível em: <http://portal.anvisa.gov.br/documents/33852/271855/Medidas+de+Prevenção+de+Infecção+Relacionada+à+Assistência+à+Saúde/6b16dab3-6d0c-4399-9d84-141d2e81c809>.

COREN-SP. Parecer Coren-Sp no 019/2010 Dispõe sobre o uso de luvas de procedimento para administração de medicamentos. Disponível em: <http://portal.coren-sp.gov.br/sites/default/files/parecer_coren_sp_2010_18_0.pdf>. , 2010

GRAZIANO, M. et al. Eficácia da desinfecção com álcool 70% (p/v) de superfícies contaminadas sem limpeza prévia. Rev. Latino-Am. Enfermagem, v. 2, p. 06 telas, 2012.

MONCAIO, A. C. S.; FIGUEIREDO, R. M. DE. Conhecimentos e práticas no uso do cateter periférico intermitente pela equipe de enfermagem. Revista Eletrônica de Enfermagem, 2017.

O'GRADY, N. P. et al. Guidelines for the prevention of intravascular catheter-related infections. Clinical infectious diseases: an official publication of the Infectious Diseases Society of America, 2011.
SÃO PAULO (CIDADE). SECRETARIA DA SAÚDE. Manual de normas, rotinas e procedimentos de Enfermagem- Atenção Básica. Secretaria Municipal de São Paulo. [S.l: s.n.]. Disponível em: <http://www.prefeitura.sp.gov.br/cidade/secretarias/upload/saude/arquivos/legislacao/NormaseRotinas02102015.pdf>. 2015

WHO. Glove Use Information Leaflet. Patient Safety, 2014.

WHO. WHO Best practices for injections and related procedures toolkit. Geneva, Suíça: [s.n.], 2010.

Música:
Funny by MusicLFiles
Link: https://filmmusic.io/song/6388-funny
License: http://creativecommons.org/licenses/by/4.0/

OBJETIVOS: propósitos, metas ou finalidades

1. Contempla tema proposto *

- 0- Inadequado
- 1 – Parcialmente adequado
- 2 – Adequado

2. Adequado ao processo de ensino-aprendizagem *

- 0 - Inadequado
- 1 – Parcialmente adequado
- 2 – Adequado

3. Esclarece dúvidas sobre o tema abordado *

- 0- Inadequado
- 1 – Parcialmente adequado
- 2 – Adequado

4. Proporciona reflexão sobre o tema *

- 0- Inadequado
- 1 – Parcialmente adequado
- 2 – Adequado

5. Incentiva mudança de comportamento *

- 0- Inadequado
- 1 – Parcialmente adequado
- 2 – Adequado

Sugestões (justificativa para notas 0 ou 1 e colaboração para melhoria do material):

Sua resposta

ESTRUTURA/APRESENTAÇÃO: organização, estrutura, estratégia, coerência e suficiência

6. Linguagem adequada ao público-alvo *

- 0- Inadequado
- 1 – Parcialmente adequado
- 2 – Adequado

7. Linguagem apropriada ao material educativo *

- 0- Inadequado
- 1 – Parcialmente adequado
- 2 – Adequado

8. Linguagem interativa, permitindo envolvimento ativo no processo educativo *

- 0- Inadequado
- 1 – Parcialmente adequado
- 2 – Adequado

9. Informações corretas *

- 0- Inadequado
- 1 – Parcialmente adequado
- 2 – Adequado

10. Informações objetivas *

- 0- Inadequado
- 1 – Parcialmente adequado
- 2 – Adequado

11. Informações esclarecedoras *

- 0- Inadequado
- 1 – Parcialmente adequado
- 2 – Adequado

12. Informações necessárias *

- 0- Inadequado
- 1 – Parcialmente adequado
- 2 – Adequado

13. Sequência lógica das ideias *

- 0- Inadequado
- 1 – Parcialmente adequado
- 2 – Adequado

14. Tema atual *

- ◯ 0- Inadequado
- ◯ 1 – Parcialmente adequado
- ◯ 2 – Adequado

15. Tamanho do texto adequado *

- ◯ 0- Inadequado
- ◯ 1 – Parcialmente adequado
- ◯ 2 – Adequado

Sugestões (justificativa para notas 0 ou 1 e colaboração para melhoria do material):

Sua resposta

RELEVÂNCIA: significância, impacto, motivação e interesse

16. Estimula o aprendizado *

- 0- Inadequado
- 1 – Parcialmente adequado
- 2 – Adequado

17. Contribui para o conhecimento na área *

- 0- Inadequado
- 1 – Parcialmente adequado
- 2 – Adequado

18. Desperta interesse pelo tema *

- 0- Inadequado
- 1 – Parcialmente adequado
- 2 – Adequado

Sugestões (justificativa para notas 0 ou 1 e colaboração para melhoria do material):

Sua resposta

VALIDAÇÃO DAS QUESTÕES AVALIATIVAS DO CONTEÚDO DO CURSO

Estas questões objetivas foram consolidadas para a emissão de certificado, e, elaboradas conforme as seguintes referências:

BRASIL. Medidas de Prevenção de Infecção Relacionada à Assistência à Saúde. [S.l: s.n.], 2017. Disponível em: <http://portal.anvisa.gov.br/documents/33852/271855/Medidas+de+Prevenção+de+Infecção+Relacionada+à+Assistência+à+Saúde/6b16dab3-6d0c-4399-9d84-141d2e81c809>.

COREN-SP. Parecer Coren-Sp no 019/2010 Dispõe sobre o uso de luvas de procedimento para administração de medicamentos. Disponível em: <http://portal.coren-sp.gov.br/sites/default/files/parecer_coren_sp_2010_18_0.pdf>. , 2010

MONCAIO, A. C. S.; FIGUEIREDO, R. M. DE. Conhecimentos e práticas no uso do cateter periférico intermitente pela equipe de enfermagem. Revista Eletrônica de Enfermagem, 2017.

FERRONI, A et al. Pulsative flushing as a strategy to prevent bacterial colonization of vascular access devices. Medical Devices: Evidence and Research, vol. 7, pp. 379– 383, 2014.

O'GRADY, N. P. et al. Guidelines for the prevention of intravascular catheter-related infections. Clinical infectious diseases : an official publication of the Infectious Diseases Society of America, 2011.
SÃO PAULO (CIDADE). SECRETARIA DA SAÚDE. Manual de normas, rotinas e procedimentos de Enfermagem- Atenção Básica. Secretaria Municipal de São Paulo. [S.l: s.n.]. Disponível em: <http://www.prefeitura.sp.gov.br/cidade/secretarias/upload/saude/arquivos/legislacao/NormaseRotinas02102015.pdf>. 2015

WHO. Glove Use Information Leaflet. Patient Safety, 2014.

WHO. WHO Best practices for injections and related procedures toolkit. . Geneva, Suiça: [s.n.], 2010.

Orientações para avaliação do questionário:

Avalie cada questão segundo clareza e pertinência, conforme escala de 1 a 4 abaixo:

- Clareza: avaliar se os itens são redigidos de modo que o conceito esteja compreensível e se expresse adequadamente ao que se espera medir.
1:Não claro
2: Item necessita de grande revisão para ser claro
3: Item necessita de pequena revisão para ser claro
4:Item claro

-Pertinência: avaliar se os itens refletem os conceitos envolvidos, se são relevantes e adequados para atingir objetivos almejados.
1: Não pertinente
2: Item necessita de grande revisão para ser relevante
3: Item necessita de pequena revisão para ser relevante
4: Item pertinente

Modelo estrutural das questões obrigatórias para conclusão do curso: elementos pertencentes às questões objetivas

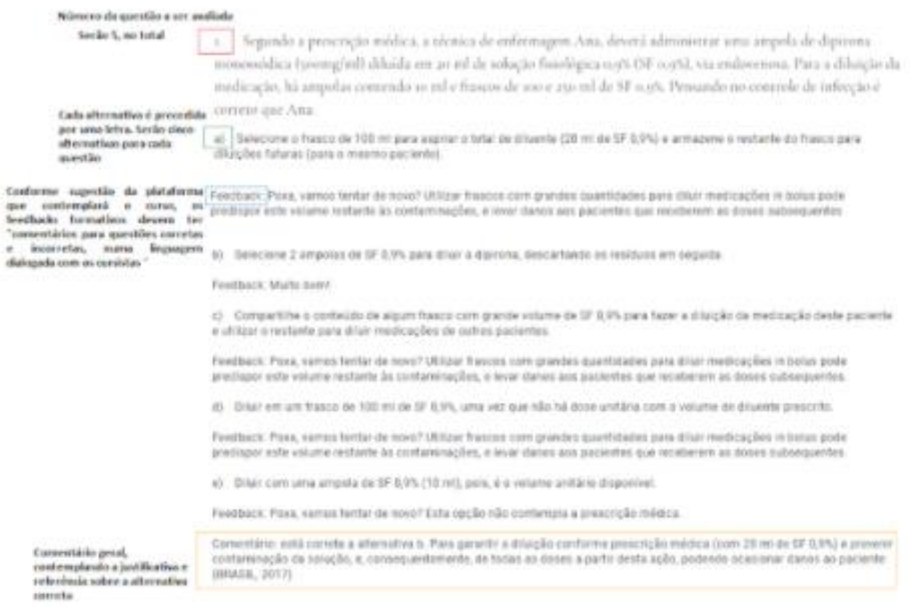
Número da questão a ser avaliada

Seção 5, no total

[illegible] Segundo a prescrição médica, a técnica de enfermagem Ana, deverá administrar uma ampola de dipirona monossódica [illegible] diluída em 20 ml de solução fisiológica 0,9% (SF 0,9%), via endovenosa. Para a diluição da medicação, há ampolas contendo 10 ml e frascos de 100 e 250 ml de SF 0,9%. Pensando no controle de infecção é correto que Ana:

Cada alternativa é precedida por uma letra. Serão cinco alternativas para cada questão

a) Selecione o frasco de 100 ml para aspirar o total de diluente (20 ml de SF 0,9%) e armazene o restante do frasco para diluições futuras (para o mesmo paciente).

Conforme sugestão da plataforma que contemplará o curso, os feedbacks formativos devem ter "comentários para questões corretas e incorretas, numa linguagem dialogada com os cursistas."

Feedback: Poxa, vamos tentar de novo? Utilizar frascos com grandes quantidades para diluir medicações in bolus pode predispor este volume restante às contaminações, e levar danos aos pacientes que receberem as doses subsequentes.

b) Selecione 2 ampolas de SF 0,9% para diluir a dipirona, descartando os resíduos em seguida.

Feedback: Muito bem!

c) Compartilhe o conteúdo de algum frasco com grande volume de SF 0,9% para fazer a diluição da medicação deste paciente e utilizar o restante para diluir medicações de outros pacientes.

Feedback: Poxa, vamos tentar de novo? Utilizar frascos com grandes quantidades para diluir medicações in bolus pode predispor este volume restante às contaminações, e levar danos aos pacientes que receberem as doses subsequentes.

d) Diluir em um frasco de 100 ml de SF 0,9%, uma vez que não há dose unitária com o volume de diluente prescrito.

Feedback: Poxa, vamos tentar de novo? Utilizar frascos com grandes quantidades para diluir medicações in bolus pode predispor este volume restante às contaminações, e levar danos aos pacientes que receberem as doses subsequentes.

e) Diluir com uma ampola de SF 0,9% (10 ml), pois, é o volume unitário disponível.

Feedback: Poxa, vamos tentar de novo? Esta opção não contempla a prescrição médica.

Comentário geral, contemplando a justificativa e referência sobre a alternativa correta

Comentário: está correta a alternativa b. Para garantir a diluição conforme prescrição médica (com 20 ml de SF 0,9%) e prevenir contaminação da solução, e, consequentemente, de todas as doses a partir desta ação, podendo ocasionar danos ao paciente (BRASIL, 2017).

1. Segundo a prescrição médica, a técnica de enfermagem Ana, deverá administrar uma ampola de dipirona monossódica (500mg/ml) diluída em 20 ml de solução fisiológica 0,9% (SF 0,9%), via endovenosa. Para a diluição da medicação, há ampolas contendo 10 ml e frascos de 100 e 250 ml de SF 0,9%. Pensando no controle de infecção é correto que Ana:

a) Selecione o frasco de 100 ml para aspirar o total de diluente (20 ml de SF 0,9%) e armazene o restante do frasco para diluições futuras (para o mesmo paciente).

Feedback: Poxa, vamos tentar de novo? Utilizar frascos com grandes quantidades para diluir medicações in bolus pode predispor este volume restante às contaminações, e levar danos aos pacientes que receberem as doses subsequentes

b) Selecione 2 ampolas de SF 0,9% para diluir a dipirona, descartando os resíduos em seguida.

Feedback: Muito bem!

c) Compartilhe o conteúdo de algum frasco com grande volume de SF 0,9% para fazer a diluição da medicação deste paciente e utilizar o restante para diluir medicações de outros pacientes.

Feedback: Poxa, vamos tentar de novo? Utilizar frascos com grandes quantidades para diluir medicações in bolus pode predispor este volume restante às contaminações, e levar danos aos pacientes que receberem as doses subsequentes.

d) Diluir em um frasco de 100 ml de SF 0,9%, uma vez que não há dose unitária com o volume de diluente prescrito.

Feedback: Poxa, vamos tentar de novo? Utilizar frascos com grandes quantidades para diluir medicações in bolus pode predispor este volume restante às contaminações, e levar danos aos pacientes que receberem as doses subsequentes

e) Diluir com uma ampola de SF 0,9% (10 ml), pois, é o volume unitário disponível.

Feedback: Poxa, vamos tentar de novo? Esta opção não contempla a prescrição médica.

Comentário: está correta a alternativa b. Para garantir a diluição conforme prescrição médica (com 20 ml de SF 0,9%) e prevenir contaminação da solução, e, consequentemente, de todas as doses a partir desta ação, podendo ocasionar danos ao paciente (BRASIL, 2017)

Clareza: foi redigida de forma clara, compreensível e sem ambiguidade? *

- ◯ 1 - Não claro
- ◯ 2 - Item necessita de grande revisão para ser claro
- ◯ 3 - Item necessita de pequena revisão para ser claro
- ◯ 4 - Item claro

Pertinência: reflete os conceitos envolvidos, são relevantes e adequados à temática do estudo? *

- ◯ 1 - Não pertinente
- ◯ 2 - Item necessita de grande revisão para ser relevante
- ◯ 3 - Item necessita de pequena revisão para ser relevante
- ◯ 4 - -Item pertinente

Observação

Sua resposta

Sugestão de nova redação

Sua resposta

2. A higienização das mãos é primordial para a redução de infecção em serviços de saúde, pois, remove os microrganismos a partir do emprego de água e sabão ou solução alcoólica. É correto considerar que, ao realizar uma medicação endovenosa:I. Deve-se higienizar as mãos antes da inserção do cateter venoso a fim de prevenir algum dano infeccioso no paciente.II. Como, obrigatoriamente, deve-se utilizar luvas de procedimento para realizar o procedimento, é facultativa a higienização das mãos após a retirada deste equipamento de proteção individual.III. Deve-se higienizar as mãos após a manipulação de cateteres venosos periféricos pois protege ao profissional ou estudante que realizou esta ação. É correto o que se afirma:

a) I, II e III

Feedback: Poxa, vamos tentar de novo? O uso de luvas não substitui a higienização das mãos: são complementares, pois, não impede que o profissional ou estudante entre em contato com microrganismos, uma vez que microfuros podem estar presentes nas luvas.

b) I e II

Feedback: Poxa, vamos tentar de novo? O uso de luvas não substitui a higienização das mãos: são complementares, pois, não impede que o profissional ou estudante entre em contato com microrganismos, uma vez que microfuros podem estar presentes nas luvas.

c) I e III

Feedback: Muito bem!

d) II e III

Feedback: Poxa, vamos tentar de novo? O uso de luvas não substitui a higienização das mãos: são complementares, pois, não impede que o profissional ou estudante entre em contato com microrganismos, uma vez que microfuros podem estar presentes nas luvas.

e) Apenas II

Feedback: Poxa, vamos tentar de novo? O uso de luvas não substitui a higienização das mãos: são complementares, pois, não impede que o profissional ou estudante entre em contato com microrganismos, uma vez que microfuros podem estar presentes nas luvas.

Comentário: está correta a alternativa c. A higienização das mãos, quando precedente aos procedimentos intravenosos, visa a segurança do paciente, ou seja, previne agravos a este. Quando após procedimento, é para a segurança daquele que executa a técnica. Além disso, o uso de luvas protege o sujeito do risco de exposição ao sangue do paciente, enquanto a higienização das mãos é um mecanismo de redução de exposição e prevenção de patógenos, portanto, são complementares (BRASIL, 2017; WHO, 2010)

Clareza: foi redigida de forma clara, compreensível e sem ambiguidade? *

- ◯ 1 - Não claro
- ◯ 2 - Item necessita de grande revisão para ser claro
- ◯ 3 - Item necessita de pequena revisão para ser claro
- ◯ 4 - Item claro

Pertinência: reflete os conceitos envolvidos, são relevantes e adequados à temática do estudo? *

- ◯ 1 - Não pertinente
- ◯ 2 - Item necessita de grande revisão para ser relevante
- ◯ 3 - Item necessita de pequena revisão para ser relevante
- ◯ 4 - Item pertinente

Sugestão de nova redação

Sua resposta

Observação

Sua resposta

3. Diz-se __________________ a administração de solução fisiológica com seringa de 10 ml para gerar uma __________ pressão no lúmen do cateter intravenoso, a fim de manter sua integridade e ____________, prevenir obstrução e __________ de microrganismos. Deve-se realizar o procedimento antes e após cada administração endovenosa. É importante utilizar seringas ______________ pois o reuso é ______________ mesmo se tratando de procedimento no mesmo paciente.

a) Lock, alta, funcionalidade, nidação, compartilhadas, permitido

Feedback: Poxa, vamos tentar de novo? O conceito de lock não se aplica, neste caso... A alta pressão, quando aplicada em cateteres venosos periféricos, pode danificar o dispositivo e deslocar trombos, liberando-o para a corrente sanguínea. Não se deve reutilizar seringas ou agulhas, pois, estão são descartáveis, portanto, de uso único a cada procedimento, independentemente do contexto.

b) Flushing, alta, funcionalidade, esterilidade, estéreis, proibido

Feedback: Poxa, vamos tentar de novo? A alta pressão, quando aplicada em cateteres venosos periféricos, pode danificar o dispositivo e deslocar trombos, liberando-o para a corrente sanguínea. O conceito de esterilidade não se aplica à presença de microrganismos.

c) Flushing, baixa, funcionalidade, nidação, estéreis, proibido

Feedback: Muito bem!

d) Lock, baixa, funcionalidade, nidação, novas, proibido

Feedback: Poxa, vamos tentar de novo? O conceito de lock não se aplica, neste caso...

e) Flushing, baixa, funcionalidade, esterilidade, compartilhadas, permitido

Feedback: Poxa, vamos tentar de novo? A alta pressão, quando aplicada em cateteres venosos periféricos, pode danificar o dispositivo e deslocar trombos, liberando-o para a corrente sanguínea. O conceito de esterilidade não se aplica à presença de microrganismos. Além disso, não se deve reutilizar seringas ou agulhas, pois, estão são descartáveis, portanto, de uso único a cada procedimento, independentemente do contexto.

Comentário: está correta a alternativa c. O flushing é um procedimento que deve suceder toda medicação injetável. Deve ser realizado com seringa de 10 ml, assim, exerce uma baixa pressão no lúmen do cateter, prevenido danificações no cateter, sem prejuízo na verificação de resistência. O flushing visa remover resíduos do cateter, prevenindo a nidação (ou fixação) de microrganismos. Para garantir a esterilidade e segurança deste procedimento, deve-se utilizar insumos estéreis e descartá-los em seguida, pois, seringas e agulhas são insumos descartáveis, de uso único e não podem ser reutilizadas, mesmo se tratando do mesmo paciente (BRASIL, 2017; FERRONI, 2014).

Clareza: foi redigida de forma clara, compreensível e sem ambiguidade? *

- 1 - Não claro
- 2 - Item necessita de grande revisão para ser claro
- 3 - Item necessita de pequena revisão para ser claro
- 4 - Item claro

-Pertinência: reflete os conceitos envolvidos, são relevantes e adequados à temática do estudo? *

- 1 - Não pertinente
- 2 - Item necessita de grande revisão para ser relevante
- 3 - Item necessita de pequena revisão para ser relevante
- 4 - Item pertinente

Sugestão de nova redação

Sua resposta

Observação

Sua resposta

4. Ao realizar uma medicação intramuscular em uma Unidade de Pronto Atendimento (UPA), Fernando acaba perfurando sua mão, ao reencapar a agulha já utilizada. Como ele é recém-contratado, está receoso de sofrer alguma sanção ao reportar o ocorrido ao serviço, já que, na ocasião, não estava utilizando luvas de procedimento.

Analise as colocações a seguir:

I) É obrigatório o uso de luvas para qualquer injeção intramuscular.

II) Caso ele esteja com o esquema vacinal contra hepatite B, não há risco algum neste evento acidental.

III) O evento deve ser reportado e notificado ao serviço, pois, há riscos à saúde do profissional

IV) O evento deve ser reportado e notificado ao serviço, pois, há riscos à saúde do profissional e do paciente que recebeu a injeção

É correto o que se afirma em:

a) I e III

Feedback: Poxa, vamos tentar de novo? Não é obrigatório o uso de luvas em injeções intramusculares, necessitando de uma avaliação prévia do profissional antes da realização destes procedimentos, que deve observar tanto as condições do paciente, do ambiente e de suas mãos, quanto compreender a posição do serviço sobre estes procedimentos através dos protocolos operacionais. Além disso, conforme acidente aqui narrado, o evento expôs o profissional ao risco, e não o paciente.

b) Apenas IV

Feedback: Poxa, vamos tentar de novo? No exemplificado, o evento expôs o profissional ao risco, e não o paciente.

c) I e II

Feedback: Poxa, vamos tentar de novo? Não é obrigatório o uso de luvas em injeções intramusculares, necessitando de uma avaliação prévia do profissional antes da realização destes procedimentos, que deve observar tanto as condições do paciente, do ambiente e de suas mãos, quanto compreender a posição do serviço sobre estes procedimentos através dos protocolos operacionais. Além disso, há outras doenças passíveis de transmissão pela corrente sanguínea, além da hepatite B, não é mesmo?

d) Apenas III

Feedback: Muito bem!

e) II e IV

Feedback: Poxa, vamos tentar de novo? há outras doenças passíveis de transmissão pela corrente sanguínea, além da hepatite B, não é mesmo? Além disso, conforme acidente aqui narrado, o evento expôs o profissional ao risco, e não o paciente.

Comentário: está correta a alternativa d. Não é obrigatório o uso de luvas em injeções intramusculares, necessitando de uma avaliação prévia do profissional antes da realização destes procedimentos, que deve observar tanto as condições do paciente, do ambiente e de suas mãos, quanto compreender a posição do serviço sobre estes procedimentos através dos protocolos operacionais. É interessante reportar e notificar o serviço pois, mesmo com a situação vacinal atualizada contra hepatite B, pode estar exposto a outras doenças transmissíveis pela corrente sanguíne (COREN-SP, 2010; MONCAIO; FIGUEIREDO, 2017; SÃO PAULO (CIDADE). SECRETARIA DA SAÚDE, 2015; WHO, 2010).

Clareza: foi redigida de forma clara, compreensível e sem ambiguidade? *

- ◯ 1 - Não claro
- ◯ 2 - Item necessita de grande revisão para ser claro
- ◯ 3 - Item necessita de pequena revisão para ser claro
- ◯ 4 - Item claro

Pertinência: reflete os conceitos envolvidos, são relevantes e adequados à temática do estudo? *

- ◯ 1 - Não pertinente
- ◯ 2 - Item necessita de grande revisão para ser relevante
- ◯ 3 - Item necessita de pequena revisão para ser relevante
- ◯ 4 - Item pertinente

Sugestão de nova redação

Sua resposta

Observação

Sua resposta

5. O álcool a 70% é uma solução muito importante nos serviços de saúde. Além de seu reconhecimento enquanto desinfetante, podendo ser utilizado na limpeza de bandejas e superfícies, pode ser empregado para a higienização das mãos, otimizando o tempo para esta ação, e também aplicado às lesões decorrentes de acidentes com perfurocortantes, já que reduz a quantidade de microrganismos que podem ter adentrado à pele do profissional, sendo assim, a solução de escolha para o primeiro cuidado com lesões deste tipo.

a) Esta afirmação é parcialmente verdadeira, pois, não se deve aplicar solução alcoólica às lesões por perfurocortante, visto potencial irritante.

Feedback: Muito bem!

b) Esta afirmação é totalmente verdadeira.

Feedback: Poxa... Vamos tentar de novo? O cuidado à lesão, em um primeiro momento, deve ser realizado apenas com água e sabão, assim, não há risco de irritar a pele, ou mesmo, aumentar a área lesionada.

c) Esta afirmação é parcialmente verdadeira, visto que não se deve empregar solução alcoólica para a limpeza de bandejas, já que não abrange microrganismos que possam estar nestes recursos.

Feedback: Poxa... Vamos tentar de novo? A versatilidade da solução alcoólica enquanto desinfetante e solução antisséptica de para higienização das mãos é cientificamente comprovada.

d) Esta afirmação é totalmente falsa.

Feedback: A versatilidade da solução alcoólica enquanto desinfetante e solução antisséptica de para higienização das mãos é cientificamente comprovada, dentre as vantagens, encontra-se o menor tempo dispendido para esta ação, em comparação a higiene das mãos, contudo, em lesões por perfurocortante, o cuidado à lesão, em um primeiro momento, deve ser realizado apenas com água e sabão, assim, não há risco de irritar a pele, ou mesmo, aumentar a área lesionada.

e) Esta afirmação é parcialmente verdadeira, visto que o tempo de secagem do álcool a 70%, comparativamente à higienização das mãos com álcool e sabão, não apresenta vantagem.

Feedback: A versatilidade da solução alcoólica enquanto desinfetante e solução antisséptica de para higienização das mãos é cientificamente comprovada, dentre as vantagens, encontra-se o menor tempo dispendido para esta ação

Comentário: está correta a alternativa a. A versatilidade da solução alcoólica enquanto desinfetante e solução antisséptica de para higienização das mãos é cientificamente comprovada, dentre as vantagens, encontra-se o menor tempo dispendido para esta ação, em comparação a higiene das mãos com água e sabão, contudo, em lesões por perfurocortante, o cuidado à lesão, em um primeiro momento, deve ser realizado apenas com água e sabão, assim, não há risco de irritar a pele, ou mesmo, aumentar a área lesionada (WHO, 2010).

Clareza: foi redigida de forma clara, compreensível e sem ambiguidade? *

- 1 - Não claro
- 2 - Item necessita de grande revisão para ser claro
- 3 - Item necessita de pequena revisão para ser claro
- 4 - Item claro

Pertinência: reflete os conceitos envolvidos, são relevantes e adequados à temática do estudo? *

- 1 - Não pertinente
- 2 - Item necessita de grande revisão para ser relevante
- 3 - Item necessita de pequena revisão para ser relevante
- 4 - Item pertinente

Sugestão de nova redação

Sua resposta

Observação

Sua resposta

Voltar   Próxima   Página 3 de 4

Nunca envie senhas pelo Formulários Google.

Este conteúdo não foi criado nem aprovado pelo Google. Denunciar abuso - Termos de Serviço - Política de Privacidade

Google Formulários

VALIDAÇÃO DAS QUESTÕES DISPARADORAS DE DISCUSSÃO DO CONTEÚDO DO CURSO

As duas questões disparadoras de discussão (abertas) serão para o fórum, não têm caráter avaliativo ou tutoria para moderação.

Orientações para avaliação do questionário:

Avalie cada questão segundo clareza e pertinência, conforme escala de 1 a 4 abaixo:

- Clareza: avaliar se os itens são redigidos de modo que o conceito esteja compreensível e se expresse adequadamente ao que se espera medir.
1:Não claro
2: Item necessita de grande revisão para ser claro
3: Item necessita de pequena revisão para ser claro
4:Item claro

-Pertinência: avaliar se os itens refletem os conceitos envolvidos, se são relevantes e adequados para atingir objetivos almejados.
1: Não pertinente
2: Item necessita de grande revisão para ser relevante
3: Item necessita de pequena revisão para ser relevante
4: Item pertinente

1) Você já experienciou, algum acidente com perfurocortante, durante sua prática em serviços de saúde? Como isso influenciou a forma como você encara a prática com injetáveis?

Clareza: foi redigida de forma clara, compreensível e sem ambiguidade? *

- 1 - Não claro
- 2 - Item necessita de grande revisão para ser claro
- 3 - Item necessita de pequena revisão para ser claro
- 4 - Item claro

Pertinência: reflete os conceitos envolvidos, são relevantes e adequados à temática do estudo? *

- 1 - Não pertinente
- 2 - Item necessita de grande revisão para ser relevante
- 3 - Item necessita de pequena revisão para ser relevante
- 4 - Item pertinente

Sugestão de nova redação

Sua resposta

Observação

Sua resposta

2) Você costuma realizar treinamentos para manuseio de injetáveis com dispositivos de segurança? Conte um pouco sobre sua experiência com estes recursos.

Clareza: foi redigida de forma clara, compreensível e sem ambiguidade? *

- 1 - Não claro
- 2 - Item necessita de grande revisão para ser claro
- 3 - Item necessita de pequena revisão para ser claro
- 4 - Item claro

Pertinência: reflete os conceitos envolvidos, são relevantes e adequados à temática do estudo? *

- 1 - Não pertinente
- 2 - Item necessita de grande revisão para ser relevante
- 4 - Item pertinente

Sugestão de nova redação

Sua resposta

Observação

Sua resposta

Envie-me uma cópia das minhas respostas.

Voltar Enviar Página 4 de 4

Nunca envie senhas pelo Formulários Google.

reCAPTCHA
PrivacidadeTermos

Este conteúdo não foi criado nem aprovado pelo Google. Denunciar abuso - Termos de Serviço - Política de

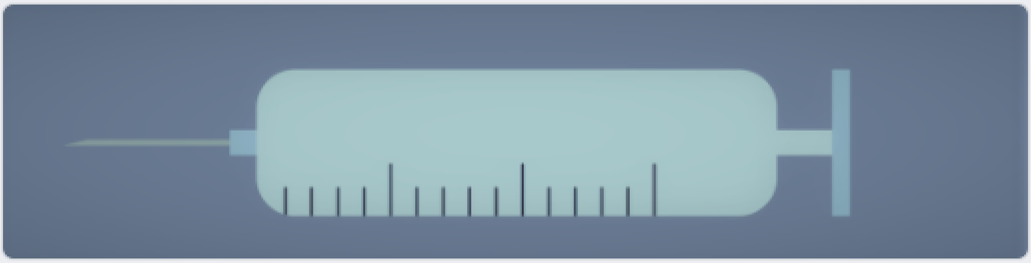

Validação de material educativo: Boas Práticas em Medicações Injetáveis

Agradeço pela sua participação!

Este conteúdo não foi criado nem aprovado pelo Google. Denunciar abuso - Termos de Serviço - Política de Privacidade

Google Formulários

APÊNDICE K – Ebook "Boas Práticas com Injetáveis: ações para o controle de infecção" validado

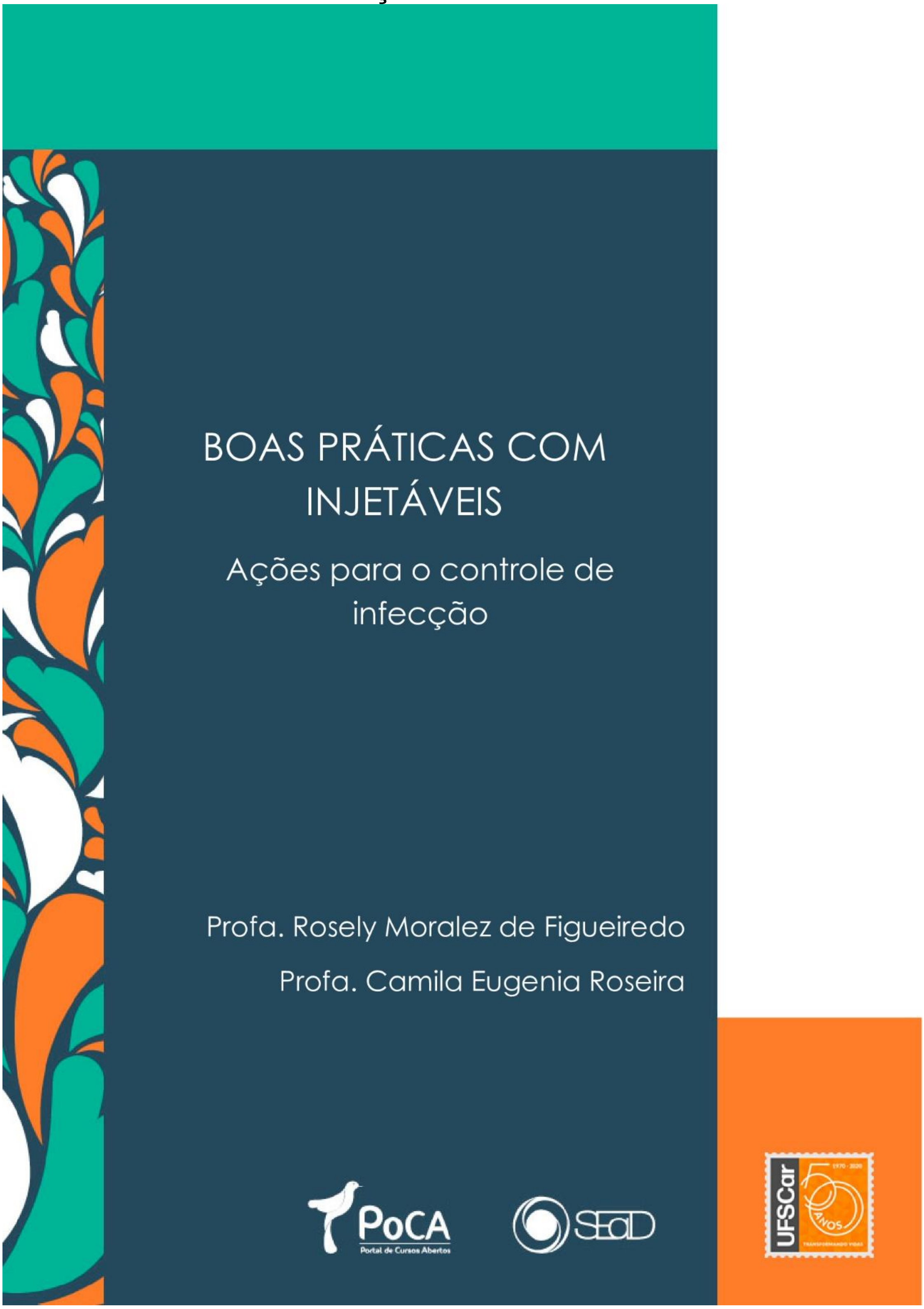

Apresentação

Olá! Este curso tem o objetivo de apoiar as práticas em serviços de saúde, a partir da elucidação de ações que contemplam a segurança e o controle de infecção para a manipulação e administração de medicações injetáveis.

Se você realiza estes procedimentos, seja profissional ou estudante, este curso é para você! Aqui você encontrará ações possíveis de inserção no seu dia-a-dia, e, com isso, tornar esta prática segura, tanto para você, quanto para o paciente e equipe de saúde que você integra. Vamos lá?

Boas Práticas com Injetáveis: ações para o controle de infecção

Além da técnica utilizada para a administração de injetáveis ou **injeções parenterais** (que englobam a via intramuscular, subcutânea ou endovenosa), é preciso se atentar a alguns fatores que podem comprometer a assepsia do procedimento e causar agravos ao paciente, sejam infecciosos ou não infecciosos como abcessos e reações tóxicas (RIGOTTI, 2012).

Você identifica quais fatores podem comprometer a administração injetável e causar estes danos ao paciente?

Talvez você tenha pensado em uma manipulação insegura dos insumos (por exemplo, manipular seringas e agulhas sem higienizar as mãos ou utilização de solução injetável previamente contaminada que não foi descartada oportunamente) uma antissepsia ineficaz previamente à administração de uma medicação intramuscular, ou ainda, a não identificação prévia de sinais locais que sugiram flebite em uma punção venosa já instalada.

Por falar em **flebite**, você sabe o que é e quais os seus sinais?

A flebite é uma irritação que ocorre na parede da veia, que pode ser mecânica (ocasionada por cateteres venosos de calibres maiores, geralmente), química (em decorrência do próprio medicamento infundido), relacionada às condições clínicas do paciente, bacteriana (quando há **quebra na técnica asséptica**) ou pós-infusão, quando surge após 48 de remoção do acesso venoso periférico (BRASIL, 2017, INFUSION NURSEES SOCIETY, 2016).
São sinais/sintomas desta inflamação: dor, sensibilidade, eritema, calor, inchaço, endurecimento, purulência ou cordão venoso palpável (INFUSION NURSEES SOCIETY, 2016)

Estes fatores podem ser prevenidos a partir de um planejamento prévio de ações e inserção de cuidados que devem estar em conformidade com as diretrizes da Organização Mundial da Saúde (OMS) e da Agência Nacional de Vigilância Sanitária (ANVISA).

Tanto as recomendações da OMS quanto da ANVISA foram consideradas na elaboração deste material. Tais recomendações devem ser seguidas por todos que administram injetáveis, pois assim, prevenimos infecções e tornamos o procedimento seguro para profissionais, estudantes da área da saúde e pacientes!

Higienização das mãos: elemento de destaque na prática com injetáveis!

A higienização das mãos, assim como ocorre em todos os procedimentos que permeiam a assistência segura, é primordial para reduzir o risco de infecção. Há comprovação científica da remoção de microrganismos a partir do emprego de água e sabão ou solução alcoólica (BRASIL, 2017).

Sobre a higienização das mãos, quando houver sujidade visível, recomenda-se a prática com água e sabão, para os demais casos, pode-se priorizar a fricção com solução alcoólica (60 a 80%) (BRASIL, 2018).

Em suma, não havendo a sujidade visível, você pode dar preferência a uma ou outra forma, segundo critérios pessoais, mas lembre-se de questões como tempo de ação (visto que o uso de álcool demanda metade do tempo da higienização das mãos com água e sabão), conforto a partir da qualidade da solução empregada e recursos disponíveis no local, como dispensadores a beira leito (BRASIL, 2018; BRASIL, 2017; WHO, 2010).

Já os momentos para a realização da higienização das mãos, para a prática com injetáveis, são sumarizados na figura a seguir (BRASIL, 2018; BRASIL, 2017; WHO, 2010):

Figura 1. Momentos da higienização das mãos para a prática com injetáveis. São Carlos-SP, 2020.

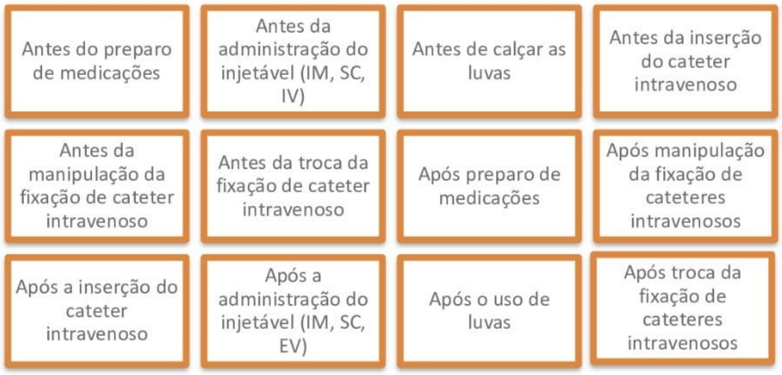

Fonte da imagem: elaborada pelas autoras.

Além da atenção à higienização das mãos, devemos identificar quais equipamentos de proteção individual (ou EPI) deveremos selecionar, para garantir que não soframos danos decorrentes destes procedimentos. Dentre estes, encontramos as luvas.

FIQUE ATENTO: o **uso de luvas** é complementar à higienização das mãos, não a substitui, já que o uso de luvas de procedimentos **protege apenas o profissional** do risco de exposição aos fluidos corporais do usuário ou potenciais microrganismos colonizadores deste, enquanto **a higienização das mãos protege a ambos.** A higienização das mãos é um mecanismo de redução de exposição e prevenção de patógenos (BRASIL, 2017; WHO, 2010)!

Considere que o uso de luvas não impede que o profissional ou estudante entre em contato com microrganismos, uma vez que microfuros podem estar presentes nas luvas, ou que se contamine durante a remoção deste equipamento, contudo, estes microrganismos são passíveis de remoção pela **higienização das mãos** que sucederá o procedimento e retirada das luvas: estas são complementares (BRASIL, 2017; WHO, 2010)!

Insumos para a prática com injetáveis

Se você já ouviu falar deste tema, é provável que você reconheça alguns elementos associados a esta prática e que citaremos ao longo do curso:

Figura 2. Componentes para a administração de injetáveis. São Carlos-SP, 2020.

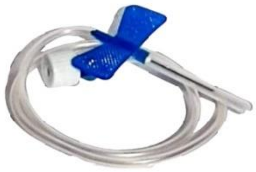

Cateter intravenoso agulhado (scalp)

Fonte da imagem: elaborada pelas autoras

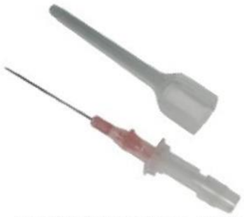

Cateter intravenoso flexível (abocath/jelco)

Fonte da imagem: elaborada pelas autoras

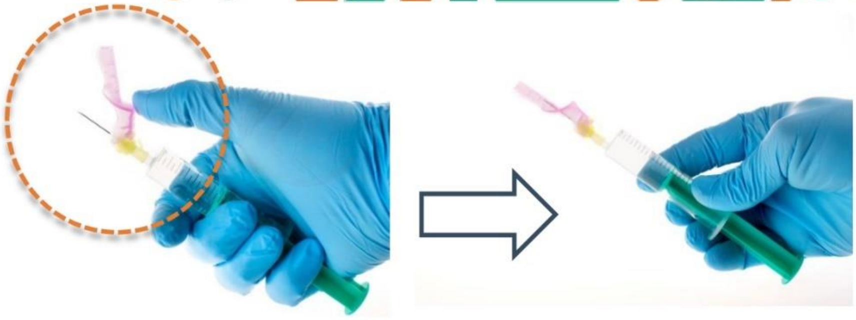

Seringa e agulha com dispositivo de segurança
Fonte das imagens: <https://www.istockphoto.com/br/foto/fechando-a-tampa-de-seguran%C3%A7a-de-uma-agulha-de-seringa-gm1210303083-350569156?clarity=false (modificada) e https://www.istockphoto.com/br/foto/tampa-de-seguran%C3%A7a-fechada-de-uma-agulha-gm1210303058-350569142?clarity=false>

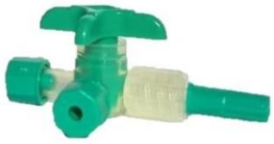

Dânula (três vias)
Fonte da imagem: elaborada pelas autoras

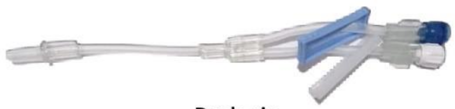

Dupla via
Fonte da imagem: elaborada pelas autoras

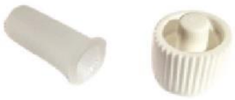

Oclusores para extensores e equipos
Fonte da imagem: elaborada pelas autoras

Você pode estar questionando sobre o tempo de troca destes dispositivos após sua inserção.

O recomendado é que não ocorra em um período inferior a 96 horas, contudo, requer avaliação contínua e frequente tanto das condições do paciente quanto do sítio de inserção do dispositivo (BRASIL, 2017).

Para pacientes neonatais e pediátricos, não se recomenda a troca rotineira, (BRASIL, 2017) sendo assim, a avaliação contínua se faz imprescindível para esta população.

Equipos e dispositivos complementares devem ser trocados a cada troca de cateter venoso periférico, caso não haja restrição de acordo com a solução administrada

(por exemplo, para administração de hemocomponentes, a troca destes deve ser a cada bolsa), quebra da integridade do produto ou suspeita de contaminação deste ou do sistema, o que enseja troca imediata (BRASIL, 2017).

Em caso de equipos para administração endovenosa intermitente, recomenda-se a troca a cada 24 horas (BRASIL, 2017).

Já se tratando dos oclusores, em caso de desconexão do sistema de infusão, sujidade ou sangue, o recomendado é que sejam imediatamente trocados (BRASIL, 2017).

Você percebe a importância das Boas Práticas na administração de injetáveis para a manutenção do dispositivo, conforto e segurança dos pacientes?

Tais elementos que estão presentes na prática com injetáveis precisam de manipulação asséptica, consideração à sua validade e seleção de acordo com a indicação de uso, conforme veremos a seguir.

A escolha dos insumos utilizados: qual a influência no controle de infecção?

Você sabia que a manutenção de uma assistência segura se inicia com o planejamento de nossas ações? Por exemplo, ao selecionarmos os insumos que utilizaremos durante uma administração de medicação injetável, poderemos inserir ações das Boas Práticas em nosso dia-a-dia.

Então, vamos a elas!

Pensando no controle do ambiente para o preparo de injetáveis, recomenda-se a manipulação dos insumos e soluções estéreis em uma superfície limpa, seja o balcão ou a própria bandeja (WHO, 2010).

Esta limpeza (que visa reduzir a carga microbiana) pode ser feita com solução alcoólica a 70%, pois sugere-se a sua eficácia mesmo em presença de superfícies contaminadas (GRAZIANO et al., 2012).

Aguarde a secagem espontânea e completa da superfície antes de utilizá-la como base para o preparo de injetáveis, garantindo, assim, o tempo de ação para que seja eficiente (WHO, 2010)!

Sobre os insumos utilizados, como seringas e agulhas, para a realização de medicações injetáveis, independentemente da via, estas devem estar dentro do prazo de validade, com embalagem íntegra e limpa. Devem ser utilizados para **um único procedimento** e em **um único paciente** (WHO, 2010).

RESSALTA-SE: a cada **novo procedimento**, **novos dispositivos** devem ser utilizados, garantindo sua esterilidade. Esta medida é muito importante para a segurança do

paciente, pois, a desatenção a estes eventos pode viabilizar a contaminação da solução a ser administrada, ocasionando danos como infecções de corrente sanguínea, abcessos, flebite, dentre outros (WHO, 2010). Ou seja, ao administrar medicações, independentemente da via, é preciso certificar-se de que a seringa e agulha a serem utilizadas não foram utilizadas em outros pacientes (WHO, 2010), anteriormente.

Isso pode parecer um cuidado trivial de ser mencionado, contudo, destaca-se que por compartilhar seringas entre pacientes, por desconhecimento do risco inerente a esta prática insegura, no Texas, houve um surto de hepatite B (VAN TUONG et al., 2017).

Ressalta-se que o **reuso de dispositivos é proibido** mesmo quando se tratar do mesmo paciente.

Embora o risco de infecção de corrente sanguínea associada ao cateter periférico seja um evento raro, sua ocorrência já foi associada a microrganismos gram-positivos (58%), gram-negativo (35,8%), Candida spp (6.2%), dentre outros. Além disso, identificou-se que pacientes com hemocultura positiva vieram a óbito dentro de 30 dias do diagnóstico, os quais apresentaram proporção maior de infecção por *Staphylococcus aureus* em relação aos sobreviventes (SATO et al., 2017).

É preciso, ainda, considerar o tipo dos frascos de soluções utilizados para a reconstituição de doses. Você sabe quais as implicações desta ação?

É preconizado que a diluição de medicamentos ocorra a partir de doses únicas para este fim e que não sejam compartilhadas bolsas ou frascos de solução para reconstituição de doses medicamentosas.

Por exemplo, ao se usar frasco de 100 ml de solução fisiológica 0,9% para diferentes reconstituições assume-se o risco de que, caso ocorra contaminação da solução, todas as doses a partir de então estarão contaminadas, também podendo ocasionar danos ao paciente. Portanto, para reconstituição de doses, utilize diluentes disponibilizados em dose única, ou ampolas (BRASIL, 2017).

Figura 3. Apresentação de diluente. São Carlos-SP, 2020.

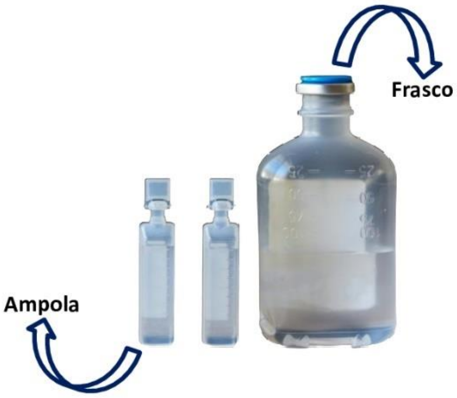

Fonte da imagem: <https://www.istockphoto.com/br/foto/um-jogo-do-saline-para-a-prepara%C3%A7%C3%A3o-da-inje%C3%A7%C3%A3o-antibi%C3%B3tica-gm1190410925-337454173?clarity=false> **(modificada)**

Partindo disto, ampolas de soro fisiológico a 0,9% podem ser utilizadas tanto para diluir medicações ou administrar medicações em bôlus quanto para remover o depósito sólido no lúmen do cateter a partir do *flushing* pulsátil (BRASIL, 2017).

O *flushing* pulsátil consiste na administração de soro fisiológico 0,9% com seringa de 10 ml (esta garante baixa pressão no lúmen do cateter e avalia fatores de resistência, sem danificá-lo) imediatamente antes e após cada uso (BRASIL, 2017).

Se você fechar o *clamp* e desconectar a seringa posteriormente a realização do *flushing*, haverá minimização do refluxo de sangue, mantendo o extensor (como o dupla via) sem resíduos orgânicos (BRASIL, 2017), prevenindo não apenas a obstrução e o mal funcionamento do cateter como a potencial colonização por microrganismos, reduzindo assim o risco de infecção de corrente sanguínea (FERRONI et al, 2014).

A mesma importância se dá ao manejo de frascos e ampolas contendo medicamentos.

Você sabe quais ações podem auxiliar a tornar o manuseio de frascos multidoses seguro?

É importante salientar que os **frascos multidoses** devem ser utilizados **apenas** quando não há alternativa e, se possível, para pacientes individualizados (ou seja, lembre-se de identificar sempre o rótulo, com nome do usuário, data e horário de abertura, volume de reconstituição e assinatura, assim você garante que sua solução será utilizada no tempo de validade estabelecido pelo fabricante) (BRASIL, 2017).

Deve-se manter técnica asséptica durante o manuseio e utilizar novos dispositivos sempre que for necessário aspirar nova dose, ou seja, utilize sempre nova seringa e agulha **a cada** aspiração medicamentosa, mesmo que seja dose complementar para o mesmo usuário (BRASIL, 2017).

Antes da aspiração do conteúdo de frascos (incluindo-se aqueles com dose única) deve-se realizar a desinfecção do septo, reduzindo-se o risco de contaminar a solução a ser aspirada. Em caso de dúvidas sobre esterilidade ou a forma de manuseio anterior, este frasco deve ser descartado (WHO, 2010).

Figura 4. Apresentação da solução medicamentosa. São Carlos-SP, 2020.

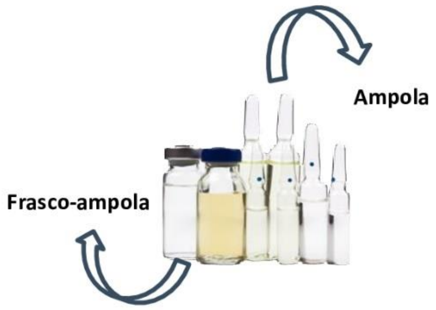

Fonte da imagem: <https://www.istockphoto.com/br/foto/ampolas-gm500945511-42976244?clarity=false> **(modificada)**

Após a manipulação do acesso venoso de pacientes que estejam em uso de cateteres venosos, será necessário realizar a troca dos oclusores, que também demanda manuseio asséptico. Então, leve com você oclusores esterilizados, com embalagem íntegra e de uso único que sejam compatíveis com o tipo de conexão da dânula ou dupla via (*luer lock*, que tem fechamento a partir de uma rosca, ou *luer slip*, com fechamento tipo encaixe), assim, não haverá risco de abertura acidental da linha venosa favorecendo exposição aos patógenos.

Figura 5. Exemplo de oclusores de dânula e equipo com fechamento tipo luer lock e luer slip, respectivamente. São Carlos-SP, 2020.

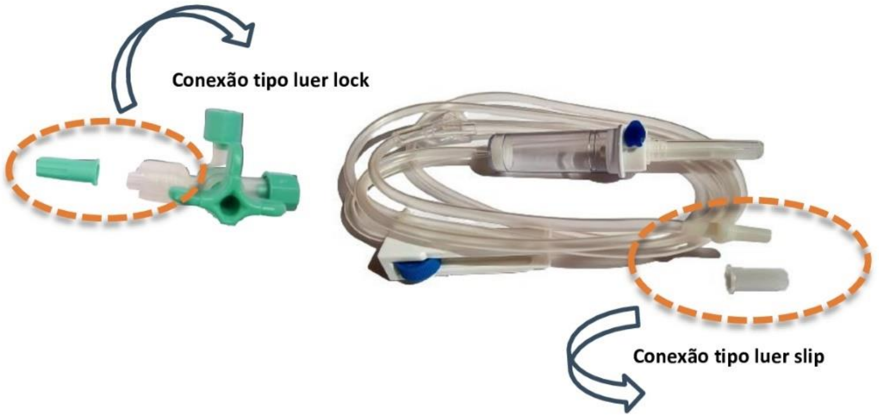

Fonte da imagem: elaborado pelas autoras.

E lembre-se: antes da manipulação, os oclusores do dispositivo intravenoso devem passar por desinfecção por solução alcoólica a 70% com tempo de contato entre 5 e 15 segundos (WHO, 2010)!

Você sabia?

A prevenção e controle da infecção de corrente sanguínea, uma Infecção Associada à Assistência à Saúde (IRAS), aborda ações que devem permear a prática de todos os membros da equipe de saúde e está em consonância com o Código de Ética dos Profissionais de Enfermagem e legislações que regem a profissão. Segue sugestão de leitura para aprofundamento no tema:

CONSELHO REGIONAL DO ESTADO DE SÃO PAULO. Código de Ética e Principais Legislações para o Exercício da Enfermagem. São Paulo. 2018. Disponível em <https://portal.coren-sp.gov.br/wp-content/uploads/2018/11/Codigo-de-etica.pdf >

As ações aqui abordadas auxiliam ao profissional ou estudante da área a manusear os dispositivos injetáveis a partir de uma técnica asséptica, mantendo sua esterilidade e realizando seu descarte de forma oportuna, identificando, a partir de uma atuação crítica, recursos preventivos e de controle de transmissão de patógenos para uma assistência segura ao paciente.

Se você está se perguntando sobre os cuidados após a administração medicamentosa e o que pode ser feito em caso de acidentes com perfurocortantes, convido a ouvir nosso podcast que abordará mais algumas ações passíveis de serem inseridas em nossa rotina de atuação dentro do serviço de saúde, disponível para conclusão deste curso.

Referências

BRASIL. *Medidas de Prevenção de Infecção Relacionada à Assistência à Saúde*. [S.l: s.n.], 2017.

BRASIL. Agência Nacional de Vigilância Sanitária. Nota Técnica Nº 01/2018 GVIMS/GGTES/ANVISA: Orientações Gerais para Higiene das Mãos em Serviços de Saúde. Brasília: ANVISA, 2018.

FERRONI, A *et al*. Pulsative flushing as a strategy to prevent bacterial colonization of vascular access devices. *Medical Devices: Evidence and Research*, vol. 7, pp. 379– 383, 2014.

GRAZIANO, M. *et al.* Eficácia da desinfecção com álcool 70% (p/v) de superfícies contaminadas sem limpeza prévia. *Rev. Latino-Am. Enfermagem*, v. 2, p. 06 telas, 2012.

INFUSION NURSES SOCIETY (US). Infusion nursing standards of practice. *Journal of Infusion Nursing*. vol.39, n. 1S, 2016.

RIGOTTI, M. A. *Segurança microbiológica na abertura de ampolas com ênfase no procedimento de desinfecção*. Dissertação (Mestrado). Escola de Enfermagem de Ribeirão Preto: São Paulo, 2012. 88f.

SATO, A. et al. Peripheral venous catheter-related bloodstream infection is associated with severe complications and potential death: A retrospective observational study. *BMC Infectious Diseases*, 2017.

VAN TUONG, P. et al. Assessment of injection safety in Ha Dong General Hospital, Hanoi, in 2012. *F1000Research*, vol.6, n2, pp.1–18, 2017.

WHO. *WHO Best practices for injections and related procedures toolkit*. Geneva, Suíça: [s.n.], 2010.

O material *Boas Práticas com Injetáveis: ações para o controle de infecção* de Rosely Moralez de Figueiredo e Camila Eugenia Roseira está licenciado com uma licença Creative Commons - Atribuição-CompartilhaIgual 4.0 Internacional.

Este curso tem o objetivo de apoiar as práticas em serviços de saúde, voltado para profissionais e estudantes de enfermagem, apresentando recomendações sobre o preparo e manipulação segura de injetáveis, sob a perspectiva do Controle de Infecção Relacionada à Assistência à Saúde e a Segurança Ocupacional.

PoCA
Portal de Cursos Abertos

http://poca.ufscar.br/

SEaD

UFSCar 50 anos 1970 · 2020 Transformando vidas

APÊNDICE L – Transcrição do áudio do podcast "Boas Práticas com Medicações Injetáveis: subsídios para o autocuidado"

Transcrição do áudio:

BOAS PRÁTICAS COM MEDICAÇÕES INJETÁVEIS: subsídios para o autocuidado

Prof.ª Dra. Rosely Moralez de Figueiredo

Ma. Camila Eugenia Roseira

Você sabia que as boas práticas com medicações injetáveis envolvem também, as ações para a segurança do profissional ou estudante que realizam estes procedimentos?

Sou Camila, doutoranda em Ciências da Saúde pela UFSCar e professora de enfermagem do Centro Paula Souza.

Gostaria de conversar com você a respeito de ações que devem ser inseridas na nossa rotina para tornar a prática mais segura. Para tanto vamos considerar as recomendações da Organização Mundial da Saúde e Agência Nacional de Vigilância Sanitária.

Que tal começarmos pelo uso de luvas? Gostaria de saber como este Equipamento de Proteção Individual se insere em sua rotina.

Temos que a adesão às luvas não depende da ciência sobre alguma doença pré0existente no paciente e que seja passível de transmissão via corrente sanguínea e sim, da possibilidade de exposição das nossas mãos ao sangue, que pode ou não ser veículo de microrganismos. Por isso, obrigatoriamente, devemos utilizar luvas de procedimento para todas as medicações via endovenosa (ANVISA, 2017; WHO, 2014).

Já para as injeções subcutâneas ou intramusculares, é sempre importante identificarmos as condições clínicas do paciente, do ambiente em que a injeção será administrada e das próprias mãos. (COREN0SP, 2010; MONCAIO; FIGUEIREDO, 2017; SECRETARIA DA SAÚDE DA CIDADE DE SÃO PAULO, 2015; WHO, 2010).

Devemos adotar o uso de luvas para administrar injetáveis por estas vias quando o paciente estiver em risco de sangramento associado a algum distúrbio de coagulação ou se ele estiver em precaução por contato (WHO, 2014).

Além disso, deveremos adotar o uso de luvas para a administração de injetáveis quando o ambiente em que a injeção for administrada envolver alguma situação de surto.

De qualquer forma, tome nota do Protocolo Operacional Padrão da instituição em que você atua e que aborde estes procedimentos, já que não há obrigatoriedade ao uso de luvas para injeções por via intramuscular e subcutânea (COREN0SP, 2010; MONCAIO; FIGUEIREDO, 2017; SÃO PAULO (CIDADE). SECRETARIA DA SAÚDE, 2015; WHO, 2010).

Quanto à avaliação das mãos procure sempre observar a presença de lesões. Se suas mãos apresentarem lesões pequenas e superficiais, utilize luvas para todas as injeções que realizar, independentemente da via, e higienize as mãos com água e sabão para tornar a prática mais segura e confortável para você (WHO, 2010).

Mas, caso você apresente lesões maiores, o recomendado é uma avaliação prévia para prevenir que haja exposição aos fluidos do paciente durante algum procedimento que você vá realizar (WHO, 2010).

E sobre a prevenção de acidentes com perfurocortantes? Você sabe quais as ações necessárias?

O recomendado é não reencapar agulhas com a própria tampa ou desconectá-las manualmente após a sua utilização, porque muitos acidentes ocorrem devido a estas ações (ANVISA, 2017; WHO, 2010).

Falando nisso, é importante citar a adesão aos dispositivos de segurança. Estes são recursos que visam cobrir a agulha após utilização com uma capa protetora ou com o próprio corpo da seringa, tornando, assim, o manuseio mais seguro. Para isto, esteja atento aos treinamentos que venham a ocorrer em seu ambiente de trabalho ou estudo e busque informações e cursos sobre esta prática.

Embora essas medidas previnam a grande maioria dos acidentes por perfurocortantes, é possível que você, ainda, experiencie algum evento como este em sua rotina.

E aí? Você sabe quais ações são necessárias, caso isso ocorra?

Se você pensou em acolher a vítima, cuidar da lesão, reportar e notificar o evento ao serviço de saúde... Você acertou!

Após o acidente com perfurocortante, havendo ou não perda sanguínea, seja uma lesão superficial ou profunda, haja ou não uso de luvas durante o procedimento, se envolveu material biológico de outra pessoa, o evento deve ser reportado e notificado ao serviço.

Essa ação é necessária porque a vítima pode precisar de tratamento ou acompanhamento (o qual pode perdurar cerca de seis meses ou mais).

Um momento para abrir parênteses a uma informação muito importante: não há relação entre o acidente e culpa para a vítima, portanto, o manejo acertado e o apoio institucional são primordiais caso estes venham a ocorrer, pois reduzem tanto o risco de transmissão de patógenos quanto a ansiedade decorrente deste evento, devendo ser aderidos precocemente. Assim eles vão minimizar a ocorrência de agravos como a transmissão de infecções virais, por exemplo, hepatite B, C e HIV (WHO, 2010).

E por falar em hepatite B, como está sua carteira de vacinação? Completa? A carteira de vacinação atualizada contra essa doença é a medida mais eficaz para a prevenção de transmissão do vírus em caso de acidentes (WHO, 2010).

Já considerando a lesão ocasionada por perfurocortante, esta requer uma limpeza da área afetada com água corrente e sabão... E nada além disso! Aplicar álcool no local ou espremer a área pode irritar a pele e aumentar a lesão, ou seja, não haverá redução da carga microbiana que adentrou ao tecido epitelial (WHO, 2010).

Portanto, ao presenciar um acidente com perfurocortante mantenha a calma e adote as medidas para acompanhamento, tratamento e notificação deste evento (WHO, 2010)!

Espero que este conteúdo ajude você a fazer escolhas seguras e conscientes durante a sua prática.

Referências

BRASIL. Medidas de Prevenção de Infecção Relacionada à Assistência à Saúde. 2017.
Disponível em:
<http://portal.anvisa.gov.br/documents/33852/271855/Medidas+de+Prevenção+de+Infecçã
o+Relacionada+à+Assistência+à+Saúde/6b16dab306d0c0439909d840141d2e81c809>.

COREN-SP. Parecer Coren0Sp no 019/2010 Dispõe sobre o uso de luvas de procedimento para administração de medicamentos. Disponível em: <http://portal.corensp.gov.br/sites/default/files/parecer_coren_sp_2010_18_0.pdf>. , 2010

GRAZIANO, M. et al. Eficácia da desinfecção com álcool 70% (p/v) de superfícies contaminadas sem limpeza prévia. Rev. Latino0Am. Enfermagem, v. 2, p. 06 telas, 2012.

MONCAIO, A. C. S.; FIGUEIREDO, R. M. DE. Conhecimentos e práticas no uso do cateter periférico intermitente pela equipe de enfermagem. Revista Eletrônica de Enfermagem, 2017.

O'GRADY, N. P. et al. Guidelines for the prevention of intravascular catheter0related infections. Clinical infectious diseases : an official publication of the Infectious Diseases Society of America, 2011.

SÃO PAULO (CIDADE). SECRETARIA DA SAÚDE. Manual de normas, rotinas e procedimentos de Enfermagem0 Atenção Básica. Secretaria Municipal de São Paulo. [S.l: s.n.]. Disponível em: <http://www.prefeitura.sp.gov.br/cidade/secretarias/upload/saude/arquivos/legislacao/Nor maseRotinas02102015.pdf>. 2015

WHO. Glove Use Information Leaflet. Patient Safety, 2014.

WHO. WHO Best practices for injections and related procedures toolkit. Geneva, Suíça: [s.n.], 2010.

Música:

Funny by MusicLFiles
Link: https://filmmusic.io/song/63880funny

License: http://creativecommons.org/licenses/by/4.0/

APÊNDICE M – Questões Avaliativas do curso “Boas Práticas com Injetáveis: ações para o controle de infecção”

Questionário para avaliação do conteúdo e certificação do participante sobre “Boas Práticas com Medicações Injetáveis: ações para o controle de infecção

Questão 1

Ainda não respondida

Vale 1,00 ponto(s).

Marcar questão

Editar questão

Segundo a prescrição médica, a técnica de enfermagem Ana, deverá administrar uma ampola de dipirona monossódica (500mg/ml) diluída em 20 ml de solução fisiológica 0,9% (SF 0,9%), via endovenosa. Para a diluição da medicação, há ampolas contendo 10 ml e frascos de 100 e 250 ml de SF 0,9%. Pensando no controle de infecção é correto que Ana:

Escolha uma opção:

- ○ Selecione o frasco de 100 ml para aspirar o total de diluente (20 ml de SF 0,9%) e armazene o restante do frasco para diluições futuras (para o mesmo paciente).
- ○ Selecione 2 ampolas de SF 0,9% para diluir a dipirona, descartando os resíduos em seguida.
- ○ Compartilhe o conteúdo de algum frasco com grande volume de SF 0,9% para fazer a diluição da medicação deste paciente e utilizar o restante para diluir medicações de outros pacientes.
- ○ Diluir em um frasco de 100 ml de SF 0,9%, uma vez que não há dose unitária com o volume de diluente prescrito.
- ○ Diluir com uma ampola de SF 0,9% (10 ml), pois, é o volume unitário disponível.

Próxima página

Questão 2

Ainda não respondida

Vale 1,00 ponto(s).

Marcar questão

Editar questão

A higienização das mãos é primordial para a redução de infecção em serviços de saúde, pois, remove os microrganismos a partir do emprego de água e sabão ou solução alcoólica. Considere as afirmações a seguir:

I. Deve-se higienizar as mãos antes da inserção do cateter venoso a fim de prevenir algum dano infeccioso no paciente.
II. Como, obrigatoriamente, deve-se utilizar luvas de procedimento para realizar o procedimento, é facultativa a higienização das mãos após a retirada deste equipamento de proteção individual.
III. Deve-se higienizar as mãos após a manipulação de cateteres venosos periféricos para prevenir a contaminação das mãos daquele que realizou esta ação.

Assinale as alternativas que contenham as afirmações corretas:

Escolha uma opção:

- I, II e III
- I e II
- I e III
- II e III
- Apenas II

Página anterior

Próxima página

Questão 3
Ainda não respondida
Vale 1,00 ponto(s).
Marcar questão
Editar questão

Complete o parágrafo com as palavras corretas:

[ ] é o termo utilizado para a administração de solução fisiológica com seringa de 10 ml para gerar uma [ ] pressão no lúmen do cateter intravenoso, a fim de manter sua integridade e [ ] , prevenir obstrução e [ ] de microrganismos. Deve-se realizar o procedimento antes e após cada administração endovenosa. É importante utilizar seringas [ ] pois o reuso é [ ] mesmo se tratando de procedimento no mesmo paciente.

Flushing	baixa	funcionalidade	colonização	estéreis	proibido

Página anterior

Próxima página

Questão 4
Ainda não respondida
Vale 1,00 ponto(s).
Marcar questão
Editar questão

Ao realizar uma medicação intramuscular em uma Unidade de Pronto Atendimento (UPA), Fernando acaba perfurando sua mão, ao reencapar a agulha já utilizada. Como ele é recém-contratado, está receoso de sofrer alguma sanção ao reportar o ocorrido ao serviço, já que, na ocasião, não estava utilizando luvas de procedimento.
Analise as colocações a seguir:

I) É obrigatório o uso de luvas para qualquer injeção intramuscular.
II) Caso ele esteja com o esquema vacinal completo contra hepatite B, não há risco algum neste evento acidental.
III) O evento deve ser reportado e notificado ao serviço, pois, há riscos à saúde do profissional.
IV) O evento deve ser reportado e notificado ao serviço, pois, há riscos à saúde do profissional e do paciente que recebeu a injeção.

É correto o que se afirma em:

Escolha uma opção:

- I e III
- Apenas IV
- I e II
- Apenas III
- II e IV

Página anterior

Próxima página

Questão 5
Ainda não respondida
Vale 1,00 ponto(s).
Marcar questão
Editar questão

"O álcool a 70% é uma solução muito importante nos serviços de saúde. Além de seu reconhecimento enquanto desinfetante, podendo ser utilizado na limpeza de bandejas e superfícies, pode ser empregado para a higienização das mãos, otimizando o tempo para esta ação, e também aplicado às lesões decorrentes de acidentes com perfurocortantes, já que reduz a quantidade de microrganismos que podem ter adentrado à pele do profissional, sendo assim, a solução de escolha para o primeiro cuidado com lesões deste tipo".

Escolha uma opção:

- Esta afirmação é parcialmente verdadeira, pois, não se deve aplicar solução alcoólica às lesões por perfurocortante, visto potencial irritante.
- Esta afirmação é totalmente verdadeira.
- Esta afirmação é parcialmente verdadeira, visto que não se deve empregar solução alcoólica para a limpeza de bandejas, já que não abrange microrganismos que possam estar nestes materiais.
- Esta afirmação é totalmente falsa.
- Esta afirmação é parcialmente verdadeira, visto que o tempo de secagem do álcool a 70%, comparativamente à higienização das mãos com álcool e sabão, não apresenta vantagem.

Página anterior

Finalizar tentativa ...

APÊNDICE N – Questionário para identificação da percepção da população-alvo sobre o curso “Boas Práticas com Injetáveis: ações para o controle de infecção”

Questionário sobre a percepção do curso: Boas Práticas em Medicações Injetáveis

Olá!
Você está sendo convidado(a) para avaliar, conforme sua percepção, o curso "Boas Práticas em Medicações Injetáveis" disponível no Portal de Cursos Abertos - PoCA (https://cursos.poca.ufscar.br/course/view.php?id=79).
Sua participação nesta pesquisa não influencia na conclusão do curso “Boas Práticas em Medicações Injetáveis” e tampouco tem relação com a avaliação para a emissão do certificado, contudo, contribuirá para identificarmos como o material sobre Boas Práticas em Medicações Injetáveis se comporta enquanto estratégia educativa.
Este material foi elaborado como etapa final de uma tese de doutorado, após devolutiva de questionário auto-aplicado por 1295 profissionais de enfermagem do estado de São Paulo distribuído eletronicamente, no qual foi identificado que, embora haja conhecimento dentro da prática segura com injetáveis, há práticas que demandam um aprofundamento no tema, como: relação entre uso de luvas com as condições clínicas do paciente e risco de contato aos fluidos independentemente da presença de doenças transmissíveis pela corrente sanguínea, higienização das mãos em todas as etapas do processo com injetáveis para garantir a segurança de todos os envolvidos, proibição ao reuso de seringas e agulhas e cuidados para o profissional frente ao manejo com perfurocortantes.
O curso, que contempla este material educativo, objetiva subsidiar a prática com injetáveis em serviços de saúde, visando o preparo e a manipulação segura de injetáveis de acordo com os princípios de biossegurança, controle e prevenção de infecções relacionadas à assistência em saúde, tendo por foco a equipe de enfermagem.
Ressalta-se que se trata de um curso sem tutoria, ou seja, prioriza-se o autodidatismo do participante.
Leia atentamente o Termo de Consentimento Livre e Esclarecido e, concordando em participar, clique em aceitar.
Sua participação é muito importante!
Agradeço sua disponibilidade e atenção.

*Obrigatório

Endereço de e-mail *

Seu e-mail

Termo de Consentimento Livre e Esclarecido *

UNIVERSIDADE FEDERAL DE SÃO CARLOS
DEPARTAMENTO DE ENFERMAGEM
PROGRAMA DE PÓS GRADUAÇÃO EM ENFERMAGEM

TERMO DE CONSENTIMENTO LIVRE E ESCLARECIDO

(Resolução 466/2012 do CNS)

BOAS PRÁTICAS EM MEDICAÇÕES INJETÁVEIS: UMA INTERVENÇÃO EDUCATIVA PARA A EQUIPE DE ENFERMAGEM NO ESTADO DE SÃO PAULO

Prezado(a), o senhor(a) está sendo convidado(a) para participar da pesquisa "Boas práticas em medicações injetáveis: uma intervenção educativa para a equipe de enfermagem no estado de São Paulo". Esta pesquisa se justifica pela literatura científica, que aponta para a necessidade emergente de estratégias que aperfeiçoem a adesão às boas práticas de medicações injetáveis.

O objetivo deste estudo é desenvolver e validar uma estratégia educativa para profissionais de enfermagem visando às boas práticas na administração de medicações injetáveis. O material educativo anteriormente citado refere-se ao curso aberto "Boas Práticas em Medicações Injetáveis".

O curso "Boas Práticas em Medicações Injetáveis" aborda as boas práticas de medicações injetáveis a partir de um diagnóstico situacional realizado previamente no estado de São Paulo, do qual participaram profissionais de enfermagem deste estado, e objetiva o aperfeiçoamento e a implantação de medidas que minimizam o risco de infecção relacionada à assistência à saúde.

O (a) senhor(a) foi selecionado(a) por ser um(a) aluno(a) do curso "Boas Práticas em Medicações Injetáveis", ter mais de 18 anos e ser profissional ou estudante de enfermagem. Sua participação é voluntária e consiste em preencher um questionário abordando seus dados sociodemográficos e sua percepção sobre o curso em questão. O tempo utilizado para responder ao questionário será de aproximadamente 45 minutos.

A qualquer momento o (a) senhor(a) pode desistir de participar e retirar seu consentimento. A sua recusa não trará nenhum prejuízo na sua relação com o pesquisador ou com a instituição. Em caso de esclarecimentos quanto à pesquisa, poderá entrar em contato com a pesquisadora pelo telefone ou e-mail abaixo deste termo, a qualquer momento.

Ressalta-se que a sua participação nesta pesquisa não influencia na conclusão do curso "Boas Práticas em Medicações Injetáveis" e tampouco tem relação com a avaliação para a emissão do certificado.

Suas respostas serão tratadas de forma anônima e confidencial em qualquer fase do estudo, sendo sua privacidade assegurada. Os dados coletados poderão ter seus resultados divulgados em eventos, revistas e/ou trabalhos científicos.

O preenchimento destes questionários não oferece risco imediato ao (a) senhor (a), porém considera-se a possibilidade de um risco subjetivo, pois algumas perguntas podem levar a sentir-se desconfortável, evocar sentimentos ou lembranças desagradáveis, sentir-se cansado após responder o questionário. Caso algumas dessas possibilidades ocorram, o senhor (a) poderá optar pela suspensão imediata de sua participação nesta pesquisa.

O senhor (a) não terá nenhum custo ou compensação financeira ao participar do estudo. Contudo, ressalta-se que a sua participação auxiliará na reflexão sobre como o material sobre Boas Práticas em Medicações Injetáveis se comporta enquanto estratégia educativa segundo a percepção dos profissionais ou estudantes da área da enfermagem. Há direito a indenização por qualquer tipo de dano comprovadamente resultante da sua participação na pesquisa.

Ao assinalar eletronicamente que aceita participar da pesquisa, o (a) senhor (a) receberá por e-mail uma via assinada e rubricada deste termo onde consta o telefone e o endereço do pesquisador principal, podendo tirar suas dúvidas sobre o projeto e sua participação, agora ou a qualquer momento.

Declaro que entendi os objetivos, riscos e benefícios de minha participação na pesquisa e concordo em participar. O pesquisador me informou que o projeto foi aprovado pelo Comitê de Ética em Pesquisa em Seres Humanos da UFSCar que funciona na Pró-Reitoria de Pesquisa da Universidade Federal de São Carlos, localizada na Rodovia Washington Luiz, Km. 235 - Caixa Postal 676 - CEP 13.565-905 - São Carlos - SP – Brasil. Fone (16) 3351-8028. Endereço eletrônico: cephumanos@ufscar.br

Endereço para contato

Pesquisador Responsável: Camila Eugenia Roseira

Endereço: UFSCar, Rod. Washington Luís, Km 235, São Carlos/SP

Contato telefônico: (16) 3351-8338. e-mail: roseira.camila@gmail.com

○ Li o Termo de Consentimento Livre e Esclarecido e aceito participar.

○ Não aceito

Endereço de e-mail (para recebimento do TCLE assinado) *

Sua resposta

Idade *

Sua resposta

Sexo *

- ○ Feminino
- ○ Masculino

Se estudante, qual o seu curso? *

- ◯ Graduação de enfermagem
- ◯ Curso técnico de enfermagem
- ◯ Pós-graduação de enfermagem
- ◯ Não se aplica

Se profissional, qual sua categoria? *

- ◯ Enfermeiro
- ◯ Técnico de enfermagem
- ◯ Auxiliar de enfermagem
- ◯ Não se aplica

Qual a sua cidade e estado? *

Sua resposta

Por qual dispositivo acessou ao curso: *

- ☐ Celular
- ☐ Computador
- ☐ Tablet
- ☐ Outro: ____________________

Questionário sobre a percepção do curso

Avalie cada critério conforme a gradação de concordância de atendimento ao item, conforme sua percepção no decorrer do curso.

O conteúdo abordado pelo material educativo está relacionado ao objetivo do curso *

- ◯ Concordo totalmente
- ◯ Concordo parcialmente
- ◯ Não concordo, nem discordo
- ◯ Discordo parcialmente
- ◯ Discordo totalmente

O conteúdo abordado pelo curso é relevante para a prática com injetáveis em serviços de saúde *

- ◯ Concordo totalmente
- ◯ Concordo parcialmente
- ◯ Não concordo, nem discordo
- ◯ Discordo parcialmente
- ◯ Discordo totalmente

O conteúdo abordado é útil para os participantes do curso *

- Concordo totalmente
- Concordo parcialmente
- Não concordo, nem discordo
- Discordo parcialmente
- Discordo totalmente

O conteúdo é exposto de forma clara e coerente *

- Concordo totalmente
- Concordo parcialmente
- Não concordo, nem discordo
- Discordo parcialmente
- Discordo totalmente

A quantidade de carga horária sugerida é suficiente para abordar ao conteúdo *

- Concordo totalmente
- Concordo parcialmente
- Não concordo, nem discordo
- Discordo parcialmente
- Discordo totalmente

O material educativo possui linguagem clara, objetiva e dialógica *

- ◯ Concordo totalmente
- ◯ Concordo parcialmente
- ◯ Não concordo, nem discordo
- ◯ Discordo parcialmente
- ◯ Discordo totalmente

O curso possui uma interface simples e de fácil manuseio *

- ◯ Concordo totalmente
- ◯ Concordo parcialmente
- ◯ Não concordo, nem discordo
- ◯ Discordo parcialmente
- ◯ Discordo totalmente

O curso apresenta-se de forma atrativa *

- ◯ Concordo totalmente
- ◯ Concordo parcialmente
- ◯ Não concordo, nem discordo
- ◯ Discordo parcialmente
- ◯ Discordo totalmente

A sequência do conteúdo está organizada de forma lógica *

- ○ Concordo totalmente
- ○ Concordo parcialmente
- ○ Não concordo, nem discordo
- ○ Discordo parcialmente
- ○ Discordo totalmente

O participante é estimulado a desenvolver um papel ativo e independente durante o manuseio do material do curso *

- ○ Concordo totalmente
- ○ Concordo parcialmente
- ○ Não concordo, nem discordo
- ○ Discordo parcialmente
- ○ Discordo totalmente

O participante é estimulado a refletir criticamente sobre a prática com injetáveis em serviços de saúde *

- ◯ Concordo totalmente
- ◯ Concordo parcialmente
- ◯ Não concordo, nem discordo
- ◯ Discordo parcialmente
- ◯ Discordo totalmente

O curso instiga para o aprofundamento teórico do tema *

- ◯ Concordo totalmente
- ◯ Concordo parcialmente
- ◯ Não concordo, nem discordo
- ◯ Discordo parcialmente
- ◯ Discordo totalmente

O material educativo do texto "Boas Práticas com Injetáveis: ações para o controle de infecção" está distribuído de forma harmônica no espaço limitado à página (texto e imagens) *

- Concordo totalmente
- Concordo parcialmente
- Não concordo, nem discordo
- Discordo parcialmente
- Discordo totalmente

O material educativo do texto "Boas Práticas com Injetáveis: ações para o controle de infecção" possui texto de fácil leitura *

- Concordo totalmente
- Concordo parcialmente
- Não concordo, nem discordo
- Discordo parcialmente
- Discordo totalmente

O material educativo do texto "Boas Práticas com Injetáveis: ações para o controle de infecção" transmite a mensagem de forma clara e concisa *

- Concordo totalmente
- Concordo parcialmente
- Não concordo, nem discordo
- Discordo parcialmente
- Discordo totalmente

As imagens do material educativo do texto "Boas Práticas com Injetáveis: ações para o controle de infecção" são significativas para a aprendizagem *

- Concordo totalmente
- Concordo parcialmente
- Não concordo, nem discordo
- Discordo parcialmente
- Discordo totalmente

Os links disponibilizados para acesso complementam o material do curso *

- Concordo totalmente
- Concordo parcialmente
- Não concordo, nem discordo
- Discordo parcialmente
- Discordo totalmente

O áudio do podcast "Boas práticas com medicações injetáveis: subsídios para o autocuidado" apresenta fluidez sonora e boa dicção *

- Concordo totalmente
- Concordo parcialmente
- Não concordo, nem discordo
- Discordo parcialmente
- Discordo totalmente

O fórum possibilita interação entre os participantes do curso *

- Concordo totalmente
- Concordo parcialmente
- Não concordo, nem discordo
- Discordo parcialmente
- Discordo totalmente

O fórum favorece o aprendizado e o compartilhamento de conhecimento entre os participantes *

- Concordo totalmente
- Concordo parcialmente
- Não concordo, nem discordo
- Discordo parcialmente
- Discordo totalmente

A atividade proposta para emissão do certificado é pertinente com os conteúdos trabalhados no material sobre Boas Práticas com Injetáveis *

- Concordo totalmente
- Concordo parcialmente
- Não concordo, nem discordo
- Discordo parcialmente
- Discordo totalmente

A atividade proposta para emissão do certificado proporciona a relação entre a teoria e a prática com injetáveis *

- ◯ Concordo totalmente
- ◯ Concordo parcialmente
- ◯ Não concordo, nem discordo
- ◯ Discordo parcialmente
- ◯ Discordo totalmente

A atividade proposta para a emissão do certificado é motivadora e estimula a resolução com entusiasmo *

- ◯ Concordo totalmente
- ◯ Concordo parcialmente
- ◯ Não concordo, nem discordo
- ◯ Discordo parcialmente
- ◯ Discordo totalmente

As ferramentas, como áudio, links e carregamento de imagens apresentam boa funcionalidade *

- Concordo totalmente
- Concordo parcialmente
- Não concordo, nem discordo
- Discordo parcialmente
- Discordo totalmente

Você tem alguma sugestão de melhoria ou observação sobre o curso? *

Sua resposta

Envie-me uma cópia das minhas respostas.

Enviar

Página 1 de 1

Nunca envie senhas pelo Formulários Google.

reCAPTCHA
PrivacidadeTermos

Este conteúdo não foi criado nem aprovado pelo Google. Denunciar abuso - Termos de Serviço - Política de Privacidade

Google Formulários

Você tem alguma sugestão de melhoria ou observação sobre o curso? *

Sua resposta

Envie-me uma cópia das minhas respostas.

Enviar

Página 1 de 1

Nunca envie senhas pelo Formulários Google.

reCAPTCHA
PrivacidadeTermos

Este conteúdo não foi criado nem aprovado pelo Google. Denunciar abuso - Termos de Serviço - Política de Privacidade

Google Formulários

ANEXO A – Declaração emitida pelo Conselho Regional de Enfermagem referente a autorização para divulgação do Questionário para Identificação das Boas Práticas com Medicações Injetáveis via eletrônica aos inscritos de sua jurisdição

Coren SP
Conselho Regional de Enfermagem de São Paulo

CARTA DE AUTORIZAÇÃO

Ao Comitê de Ética em Pesquisa em Seres Humanos da Universidade Federal de São Carlos (UFSCar),

Prezado Comitê de Ética em Pesquisa da UFSCar, na função de Vice Presidente do Conselho Regional de Enfermagem do Estado de São Paulo, informo estar ciente e de acordo com a participação do COREN-SP na divulgação do projeto de pesquisa intitulado *"Boas práticas em medicações injetáveis: intervenção educativa para a equipe de enfermagem no estado de São Paulo"* apresentado pelas pesquisadoras **Camila Eugênia Roseira e Rosely Moralez de Figueiredo.**

A participação do COREN se dará por meio do envio de convite para participação no estudo, por via eletrônica, aos profissionais registrados no referido conselho. Reforço ainda que isso será realizado apenas após apresentação do parecer favorável a realização da pesquisa emitido pelo Comitê de Ética em Pesquisa em Seres Humanos da UFSCar.

Prof. Dr. Mauro Antônio Pires Dias da Silva
Vice-Presidente do COREN/SP

São Paulo, 12 de junho de 2017.

ANEXO B – Aprovação do projeto pelo Comitê de Ética e Pesquisa com Seres Humanos da Universidade Federal de São Carlos

UFSCAR - UNIVERSIDADE FEDERAL DE SÃO CARLOS

Plataforma Brasil

PARECER CONSUBSTANCIADO DO CEP

DADOS DO PROJETO DE PESQUISA

Título da Pesquisa: BOAS PRÁTICAS EM MEDICAÇÕES INJETÁVEIS: INTERVENÇÃO EDUCATIVA PARA A EQUIPE DE ENFERMAGEM NO ESTADO DE SÃO PAULO

Pesquisador: Camila Eugenia Roseira

Área Temática:

Versão: 2

CAAE: 68147317.5.0000.5504

Instituição Proponente: Programa de Pós-Graduação em Enfermagem

Patrocinador Principal: Financiamento Próprio

DADOS DO PARECER

Número do Parecer: 2.139.384

Apresentação do Projeto:

Trata-se de estudo desenvolvido em 3 etapas, o qual tematiza as boas práticas na administração de medicações injetáveis e volta-se ao desenvolvimento e validação de estratégia educativa em larga escala.

Objetivo da Pesquisa:

Primário: Desenvolver e validar estratégia educativa, para aplicação em larga escala, visando aumentar a adesão às boas práticas na administração de medicações injetáveis.

Secundário: (1) identificar fatores relacionados à práxis da administração de medicações injetáveis entre os profissionais de enfermagem do estado de São Paulo.(2) desenvolver estratégia educativa para os profissionais de enfermagem, pautada nas necessidades identificadas sobre as boas práticas para medicações injetáveis.(3) validar por especialistas a estratégia educativa (aparência e conteúdo) desenvolvida sobre boas práticas de medicações de injetáveis.

Avaliação dos Riscos e Benefícios:

Assume risco mínimo em todas as etapas para os participantes e, sintetiza o mesmo da ordem de sentir-se desconfortável para responder algum item do questionário, cansaço mediante o tempo

Endereço: WASHINGTON LUIZ KM 235
Bairro: JARDIM GUANABARA **CEP:** 13.565-905
UF: SP **Município:** SAO CARLOS
Telefone: (16)3351-9683 **E-mail:** cephumanos@ufscar.br

gasto para responder ao mesmo. Descreve que diante estes riscos poderá o participante parar de preencher o questionário, retornando ao mesmo no desejo de.
Quanto aos benefícios, aponta conhecimento do contexto atual das boas práticas de medicações injetáveis no estado de São Paulo, bem como a disponibilização de material
educativo embasado no mesmo, que será disponibilizado eletronicamente.

Comentários e Considerações sobre a Pesquisa:
As etapas do estudo são: (1) primeira etapa do estudo: elaboração de questionário pautada em literatura na temática, o qual será avaliado com fins de validação, por juízes; (2)segunda etapa: estudo quantitativo descritivo desenvolvido a partir da distribuição eletrônica do questionário validado na etapa anterior para todos os profissionais de enfermagem inscritos no Conselho Regional de Enfermagem de São Paulo; (3) terceira etapa:faz uso dos dados obtidos na etapa 2 e se volta a desenvolver material educativo eletrônico baseado na Teoria da Aprendizagem Significativa proposta por David Ausbel. Esse material será, posteriormente, submetido à validação de conteúdo e de face por juízes. Cada etapa, em termos de participantes, descrevem-se critérios distintos. Prospecta 9 juízes para a 1a e 3a etapa e cerca de 500 participantes para a 2a etapa.

Considerações sobre os Termos de apresentação obrigatória:
Apresenta adequadamente todos os termos obrigatórios, inclusive o Termo de anuência da instituição parceira faltante na versão anteriormente apresentada.

Recomendações:

Conclusões ou Pendências e Lista de Inadequações:
Não há pendências, recomendo aprovação.

Considerações Finais a critério do CEP:
O material apresentado está em concordância com as resoluções que dão diretriz e orientação à pesquisa com seres humanos. Recomendo aprovação.

Este parecer foi elaborado baseado nos documentos abaixo relacionados:

Tipo Documento	Arquivo	Postagem	Autor	Situação
Informações	PB_INFORMAÇÕES_BÁSICAS_DO_P	13/06/2017		Aceito

UFSCAR - UNIVERSIDADE FEDERAL DE SÃO CARLOS

Continuação do Parecer: 2.139.384

Básicas do Projeto	ETO_907642.pdf	10:38:10		Aceito
Outros	Resposta.docx	13/06/2017 10:36:30	Rosely Moralez de Figueiredo	Aceito
Declaração de Instituição e Infraestrutura	Anuencia.pdf	13/06/2017 10:33:33	Rosely Moralez de Figueiredo	Aceito
TCLE / Termos de Assentimento / Justificativa de Ausência	TCLE_JUIZES_ETAPA_3.pdf	24/04/2017 16:18:08	Camila Eugenia Roseira	Aceito
TCLE / Termos de Assentimento / Justificativa de Ausência	TCLE_PARTICIPANTES_QUESTIONARIO_ETAPA_2.pdf	24/04/2017 16:17:45	Camila Eugenia Roseira	Aceito
TCLE / Termos de Assentimento / Justificativa de Ausência	TCLE_JUIZES_ETAPA_1.pdf	24/04/2017 16:17:25	Camila Eugenia Roseira	Aceito
Projeto Detalhado / Brochura Investigador	Projeto_detalhado_camila_2017.pdf	24/04/2017 16:16:56	Camila Eugenia Roseira	Aceito
Folha de Rosto	Camila.pdf	24/04/2017 16:16:22	Camila Eugenia Roseira	Aceito

Situação do Parecer:
Aprovado

Necessita Apreciação da CONEP:
Não

SAO CARLOS, 26 de Junho de 2017

Assinado por:
Priscilla Hortense
(Coordenador)

UFSCAR - UNIVERSIDADE FEDERAL DE SÃO CARLOS

PARECER CONSUBSTANCIADO DO CEP

DADOS DA EMENDA

Título da Pesquisa: BOAS PRÁTICAS EM MEDICAÇÕES INJETÁVEIS: INTERVENÇÃO EDUCATIVA PARA A EQUIPE DE ENFERMAGEM NO ESTADO DE SÃO PAULO

Pesquisador: Camila Eugenia Roseira

Área Temática:

Versão: 4

CAAE: 68147317.5.0000.5504

Instituição Proponente: Programa de Pós-Graduação em Enfermagem

Patrocinador Principal: Financiamento Próprio

DADOS DO PARECER

Número do Parecer: 4.386.765

Apresentação do Projeto:

Estudo foi aprovado para ser desenvolvido a partir das seguintes etapas: (1) elaboração de questionário pautada em literatura na temática, posteriormente avaliado com fins de validação, por juízes; (2) estudo quantitativo descritivo desenvolvido a partir da distribuição eletrônica do questionário validado na etapa anterior para todos os profissionais de enfermagem inscritos no Conselho Regional de Enfermagem de São Paulo; (3) por meio do uso dos dados obtidos na etapa 2 desenvolver material educativo eletrônico, o qual foi prospectado para ser validado em seu conteúdo e aparência. A presente emenda solicita continuidade do processo de validação do material educativo, especificamente pela população-alvo dele, estudantes e profissionais de enfermagem.Prospecta para esta etapa 20 participantes.

Objetivo da Pesquisa:

Desenvolver e validar estratégia educativa para profissionais de enfermagem visando às boas práticas na administração de medicações injetáveis, aplicáveis em larga escala.

Avaliação dos Riscos e Benefícios:

Estudos dessa natureza oferecem risco mínimo em todas as etapas. O participante poderá sentir-se desconfortável para responder algum item do questionário, bem como cansaço mediante o tempo gasto para responder ao mesmo.

Endereço: WASHINGTON LUIZ KM 235
Bairro: JARDIM GUANABARA **CEP:** 13.565-905
UF: SP **Município:** SAO CARLOS
Telefone: (16)3351-9685 **E-mail:** cephumanos@ufscar.br

Conhecimento do contexto atual das boas práticas de medicações injetáveis no estado de São Paulo, bem como a disponibilização de material educativo embasado no mesmo, disponível eletronicamente, que poderá ser utilizado inclusive pelo profissional que foi participante da pesquisa.

Comentários e Considerações sobre a Pesquisa:

- Etapa 1: Juízes: ter experiências clínicas na área de controle de infecção relacionada à assistência à saúde, ou tecnologia da informação e comunicação aplicada à enfermagem, possuir publicação ou pesquisar sobre o tema, ter conhecimento sobre a construção de questionários ou atuar profissionalmente como enfermeiro e não poderá ter registro ativo no Conselho de Enfermagem de São Paulo.- Etapa 2: ter registro ativo no Conselho Regional de Enfermagem do estado de São Paulo, possuir atividade laboral relacionada à enfermagem, possuir cadastro de e-mail para receber uma mensagem de convite à pesquisa, o Termo de Consentimento Livre e Esclarecido e o questionário validado para responder, ter afinidade com informática e internet. - Etapa 3: Juízes (validação do material educativo) - ter experiências clínicas na área de controle de infecção relacionada à assistência à saúde, ou tecnologia da informação e comunicação aplicada à enfermagem, possuir publicação ou pesquisar sobre o tema, ter conhecimento sobre a construção de questionários ou atuar Profissionalmente como enfermeiro. Público-alvo (avaliação do curso sobre
Boas Práticas com Medicações Injetáveis): ter mais de 18 anos e ser profissional ou estudante de enfermagem.

Considerações sobre os Termos de apresentação obrigatória:

Estão apresentados de forma satisfatória.

Recomendações:

Conclusões ou Pendências e Lista de Inadequações:

Emenda voltada a buscar a validação de material desenvolvido ao longo do estudo, foi adequadamente sustentada e explanada em seus procedimentos. As diretrizes para pesquisas com seres humanos estão consideradas.

Considerações Finais a critério do CEP:

Diante do exposto, o Comitê de ética em pesquisa - CEP, de acordo com as atribuições definidas na Resolução CNS nº 510 de 2016 e 466 de 2012, manifesta-se por considerar "Aprovado" o projeto. A responsabilidade do pesquisador é indelegável e indeclinável e compreende os aspectos

éticos e legais, cabendo-lhe, após aprovação deste Comitê de Ética em Pesquisa: II - conduzir o processo de Consentimento e de Assentimento Livre e Esclarecido; III - apresentar dados solicitados pelo CEP ou pela CONEP a qualquer momento; IV - manter os dados da pesquisa em arquivo, físico ou digital, sob sua guarda e responsabilidade, por um período mínimo de 5 (cinco) anos após o término da pesquisa; V - apresentar no relatório final que o projeto foi desenvolvido conforme delineado, justificando, quando ocorridas, a sua mudança ou interrupção. Este relatório final deverá ser protocolado via notificação na Plataforma Brasil. OBSERVAÇÃO: Nos documentos encaminhados por Notificação NÃO DEVE constar alteração no conteúdo do projeto. Caso o projeto tenha sofrido alterações, o pesquisador deverá submeter uma "EMENDA".

Este parecer foi elaborado baseado nos documentos abaixo relacionados:

Tipo Documento	Arquivo	Postagem	Autor	Situação
Informações Básicas do Projeto	PB_INFORMAÇÕES_BÁSICAS_1628945_E1.pdf	19/10/2020 04:02:26		Aceito
Outros	carta.pdf	19/10/2020 03:57:26	Camila Eugenia Roseira	Aceito
Outros	Questionario_avaliacao.pdf	19/10/2020 03:55:49	Camila Eugenia Roseira	Aceito
Brochura Pesquisa	Projeto_detalhado_emenda.pdf	19/10/2020 03:52:21	Camila Eugenia Roseira	Aceito
TCLE / Termos de Assentimento / Justificativa de Ausência	TCLE.pdf	10/09/2020 14:53:02	Camila Eugenia Roseira	Aceito
Declaração de Instituição e Infraestrutura	Anuencia.pdf	13/06/2017 10:33:33	Rosely Moralez de Figueiredo	Aceito
TCLE / Termos de Assentimento / Justificativa de Ausência	TCLE_JUIZES_ETAPA_3.pdf	24/04/2017 16:18:08	Camila Eugenia Roseira	Aceito
TCLE / Termos de Assentimento / Justificativa de Ausência	TCLE_PARTICIPANTES_QUESTIONARIO_ETAPA_2.pdf	24/04/2017 16:17:45	Camila Eugenia Roseira	Aceito
TCLE / Termos de Assentimento / Justificativa de Ausência	TCLE_JUIZES_ETAPA_1.pdf	24/04/2017 16:17:25	Camila Eugenia Roseira	Aceito
Projeto Detalhado	Projeto_detalhado_camila_2017.pdf	24/04/2017	Camila Eugenia	Aceito

/ Brochura Investigador	Projeto_detalhado_camila_2017.pdf	16:16:56	Roseira	Aceito
Folha de Rosto	Camila.pdf	24/04/2017 16:16:22	Camila Eugenia Roseira	Aceito

Situação do Parecer:
Aprovado

Necessita Apreciação da CONEP:
Não

SAO CARLOS, 09 de Novembro de 2020

Assinado por:
ADRIANA SANCHES GARCIA DE ARAUJO
(Coordenador(a))
